# Supplementary material for: The CB1 receptor interacts with cereblon and drives cereblon deficiency-associated memory shortfalls
Source: EMBO Mol Med. 2024 Mar 21;16(4):11. doi: 10.1038/s44321-024-00054-w (PMC11018632; doi:10.1038/s44321-024-00054-w)
Supplement: Supplementary file 1 — Appendix [file 44321_2024_54_MOESM1_ESM.pdf]

APPENDIX

The CB_1 receptor interacts with cereblon and drives cereblon deficiency-associated memory shortfalls

Carlos Costas-Insua[1,2,3,#], Alba Hermoso-López[1,2,3,#], Estefanía Moreno[4,§],
Carlos Montero-Fernández[1,2,3,§], Alicia Álvaro-Blázquez[1,3], Irene B. Maroto[1,2,3],
Andrea Sánchez-Ruiz[5], Rebeca Diez-Alarcia[6,7,8], Cristina Blázquez[1,2,3], Paula Morales[9],
Enric I. Canela[4], Vicent Casadó[4], Leyre Urigüen[6,7,8], Gertrudis Perea[5],
Luigi Bellocchio[10], Ignacio Rodríguez-Crespo[1,2,3], Manuel Guzmán[1,2,3,*]

[1]Department of Biochemistry and Molecular Biology, Instituto Universitario de Investigación Neuroquímica (IUIN), Complutense University, 28040 Madrid, Spain.
[2]Centro de Investigación Biomédica en Red de Enfermedades Neurodegenerativas (CIBERNED), Instituto de Salud Carlos III, 28029 Madrid, Spain.
[3]Instituto Ramón y Cajal de Investigación Sanitaria (IRYCIS), 28034 Madrid, Spain.
[4]Department of Biochemistry and Molecular Biomedicine, Faculty of Biology and Institute of Biomedicine of the University of Barcelona, University of Barcelona, 08028 Barcelona, Spain.
[5]Cajal Institute, CSIC, 28002 Madrid, Spain.
[6]Department of Pharmacology, University of the Basque Country/Euskal Herriko Unibertsitatea, 48940 Leioa, Spain.
[7]Centro de Investigación Biomédica en Red de Salud Mental (CIBERSAM), 28029 Madrid, Spain.
[8]BioBizkaia Health Research Institute, 48903 Barakaldo, Bizkaia, Spain.
[9]Instituto de Química Médica, CSIC, 28006 Madrid, Spain.
[10]Institut National de la Santé et de la Recherche Médicale (INSERM) and University of Bordeaux, NeuroCentre Magendie, Physiopathologie de la Plasticité Neuronale, U1215, 33077 Bordeaux, France.

[#]Contributed equally as first authors
[§]Contributed equally as third authors
*Corresponding author

Table of contents

1. Appendix Table S1
Statistical analysis of potential sex differences in the behavioural tests…………………2

CRBN-KO *vs*. CRBN-WT

Figure 2	CRBN-WT		p (Males *vs*. Females)	CRBN-KO		p (Males *vs*. Females)
Behavioural test	Males mean ± SEM	Females mean ± SEM		Males mean ± SEM	Females mean ± SEM	
Body weight (g)	21.93 ± 0.9563 (n = 12)	19.26 ± 0.3352 (n = 12)	p = 0.0252	21.91 ± 0.5847 (n = 12)	19.08 ± 0.5152 (n = 12)	p = 0.0160
Body temperature (˚C)	35.62 ± 0.3441 (n = 10)	36.37 ± 0.3004 (n = 10)	p = 0.2459	35.80 ± 0.2413 (n = 10)	35.82 ± 0.2112 (n = 10)	p > 0.9999
Total distance in open field (m)	62.75 ± 4.187 (n = 9)	70.59 ± 5.270 (n = 9)	p = 0.6463	66.68 ± 5.920 (n = 8)	66.41 ± 3.710 (n = 7)	p > 0.9999
Latency to fall from rotarod (s)	79.22 ± 20.38 (n = 9)	75.24 ± 13.84 (n = 9)	p > 0.9999	87.61 ± 13.90 (n = 9)	92.92 ± 27.22 (n = 6)	p = 0.6367
Time in open arms of EPM (%)	3.419 ± 1.132 (n = 7)	4.157 ± 0.8967 (n = 6)	p = 0.9391	2.318 ± 0.5576 (n = 6)	3.638 ± 1.000 (n = 5)	p = 0.7835
Time sniffing the object in the social interaction maze (s)	416.8 ± 55.36 (n = 5)	416.5 ± 61.12 (n = 6)	p > 0.9999	470.6 ± 55.19 (n = 7)	476.7 ± 133.7 (n = 3)	p > 0.9999
Time sniffing the mouse in the social interaction maze (s)	1300 ± 68.94 (n = 5)	1449 ± 44.06 (n = 6)	p = 0.7766	1243 ± 144.5 (n = 7)	1297 ± 124.4 (n = 3)	p = 0.9903
Immobility time during FST (s)	151.3 ± 9.162 (n = 7)	150.2 ± 13.17 (n = 5)	p > 0.9999	139.0 ± 20.36 (n = 7)	149.4 ± 32.74 (n = 3)	p = 0.9832
Discrimination index in NOR (%)	0.1667 ± 0.057 (n = 6)	0.2108 ± 0.0415 (n = 6)	p = 0.9198	0.080 ± 0.020 (n = 8)	0.002 ± 0.070 (n = 6)	p = 0.6347
Distance in the familiar arm in spatial Y-maze (m)	6.055 ± 0.4568 (n = 18)	4.923 ± 0.2978 (n = 8)	p = 0.5290	7.024 ± 0.6144 (n = 14)	6.144 ± 0.8007 (n = 7)	p = 0.7658
Distance in the novel arm in spatial Y-maze (m)	6.908 ± 0.4388 (n = 18)	7.311 ± 0.9454 (n = 8)	p = 0.9653	7.358 ± 0.4945 (n = 14)	6.706 ± 0.7235 (n = 7)	p = 0.8975
Freezing time in day 2 of FC (%)	31.09 ± 7.439 (n = 5)	26.06 ± 5.681 (n = 5)	p = 0.9077	13.41 ± 5.240 (n = 3)	18.07 ± 2.915 (n = 7)	p = 0.9405

Figure EV2	CRBN-WT		p (Males *vs*. Females)	CRBN-KO		p (Males *vs*. Females)
Behavioural test	Males mean ± SEM	Females mean ± SEM		Males mean ± SEM	Females mean ± SEM	
Stride length (cm)	6.364 ± 0.1407 (n = 7)	6.628 ± 0.2159 (n = 6)	p = 0.7313	6.787 ± 0.1983 (n = 6)	5.934 ± 0.2625 (n = 2)	p = 0.1400
Normalized entries in centre in open field	0.3248 ± 0.039 (n = 9)	0.2504 ± 0.050 (n = 9)	p = 0.5192	0.2978 ± 0.029 (n = 9)	0.2572 ± 0.031 (n = 6)	p = 0.9059
Latency to pain symptoms in the hot plate test (s)	18.48 ± 2.599 (n = 9)	19.96 ± 2.341 (n = 9)	p = 0.9548	18.23 ± 1.294 (n = 9)	17.35 ± 1.562 (n = 6)	p = 0.9925
Freezing time in day 1 of FC (%)	28.77 ± 7.018 (n = 5)	32.44 ± 4.277 (n = 5)	p = 0.9730	24.91 ± 9.529 (n = 3)	29.84 ± 5.034 (n = 7)	p = 0.9518
Latency to fall from rotarod day 1 (s)	32.93 ± 8.93 (n = 9)	29.07 ± 4.59 (n = 9)	p = 0.9881	36.44 ± 12.29 (n = 9)	22.72 ± 3.80 (n = 6)	p = 0.7342
Latency to fall from rotarod day 2 (s)	65.67 ± 21.19 (n = 9)	53.48 ± 9.82 (n = 9)	p = 0.9765	77.04 ± 28.92 (n = 9)	81.44 ± 23.37 (n = 6)	p = 0.9992
Latency to fall from rotarod day 3 (s)	92.78 ± 20.84 (n = 9)	97.00 ± 18.68 (n = 9)	p = 0.9991	98.19 ± 24.85 (n = 9)	104.4 ± 31.50 (n = 6)	p = 0.9980

Figure 5	CRBN-WT + Vehicle		p (Males *vs*. Females)	CRBN-WT + THC		p (Males *vs*. Females)
Behavioural test	Males mean ± SEM	Females mean ± SEM		Males mean ± SEM	Females mean ± SEM	
Time immobile in the catalepsy test (3 mg/kg; s)	2.900 ± 0.781 (n = 10)	3.000 ± 1.309 (n = 7)	p > 0.9999	3.182 ± 1.292 (n = 11)	4.600 ± 1.887 (n = 5)	p = 0.9961
Latency to pain symptoms in the hot plate test (3 mg/kg; s)	14.31 ± 1.693 (n = 10)	16.64 ± 2.215 (n = 7)	p = 0.9081	19.05 ± 1.950 (n = 11)	17.88 ± 4.140 (n = 5)	p = 0.9945
Time immobile in the catalepsy test (10 mg/kg; s)	1.400 ± 0.872 (n = 5)	2.250 ± 2.250 (n = 4)	p > 0.9999	34.80 ± 10.31 (n = 5)	40.00 ± 20.00 (n = 2)	p = 0.9862
Latency to pain symptoms in the hot plate test (10 mg/kg; s)	13.33 ± 2.043 (n = 4)	16.63 ± 1.120 (n = 4)	p = 0.7959	20.55 ± 1.433 (n = 4)	28.00 ± 2.000 (n = 2)	p = 0.2773

	CRBN-KO + Vehicle		p (Males *vs*. Females)	CRBN-KO + THC		p (Males *vs*. Females)
Behavioural test	Males mean ± SEM	Females mean ± SEM		Males mean ± SEM	Females mean ± SEM	
Time immobile in the catalepsy test (3 mg/kg; s)	2.333 ± 0.615 (n = 6)	2.833 ± 0.757 (n = 12)	p > 0.9999	11.86 ± 4.718 (n = 7)	14.91 ± 4.988 (n = 11)	p = 0.7662
Latency to pain symptoms in the hot plate test (3 mg/kg; s)	16.97 ± 1.063 (n = 6)	18.41 ± 1.111 (n = 12)	p = 0.9840	19.61 ± 3.228 (n = 7)	22.40 ± 2.130 (n = 11)	p = 0.8299
Time immobile in the catalepsy test (10 mg/kg; s)	4.750 ± 1.436 (n = 4)	10.00 ± 3.688 (n = 5)	p= 0.9680	57.50 ± 2.500 (n = 4)	51.00 ± 9.000 (n = 5)	p = 0.9327
Latency to pain symptoms in the hot plate test (10 mg/kg; s)	15.08 ± 2.362 (n = 4)	18.84 ± 3.285 (n = 5)	p = 0.6688	28.28 ± 1.725 (n = 4)	27.68 ± 2.320 (n = 5)	p = 0.9995

Figure 6	CRBN-WT + Vehicle		p (Males *vs*. Females)	CRBN-WT + Rimo		p (Males *vs*. Females)
Behavioural test	Males mean ± SEM	Females mean ± SEM		Males mean ± SEM	Females mean ± SEM	
Discrimination index in NOR (%)	0.1750 ± 0.088 (n = 4)	0.2570 ± 0.049 (n = 7)	p = 0.7966	0.1802 ± 0.049 (n = 5)	0.1626 ± 0.024 (n = 4)	p = 0.9994
Freezing time in day 2 of FC (%)	41.26 ± 6.205 (n = 8)	32.14 ± 5.538 (n = 9)	p = 0.6953	32.69 ± 7.173 (n = 5)	22.19 ± 4.414 (n = 6)	p = 0.7529
Distance in the familiar arm in spatial Y-maze (m)	3.482 ± 0.624 (n = 4)	4.859 ± 0.661 (n = 7)	p = 0.3560	6.323 ± 0.578 (n = 4)	6.246 ± 0.217 (n = 5)	p > 0.9999
Distance in the novel arm in spatial Y-maze (m)	5.138 ± 0.527 (n = 4)	8.554 ± 0.907 (n = 7)	p = 0.0048	7.048 ± 0.120 (n = 4)	9.919 ± 0.732 (n = 5)	p = 0.0344

	CRBN-KO + Vehicle		p (Males *vs*. Females)	CRBN-KO + Rimo		p (Males *vs*. Females)
Behavioural test	Males mean ± SEM	Females mean ± SEM		Males mean ± SEM	Females mean ± SEM	
Discrimination index in NOR (%)	0.034 ± 0.100 (n = 4)	0.061 ± 0.055 (n = 7)	p = 0.9960	0.2239 ± 0.054 (n = 4)	0.2003 ± 0.036 (n = 5)	p = 0.9981
Freezing time in day 2 of FC (%)	19.11 ± 5.464 (n = 7)	13.51 ± 3.675 (n = 5)	p = 0.9630	42.97 ± 2.250 (n = 2)	47.58 ± 12.00 (n = 5)	p = 0.9953
Distance in the familiar arm in spatial Y-maze (m)	5.219 ± 0.533 (n = 6)	6.954 ± 0.545 (n = 7)	p = 0.0917	4.461 ± 0.529 (n = 4)	4,183 ± 0.692 (n = 3)	p = 0.9978
Distance in the novel arm in spatial Y-maze (m)	5.856 ± 0.472 (n = 6)	5.665 ± 0.650 (n = 7)	p = 0.9991	6.126 ± 0.177 (n = 4)	7.384 ± 0.498 (n = 3)	p = 0.7461

p values were obtained by one-way ANOVA with Tukey's post-hoc test (Figs 2 and EV2) or by two-way ANOVA with Sidak's post-hoc test (Figs 5 and 6)

Glu-CRBN-KO *vs*. CRBN-WT

Figure 2	Glu-CRBN-WT		p (Males *vs*. Females)	Glu-CRBN-KO		p (Males *vs*. Females)
**Behavioural test**	**Males mean ± SEM**	**Females mean ± SEM**		**Males mean ± SEM**	**Females mean ± SEM**	
Body weight (g)	23.81 ± 0.8411 (n = 12)	18.15 ± 0.7517 (n = 12)	p < 0.0001	21.72 ± 0.9065 (n = 12)	18.33 ± 0.5092 (n = 12)	p = 0.0160
Body temperature (˚C)	36.65 ± 0.1797 (n = 10)	36.25 ± 0.2701 (n = 10)	p = 0.5905	35.77 ± 0.2290 (n = 10)	36.47 ± 0.2055 (n = 10)	p = 0.1387
Total distance in open field (m)	59.90 ± 4.185 (n = 11)	63.38 ± 3.475 (n = 11)	p = 0.9257	55.11 ± 3.329 (n = 13)	65.96 ± 4.186 (n = 10)	p = 0.1829
Latency to fall from rotarod (s)	122.15 ± 20.48 (n = 11)	124.50 ± 21.57 (n = 9)	p = 0.9740	136.65 ± 15.50 (n = 14)	127.40 ± 21.90 (n = 10)	p = 0.9982
Time in open arms of EPM (%)	4.285 ± 1.275 (n = 8)	4.584 ± 1.443 (n = 11)	p = 0.9977	3.069 ± 0.6448 (n = 13)	1.854 ± 0.7958 (n = 5)	p = 0.9094
Time sniffing the object in the social interaction maze (s)	527.8 ± 84.50 (n = 11)	562.3 ± 99.74 (n = 11)	p = 0.9925	452.4 ± 72.33 (n = 14)	637.8 ± 119.0 (n = 6)	p = 0.5696
Time sniffing the mouse in the social interaction maze (s)	1051 ± 125.2 (n = 11)	937.4 ± 80.38 (n = 11)	p = 0.9162	1023 ± 136.5 (n = 14)	1139 ± 144.9 (n = 6)	p = 0.9383
Immobility time during FST (s)	144.7 ± 8.020 (n = 11)	143.9 ± 14.40 (n = 11)	p > 0.9999	140.5 ± 7.901 (n = 14)	129.6 ± 19.26 (n = 6)	p = 0.9311
Discrimination index in NOR (%)	0.2162 ± 0.041 (n = 10)	0.1748 ± 0.046 (n = 7)	p = 0.9592	0.056 ± 0.060 (n = 12)	0.049 ± 0.1200 (n = 3)	p > 0.9999
Distance in the familiar arm in spatial Y-maze (m)	5.550 ± 0.5271 (n = 13)	4.885 ± 0.4407 (n = 19)	p = 0.7759	5.726 ± 0.5364 (n = 14)	6.408 ± 0.5131 (n = 14)	p = 0.7884
Distance in the novel arm in spatial Y-maze (m)	6.873 ± 0.5750 (n = 13)	6.864 ± 0.4551 (n = 19)	p > 0.9999	5.910 ± 0.5171 (n = 14)	6.647 ± 0.3785 (n = 14)	p = 0.7274
Freezing time in day 2 of FC (%)	21.24 ± 2.904 (n = 12)	21.42 ± 2.783 (n = 12)	p > 0.9999	7.736 ± 2.524 (n = 12)	6.500 ± 1.923 (n = 12)	p = 0.9861

Figure EV2	Glu-CRBN-WT		p (Males *vs*. Females)	Glu-CRBN-KO		p (Males *vs*. Females)
**Behavioural test**	**Males mean ± SEM**	**Females mean ± SEM**		**Males mean ± SEM**	**Females mean ± SEM**	
Stride length (cm)	6.581 ± 0.1629 (n = 7)	6.034 ± 0.2157 (n = 10)	p = 0.4564	6.497 ± 0.2225 (n = 10)	5.848 ± 0.8805 (n = 3)	p = 0.5564
Normalized entries in centre in open field	0.2172 ± 0.022 (n = 11)	0.2860 ± 0.022 (n = 9)	p = 0.2182	0.2209 ± 0.022 (n = 13)	0.1955 ± 0.038 (n = 6)	p = 0.9102
Latency to pain symptoms in the hot plate test (s)	16.66 ± 2.182 (n = 10)	18.31 ± 1.627 (n = 10)	p = 0.9275	14.62 ± 1.818 (n = 14)	13.88 ± 0.3720 (n = 5)	p = 0.9953
Freezing time in day 1 of FC (%)	20.04 ± 2.107 (n = 12)	24.48 ± 2.144 (n = 12)	p = 0.6742	18.62 ± 2.329 (n = 12)	20.34 ± 4.063 (n = 12)	p = 0.9718
Latency to fall from rotarod day 1 (s)	60.61 ± 11.58 (n = 11)	70.48 ± 18.03 (n = 9)	p = 0.9745	67.93 ± 16.92 (n = 14)	62.30 ± 14.37 (n = 10)	p = 0.9937
Latency to fall from rotarod day 2 (s)	113.1 ± 21.21 (n = 11)	105.4 ± 19.54 (n = 9)	p = 0.9939	130.2 ± 15.78 (n = 14)	109.8 ± 25.21 (n = 10)	p = 0.8827
Latency to fall from rotarod day 3 (s)	131.2 ± 20.91 (n = 11)	143.6 ± 25.45 (n = 9)	p = 0.9782	143.1 ± 18.13 (n = 14)	145.0 ± 20.60 (n = 10)	p > 0.9999

Figure 5	Glu-CRBN-WT + Vehicle		p (Males *vs*. Females)	Glu-CRBN-WT + THC		p (Males *vs*. Females)
**Behavioural test**	**Males mean ± SEM**	**Females mean ± SEM**		**Males mean ± SEM**	**Females mean ± SEM**	
Time immobile in the catalepsy test (3 mg/kg; s)	2.857 ± 1.844 (n = 7)	1.875 ± 0.549 (n = 8)	p = 0.9950	2.375 ± 1.149 (n = 8)	3.167 ± 0.910 (n = 6)	p = 0.9982
Latency to pain symptoms in the hot plate test (3 mg/kg; s)	11.90 ± 1.831 (n = 7)	10.24 ± 1.489 (n = 8)	p = 0.9599	15.03 ± 1.540 (n = 8)	14.10 ± 2.863 (n = 6)	p = 0.9962
Time immobile in the catalepsy test (10 mg/kg; s)	0.500 ± 0.500 (n = 4)	3.000 ± 1.732 (n = 3)	p = 0.9995	48.00 ± 12.00 (n = 5)	29.67 ± 7.753 (n = 3)	p = 0.5031
Latency to pain symptoms in the hot plate test (10 mg/kg; s)	14.55 ± 1.333 (n = 4)	13.57 ± 7.269 (n = 3)	p = 0.9996	17.20 ± 3.041 (n = 5)	25.97 ± 2.640 (n = 3)	p = 0.3792

	Glu-CRBN-KO + Vehicle		p (Males *vs*. Females)	Glu-CRBN-KO + THC		p (Males *vs*. Females)
**Behavioural test**	**Males mean ± SEM**	**Females mean ± SEM**		**Males mean ± SEM**	**Females mean ± SEM**	
Time immobile in the catalepsy test (3 mg/kg; s)	1.571 ± 0.812 (n = 7)	1.714 ± 0.680 (n = 7)	p > 0.9999	13.71 ± 4.814 (n = 7)	10.57 ± 2.379 (n = 7)	p = 0.7540
Latency to pain symptoms in the hot plate test (3 mg/kg; s)	10.23 ± 1.304 (n = 7)	12.29 ± 1.360 (n = 7)	p = 0.9253	14.89 ± 3.123 (n = 7)	16.77 ± 2.244 (n = 7)	p = 0.9444
Time immobile in the catalepsy test (10 mg/kg; s)	8.000 ± 3.941 (n = 6)	4.500 ± 2.102 (n = 4)	p = 0.9965	46.33 ± 8.647 (n = 6)	28.00 ± 17.44 (n = 3)	p = 0.4722
Latency to pain symptoms in the hot plate test (10 mg/kg; s)	11.03 ± 1.900 (n = 6)	10.90 ± 1.744 (n = 4)	p > 0.9999	19.40 ± 4.664 (n = 6)	26.77 ± 2.038 (n = 3)	p = 0.5155

Figure 6	Glu-CRBN-WT + Vehicle		p (Males *vs*. Females)	Glu-CRBN-WT + Rimo		p (Males *vs*. Females)
**Behavioural test**	**Males mean ± SEM**	**Females mean ± SEM**		**Males mean ± SEM**	**Females mean ± SEM**	
Discrimination index in NOR (%)	0.1434 ± 0.037 (n = 13)	0.1697 ± 0.031 (n = 13)	p = 0.9836	0.1401 ± 0.054 (n =13)	0.1868 ± 0.031 (n = 15)	p = 0.8646
Freezing time in day 2 of FC (%)	33.03 ± 7.774 (n = 7)	33.08 ± 10.30 (n = 5)	p > 0.9999	34.60 ± 11.54 (n = 4)	26.60 ± 7.587 (n = 7)	p = 0.9399
Distance in the familiar arm in spatial Y-maze (m)	3.711 ± 0.710 (n = 8)	4.646 ± 0.281 (n = 5)	p = 0.7735	3.818 ± 0.677 (n = 6)	5.292 ± 0.291 (n = 4)	p = 0.5029
Distance in the novel arm in spatial Y-maze (m)	6.860 ± 0.773 (n = 8)	6.968 ± 1.023 (n = 5)	p > 0.9999	6.325 ± 0.936 (n = 6)	7.046 ± 0.851 (n = 4)	p = 0.9630

	Glu-CRBN-KO + Vehicle		p (Males *vs*. Females)	Glu-CRBN-KO + Rimo		p (Males *vs*. Females)
**Behavioural test**	**Males mean ± SEM**	**Females mean ± SEM**		**Males mean ± SEM**	**Females mean ± SEM**	
Discrimination index in NOR (%)	-0.018 ± 0.040 (n = 12)	0.1095 ± 0.025 (n = 9)	p = 0.1770	0.1974 ± 0.040 (n = 12)	0.1433 ± 0.050 (n = 13)	p = 0.8207
Freezing time in day 2 of FC (%)	10.42 ± 2.477 (n = 4)	9.267 ± 2.980 (n = 5)	p > 0.9999	38.61 ± 3.517 (n = 4)	47.33 ± 9.078 (n = 7)	p = 0.9196
Distance in the familiar arm in spatial Y-maze (m)	4.720 ± 0.820 (n = 5)	5.348 ± 0.727 (n = 7)	p = 0.9402	4.349 ± 0.379 (n = 5)	5.189 ± 0.674 (n = 6)	p = 0.8617
Distance in the novel arm in spatial Y-maze (m)	6.738 ± 0.934 (n = 5)	6.268 ± 0.275 (n = 7)	p = 0.9890	8.191 ± 0.433 (n = 5)	6.878 ± 0.900 (n = 6)	p = 0.7042

p values were obtained by one-way ANOVA with Tukey's post-hoc test (Figs 2 and EV2) or by two-way ANOVA with Sidak's post-hoc test (Figs 5 and 6)

GABA-CRBN-KO *vs*. CRBN-WT

Figure 2	GABA-CRBN-WT		p (Males *vs*. Females)	GABA-CRBN-KO		p (Males *vs*. Females)
**Behavioural test**	**Males mean ± SEM**	**Females mean ± SEM**		**Males mean ± SEM**	**Females mean ± SEM**	
Body weight (g)	20.80 ± 0.8965 (n = 12)	17.44 ± 0.5790 (n = 12)	p = 0.0026	20.74 ± 0.4735 (n = 12)	17.65 ± 0.4731 (n = 12)	p = 0.0062
Body temperature (˚C)	36.52 ± 0.1896 (n = 10)	36.71 ± 0.3433 (n = 10)	p = 0.9663	36.94 ± 0.1543 (n = 10)	36.56 ± 0.3964 (n = 10)	p= 0.7897
Total distance in open field (m)	60.76 ± 3.621 (n = 8)	60.48 ± 3.736 (n = 12)	p > 0.9999	64.67 ± 2.464 (n = 9)	65.78 ± 1.683 (n = 12)	p = 0.9936
Latency to fall from rotarod (s)	74.72 ± 16.23 (n = 10)	119.55 ± 14.80 (n = 14)	p = 0.2157	124.72 ± 24.42 (n = 10)	87.07 ± 21.72 (n = 14)	p = 0.5791
Time in open arms of EPM (%)	4.724 ± 1.690 (n = 7)	4.523 ± 1.254 (n = 12)	p = 0.9997	5.954 ± 1.281 (n = 9)	8.354 ± 1.527 (n = 11)	p = 0.6361
Time sniffing the object in the social interaction maze (s)	512.7 ± 36.24 (n = 3)	460.6 ± 42.84 (n = 8)	p = 0.9118	464.0 ± 55.65 (n = 7)	451.4 ± 32.76 (n = 8)	p = 0.9967
Time sniffing the mouse in the social interaction maze (s)	953.7 ± 163.7 (n = 3)	1076 ± 68.38 (n = 8)	p = 0.8949	1215 ± 122.5 (n = 7)	1045 ± 85.35 (n = 8)	p = 0.5856
Immobility time during FST (s)	175.2 ± 16.34 (n = 3)	173.9 ± 7.247 (n = 8)	p > 0.9999	151.5 ± 12.20 (n = 7)	159.2 ± 13.23 (n = 9)	p = 0.9635
Discrimination index in NOR (%)	0.2326 ± 0.061 (n = 7)	0.1319 ± 0.080 (n = 6)	p = 0.6428	0.090 ± 0.049 (n = 10)	0.1632 ± 0.034 (n = 8)	p = 0.7417
Distance in the familiar arm in spatial Y-maze (m)	5.793 ± 0.5823 (n = 11)	5.626 ± 0.7138 (n = 9)	p = 0.9978	4.535 ± 0.4874 (n = 11)	6.160 ± 0.6797 (n = 12)	p = 0.2385
Distance in the novel arm in spatial Y-maze (m)	7.853 ± 0.8075 (n = 11)	8.005 ± 0.7279 (n = 9)	p = 0.9992	7.868 ± 0.8726 (n = 11)	6.967 ± 0.7497 (n = 12)	p = 0.8426
Freezing time in day 2 of FC (%)	20.71 ± 4.553 (n = 10)	28.26 ± 7.618 (n = 5)	p = 0.9940	22.22 ± 4.663 (n = 8)	17.10 ± 3.188 (n = 9)	p = 0.8584

Figure EV2	GABA-CRBN-WT		p (Males *vs*. Females)	GABA-CRBN-KO		p (Males *vs*. Females)
**Behavioural test**	**Males mean ± SEM**	**Females mean ± SEM**		**Males mean ± SEM**	**Females mean ± SEM**	
Stride length (cm)	6.436 ± 0.2938 (n = 2)	5.942 ± 0.1434 (n = 7)	p = 0.6528	6.116 ± 0.2812 (n = 3)	6.040 ± 0.2286 (n = 8)	p = 0.9965
Normalized entries in centre in open field	0.2763 ± 0.030 (n = 8)	0.2458 ± 0.021 (n = 11)	p = 0.8341	0.2347 ± 0.019 (n = 10)	0.2102 ± 0.024 (n = 14)	p = 0.8725
Latency to pain symptoms in the hot plate test (s)	17.42 ± 1.472 (n = 9)	17.50 ± 1.558 (n = 12)	p > 0.9999	16.59 ± 1.882 (n = 10)	18.54 ± 1.615 (n = 14)	p = 0.8330
Freezing time in day 1 of FC (%)	24.71 ± 2.852 (n = 10)	32.49 ± 9.593 (n = 3)	p = 0.6629	11.72 ± 1.570 (n = 5)	25.49 ± 3.896 (n = 9)	p = 0.1048
Latency to fall from rotarod day 1 (s)	46.57 ± 10.02 (n = 10)	55.83 ± 12.05 (n = 14)	p = 0.9326	41.20 ± 6.85 (n = 10)	36.48 ± 11.06 (n = 14)	p = 0.9901
Latency to fall from rotarod day 2 (s)	68.03 ± 18.13 (n = 10)	107.5 ± 14.04 (n = 14)	p = 0.4392	95.87 ± 17.86 (n = 10)	74.05 ± 21.06 (n = 14)	p = 0.8371
Latency to fall from rotarod day 3 (s)	81.40 ± 15.52 (n = 10)	131.6 ± 16.54 (n = 14)	p = 0.4211	153.6 ± 33.41 (n = 10)	100.1 ± 23.40 (n = 14)	p = 0.3652

Figure 5	GABA-CRBN-WT + Vehicle		p (Males *vs*. Females)	GABA-CRBN-WT + THC		p (Males *vs*. Females)
**Behavioural test**	**Males mean ± SEM**	**Females mean ± SEM**		**Males mean ± SEM**	**Females mean ± SEM**	
Time immobile in the catalepsy test (3 mg/kg; s)	2.000 ± 2.000 (n = 3)	0.500 ± 0.500 (n = 4)	p = 0.9534	4.750 ± 1.702 (n = 4)	2.500 ± 2.500 (n = 4)	p = 0.7840
Latency to pain symptoms in the hot plate test (3 mg/kg; s)	17.53 ± 1.368 (n = 3)	15.43 ± 2.616 (n = 4)	p = 0.9107	20.53 ± 1.215 (n = 4)	19.00 ± 1.942 (n = 4)	p = 0.9615
Time immobile in the catalepsy test (10 mg/kg; s)	0.667 ± 0.667 (n = 3)	0.600 ± 0.600 (n = 5)	p > 0.9999	45.33 ± 14.67 (n = 3)	60.00 ± 0.000 (n = 4)	p = 0.5507
Latency to pain symptoms in the hot plate test (10 mg/kg; s)	13.50 ± 3.024 (n = 3)	16.04 ± 2.604 (n = 5)	p = 0.9299	25.00 ± 2.536 (n = 3)	30 ± 0.000 (n = 4)	p = 0.5799

	GABA-CRBN-KO + Vehicle		p (Males *vs*. Females)	GABA-CRBN-KO + THC		p (Males *vs*. Females)
**Behavioural test**	**Males mean ± SEM**	**Females mean ± SEM**		**Males mean ± SEM**	**Females mean ± SEM**	
Time immobile in the catalepsy test (3 mg/kg; s)	0.500 ± 0.500 (n = 4)	4.200 ± 1.319 (n = 5)	p = 0.3135	1.750 ± 1.181 (n = 4)	6.200 ± 1.625 (n = 5)	p = 0.1642
Latency to pain symptoms in the hot plate test (3 mg/kg; s)	18.58 ± 2.262 (n = 4)	15.12 ± 0.832 (n = 5)	p = 0.5199	21.13 ± 2.652 (n = 4)	17.88 ± 0.786 (n = 5)	p = 0.5779
Time immobile in the catalepsy test (10 mg/kg; s)	1.000 ± 1.000 (n = 4)	3.250 ± 1.250 (n = 4)	p = 0.9990	52.00 ± 8.000 (n = 4)	36.00 ± 11.34 (n = 5)	p = 0.3444
Latency to pain symptoms in the hot plate test (10 mg/kg; s)	12.55 ± 2.344 (n = 4)	16.20 ± 1.179 (n = 4)	p = 0.7648	27.40 ± 2.600 (n = 4)	23.36 ± 3.047 (n = 5)	p = 0.6518

p values were obtained by one-way ANOVA with Tukey's post-hoc test (Figs 2 and EV2) or by two-way ANOVA with Sidak's post-hoc test (Fig 5)
