# Supplementary material for: The CB1 receptor interacts with cereblon and drives cereblon deficiency-associated memory shortfalls
Source: EMBO Mol Med. 2024 Mar 21;16(4):11. doi: 10.1038/s44321-024-00054-w (PMC11018632; doi:10.1038/s44321-024-00054-w)
Supplement: Supplementary file 2 — EV Figures Source Data [file 44321_2024_54_MOESM2_ESM.zip › Raw_data_EV_figures/Figure EV1/Figure EV1D/Figure EV1D - uncropped WBs.pptx]

## Slide 1
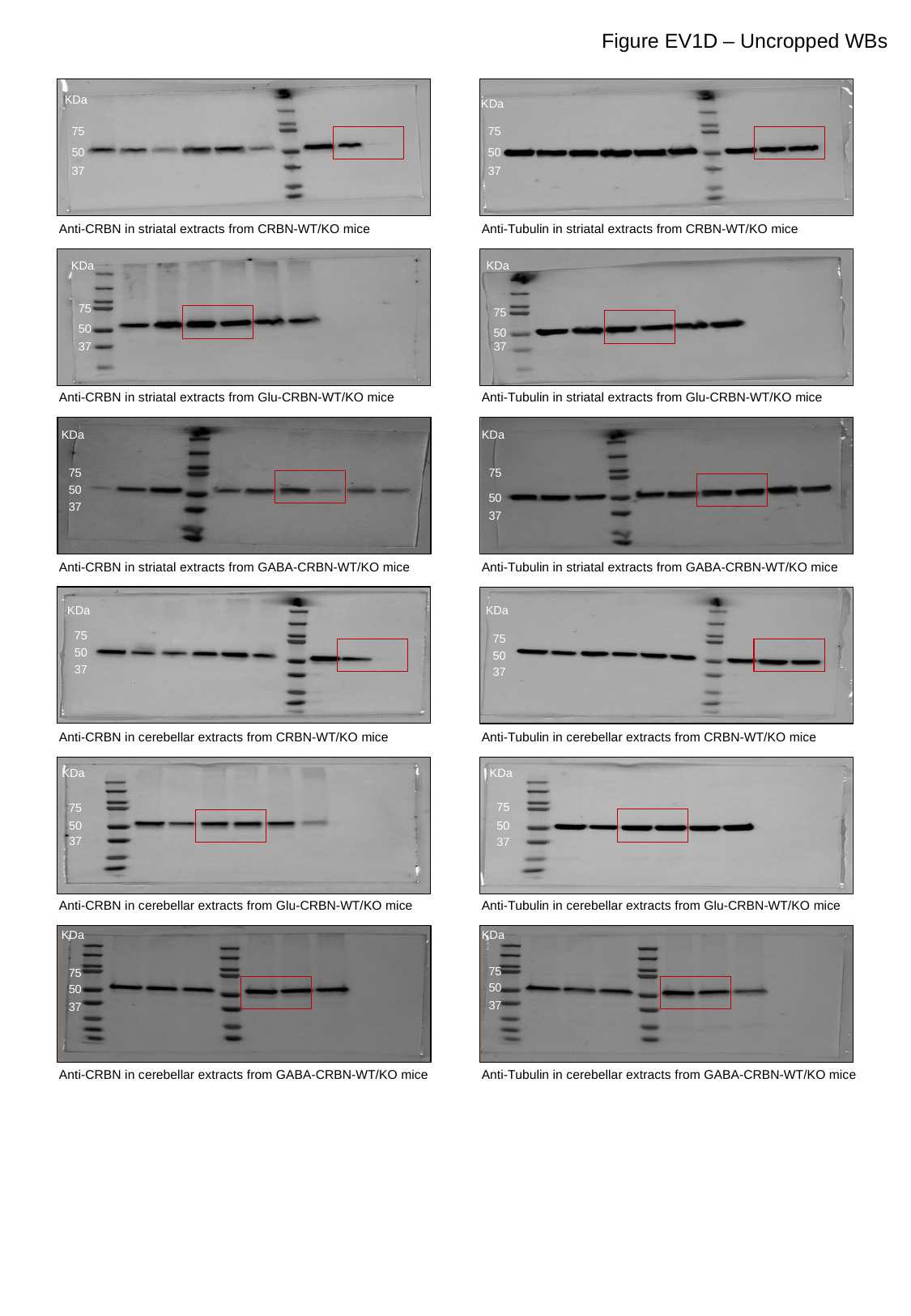

Figure EV1D – Uncropped WBs
KDa
KDa
75
75
50
50
37
37
Anti-CRBN in striatal extracts from CRBN-WT/KO mice
Anti-Tubulin in striatal extracts from CRBN-WT/KO mice
KDa
75
50
37
KDa
75
50
37
Anti-CRBN in striatal extracts from Glu-CRBN-WT/KO mice
Anti-Tubulin in striatal extracts from Glu-CRBN-WT/KO mice
KDa
75
50
37
KDa
75
50
37
Anti-CRBN in striatal extracts from GABA-CRBN-WT/KO mice
Anti-Tubulin in striatal extracts from GABA-CRBN-WT/KO mice
KDa
75
50
37
KDa
75
50
37
Anti-CRBN in cerebellar extracts from CRBN-WT/KO mice
Anti-Tubulin in cerebellar extracts from CRBN-WT/KO mice
KDa
75
50
37
KDa
75
50
37
Anti-CRBN in cerebellar extracts from Glu-CRBN-WT/KO mice
Anti-Tubulin in cerebellar extracts from Glu-CRBN-WT/KO mice
KDa
75
50
37
KDa
75
50
37
Anti-CRBN in cerebellar extracts from GABA-CRBN-WT/KO mice
Anti-Tubulin in cerebellar extracts from GABA-CRBN-WT/KO mice
