# Supplementary material for: The CB1 receptor interacts with cereblon and drives cereblon deficiency-associated memory shortfalls
Source: EMBO Mol Med. 2024 Mar 21;16(4):11. doi: 10.1038/s44321-024-00054-w (PMC11018632; doi:10.1038/s44321-024-00054-w)
Supplement: Supplementary file 2 — EV Figures Source Data [file 44321_2024_54_MOESM2_ESM.zip › Raw_data_EV_figures/Figure EV3/Figure EV3A/Figure EV3A - uncropped WBs.pptx]

## Slide 1
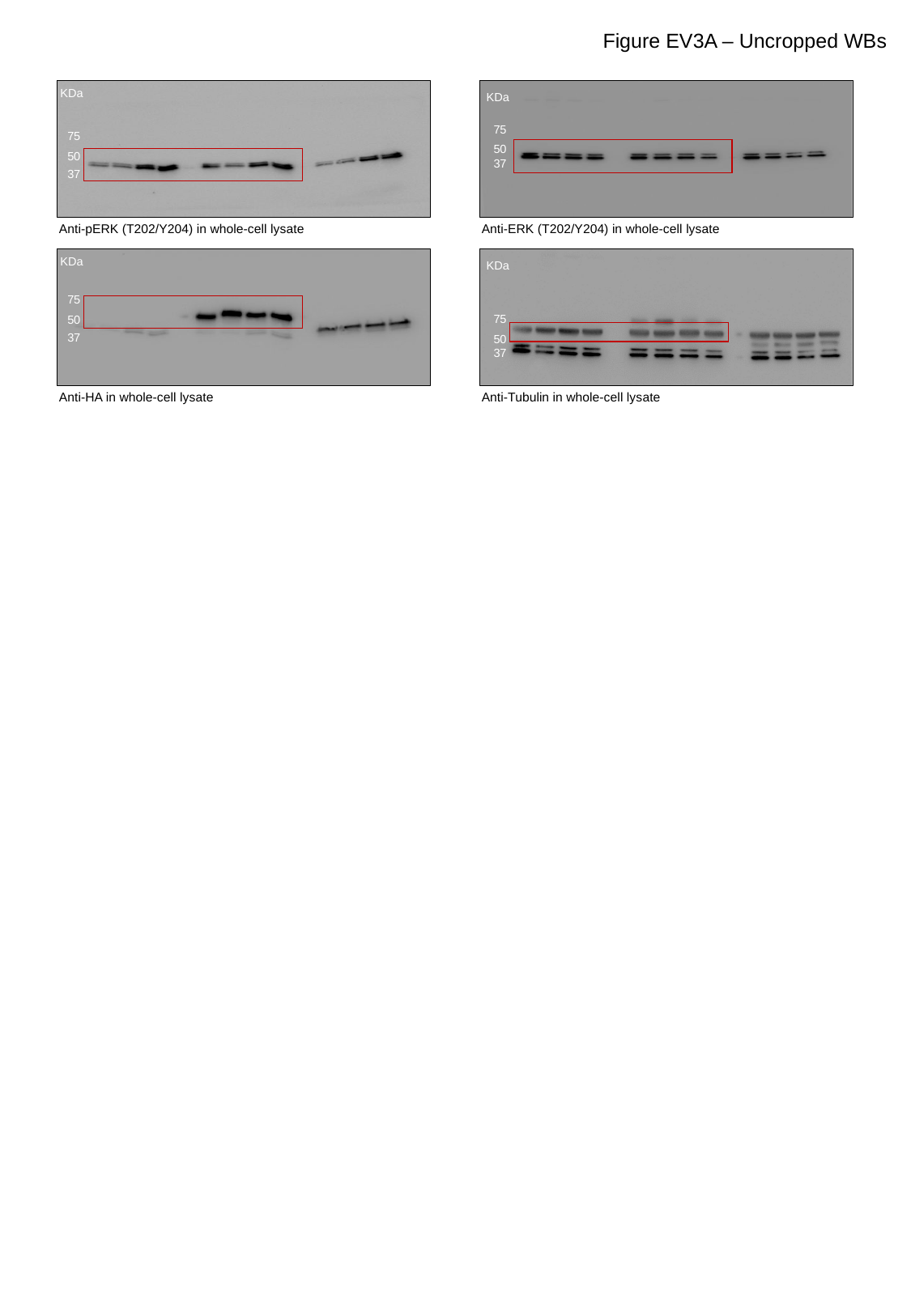

Figure EV3A – Uncropped WBs
KDa
75
50
37
KDa
75
50
37
Anti-pERK (T202/Y204) in whole-cell lysate
Anti-ERK (T202/Y204) in whole-cell lysate
KDa
75
50
37
KDa
75
50
37
Anti-HA in whole-cell lysate
Anti-Tubulin in whole-cell lysate
