# Supplementary material for: The CB1 receptor interacts with cereblon and drives cereblon deficiency-associated memory shortfalls
Source: EMBO Mol Med. 2024 Mar 21;16(4):11. doi: 10.1038/s44321-024-00054-w (PMC11018632; doi:10.1038/s44321-024-00054-w)
Supplement: Supplementary file 2 — EV Figures Source Data [file 44321_2024_54_MOESM2_ESM.zip › Raw_data_EV_figures/Figure EV4/Figure EV4A/Figure EV4A - uncropped WBs.pptx]

## Slide 1
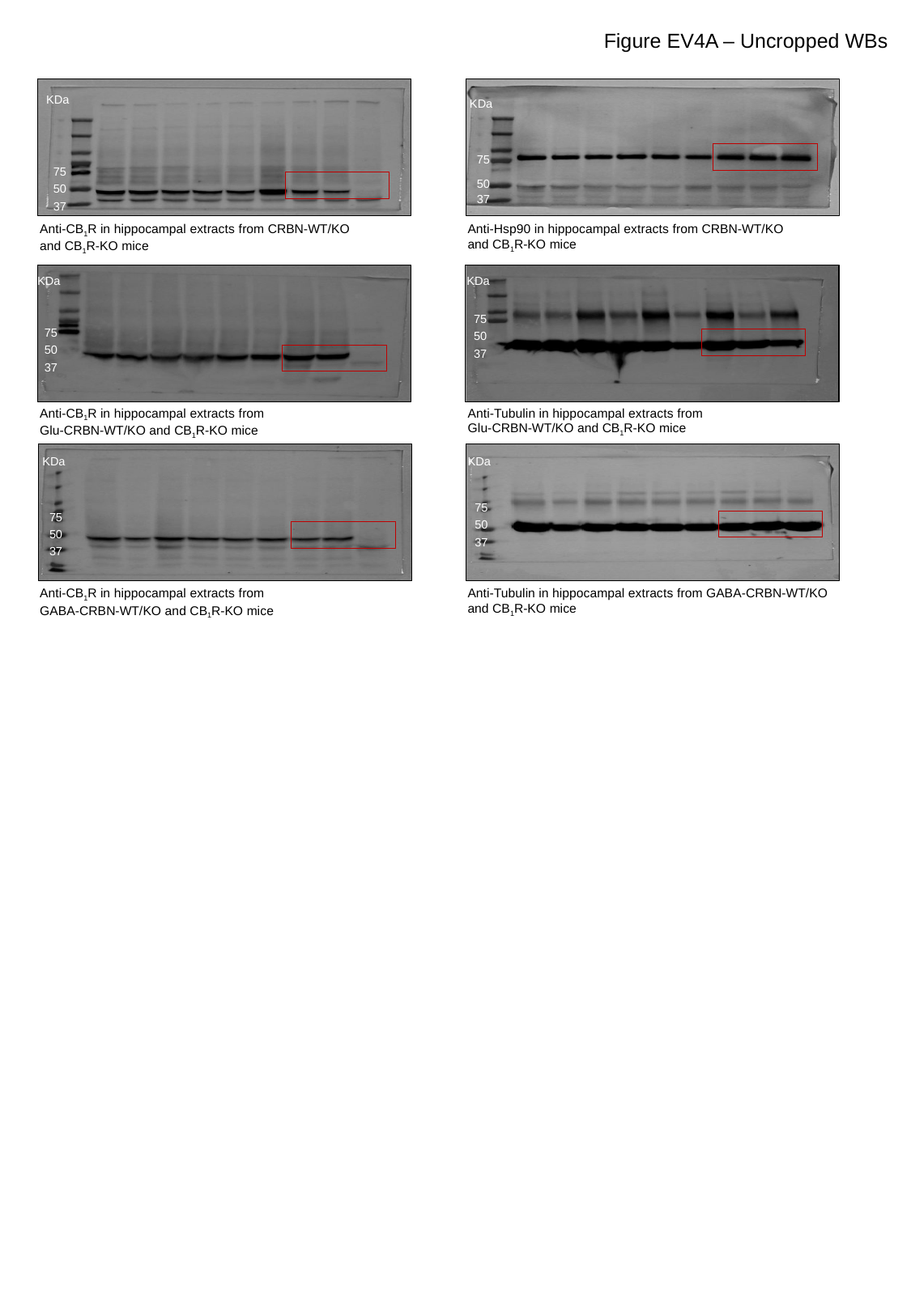

Figure EV4A – Uncropped WBs
KDa
KDa
75
50
37
75
50
37
Anti-CB1R in hippocampal extracts from CRBN-WT/KO and CB1R-KO mice
Anti-Hsp90 in hippocampal extracts from CRBN-WT/KO and CB1R-KO mice
KDa
KDa
75
50
37
75
50
37
Anti-CB1R in hippocampal extracts from Glu-CRBN-WT/KO and CB1R-KO mice
Anti-Tubulin in hippocampal extracts from Glu-CRBN-WT/KO and CB1R-KO mice
KDa
KDa
75
50
37
75
50
37
Anti-CB1R in hippocampal extracts from GABA-CRBN-WT/KO and CB1R-KO mice
Anti-Tubulin in hippocampal extracts from GABA-CRBN-WT/KO and CB1R-KO mice
