# Supplementary material for: The CB1 receptor interacts with cereblon and drives cereblon deficiency-associated memory shortfalls
Source: EMBO Mol Med. 2024 Mar 21;16(4):11. doi: 10.1038/s44321-024-00054-w (PMC11018632; doi:10.1038/s44321-024-00054-w)
Supplement: Supplementary file 2 — EV Figures Source Data [file 44321_2024_54_MOESM2_ESM.zip › Raw_data_EV_figures/Figure EV4/Figure EV4C/Figure EV4C - uncropped WBs.pptx]

## Slide 1
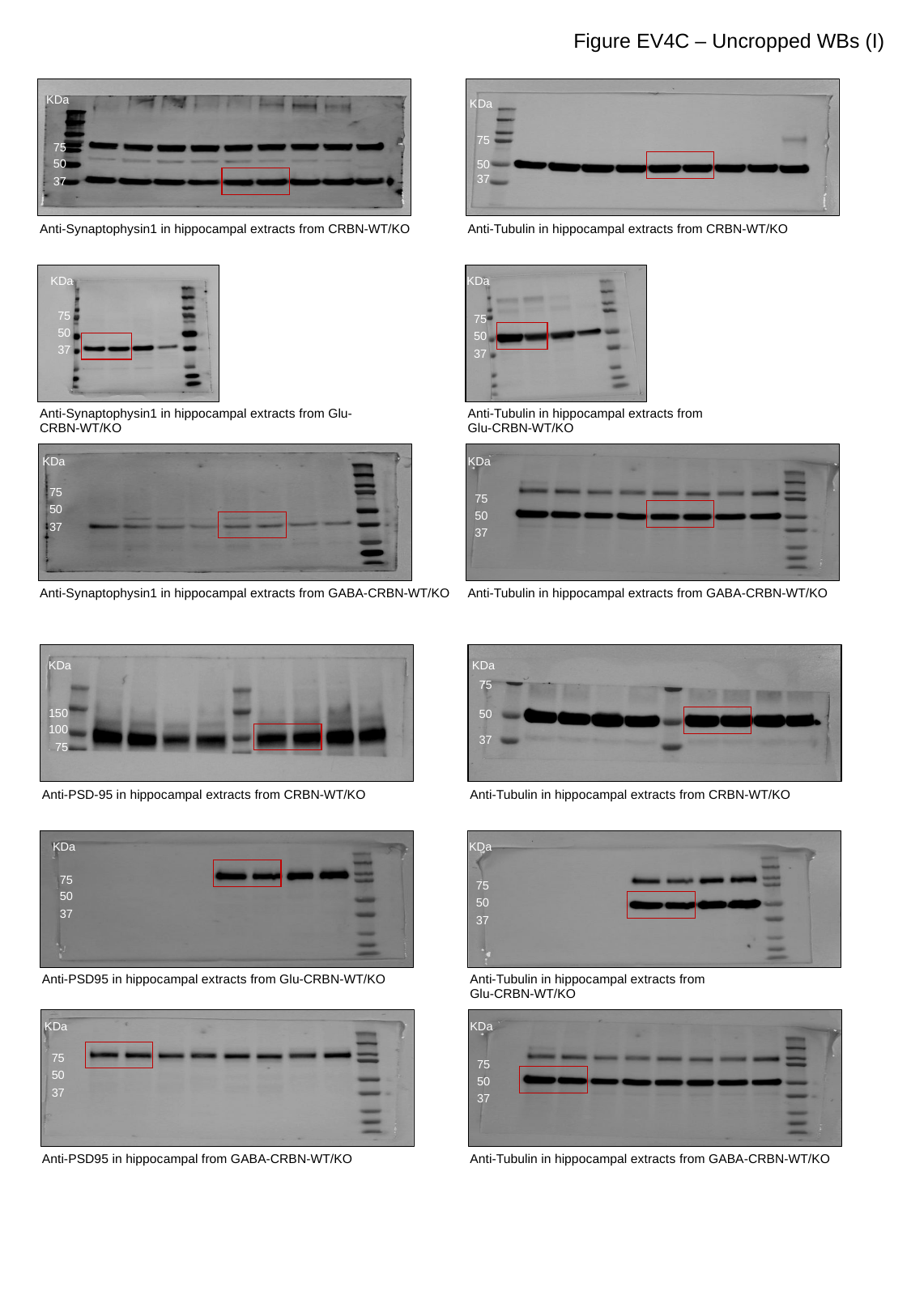

Figure EV4C – Uncropped WBs (I)
KDa
KDa
75
50
37
75
50
37
Anti-Synaptophysin1 in hippocampal extracts from CRBN-WT/KO
Anti-Tubulin in hippocampal extracts from CRBN-WT/KO
KDa
KDa
75
50
37
75
50
37
Anti-Synaptophysin1 in hippocampal extracts from Glu-CRBN-WT/KO
Anti-Tubulin in hippocampal extracts from Glu-CRBN-WT/KO
KDa
KDa
75
50
37
75
50
37
Anti-Synaptophysin1 in hippocampal extracts from GABA-CRBN-WT/KO
Anti-Tubulin in hippocampal extracts from GABA-CRBN-WT/KO
KDa
KDa
75
50
37
150
100
75
Anti-PSD-95 in hippocampal extracts from CRBN-WT/KO
Anti-Tubulin in hippocampal extracts from CRBN-WT/KO
KDa
KDa
75
50
37
75
50
37
Anti-PSD95 in hippocampal extracts from Glu-CRBN-WT/KO
Anti-Tubulin in hippocampal extracts from Glu-CRBN-WT/KO
KDa
KDa
75
50
37
75
50
37
Anti-PSD95 in hippocampal from GABA-CRBN-WT/KO
Anti-Tubulin in hippocampal extracts from GABA-CRBN-WT/KO

## Slide 2
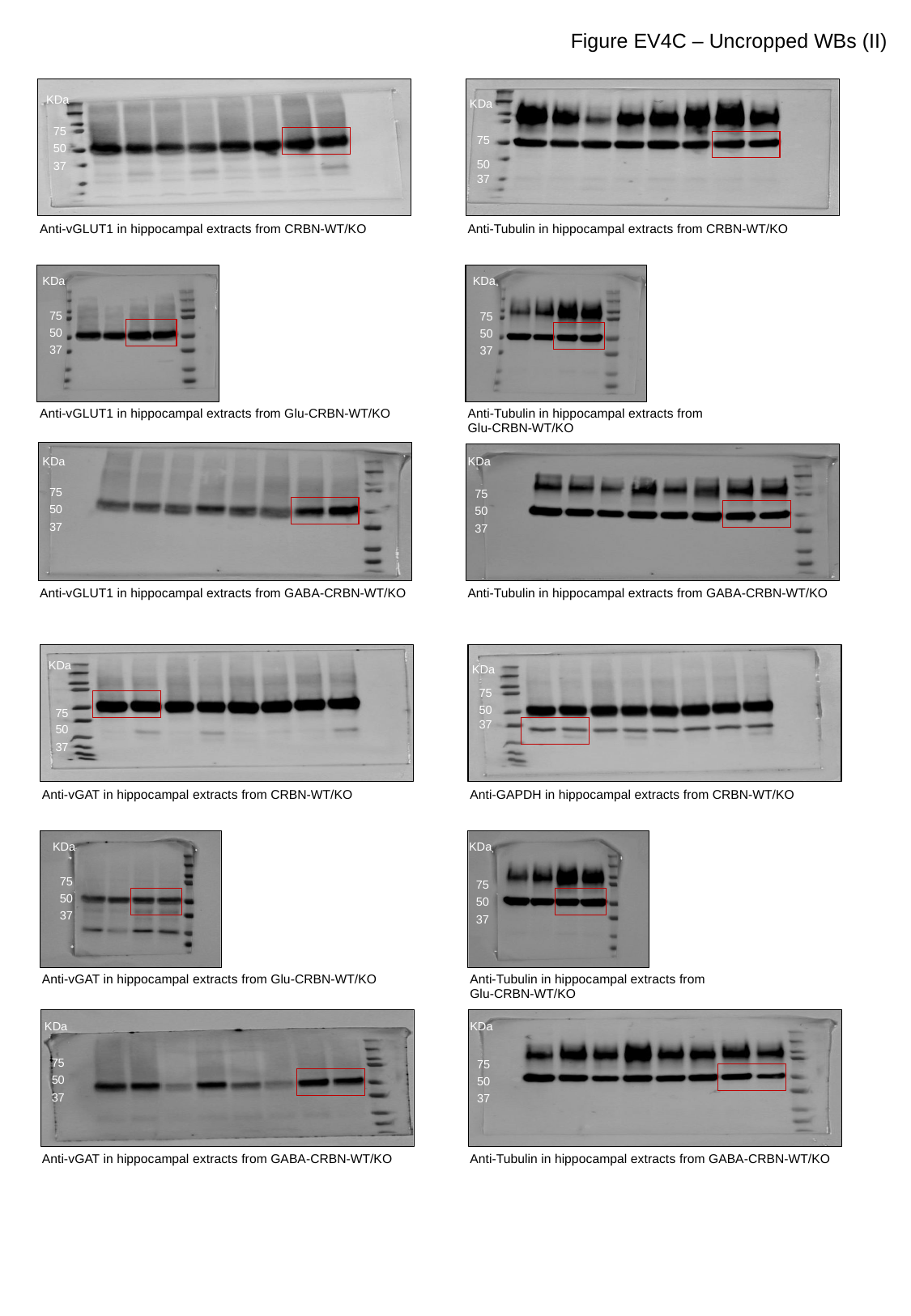

Figure EV4C – Uncropped WBs (II)
KDa
KDa
75
50
37
75
50
37
Anti-vGLUT1 in hippocampal extracts from CRBN-WT/KO
Anti-Tubulin in hippocampal extracts from CRBN-WT/KO
KDa
KDa
75
50
37
75
50
37
Anti-vGLUT1 in hippocampal extracts from Glu-CRBN-WT/KO
Anti-Tubulin in hippocampal extracts from Glu-CRBN-WT/KO
KDa
KDa
75
50
37
75
50
37
Anti-vGLUT1 in hippocampal extracts from GABA-CRBN-WT/KO
Anti-Tubulin in hippocampal extracts from GABA-CRBN-WT/KO
KDa
KDa
75
50
37
75
50
37
Anti-vGAT in hippocampal extracts from CRBN-WT/KO
Anti-GAPDH in hippocampal extracts from CRBN-WT/KO
KDa
KDa
75
50
37
75
50
37
Anti-vGAT in hippocampal extracts from Glu-CRBN-WT/KO
Anti-Tubulin in hippocampal extracts from Glu-CRBN-WT/KO
KDa
KDa
75
50
37
75
50
37
Anti-vGAT in hippocampal extracts from GABA-CRBN-WT/KO
Anti-Tubulin in hippocampal extracts from GABA-CRBN-WT/KO
