# Supplementary figures and images for: The CB1 receptor interacts with cereblon and drives cereblon deficiency-associated memory shortfalls
Source: EMBO Mol Med. 2024 Mar 21;16(4):11. doi: 10.1038/s44321-024-00054-w (PMC11018632; doi:10.1038/s44321-024-00054-w)

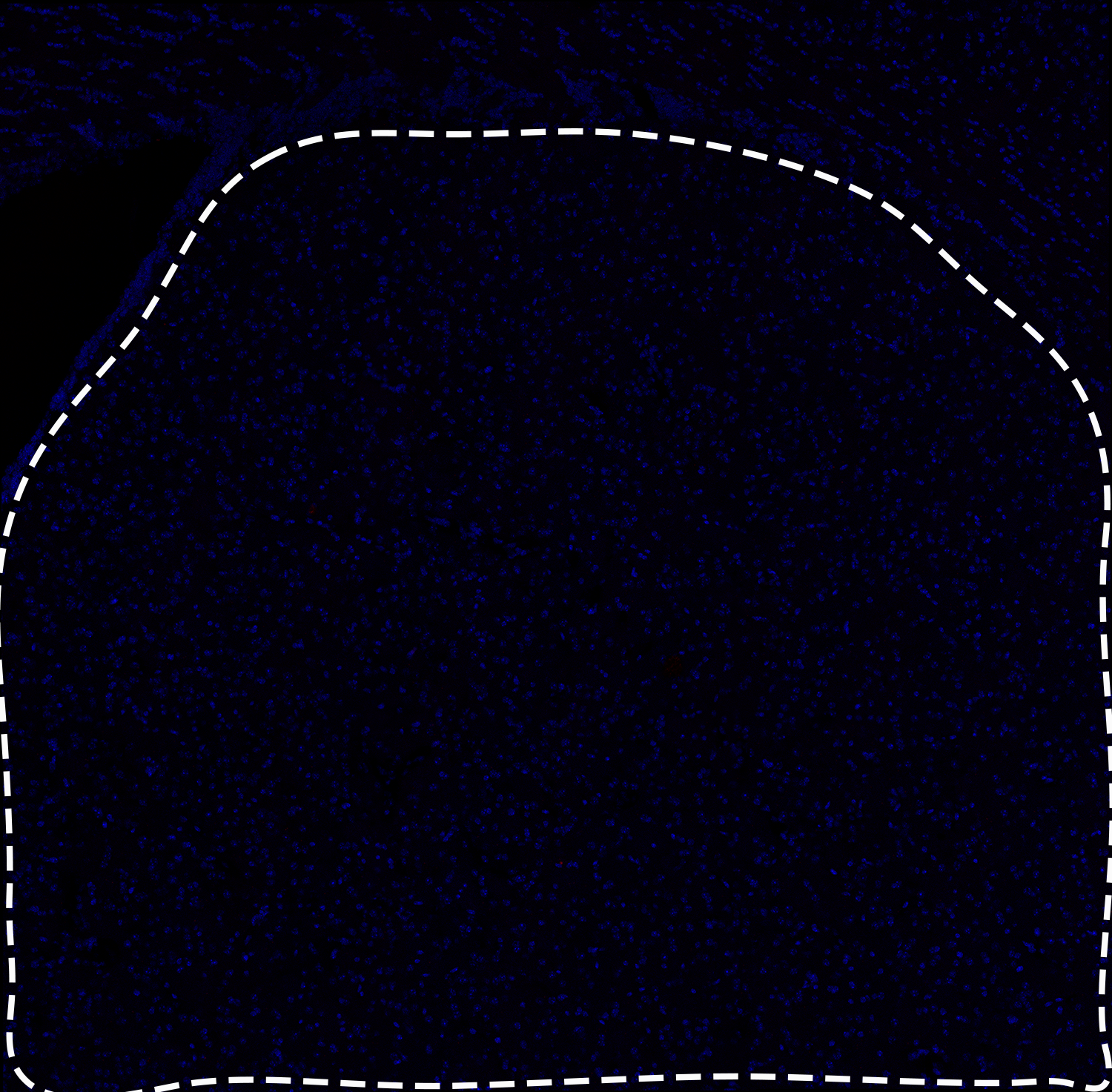

Supplement: Supplementary file 2 — EV Figures Source Data [file 44321_2024_54_MOESM2_ESM.zip › Raw_data_EV_figures/Figure EV1/Figure EV1A/CRBN-KO/CRBN-KO - STR 1.tif]

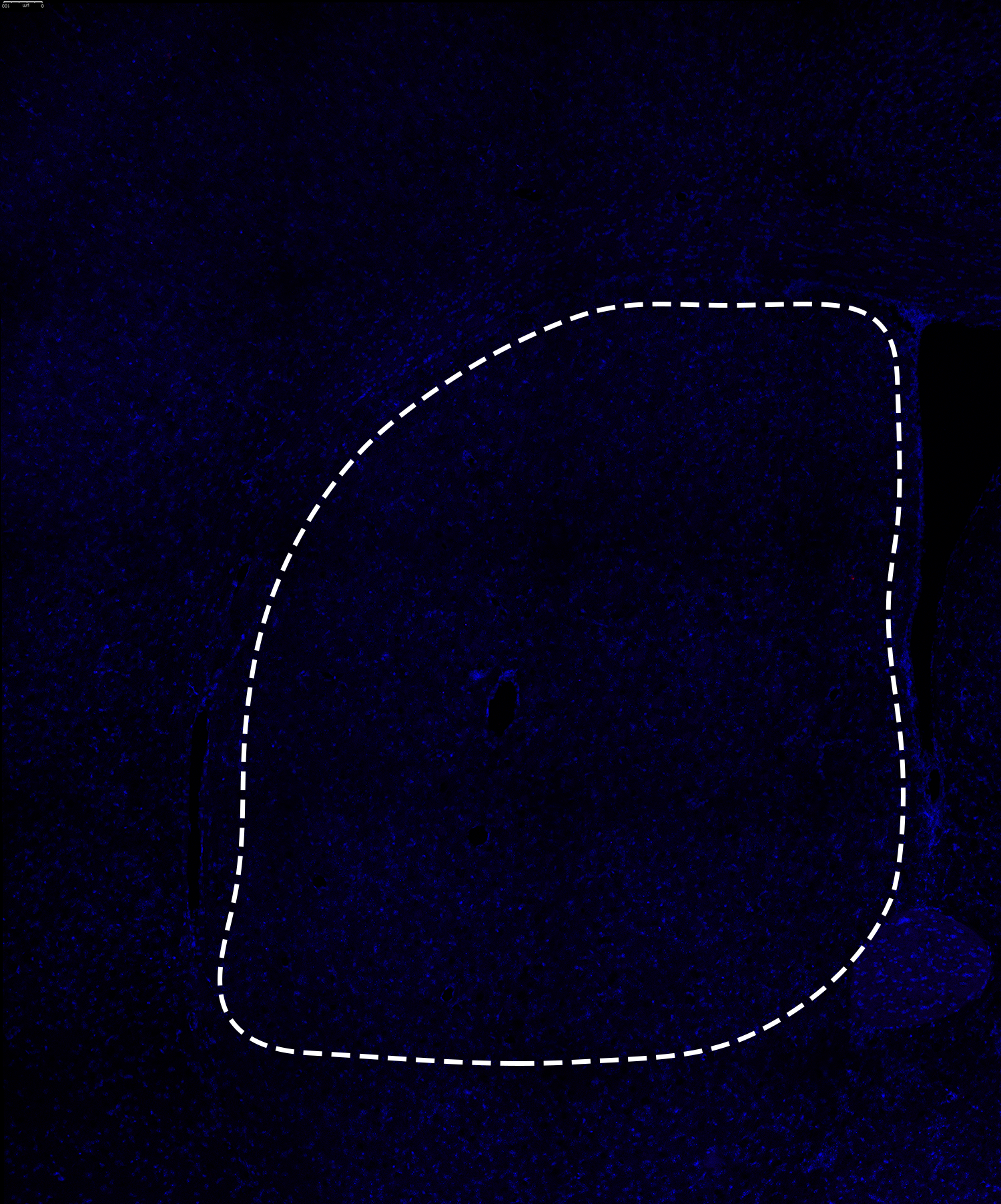

Supplement: Supplementary file 2 — EV Figures Source Data [file 44321_2024_54_MOESM2_ESM.zip › Raw_data_EV_figures/Figure EV1/Figure EV1A/CRBN-KO/CRBN-KO - STR 2.tif]

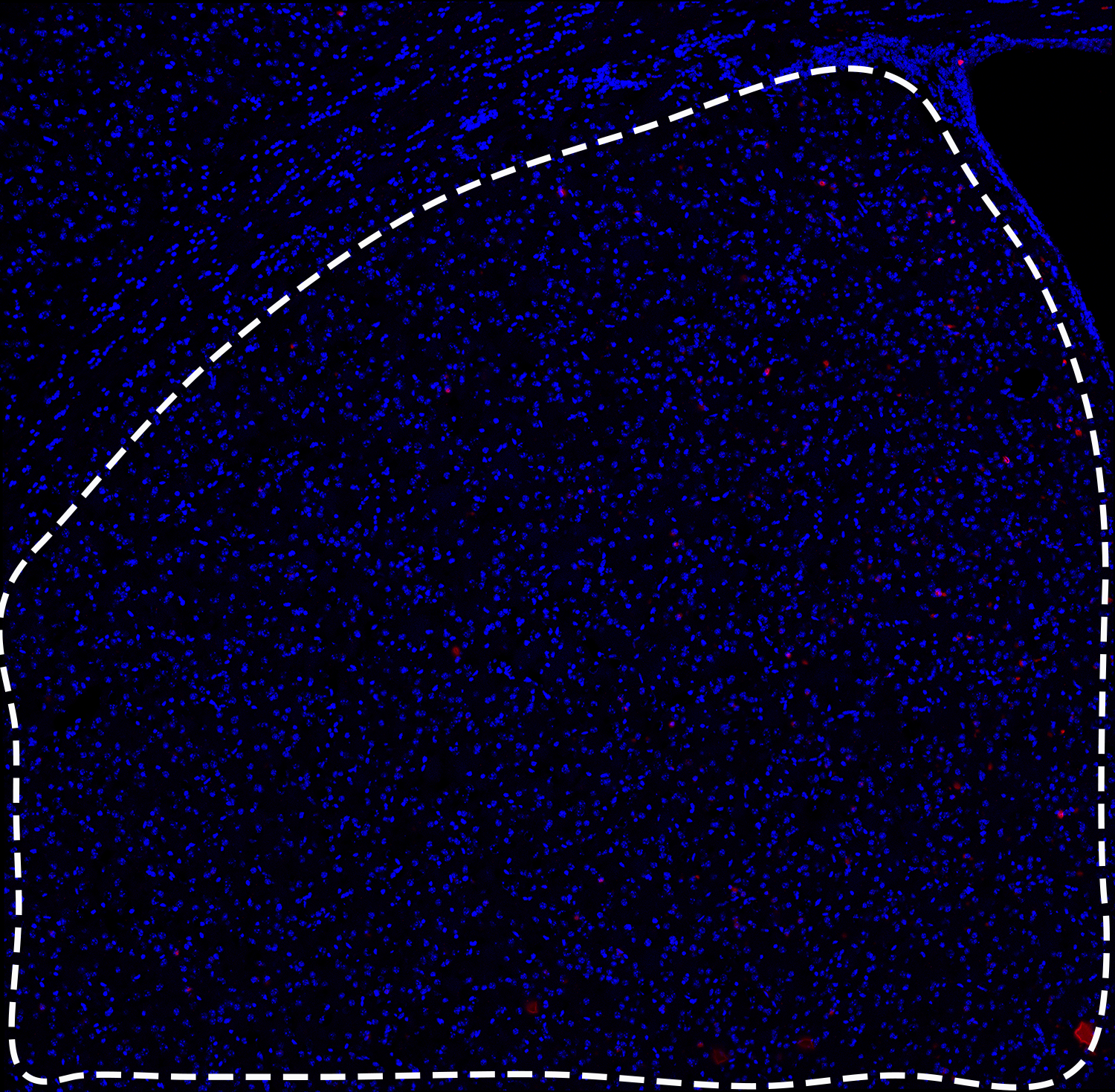

Supplement: Supplementary file 2 — EV Figures Source Data [file 44321_2024_54_MOESM2_ESM.zip › Raw_data_EV_figures/Figure EV1/Figure EV1A/CRBN-KO/CRBN-KO - STR 3.tif]

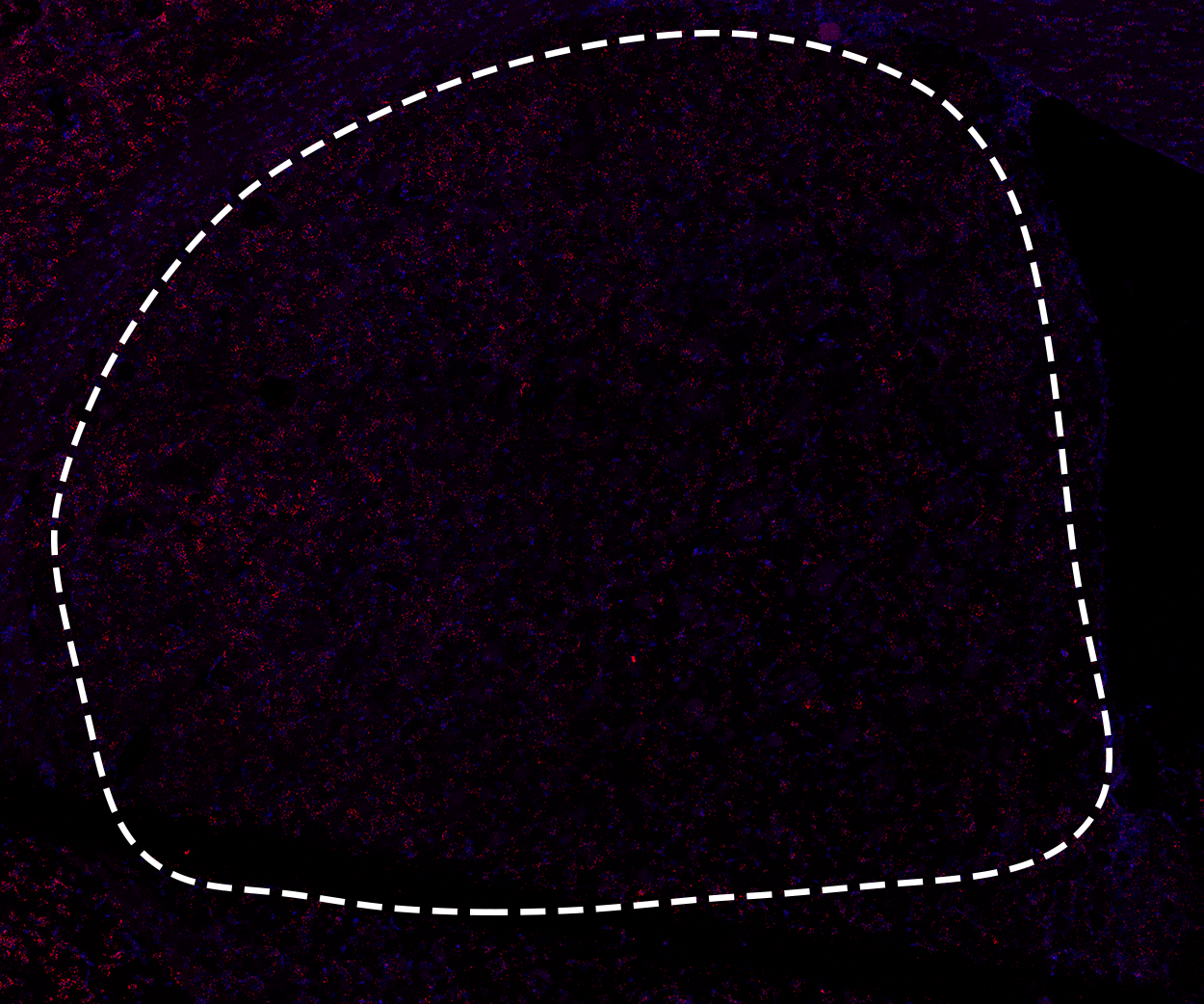

Supplement: Supplementary file 2 — EV Figures Source Data [file 44321_2024_54_MOESM2_ESM.zip › Raw_data_EV_figures/Figure EV1/Figure EV1A/CRBN-WT/CRBN-WT - STR 1.tif]

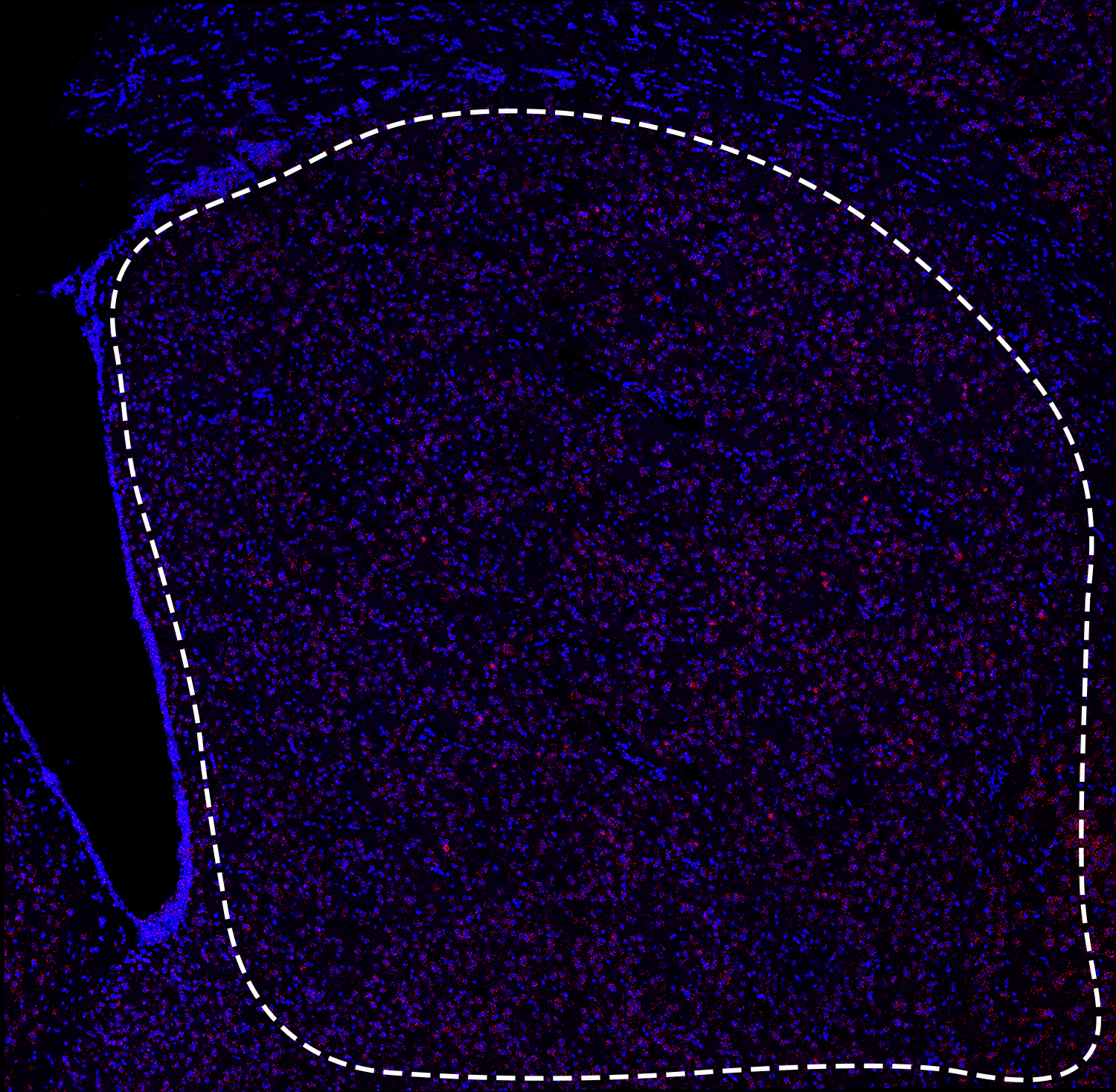

Supplement: Supplementary file 2 — EV Figures Source Data [file 44321_2024_54_MOESM2_ESM.zip › Raw_data_EV_figures/Figure EV1/Figure EV1A/CRBN-WT/CRBN-WT - STR 2.tif]

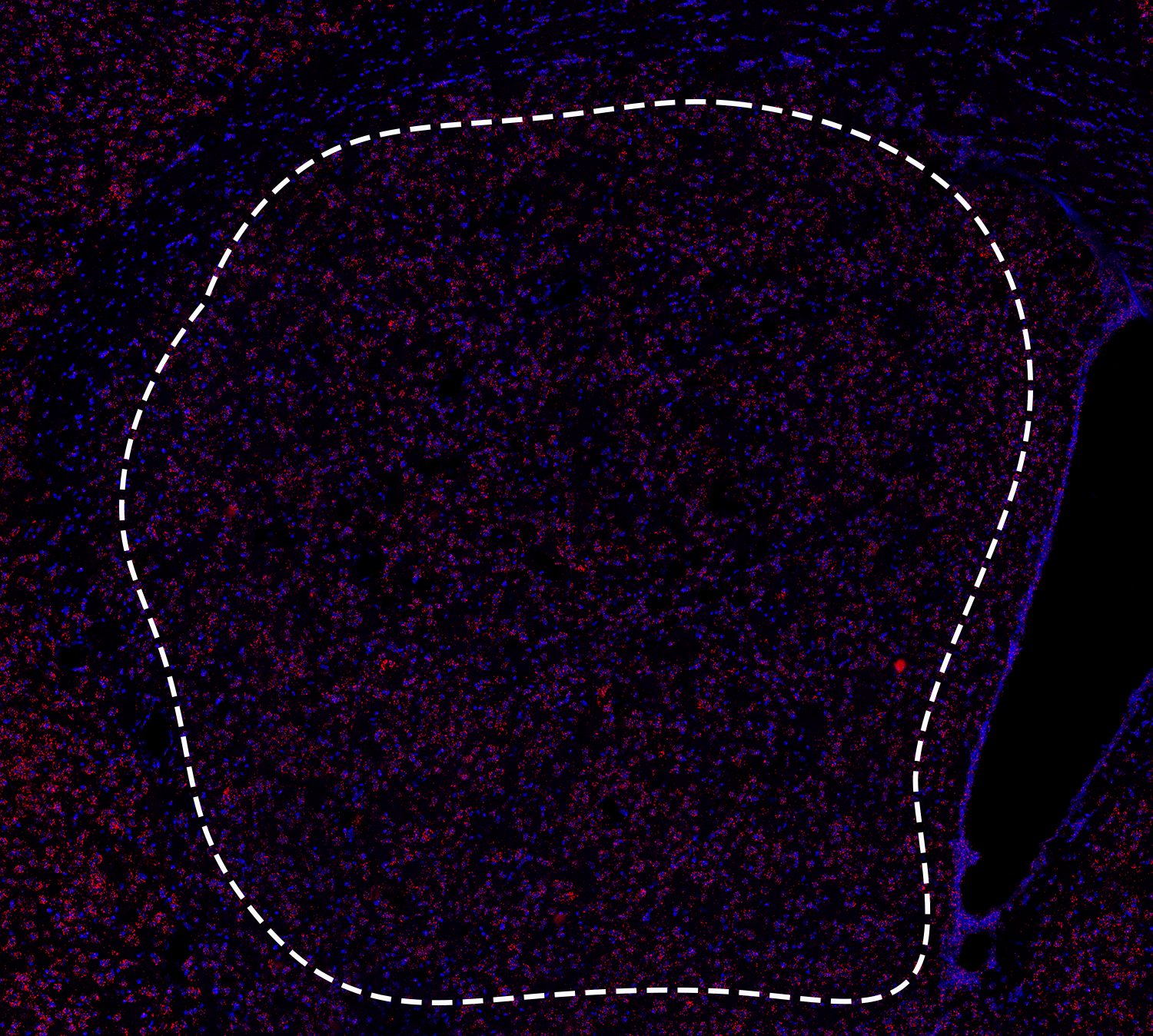

Supplement: Supplementary file 2 — EV Figures Source Data [file 44321_2024_54_MOESM2_ESM.zip › Raw_data_EV_figures/Figure EV1/Figure EV1A/CRBN-WT/CRBN-WT - STR 3.tif]

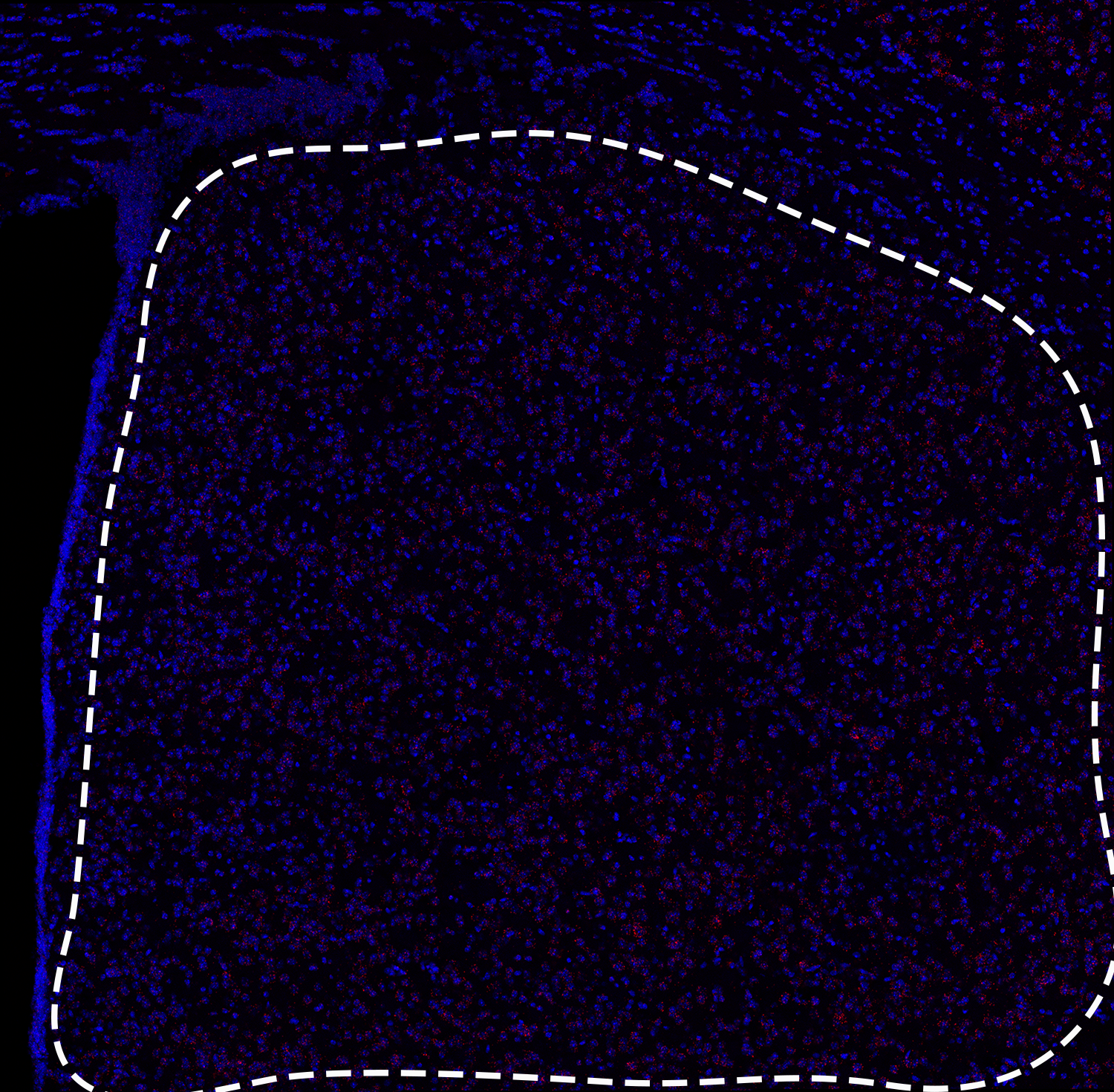

Supplement: Supplementary file 2 — EV Figures Source Data [file 44321_2024_54_MOESM2_ESM.zip › Raw_data_EV_figures/Figure EV1/Figure EV1A/CRBN-WT/CRBN-WT - STR 4.tif]

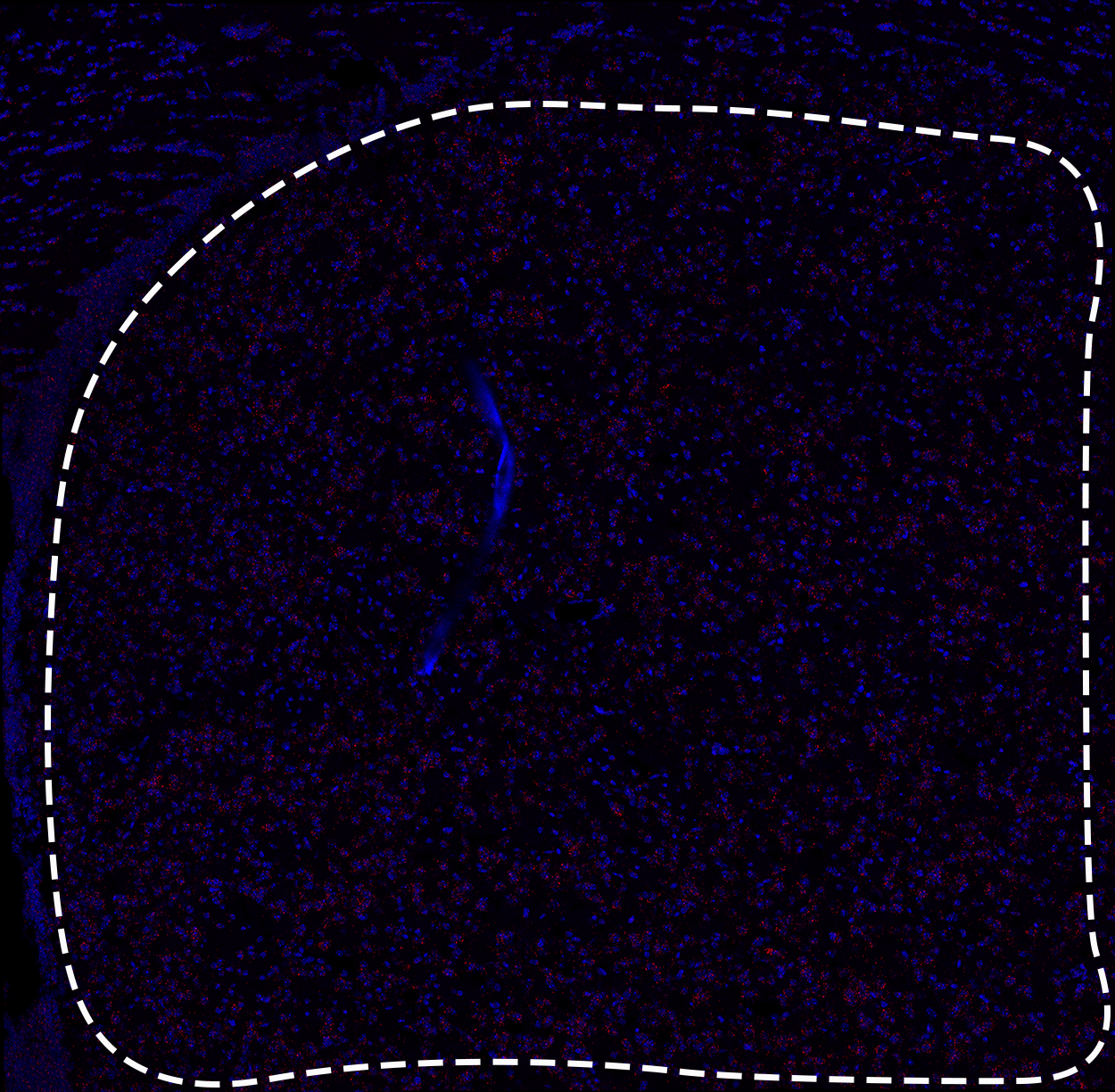

Supplement: Supplementary file 2 — EV Figures Source Data [file 44321_2024_54_MOESM2_ESM.zip › Raw_data_EV_figures/Figure EV1/Figure EV1A/CRBN-WT/CRBN-WT - STR 5.tif]

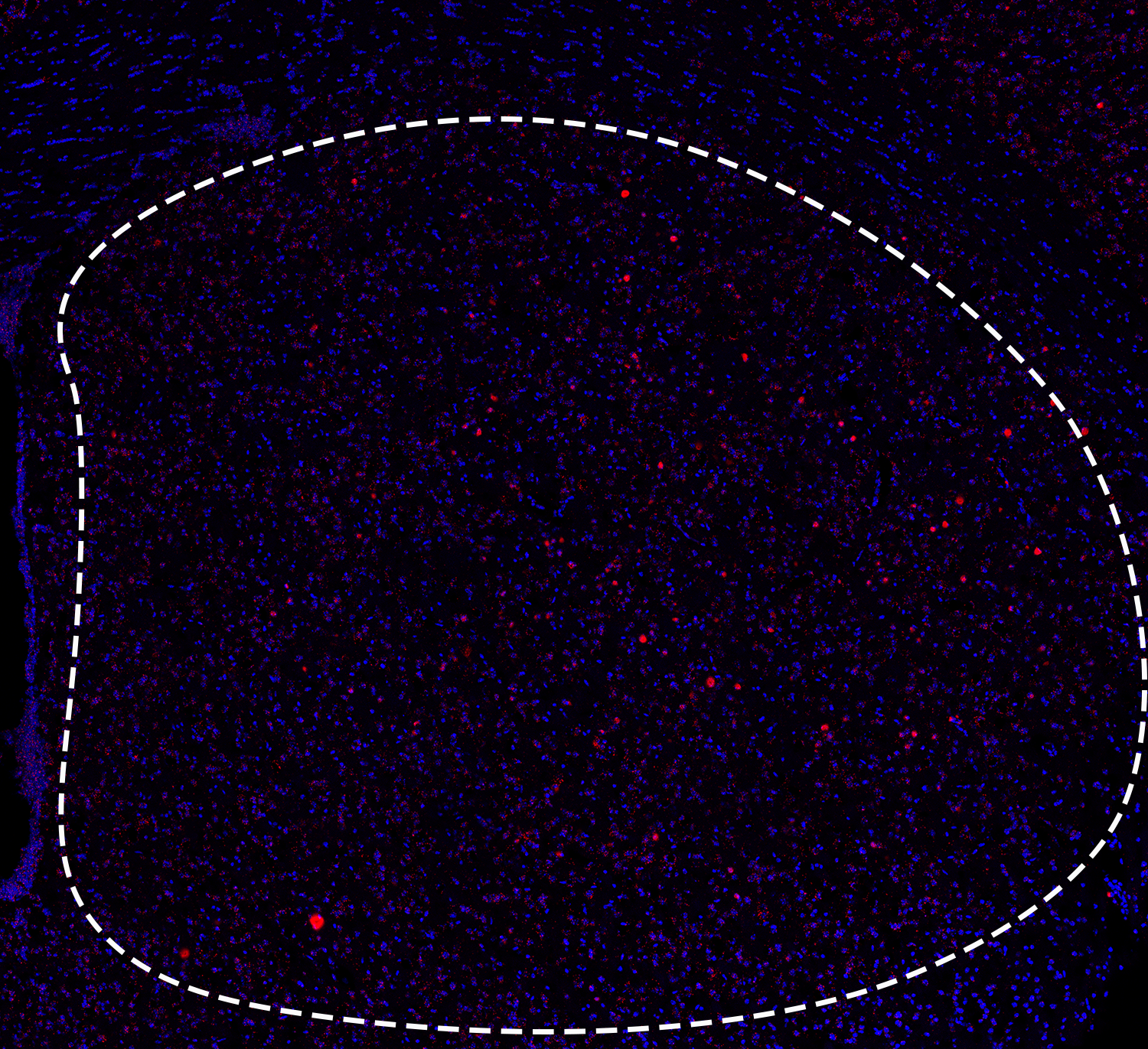

Supplement: Supplementary file 2 — EV Figures Source Data [file 44321_2024_54_MOESM2_ESM.zip › Raw_data_EV_figures/Figure EV1/Figure EV1A/CRBN-WT/CRBN-WT - STR 6.tif]

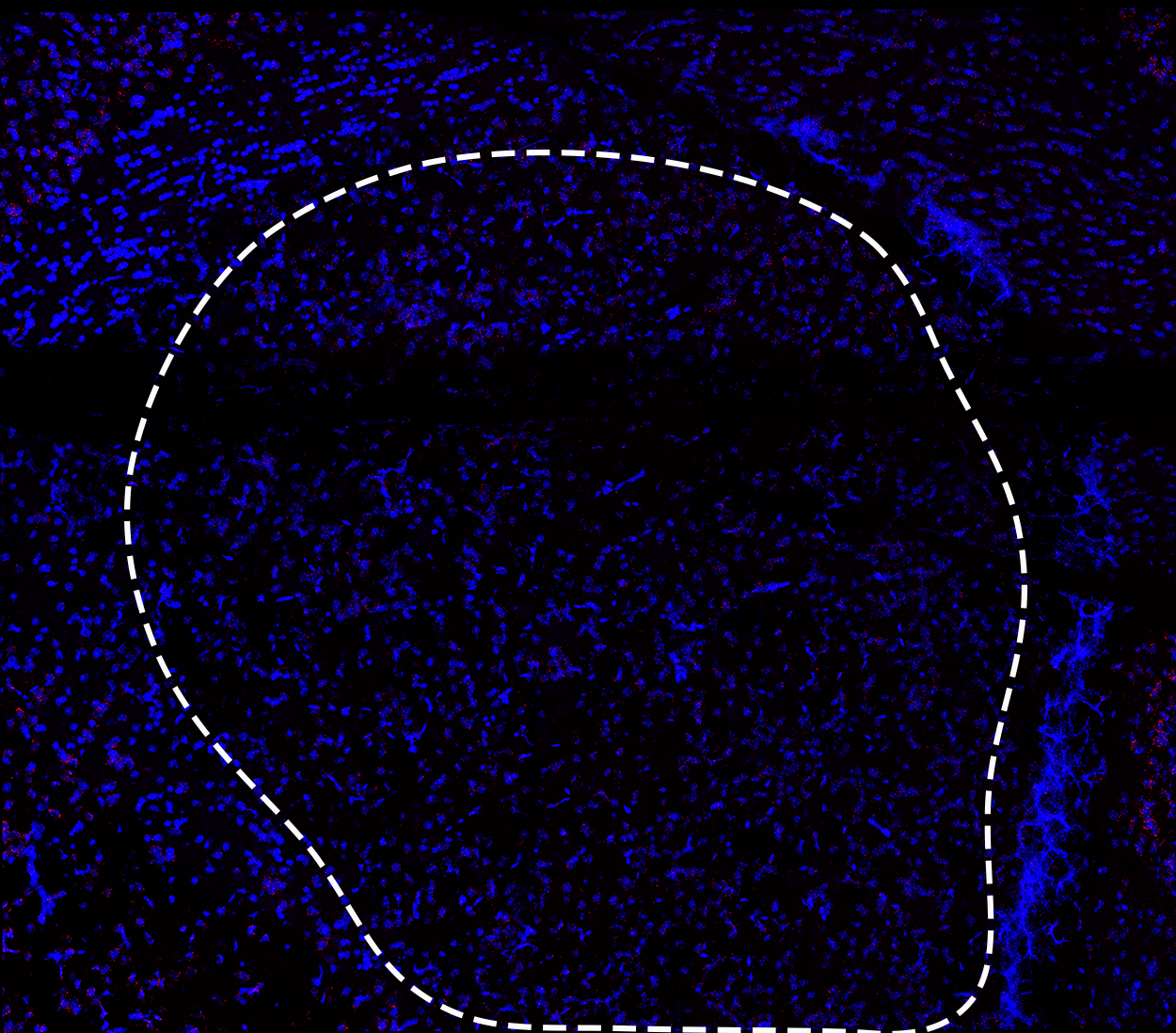

Supplement: Supplementary file 2 — EV Figures Source Data [file 44321_2024_54_MOESM2_ESM.zip › Raw_data_EV_figures/Figure EV1/Figure EV1A/GABA-CRBN-KO/GABA-CRBN-KO - STR 1.tif]

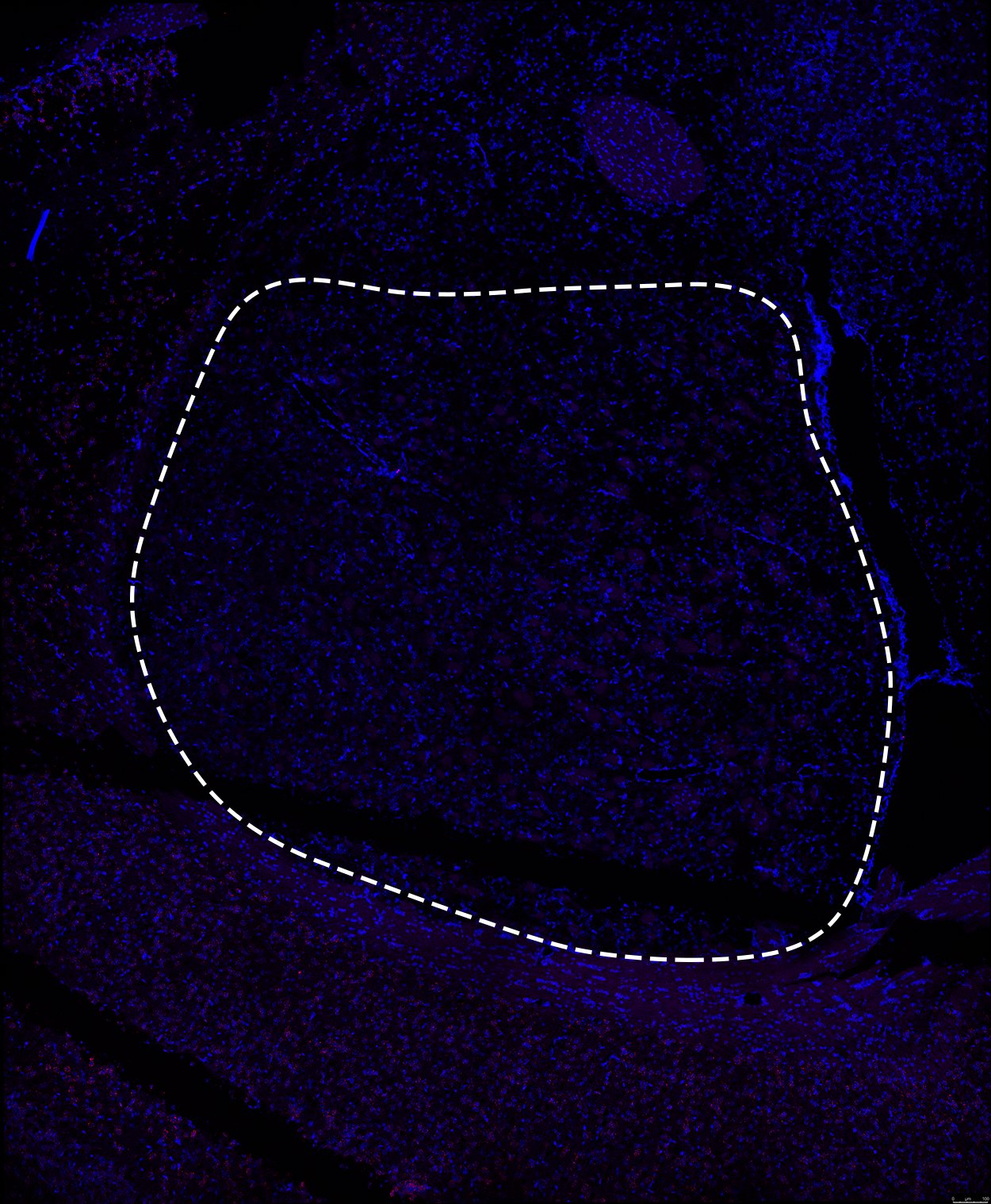

Supplement: Supplementary file 2 — EV Figures Source Data [file 44321_2024_54_MOESM2_ESM.zip › Raw_data_EV_figures/Figure EV1/Figure EV1A/GABA-CRBN-KO/GABA-CRBN-KO - STR 2.tif]

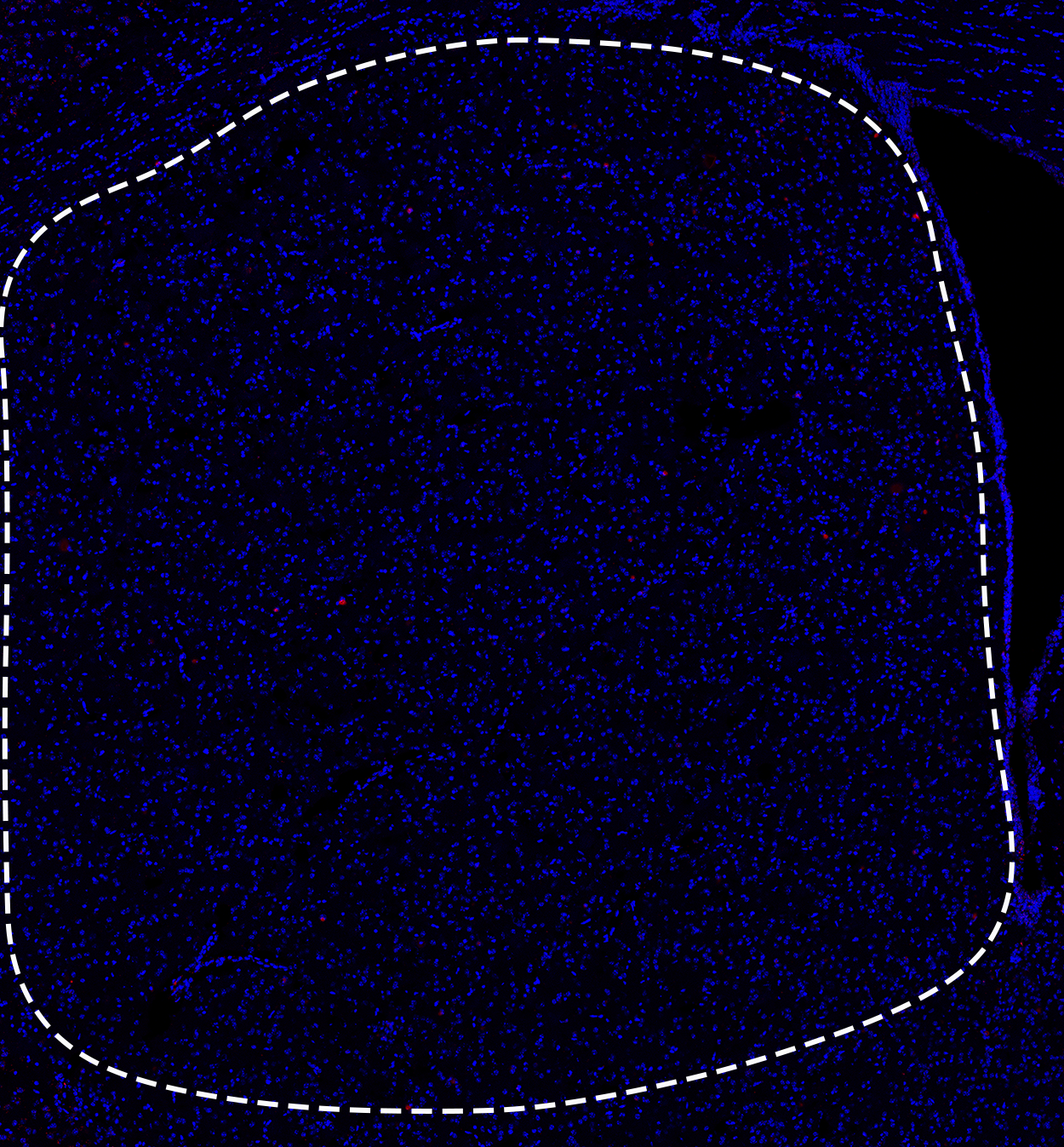

Supplement: Supplementary file 2 — EV Figures Source Data [file 44321_2024_54_MOESM2_ESM.zip › Raw_data_EV_figures/Figure EV1/Figure EV1A/GABA-CRBN-KO/GABA-CRBN-KO - STR 3.tif]

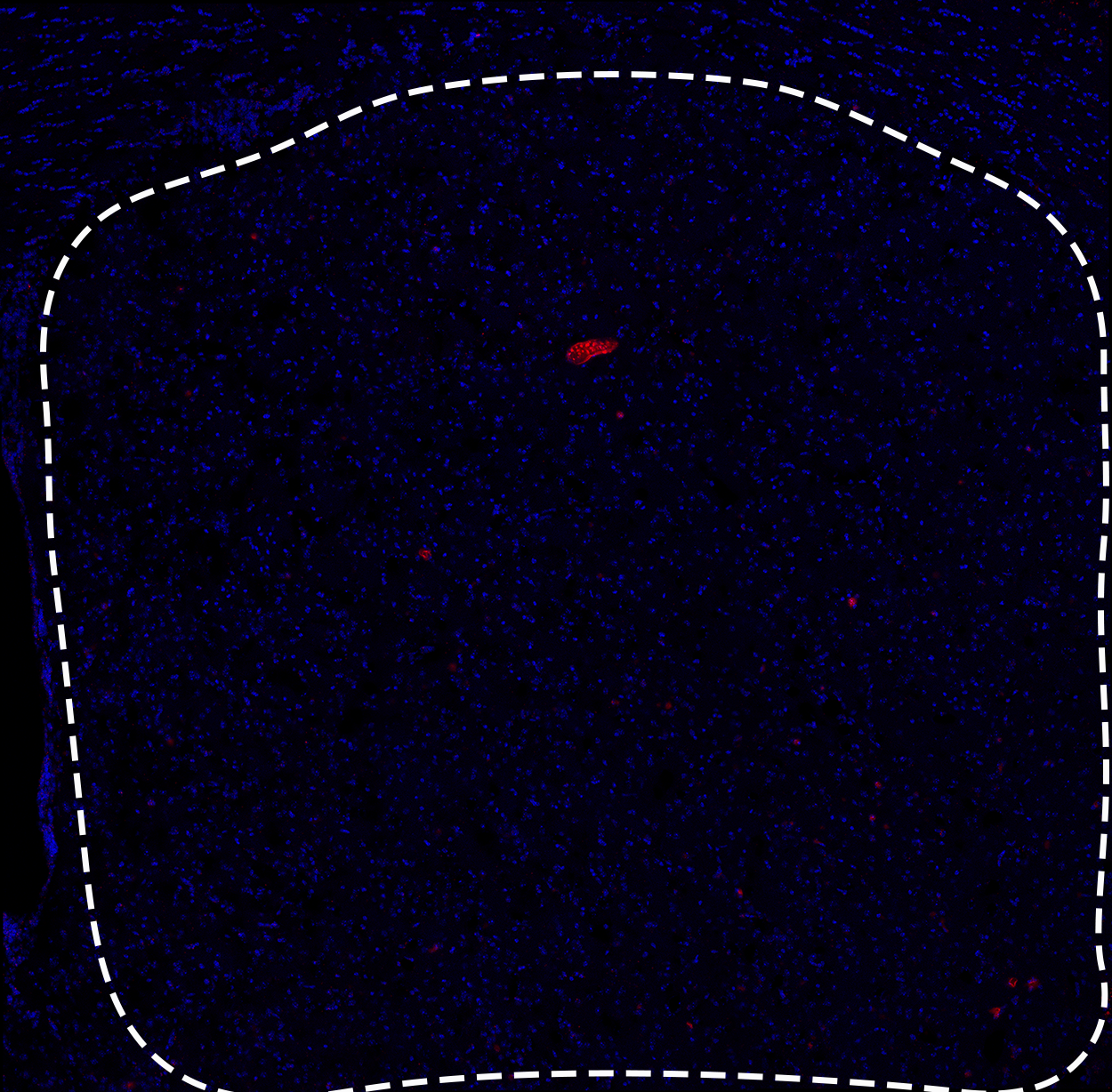

Supplement: Supplementary file 2 — EV Figures Source Data [file 44321_2024_54_MOESM2_ESM.zip › Raw_data_EV_figures/Figure EV1/Figure EV1A/GABA-CRBN-KO/GABA-CRBN-KO - STR 4.tif]

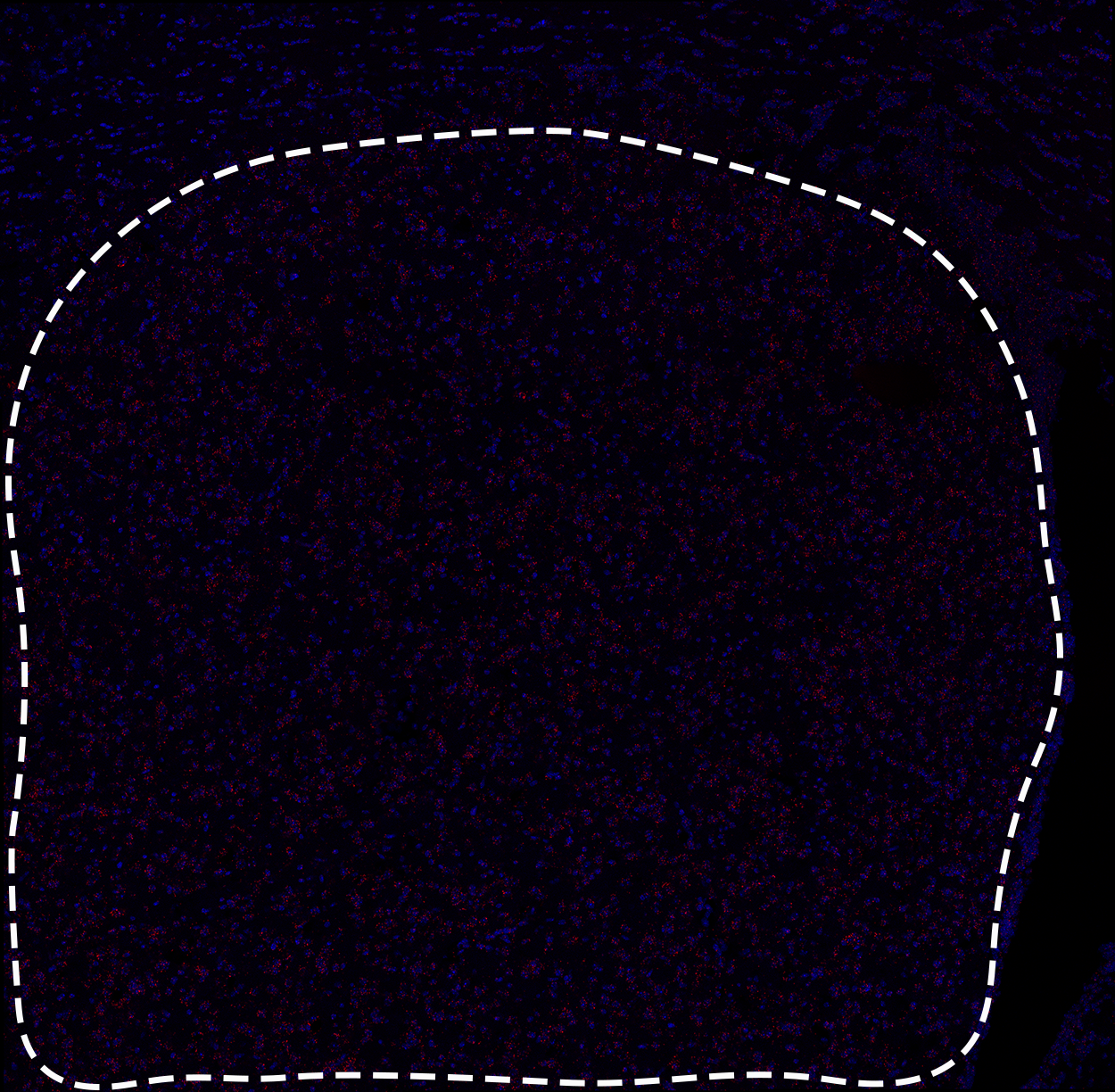

Supplement: Supplementary file 2 — EV Figures Source Data [file 44321_2024_54_MOESM2_ESM.zip › Raw_data_EV_figures/Figure EV1/Figure EV1A/Glu-CRBN-KO/Glu-CRBN-KO - STR 1.tif]

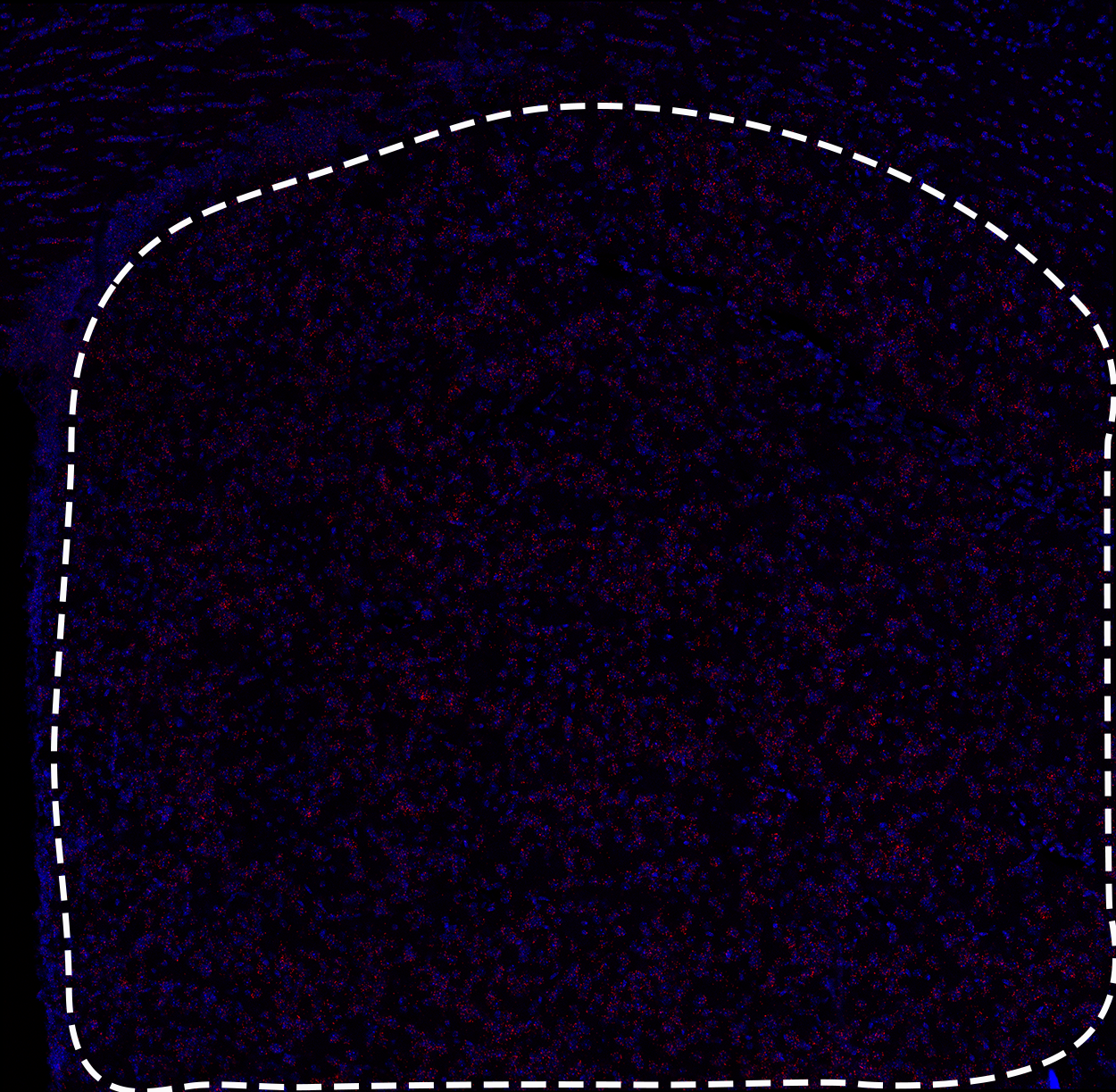

Supplement: Supplementary file 2 — EV Figures Source Data [file 44321_2024_54_MOESM2_ESM.zip › Raw_data_EV_figures/Figure EV1/Figure EV1A/Glu-CRBN-KO/Glu-CRBN-KO - STR 2.tif]

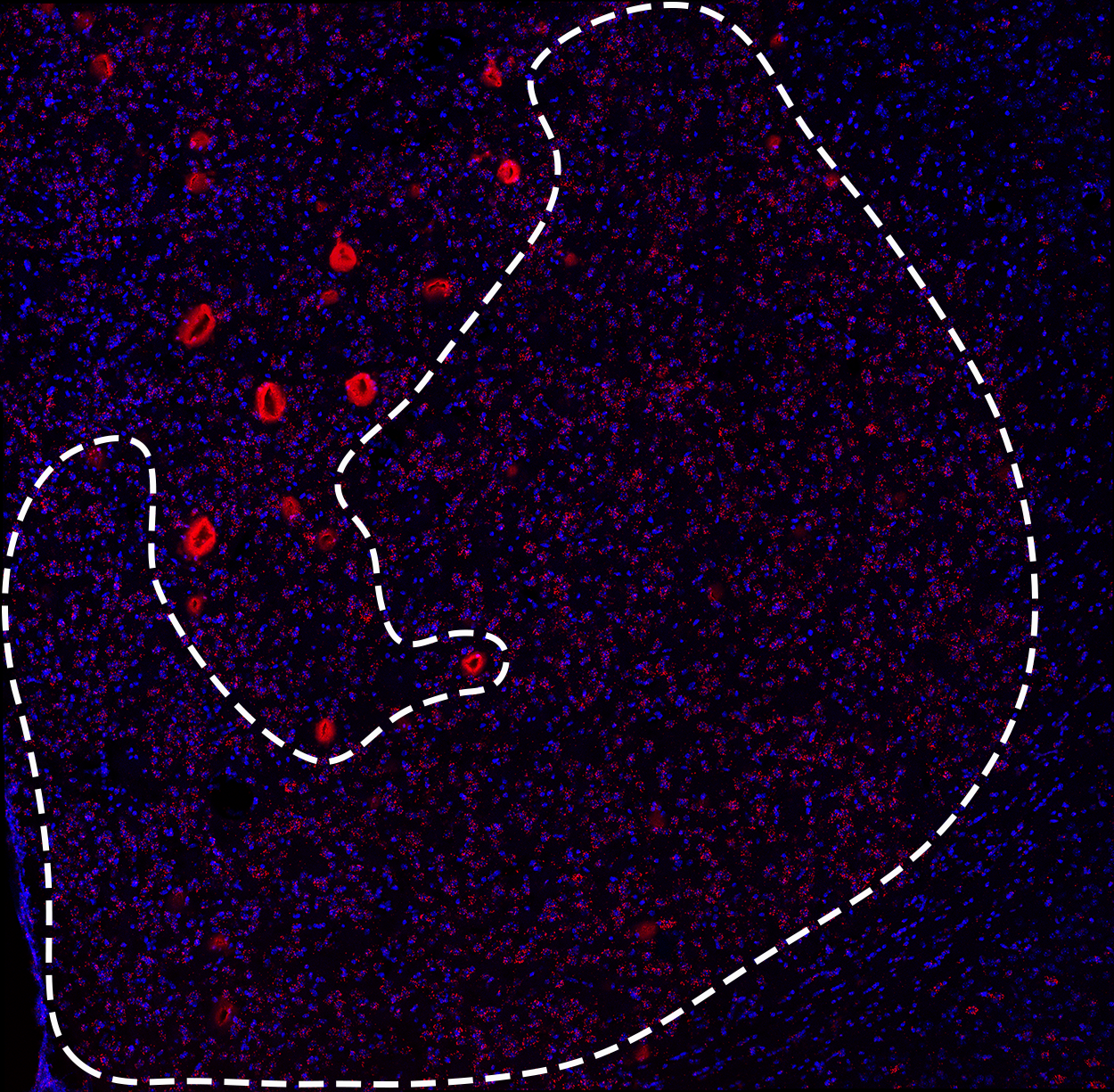

Supplement: Supplementary file 2 — EV Figures Source Data [file 44321_2024_54_MOESM2_ESM.zip › Raw_data_EV_figures/Figure EV1/Figure EV1A/Glu-CRBN-KO/Glu-CRBN-KO - STR 3.tif]

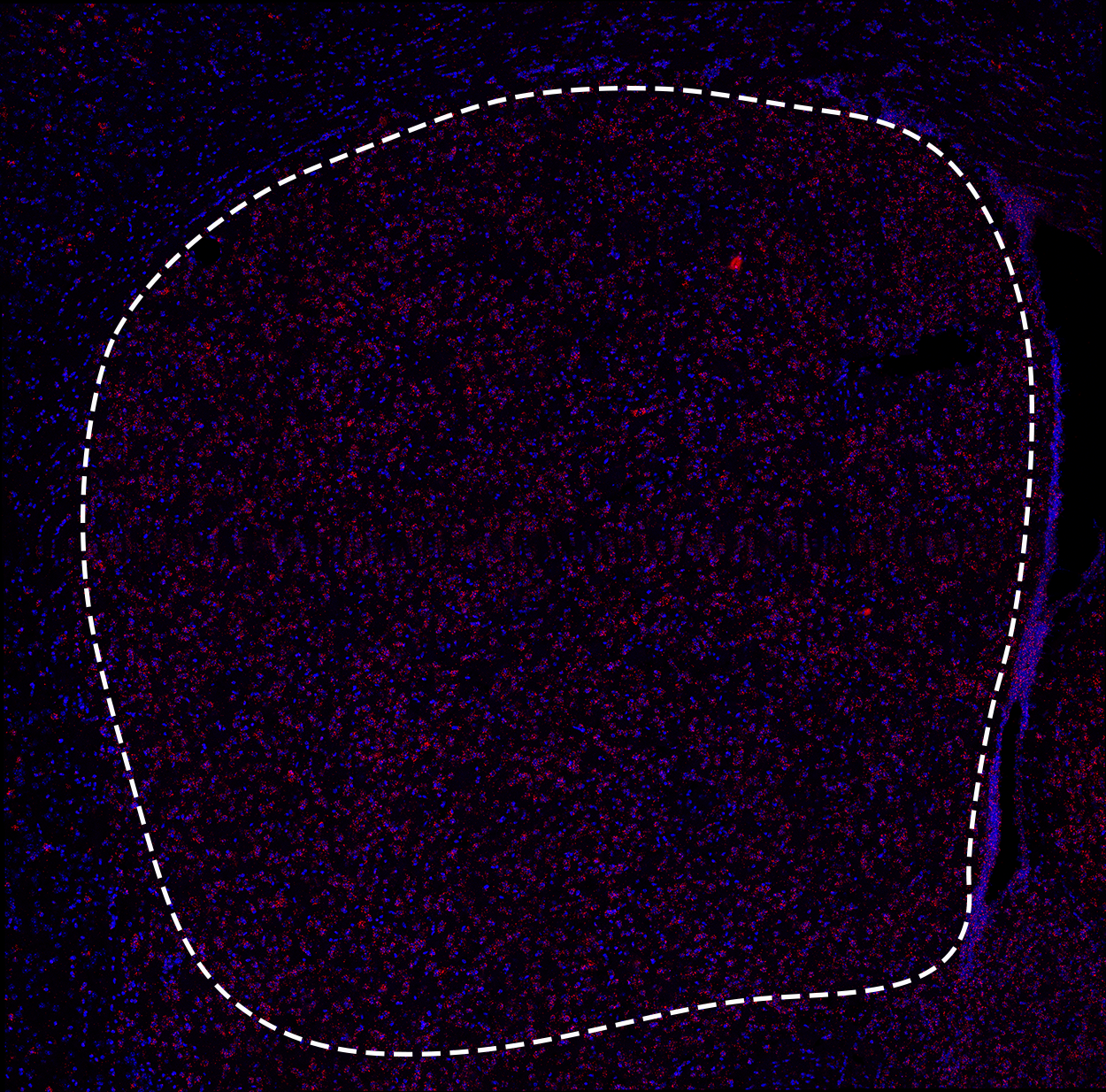

Supplement: Supplementary file 2 — EV Figures Source Data [file 44321_2024_54_MOESM2_ESM.zip › Raw_data_EV_figures/Figure EV1/Figure EV1A/Glu-CRBN-KO/Glu-CRBN-KO - STR 4.tif]

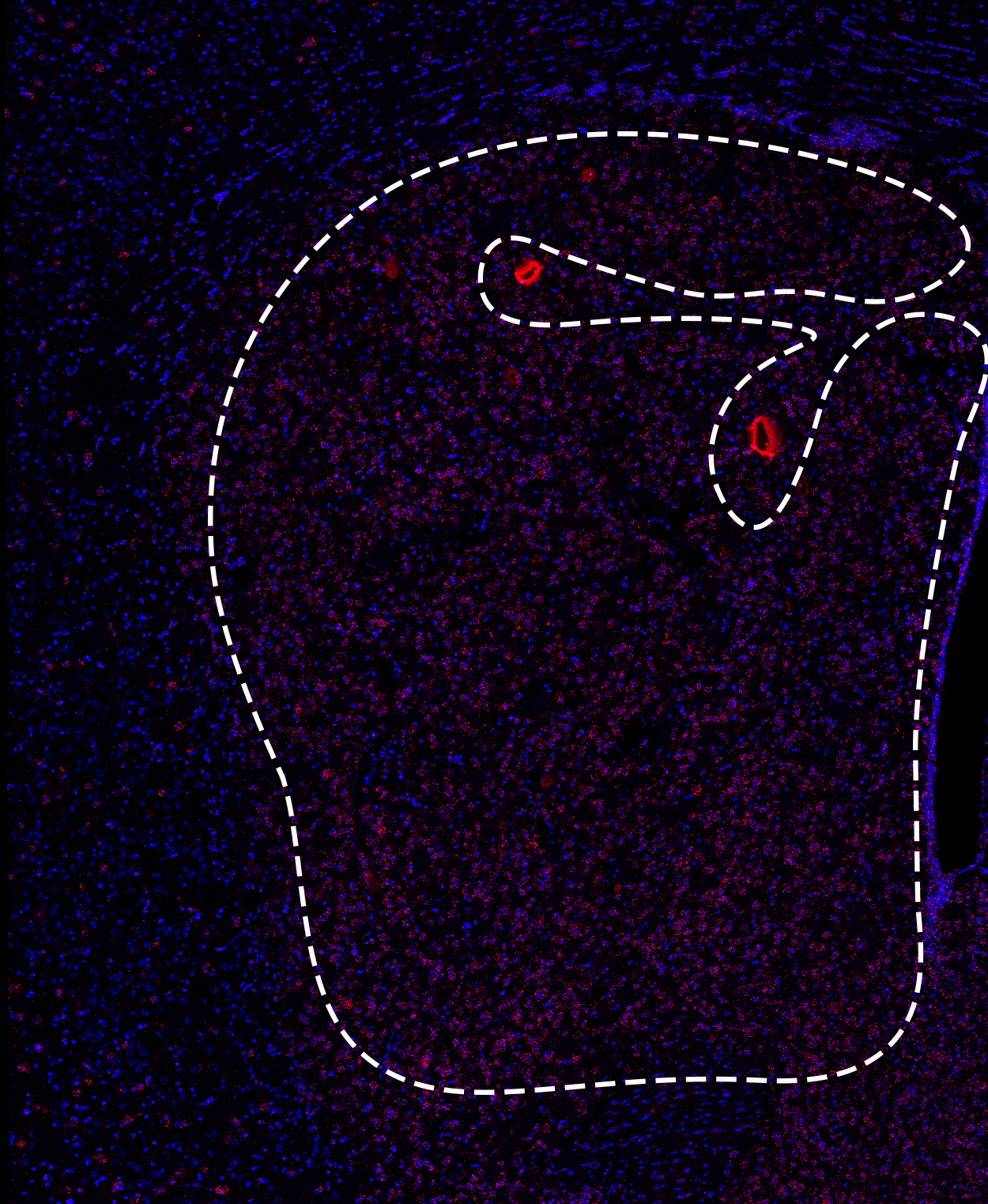

Supplement: Supplementary file 2 — EV Figures Source Data [file 44321_2024_54_MOESM2_ESM.zip › Raw_data_EV_figures/Figure EV1/Figure EV1A/Glu-CRBN-KO/Glu-CRBN-KO - STR 5.tif]

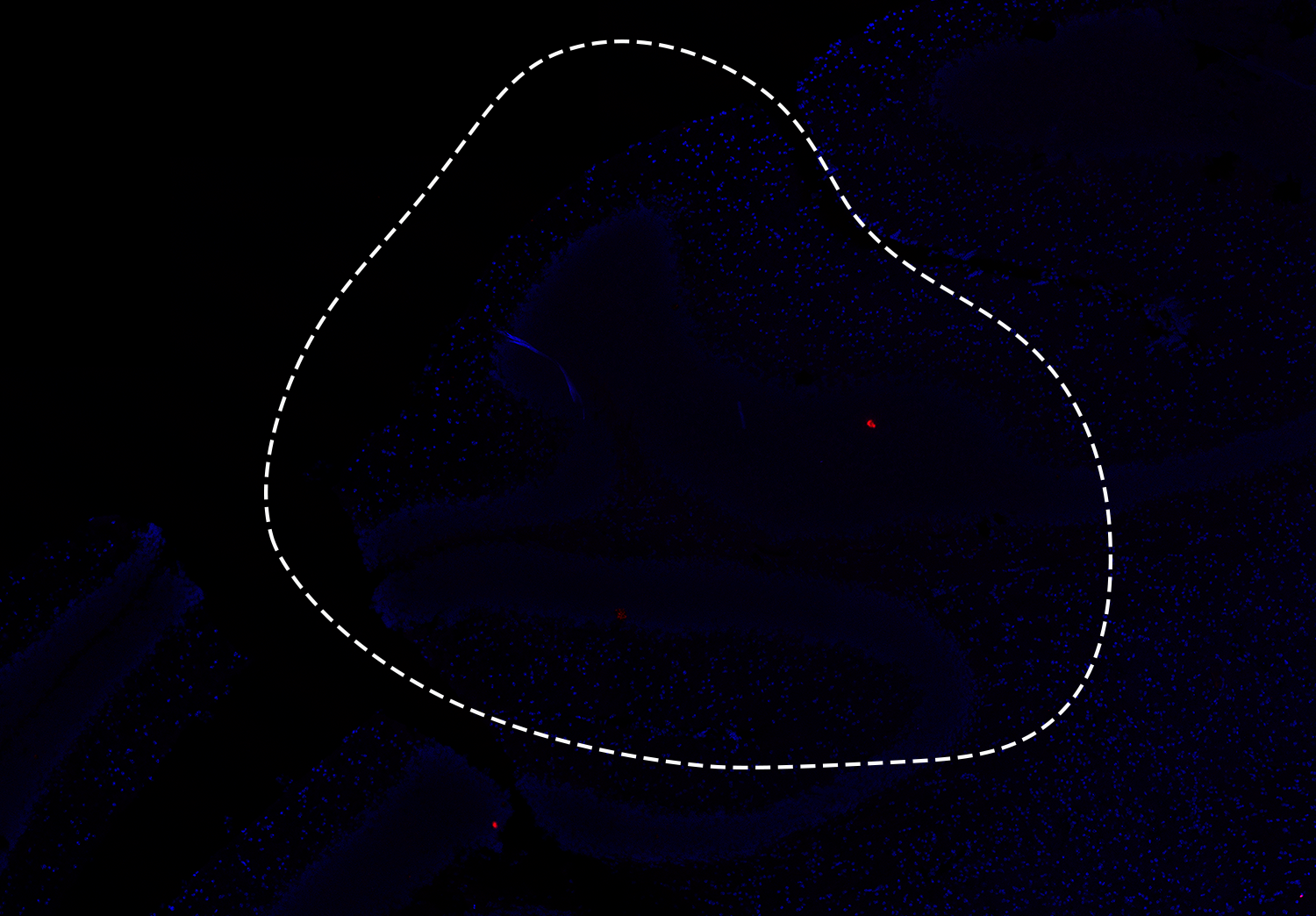

Supplement: Supplementary file 2 — EV Figures Source Data [file 44321_2024_54_MOESM2_ESM.zip › Raw_data_EV_figures/Figure EV1/Figure EV1B/CRBN-KO/CRBN-KO - CB 1.tif]

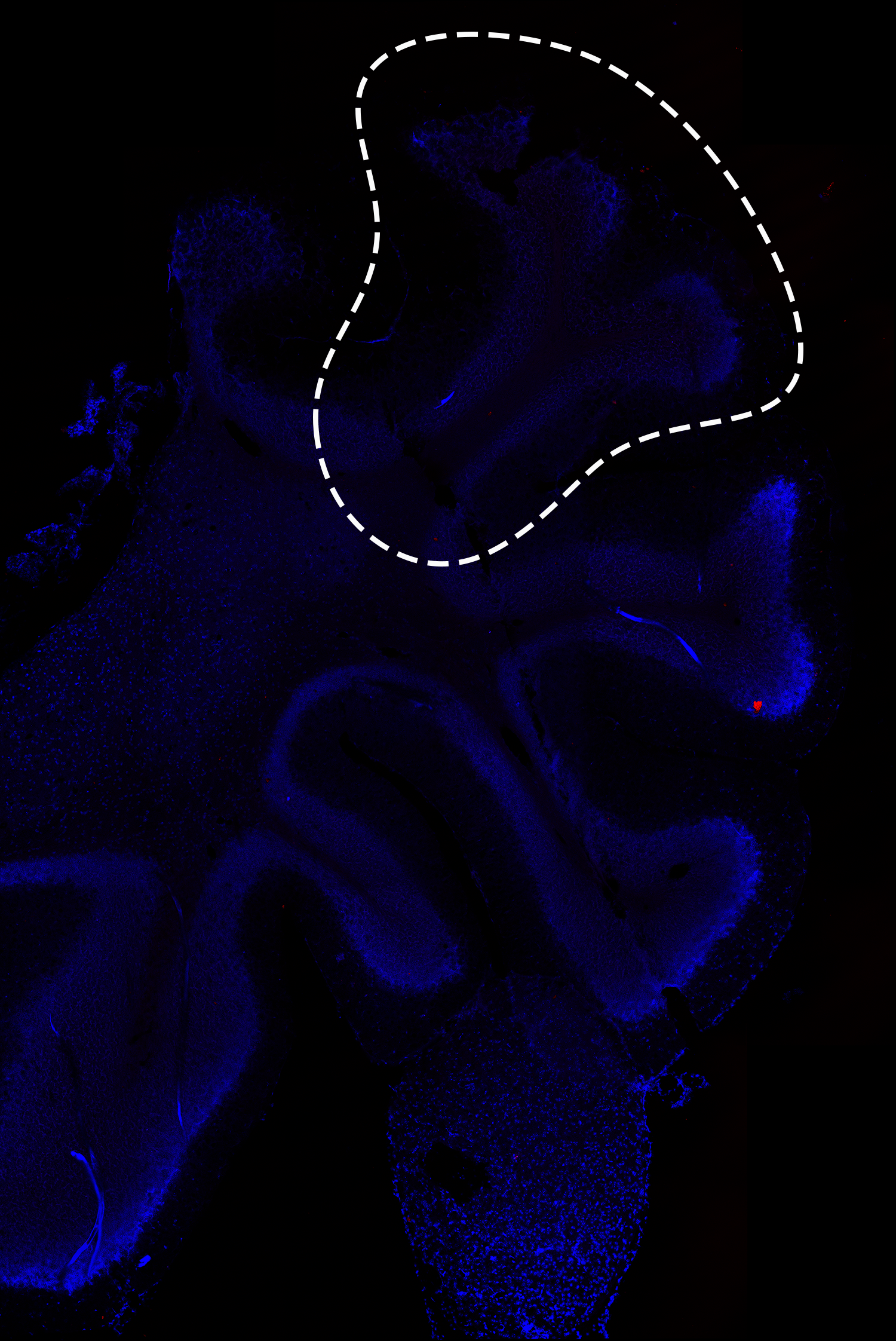

Supplement: Supplementary file 2 — EV Figures Source Data [file 44321_2024_54_MOESM2_ESM.zip › Raw_data_EV_figures/Figure EV1/Figure EV1B/CRBN-KO/CRBN-KO - CB 2.tif]

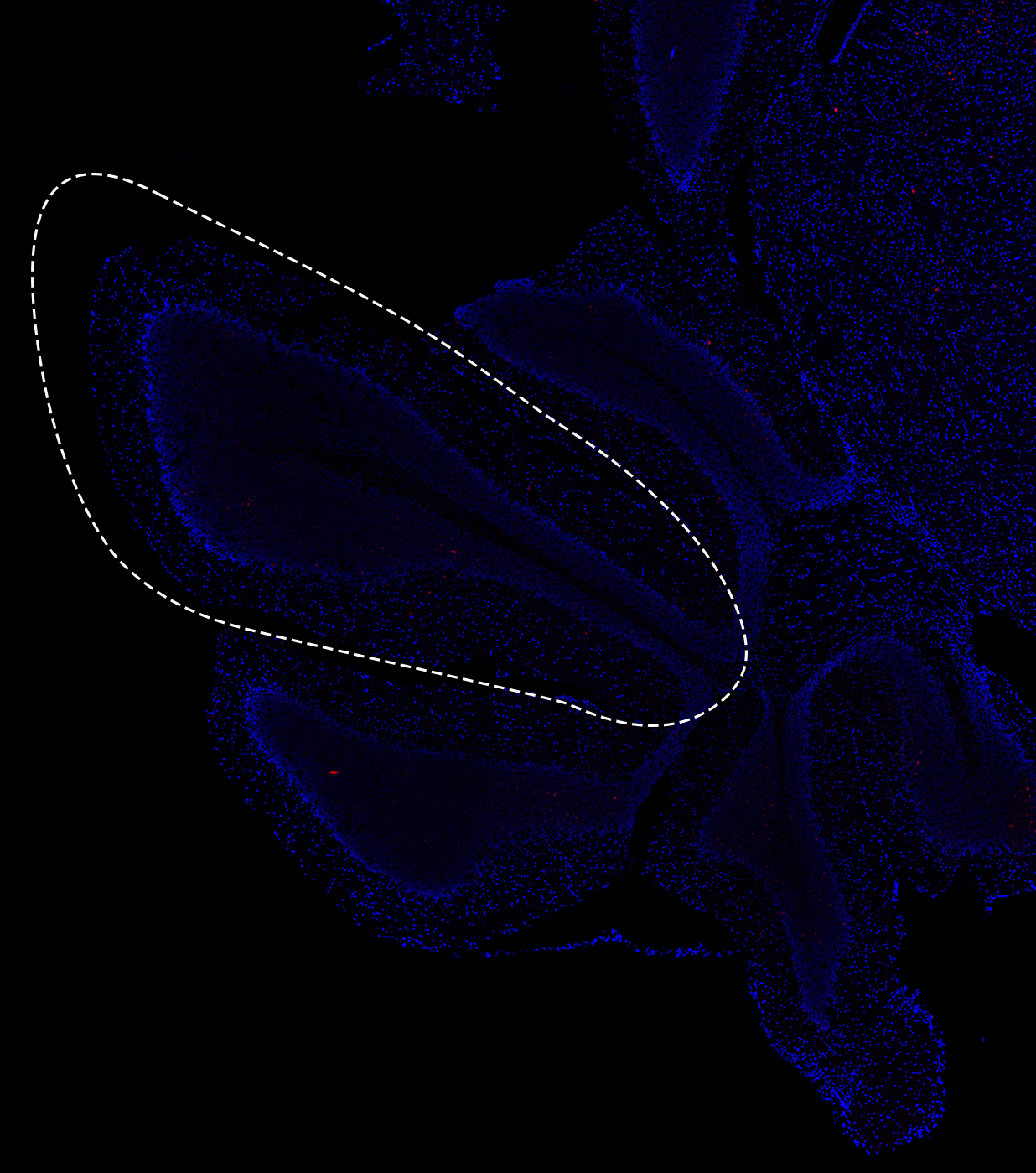

Supplement: Supplementary file 2 — EV Figures Source Data [file 44321_2024_54_MOESM2_ESM.zip › Raw_data_EV_figures/Figure EV1/Figure EV1B/CRBN-KO/CRBN-KO - CB 3.tif]

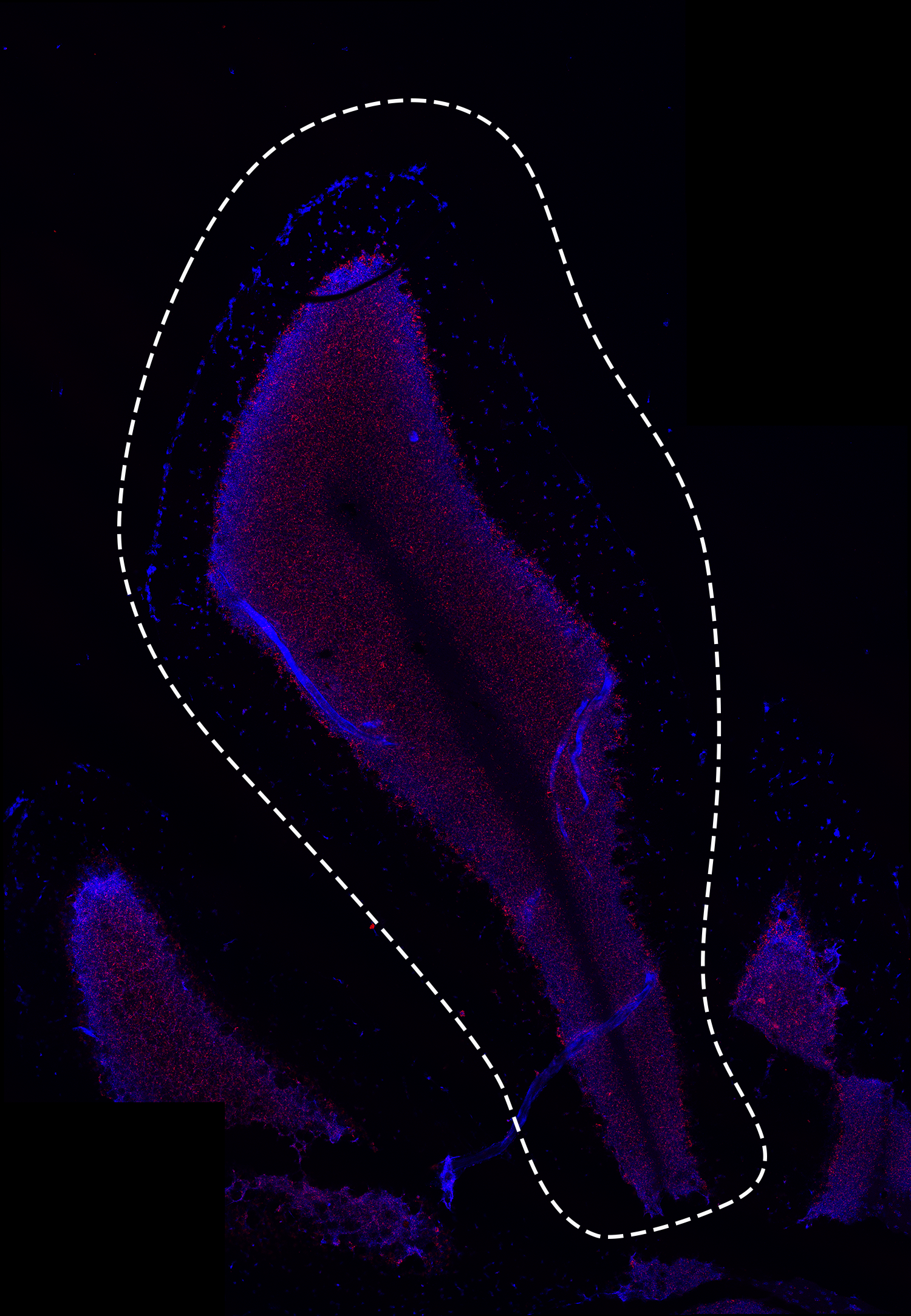

Supplement: Supplementary file 2 — EV Figures Source Data [file 44321_2024_54_MOESM2_ESM.zip › Raw_data_EV_figures/Figure EV1/Figure EV1B/CRBN-WT/CRBN WT - CB 1.tif]

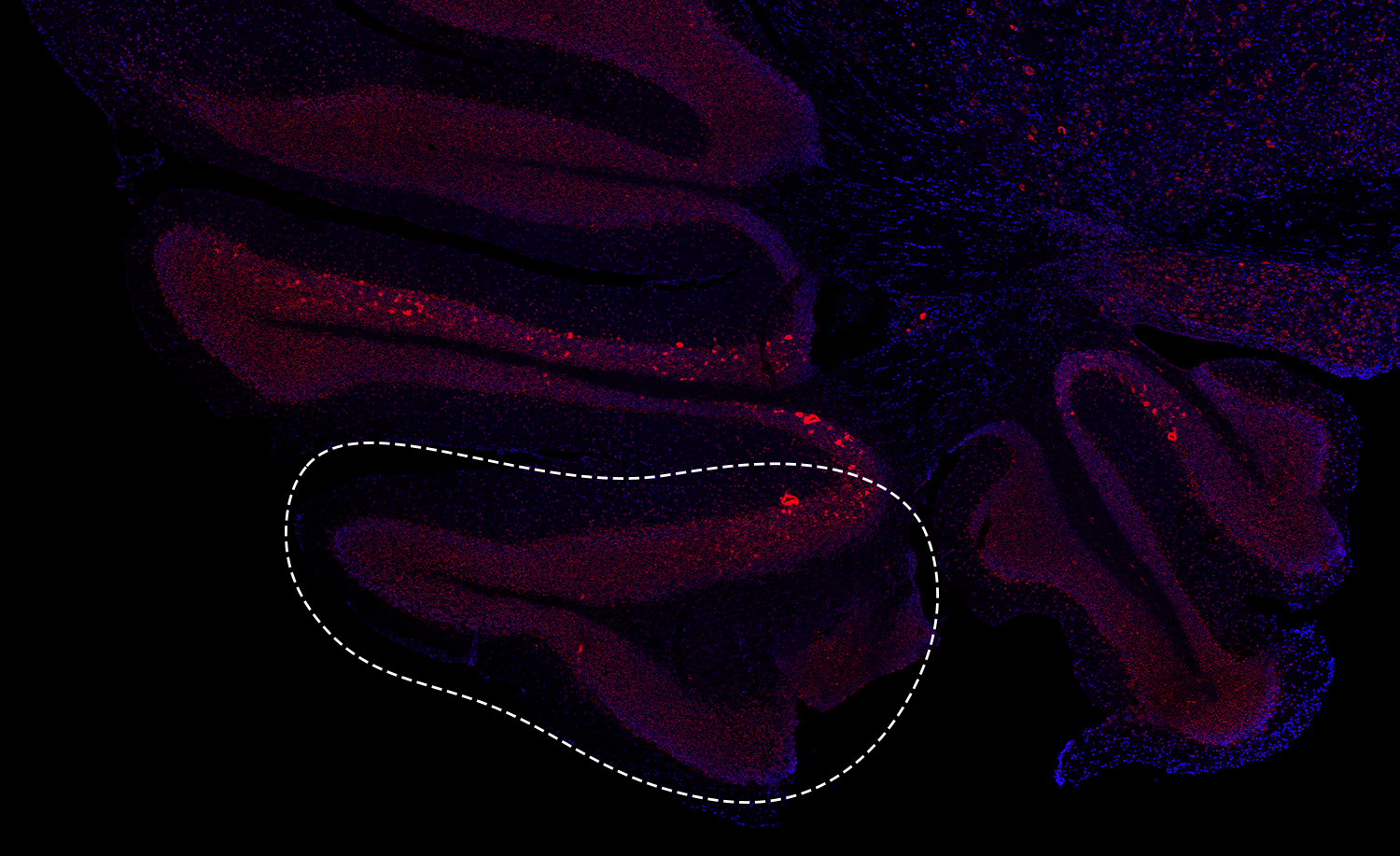

Supplement: Supplementary file 2 — EV Figures Source Data [file 44321_2024_54_MOESM2_ESM.zip › Raw_data_EV_figures/Figure EV1/Figure EV1B/CRBN-WT/CRBN WT - CB 2.tif]

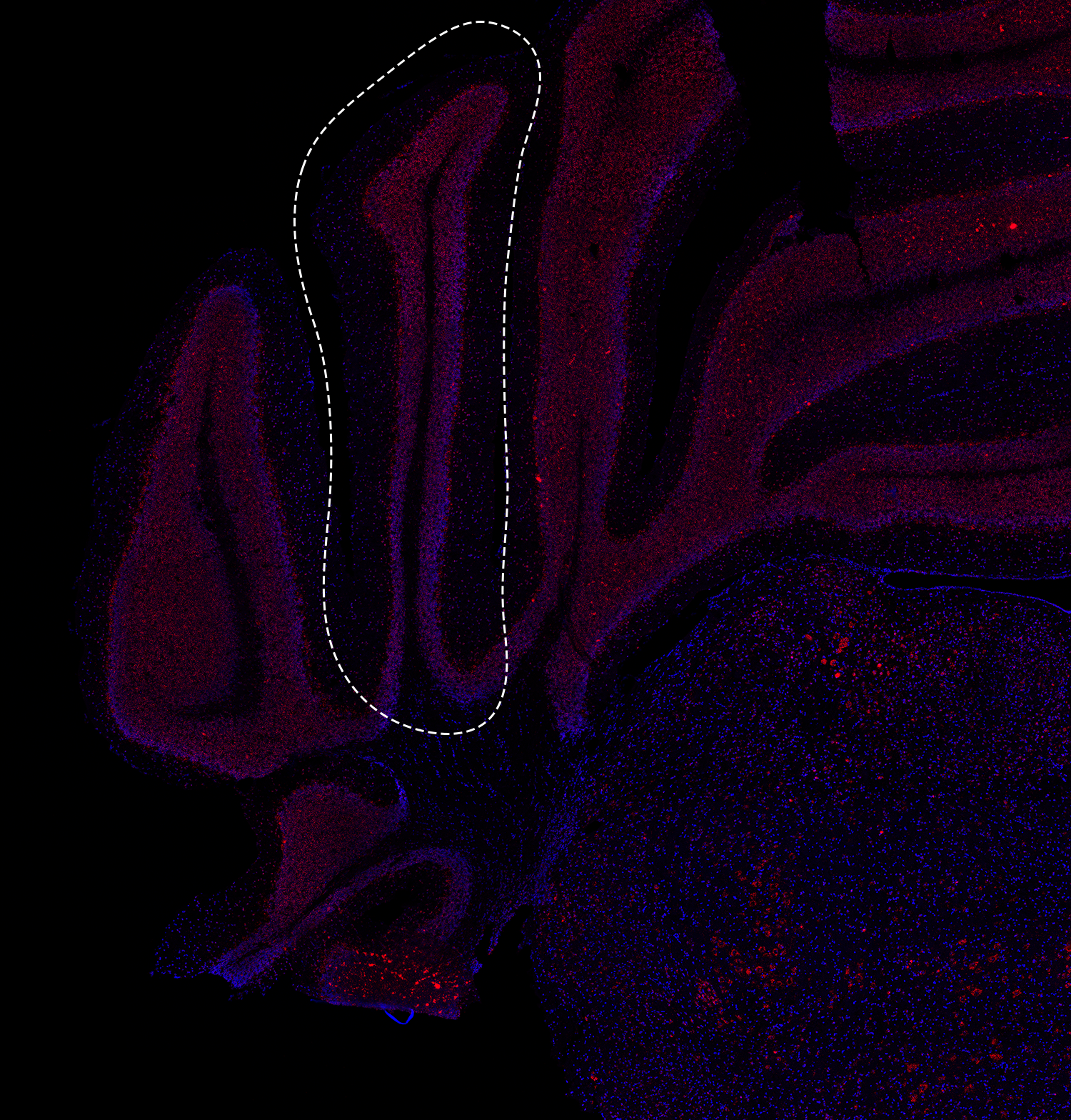

Supplement: Supplementary file 2 — EV Figures Source Data [file 44321_2024_54_MOESM2_ESM.zip › Raw_data_EV_figures/Figure EV1/Figure EV1B/CRBN-WT/CRBN WT - CB 3.tif]

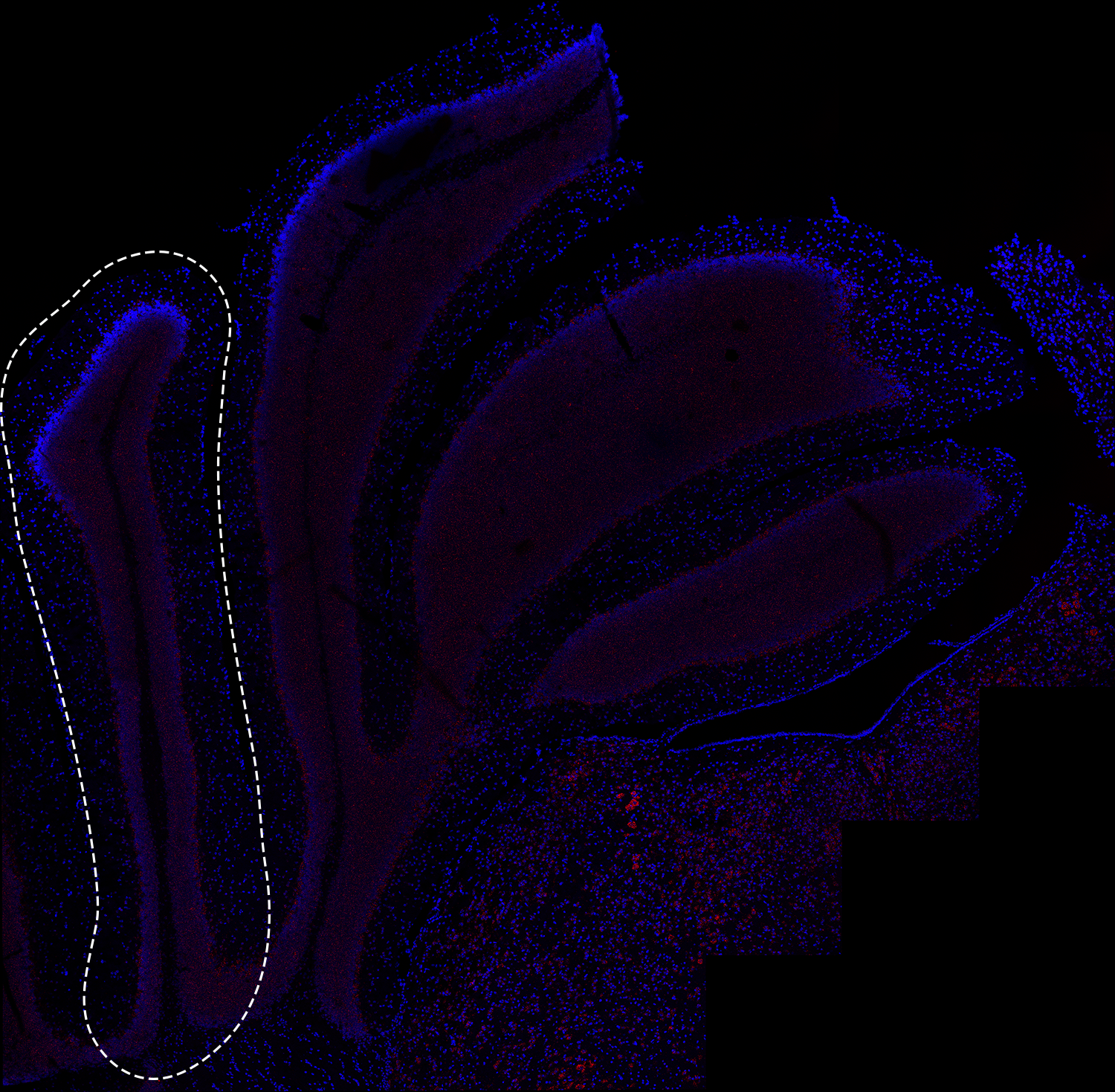

Supplement: Supplementary file 2 — EV Figures Source Data [file 44321_2024_54_MOESM2_ESM.zip › Raw_data_EV_figures/Figure EV1/Figure EV1B/CRBN-WT/CRBN WT - CB 4.tif]

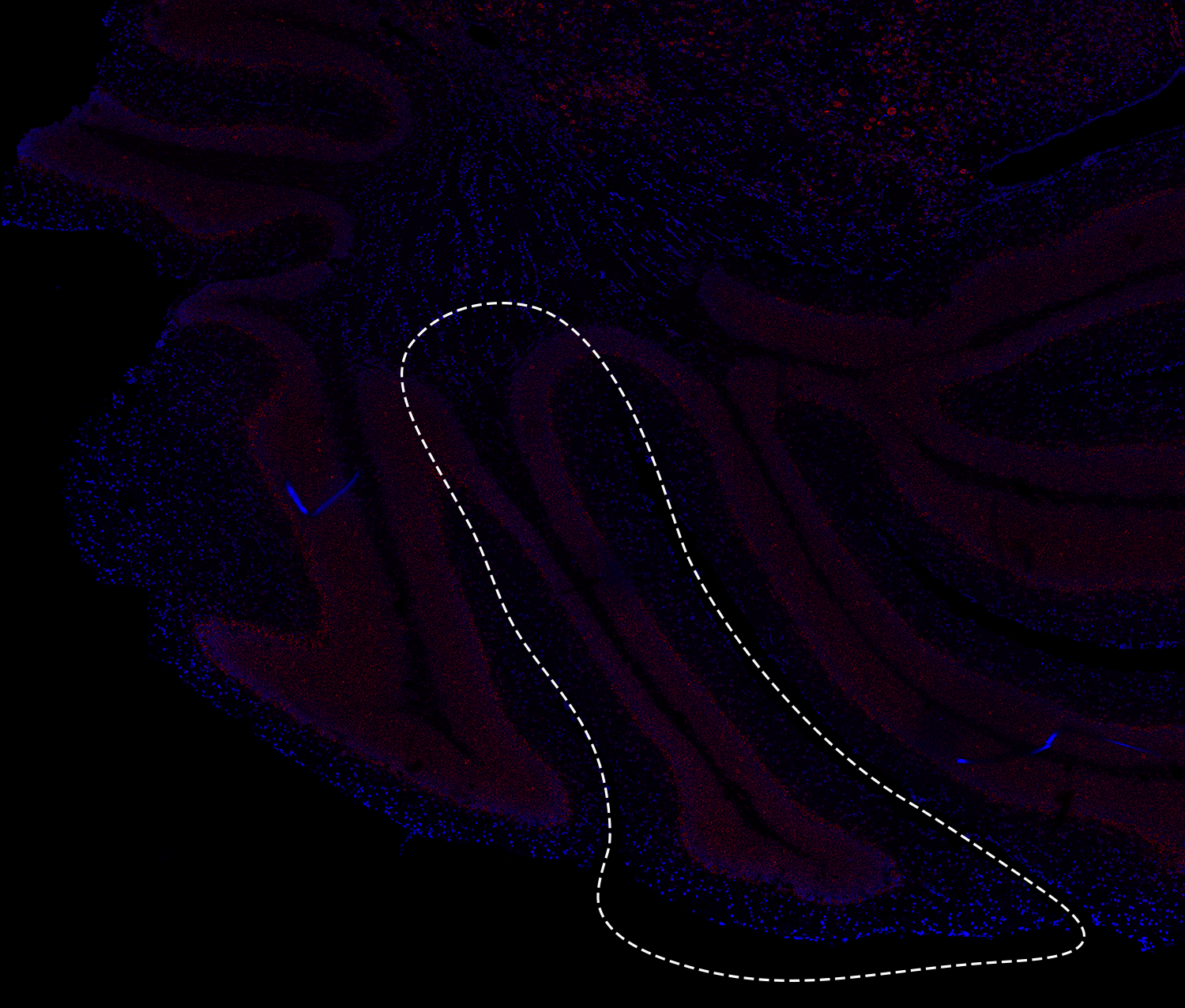

Supplement: Supplementary file 2 — EV Figures Source Data [file 44321_2024_54_MOESM2_ESM.zip › Raw_data_EV_figures/Figure EV1/Figure EV1B/CRBN-WT/CRBN WT - CB 5.tif]

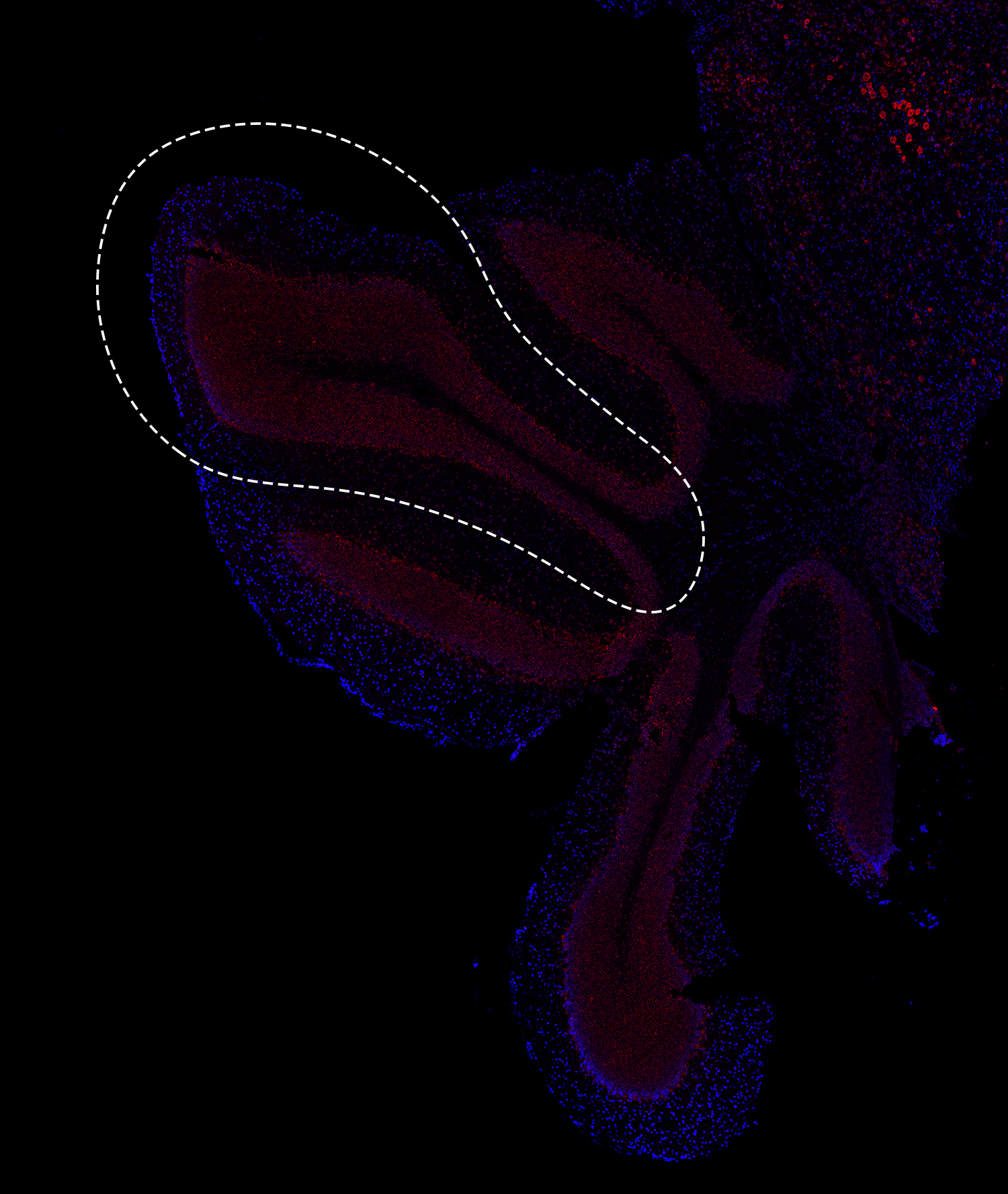

Supplement: Supplementary file 2 — EV Figures Source Data [file 44321_2024_54_MOESM2_ESM.zip › Raw_data_EV_figures/Figure EV1/Figure EV1B/CRBN-WT/CRBN WT - CB 6.tif]

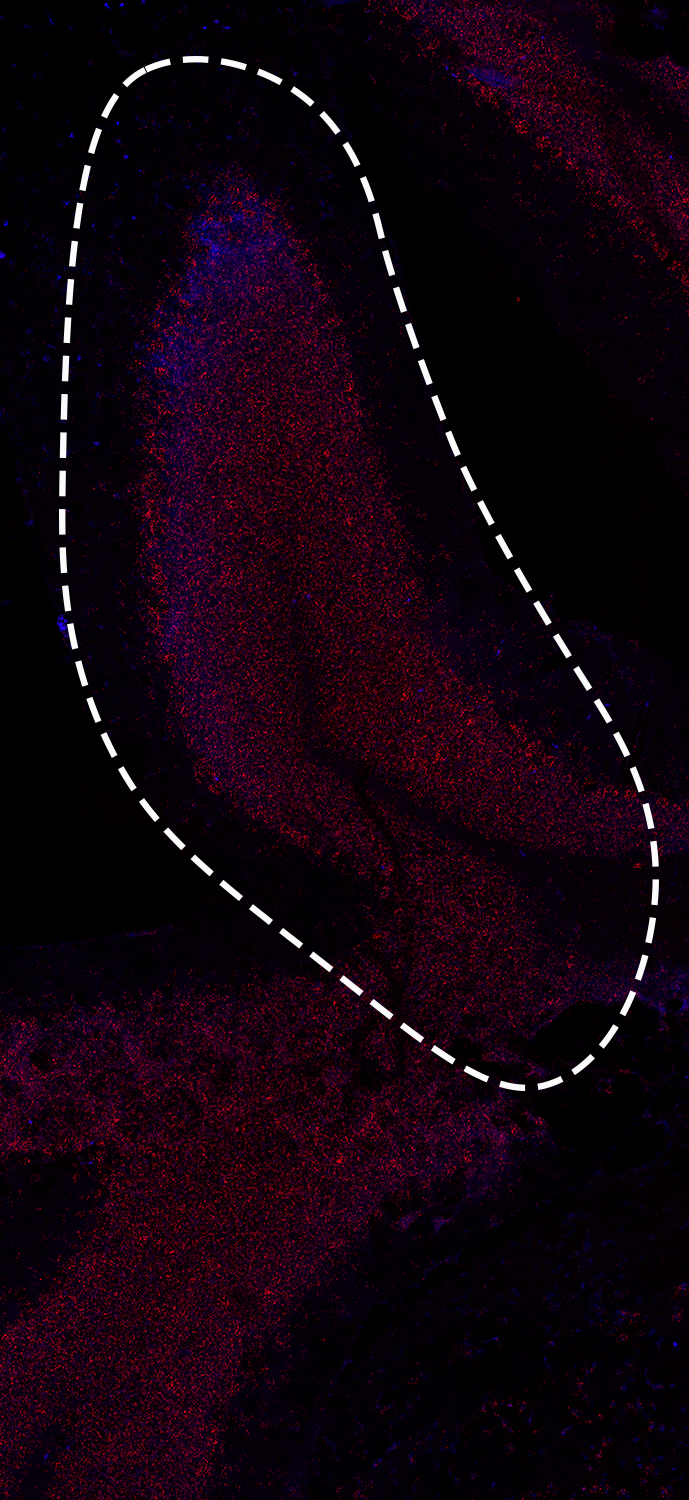

Supplement: Supplementary file 2 — EV Figures Source Data [file 44321_2024_54_MOESM2_ESM.zip › Raw_data_EV_figures/Figure EV1/Figure EV1B/GABA-CRBN-KO/GABA-CRBN-KO - CB 1.tif]

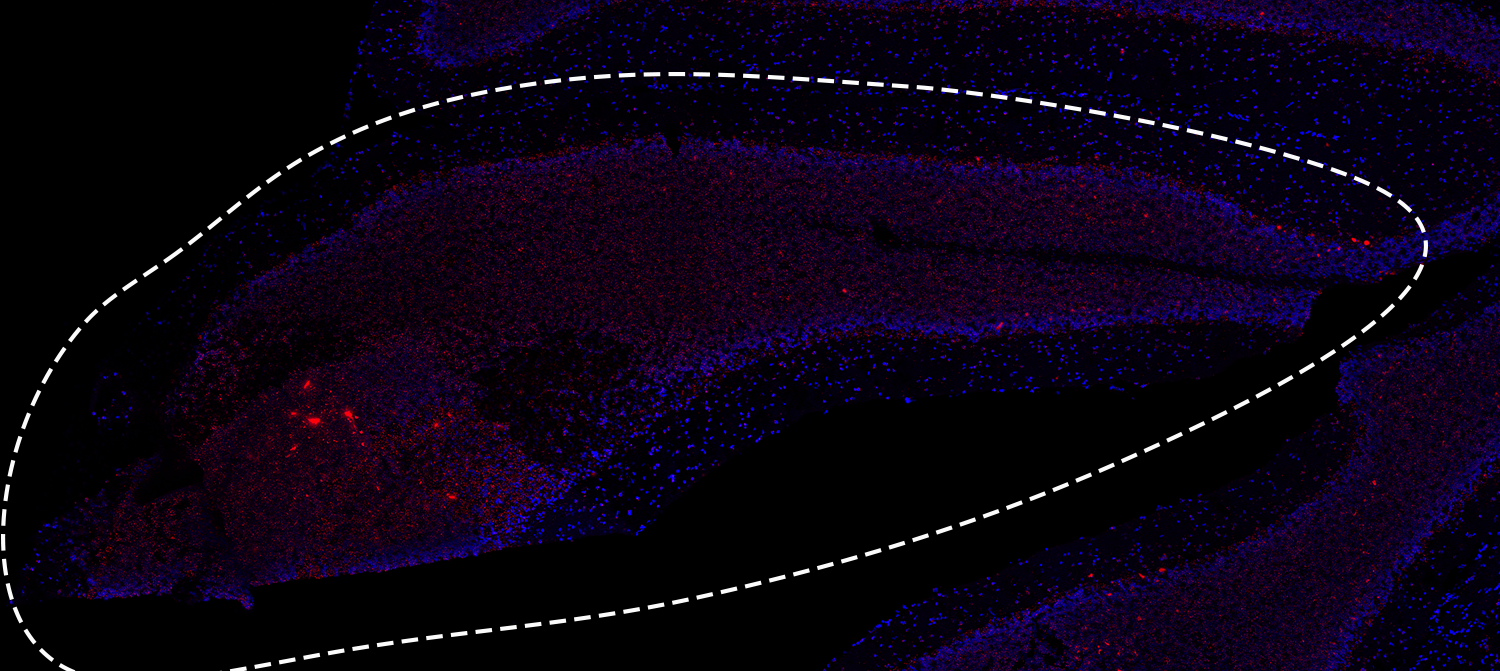

Supplement: Supplementary file 2 — EV Figures Source Data [file 44321_2024_54_MOESM2_ESM.zip › Raw_data_EV_figures/Figure EV1/Figure EV1B/GABA-CRBN-KO/GABA-CRBN-KO - CB 2.tif]

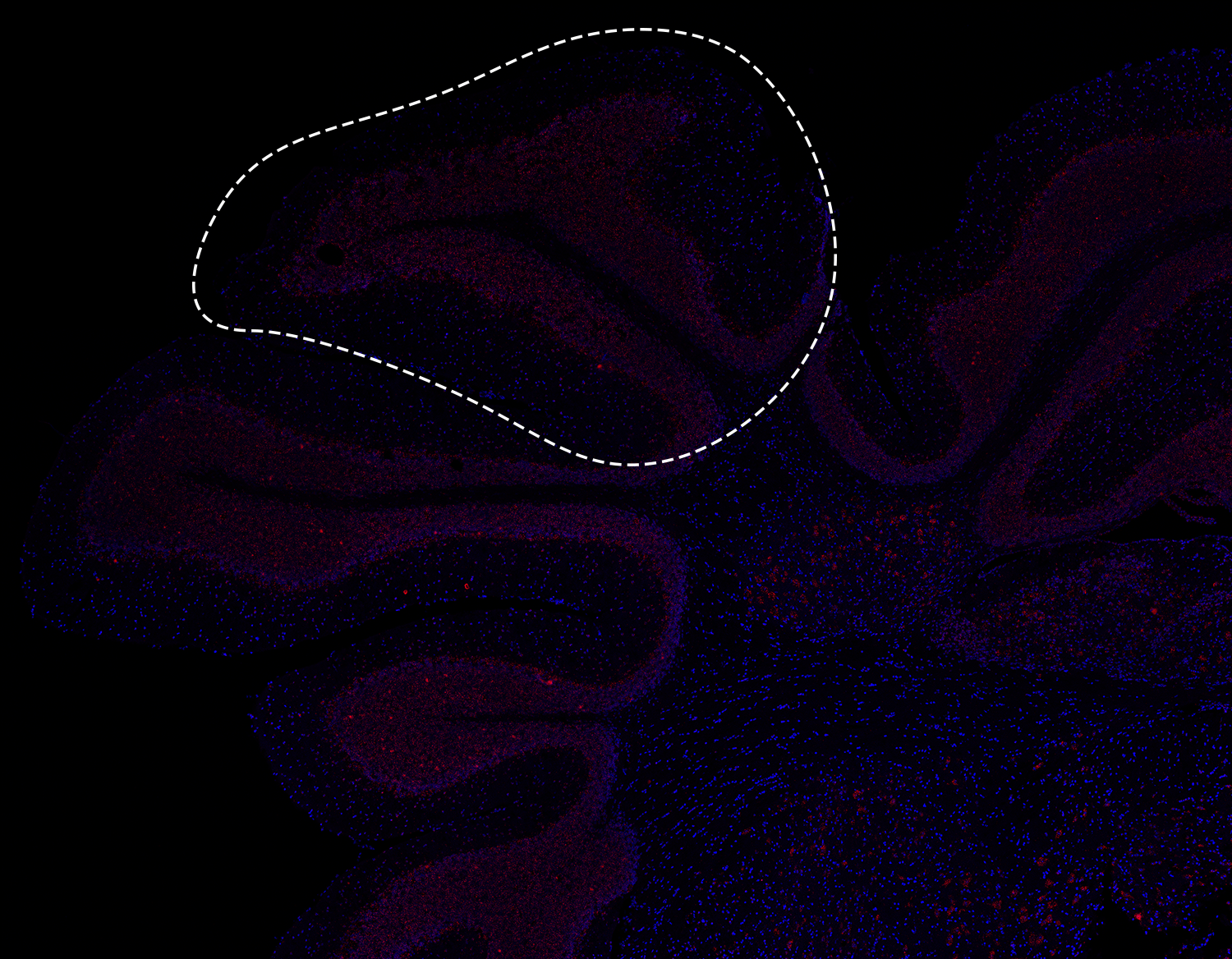

Supplement: Supplementary file 2 — EV Figures Source Data [file 44321_2024_54_MOESM2_ESM.zip › Raw_data_EV_figures/Figure EV1/Figure EV1B/GABA-CRBN-KO/GABA-CRBN-KO - CB 3.tif]

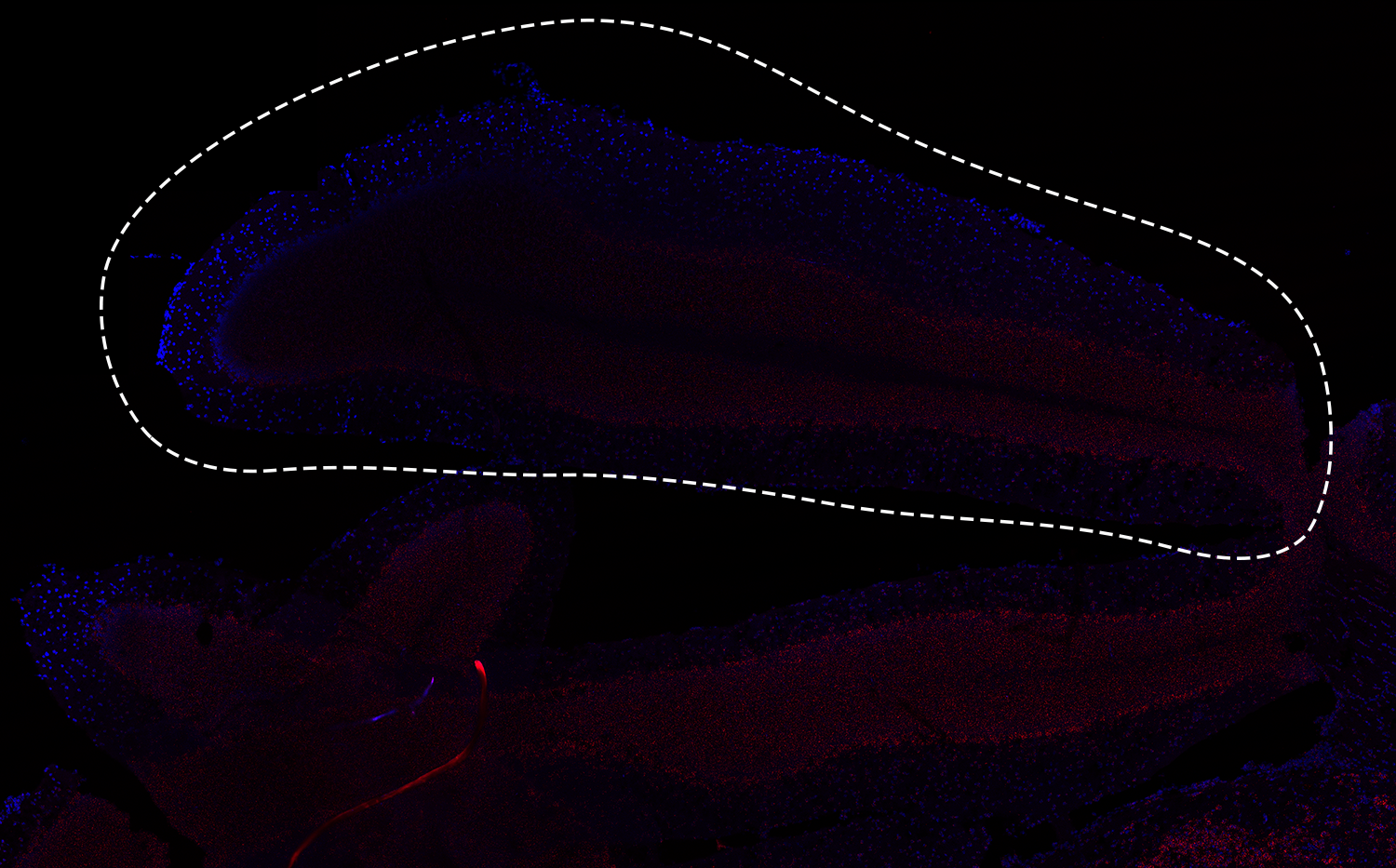

Supplement: Supplementary file 2 — EV Figures Source Data [file 44321_2024_54_MOESM2_ESM.zip › Raw_data_EV_figures/Figure EV1/Figure EV1B/Glu-CRBN-KO/Glu-CRBN-KO - CB 1.tif]

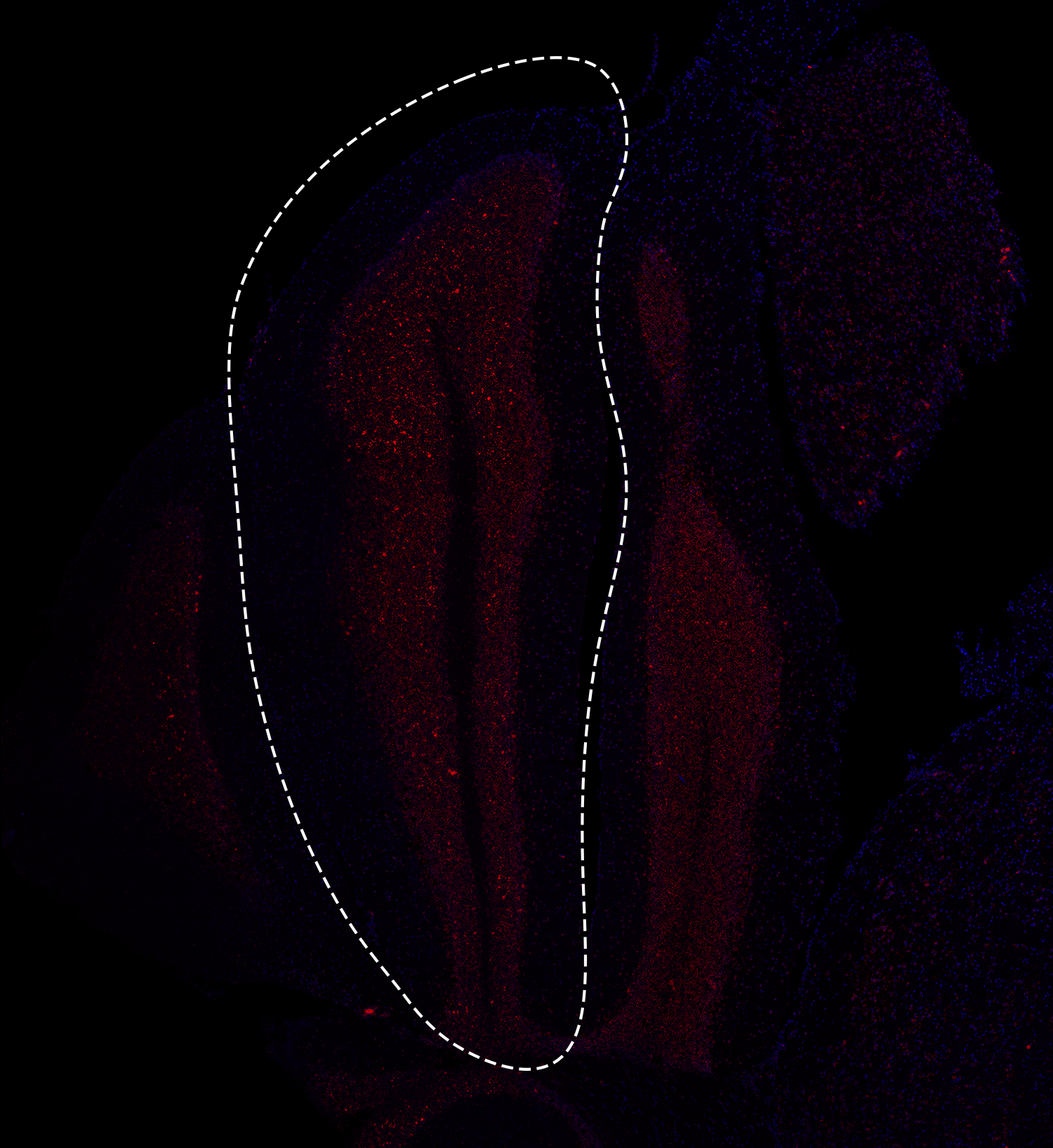

Supplement: Supplementary file 2 — EV Figures Source Data [file 44321_2024_54_MOESM2_ESM.zip › Raw_data_EV_figures/Figure EV1/Figure EV1B/Glu-CRBN-KO/Glu-CRBN-KO - CB 2.tif]

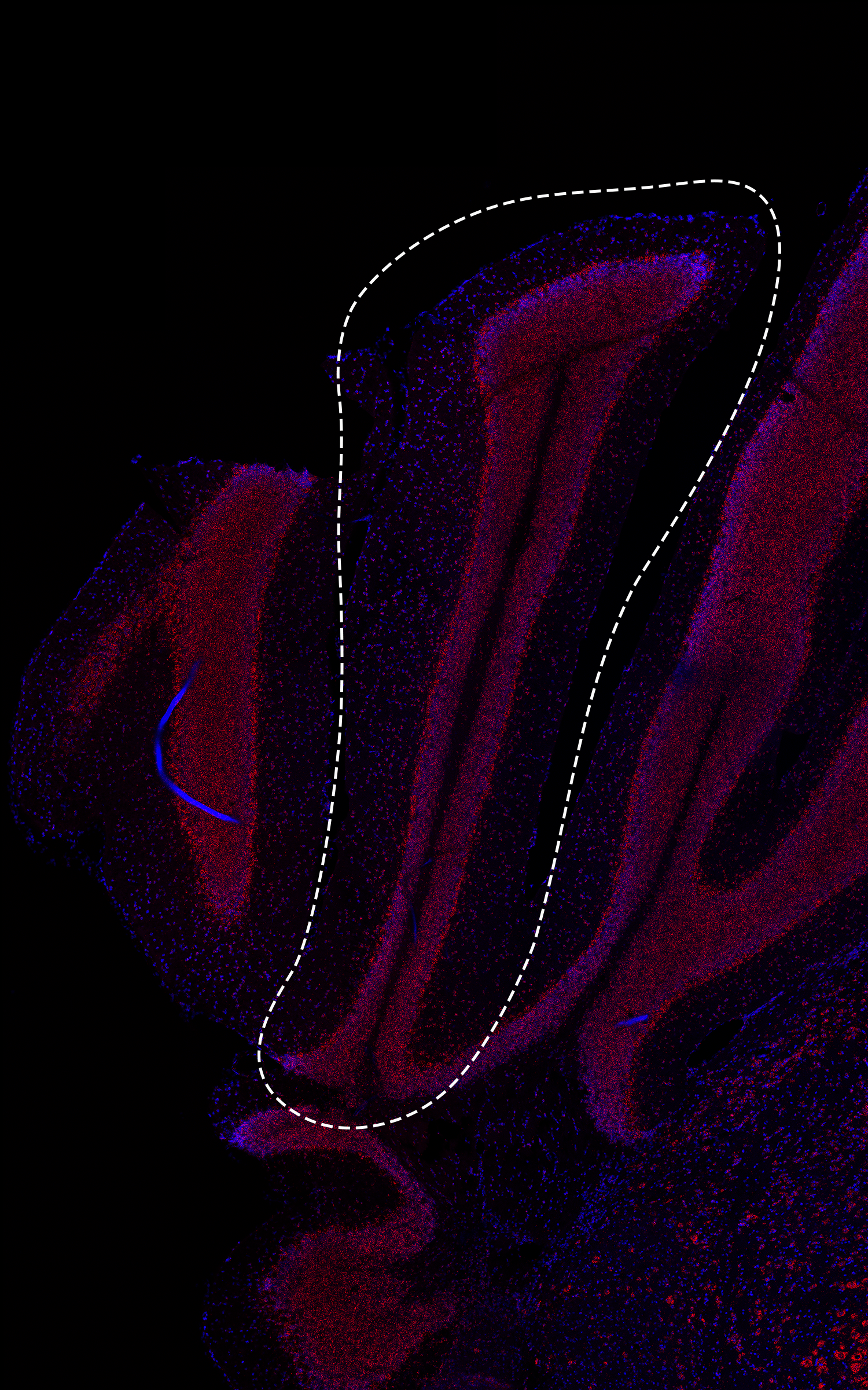

Supplement: Supplementary file 2 — EV Figures Source Data [file 44321_2024_54_MOESM2_ESM.zip › Raw_data_EV_figures/Figure EV1/Figure EV1B/Glu-CRBN-KO/Glu-CRBN-KO - CB 3.tif]

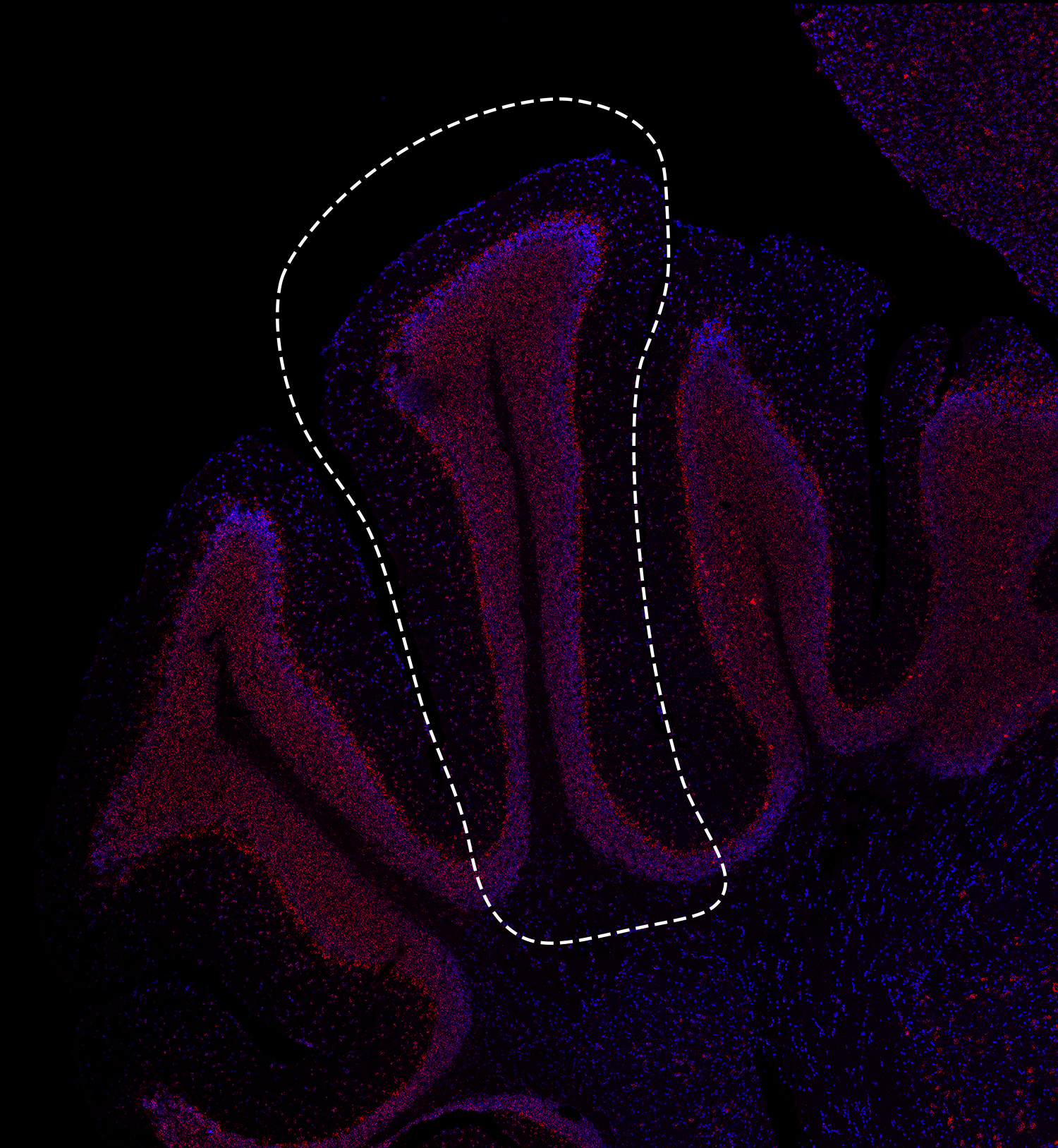

Supplement: Supplementary file 2 — EV Figures Source Data [file 44321_2024_54_MOESM2_ESM.zip › Raw_data_EV_figures/Figure EV1/Figure EV1B/Glu-CRBN-KO/Glu-CRBN-KO - CB 4.tif]

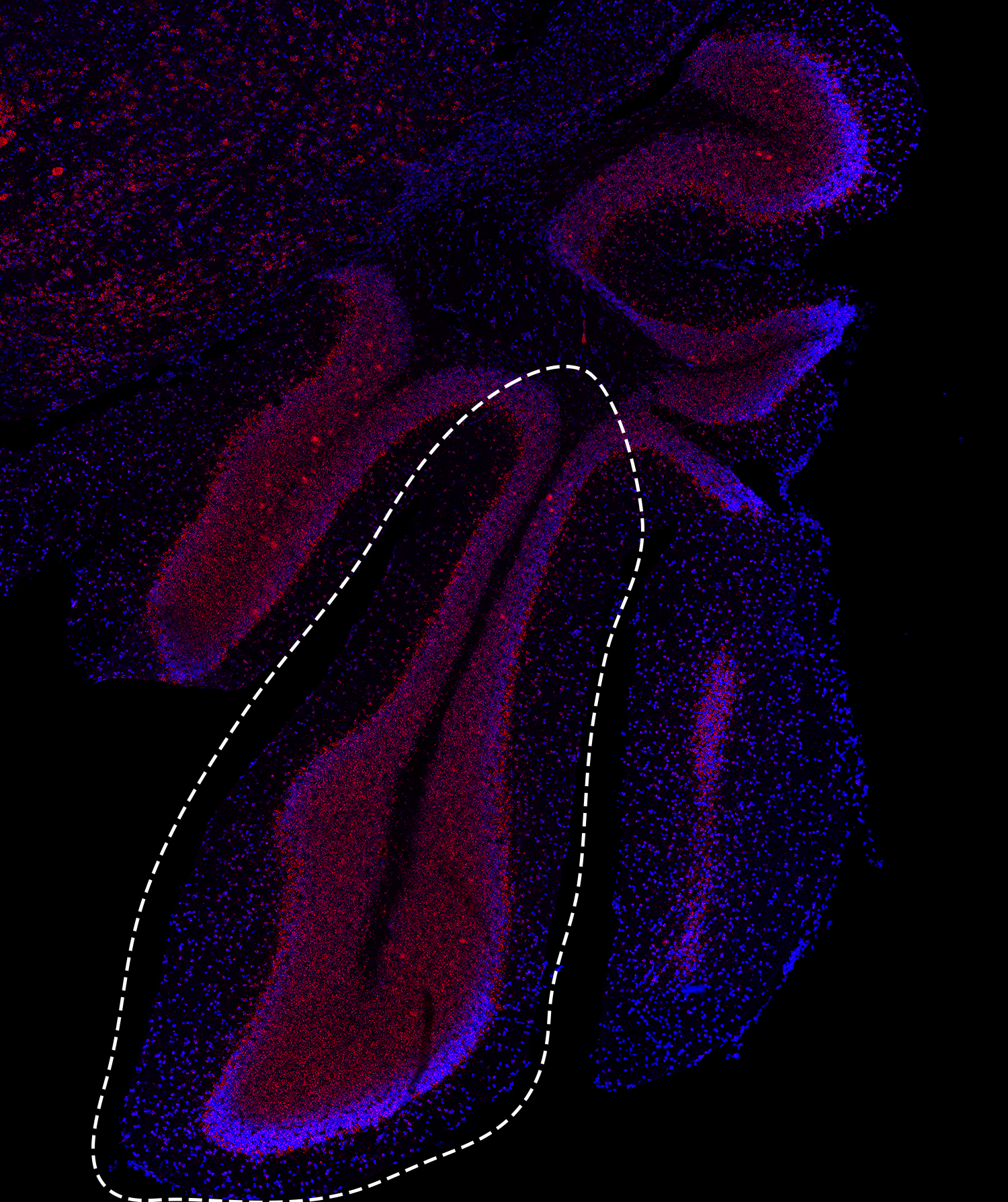

Supplement: Supplementary file 2 — EV Figures Source Data [file 44321_2024_54_MOESM2_ESM.zip › Raw_data_EV_figures/Figure EV1/Figure EV1B/Glu-CRBN-KO/Glu-CRBN-KO - CB 5.tif]

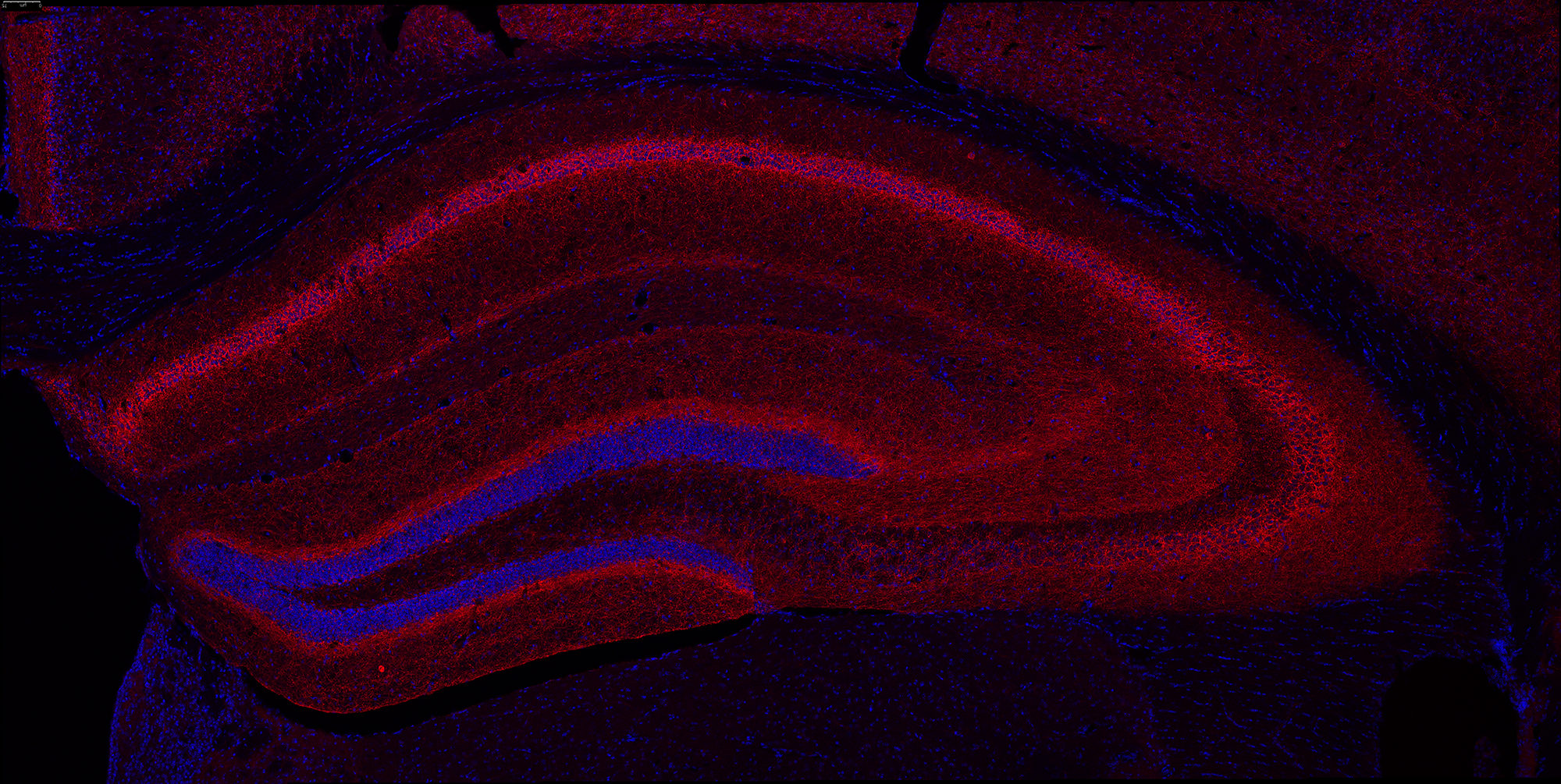

Supplement: Supplementary file 2 — EV Figures Source Data [file 44321_2024_54_MOESM2_ESM.zip › Raw_data_EV_figures/Figure EV4/Figure EV4B/KO 1.tif]

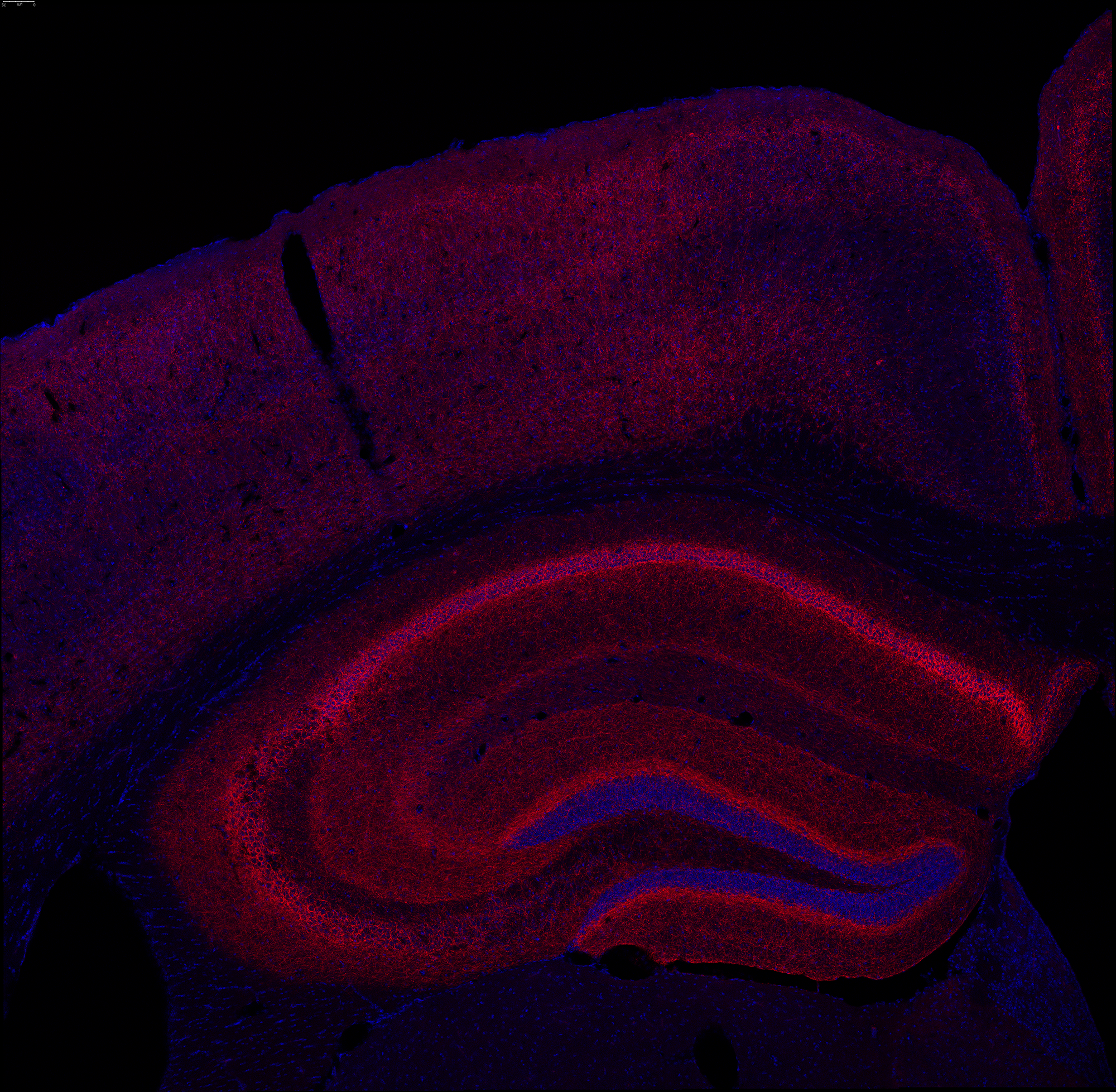

Supplement: Supplementary file 2 — EV Figures Source Data [file 44321_2024_54_MOESM2_ESM.zip › Raw_data_EV_figures/Figure EV4/Figure EV4B/KO 2.tif]

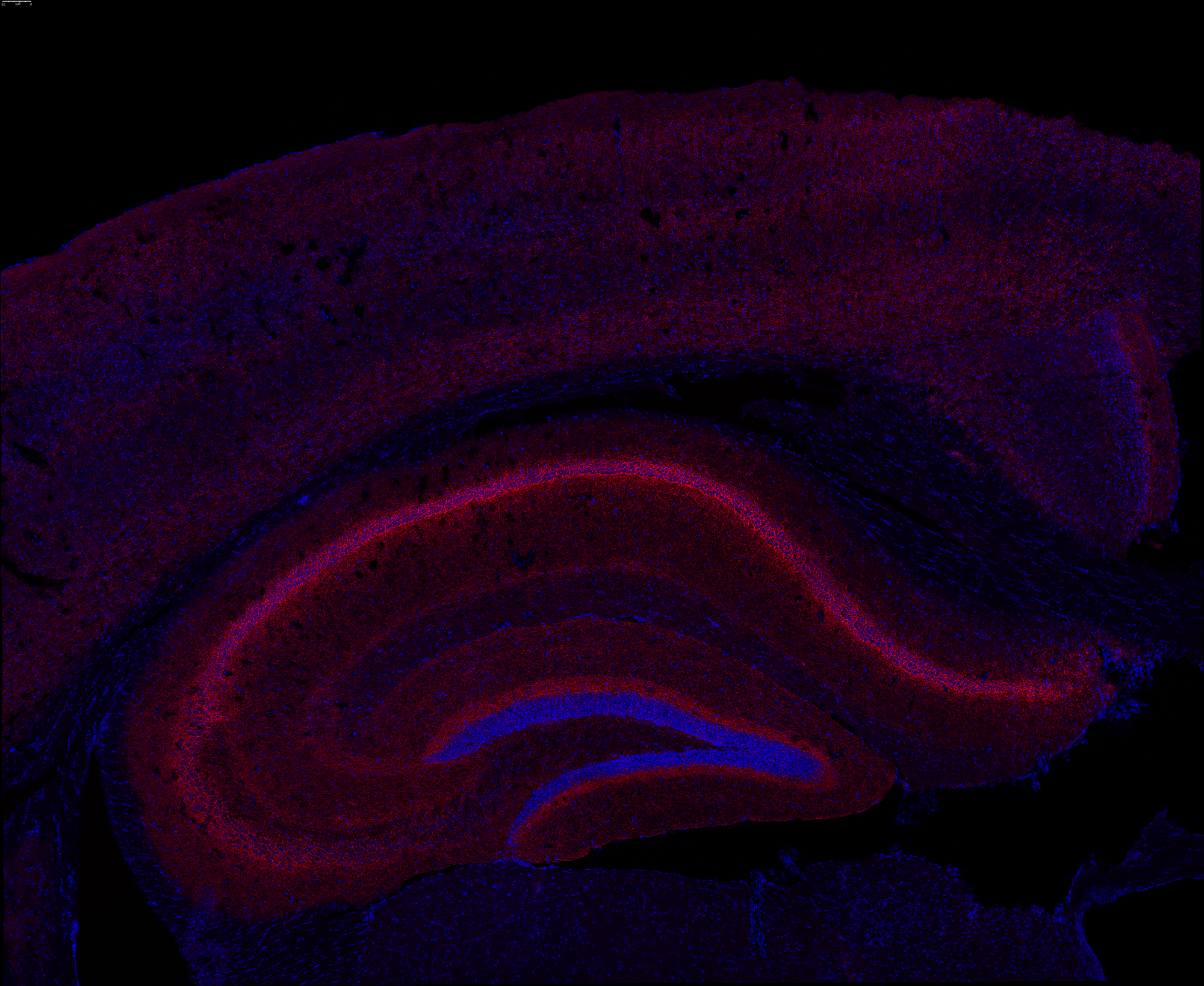

Supplement: Supplementary file 2 — EV Figures Source Data [file 44321_2024_54_MOESM2_ESM.zip › Raw_data_EV_figures/Figure EV4/Figure EV4B/KO 3.tif]

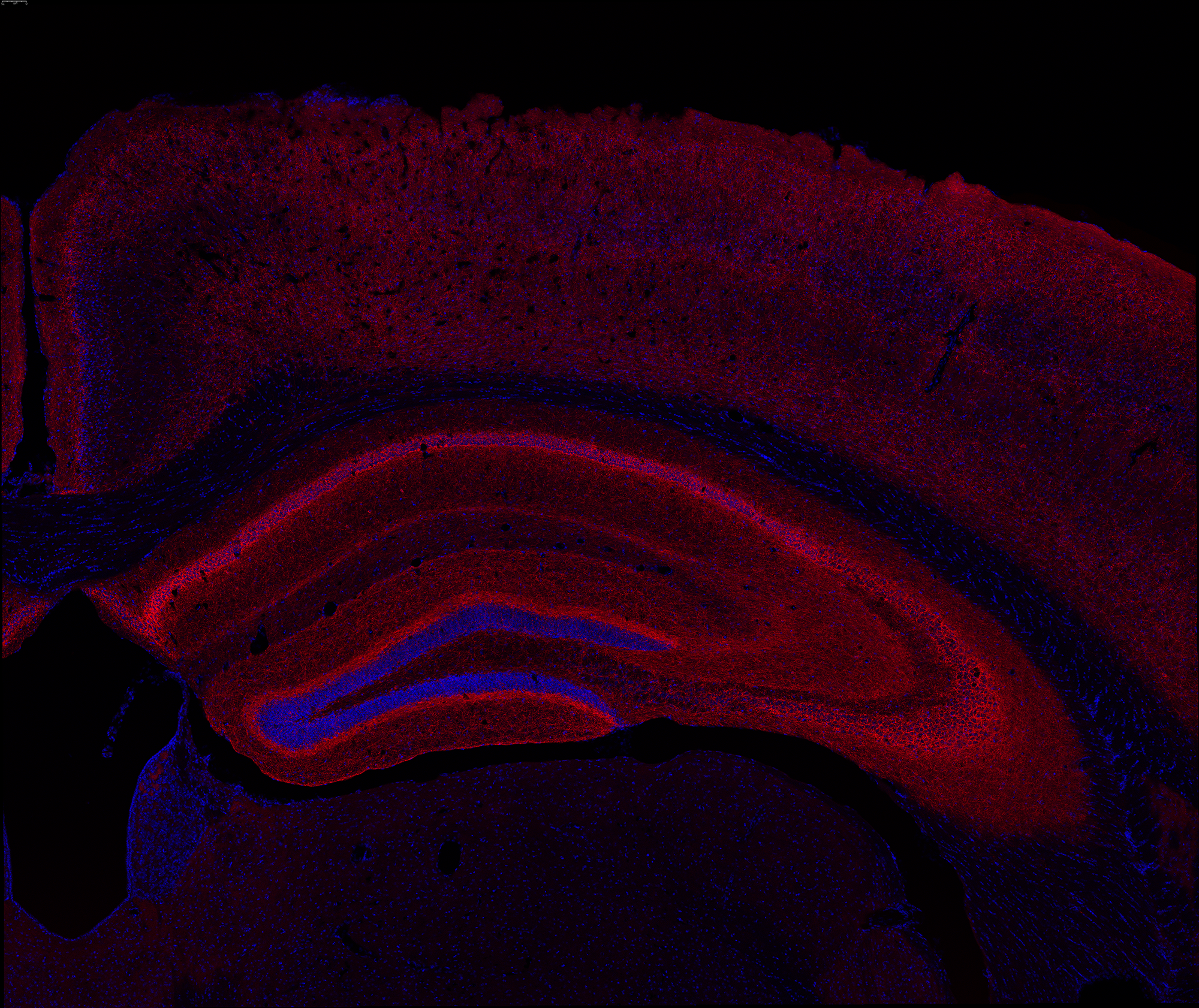

Supplement: Supplementary file 2 — EV Figures Source Data [file 44321_2024_54_MOESM2_ESM.zip › Raw_data_EV_figures/Figure EV4/Figure EV4B/WT 1.tif]

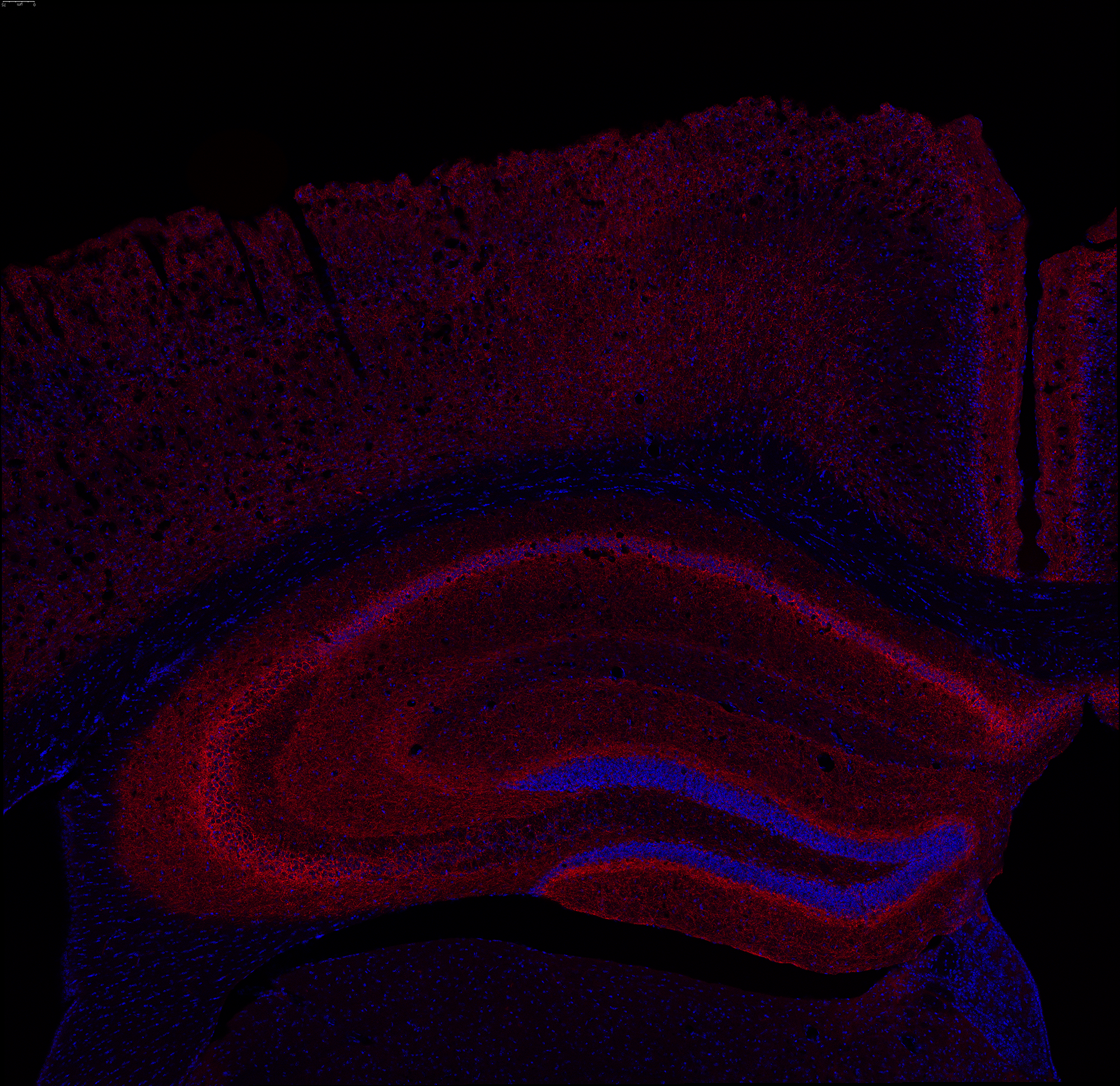

Supplement: Supplementary file 2 — EV Figures Source Data [file 44321_2024_54_MOESM2_ESM.zip › Raw_data_EV_figures/Figure EV4/Figure EV4B/WT 2.tif]

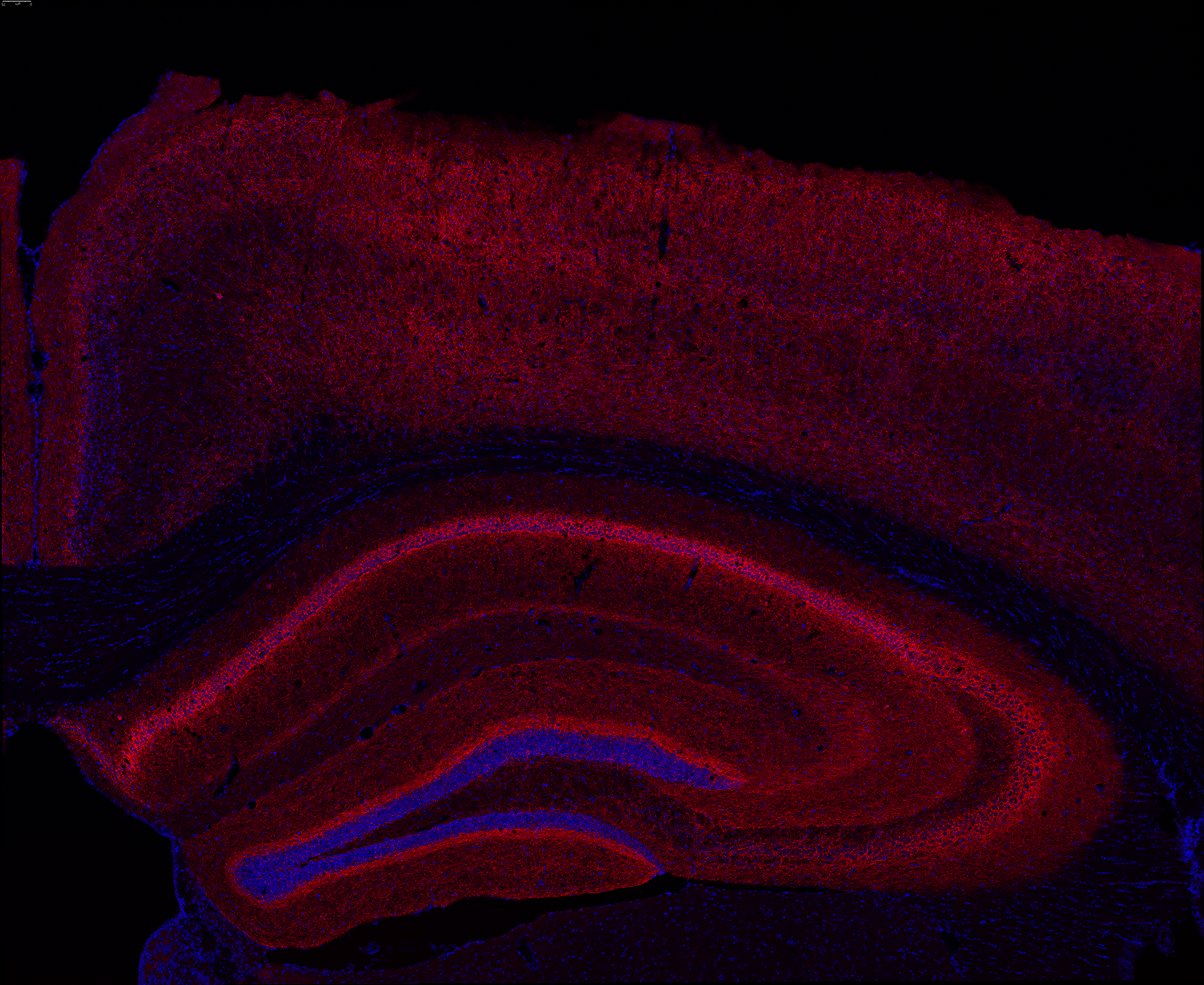

Supplement: Supplementary file 2 — EV Figures Source Data [file 44321_2024_54_MOESM2_ESM.zip › Raw_data_EV_figures/Figure EV4/Figure EV4B/WT 3.tif]

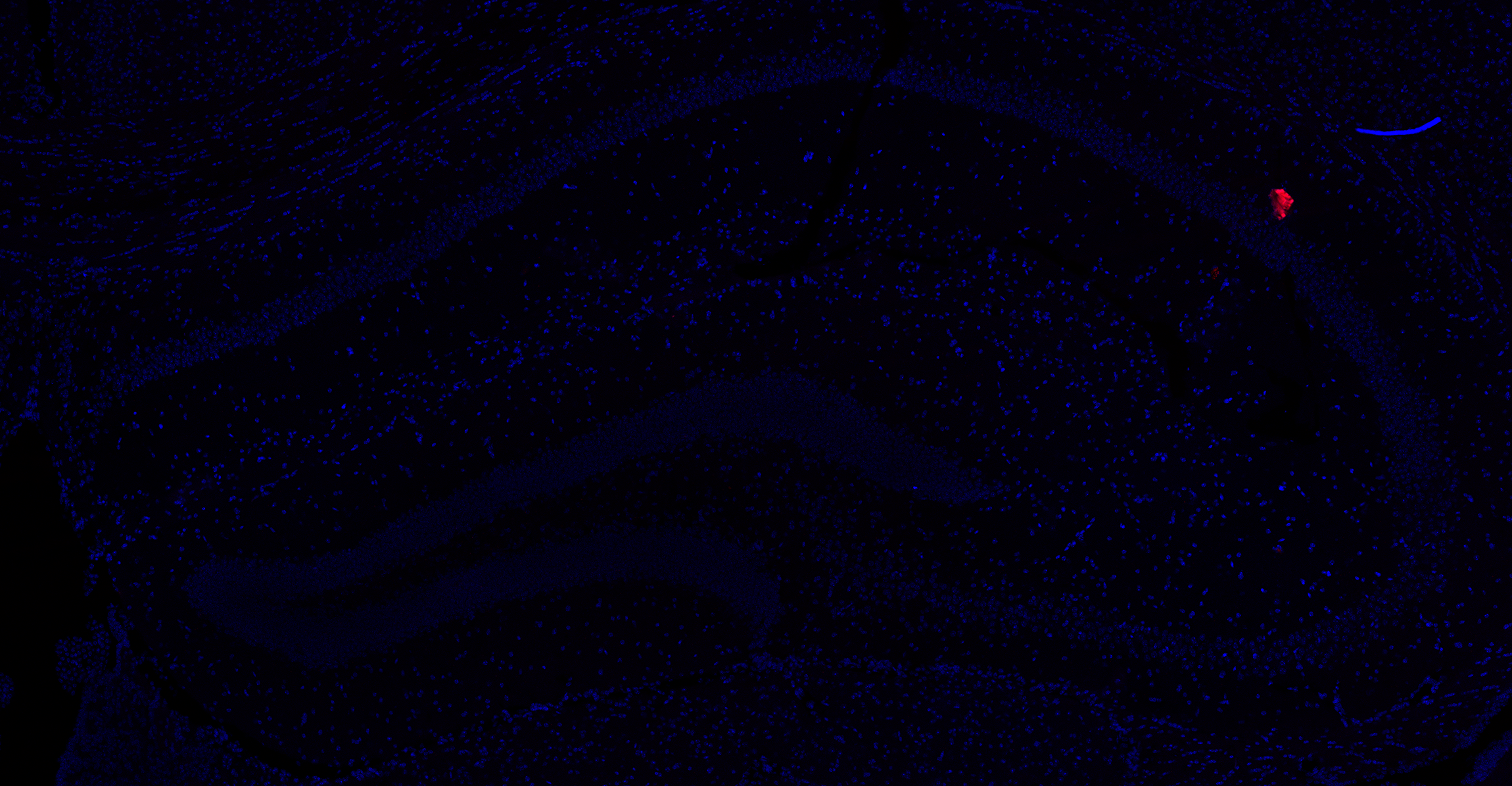

Supplement: Supplementary file 3 — Source data Fig. 1 [file 44321_2024_54_MOESM3_ESM.zip › Figure 1/1B/CRBN-KO/CRBN-KO - HC 1.tif]

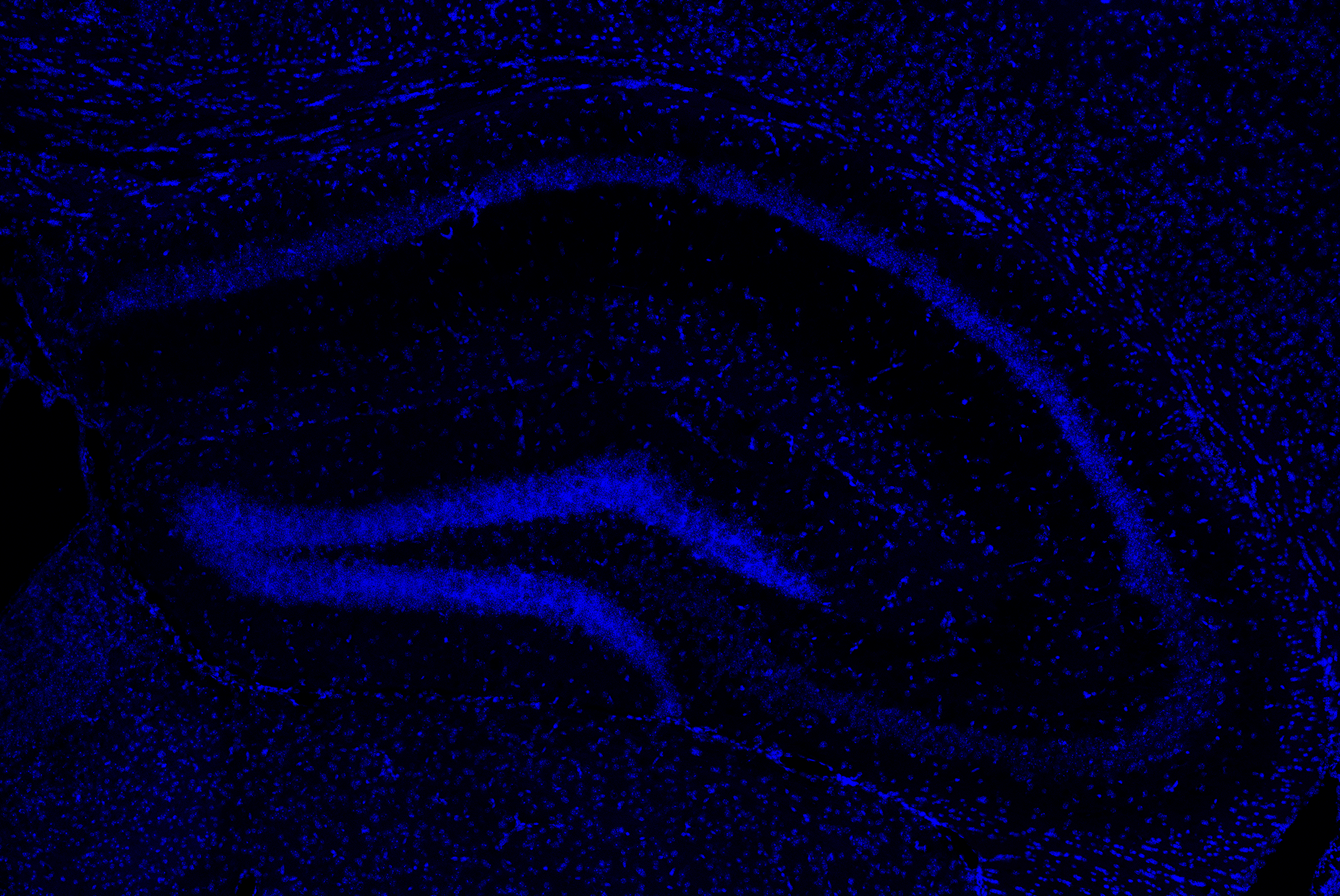

Supplement: Supplementary file 3 — Source data Fig. 1 [file 44321_2024_54_MOESM3_ESM.zip › Figure 1/1B/CRBN-KO/CRBN-KO - HC 2.tif]

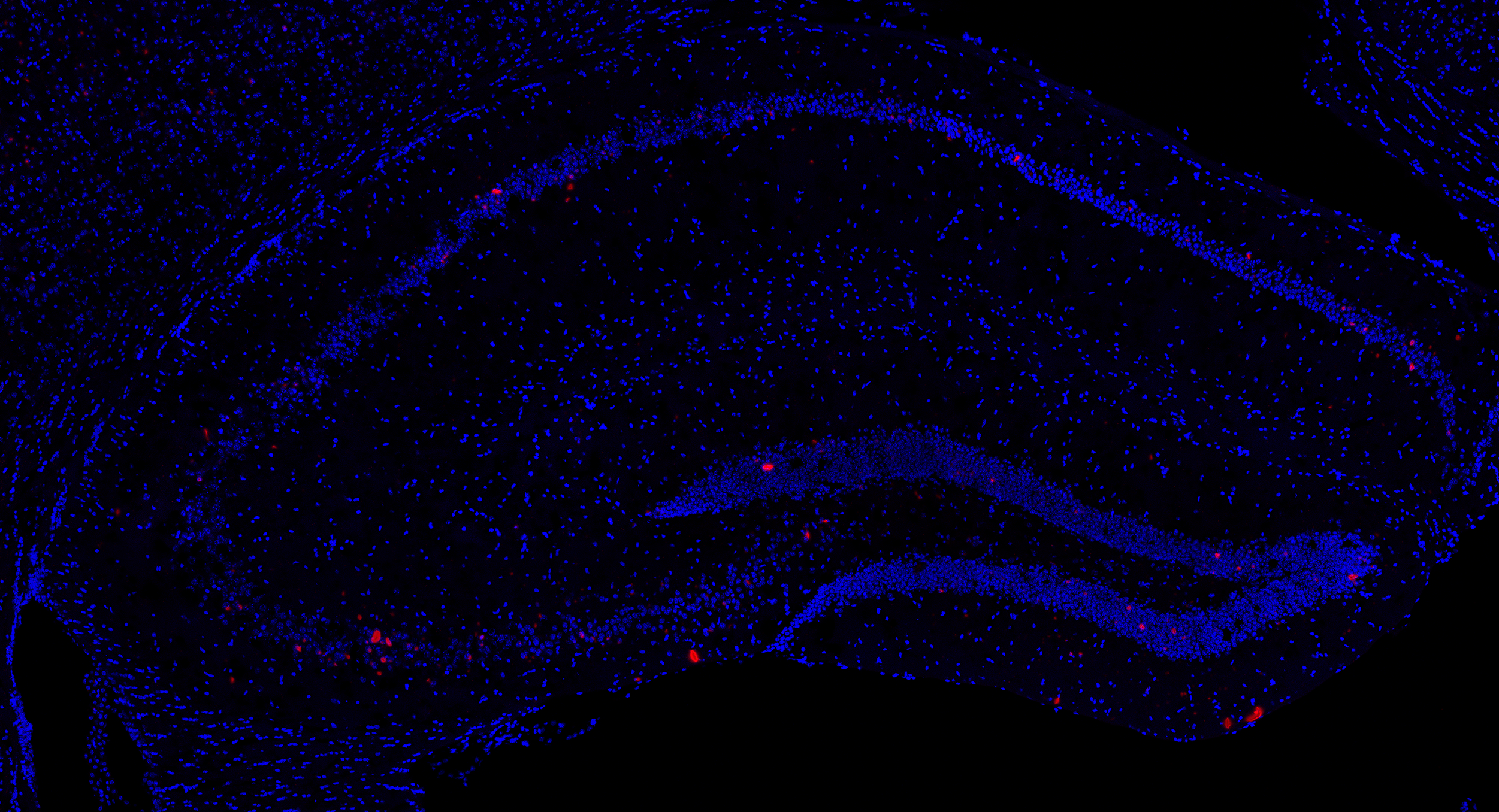

Supplement: Supplementary file 3 — Source data Fig. 1 [file 44321_2024_54_MOESM3_ESM.zip › Figure 1/1B/CRBN-KO/CRBN-KO - HC 3.tif]

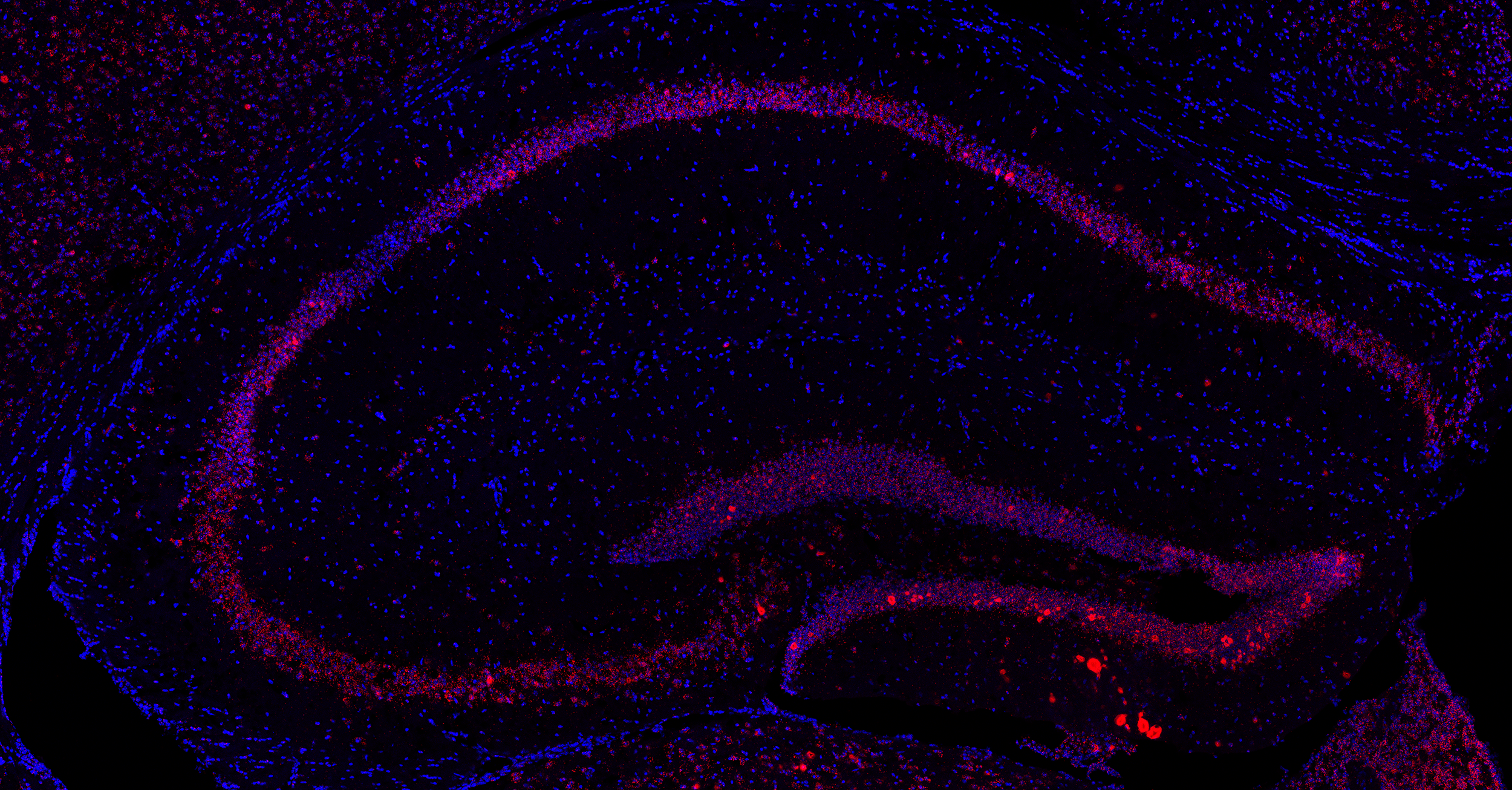

Supplement: Supplementary file 3 — Source data Fig. 1 [file 44321_2024_54_MOESM3_ESM.zip › Figure 1/1B/CRBN-WT/CRBN-WT - HC 1.tif]

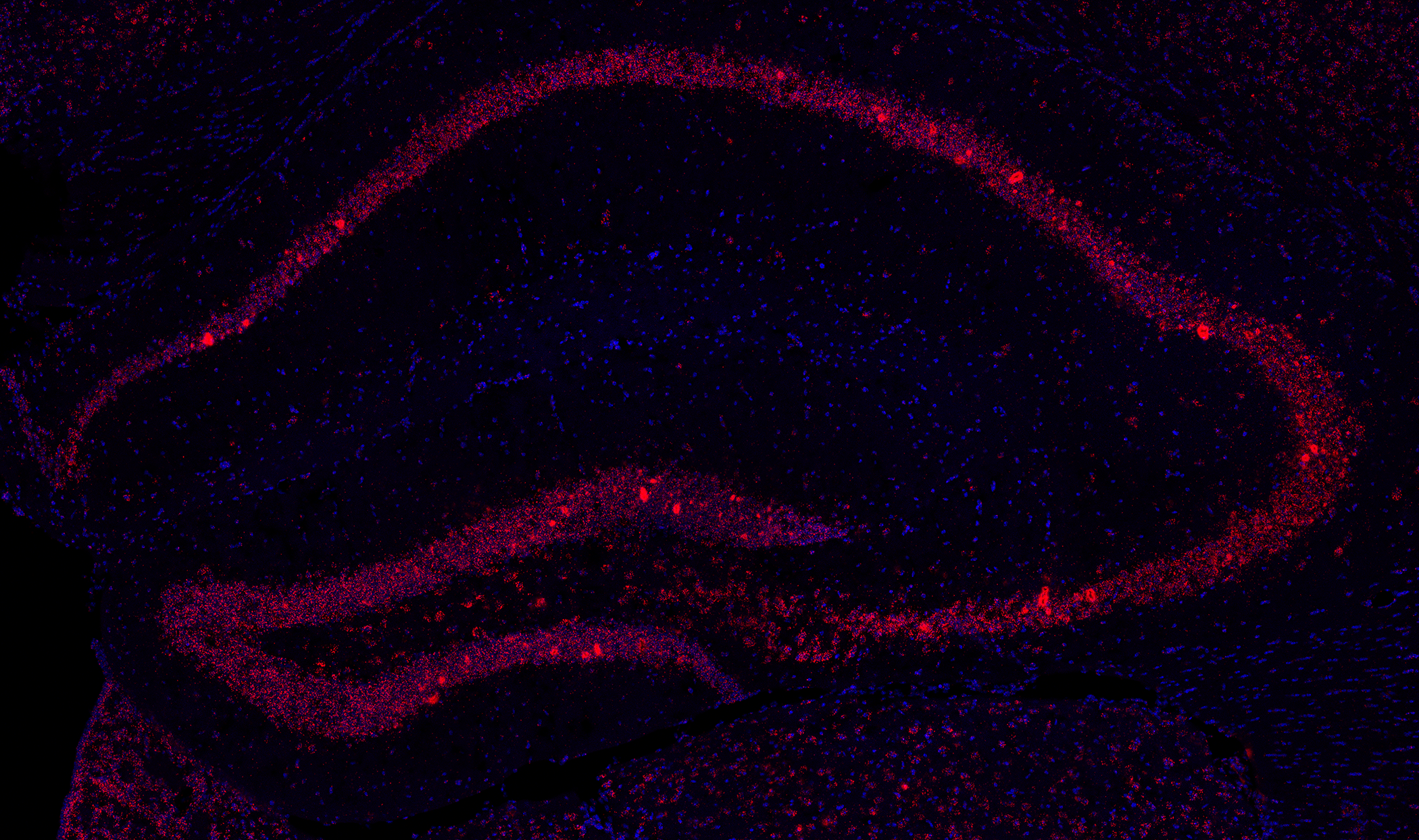

Supplement: Supplementary file 3 — Source data Fig. 1 [file 44321_2024_54_MOESM3_ESM.zip › Figure 1/1B/CRBN-WT/CRBN-WT - HC 2.tif]

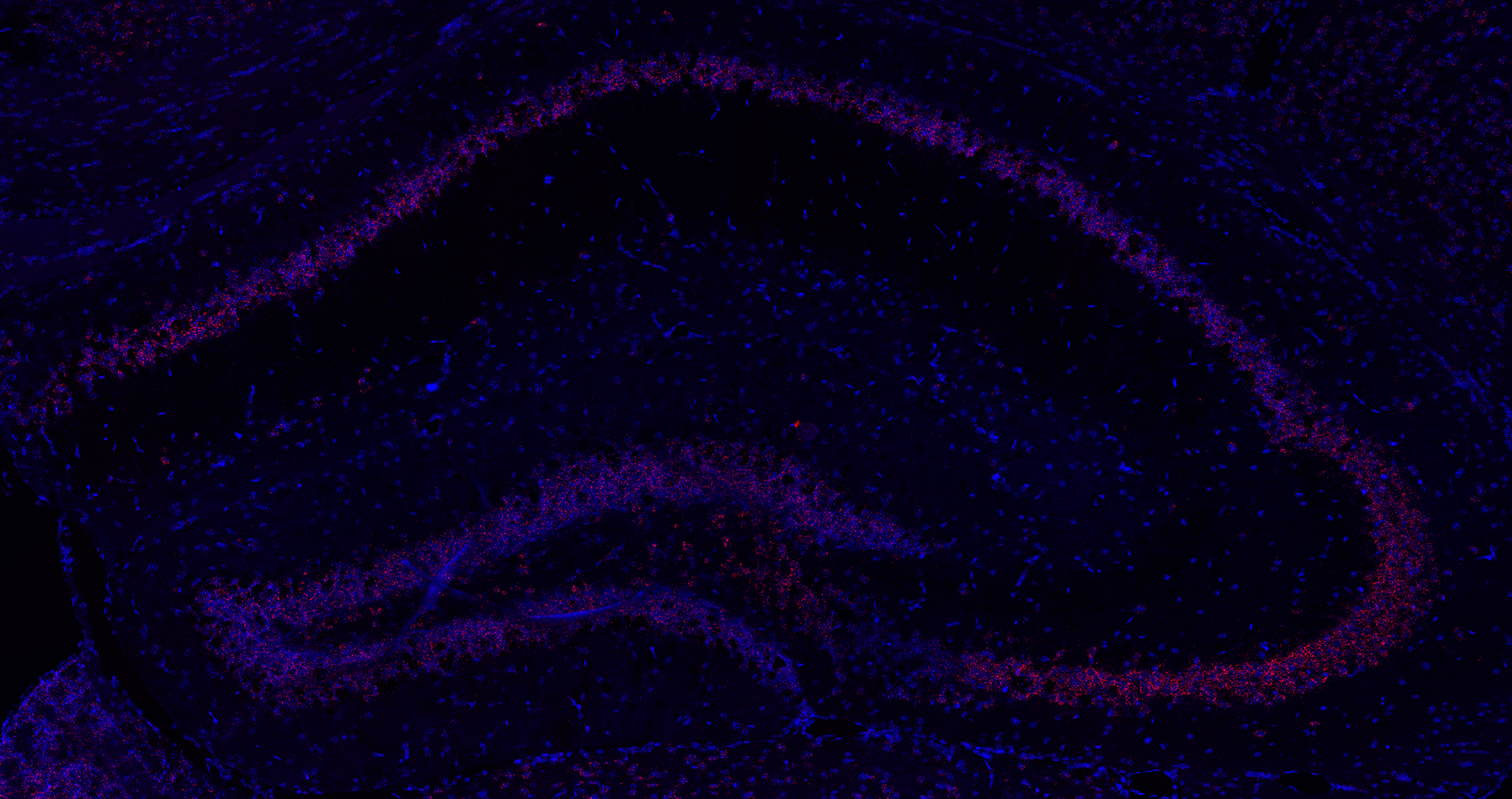

Supplement: Supplementary file 3 — Source data Fig. 1 [file 44321_2024_54_MOESM3_ESM.zip › Figure 1/1B/CRBN-WT/CRBN-WT - HC 3.tif]

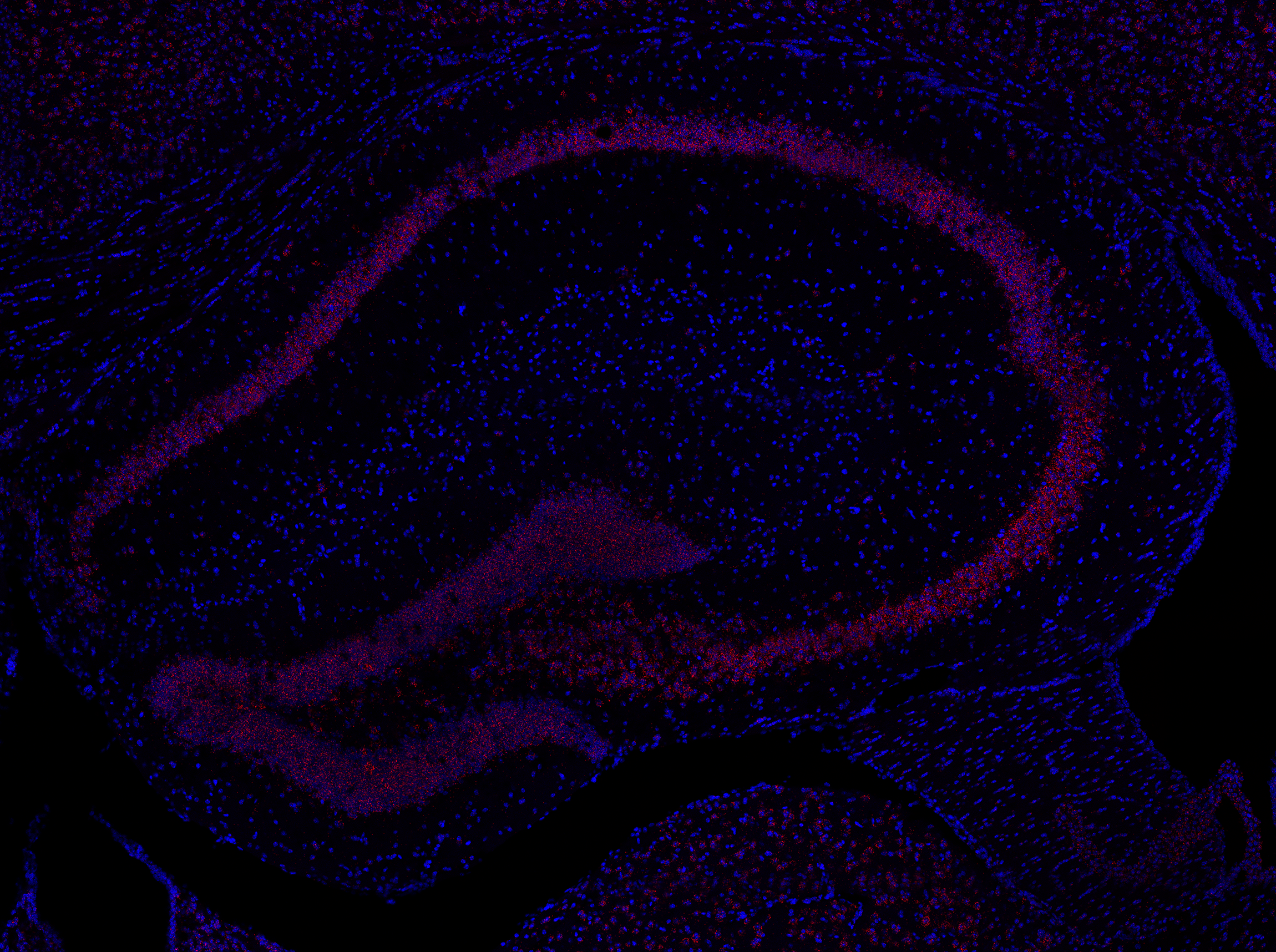

Supplement: Supplementary file 3 — Source data Fig. 1 [file 44321_2024_54_MOESM3_ESM.zip › Figure 1/1B/CRBN-WT/CRBN-WT - HC 4.tif]

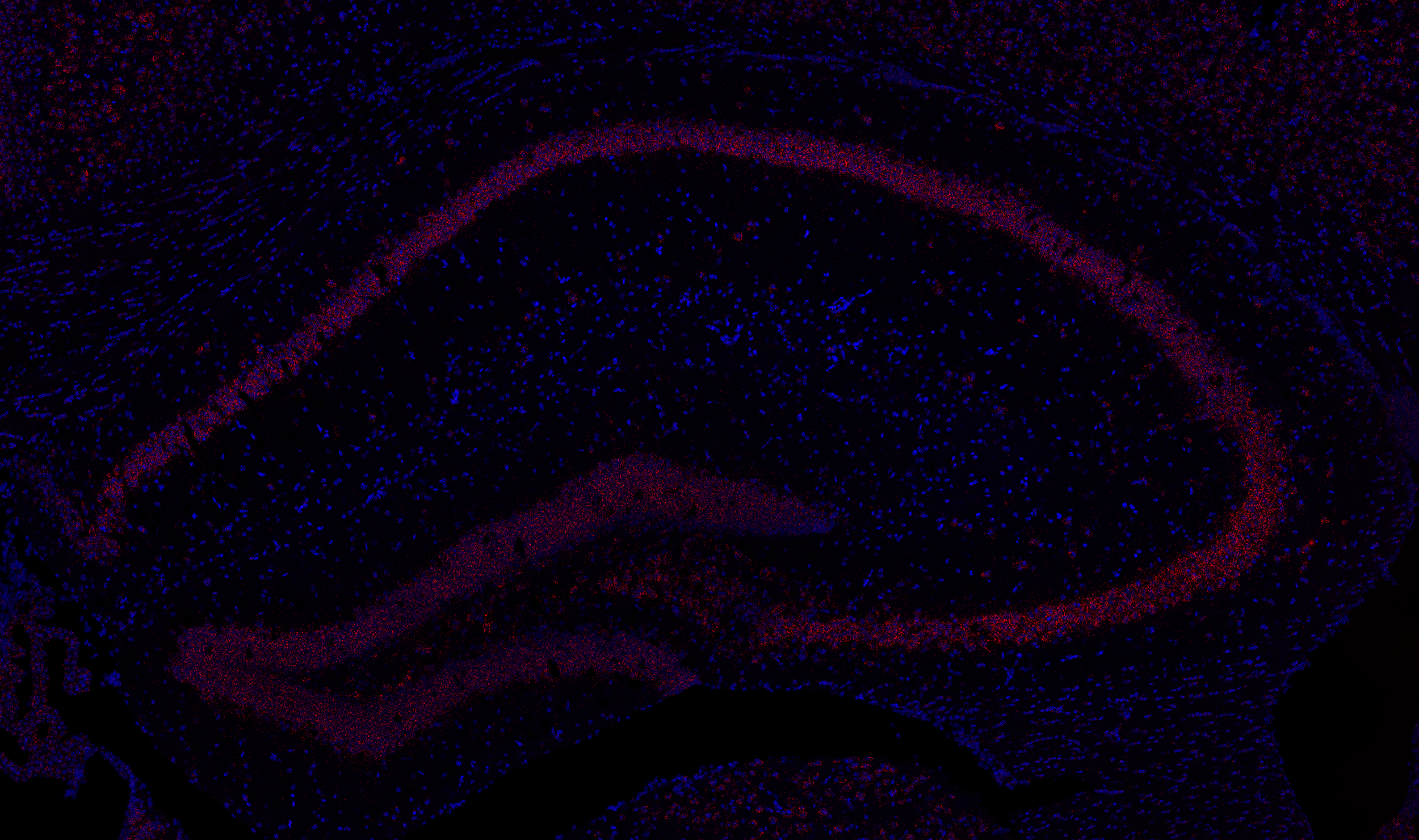

Supplement: Supplementary file 3 — Source data Fig. 1 [file 44321_2024_54_MOESM3_ESM.zip › Figure 1/1B/CRBN-WT/CRBN-WT - HC 5.tif]

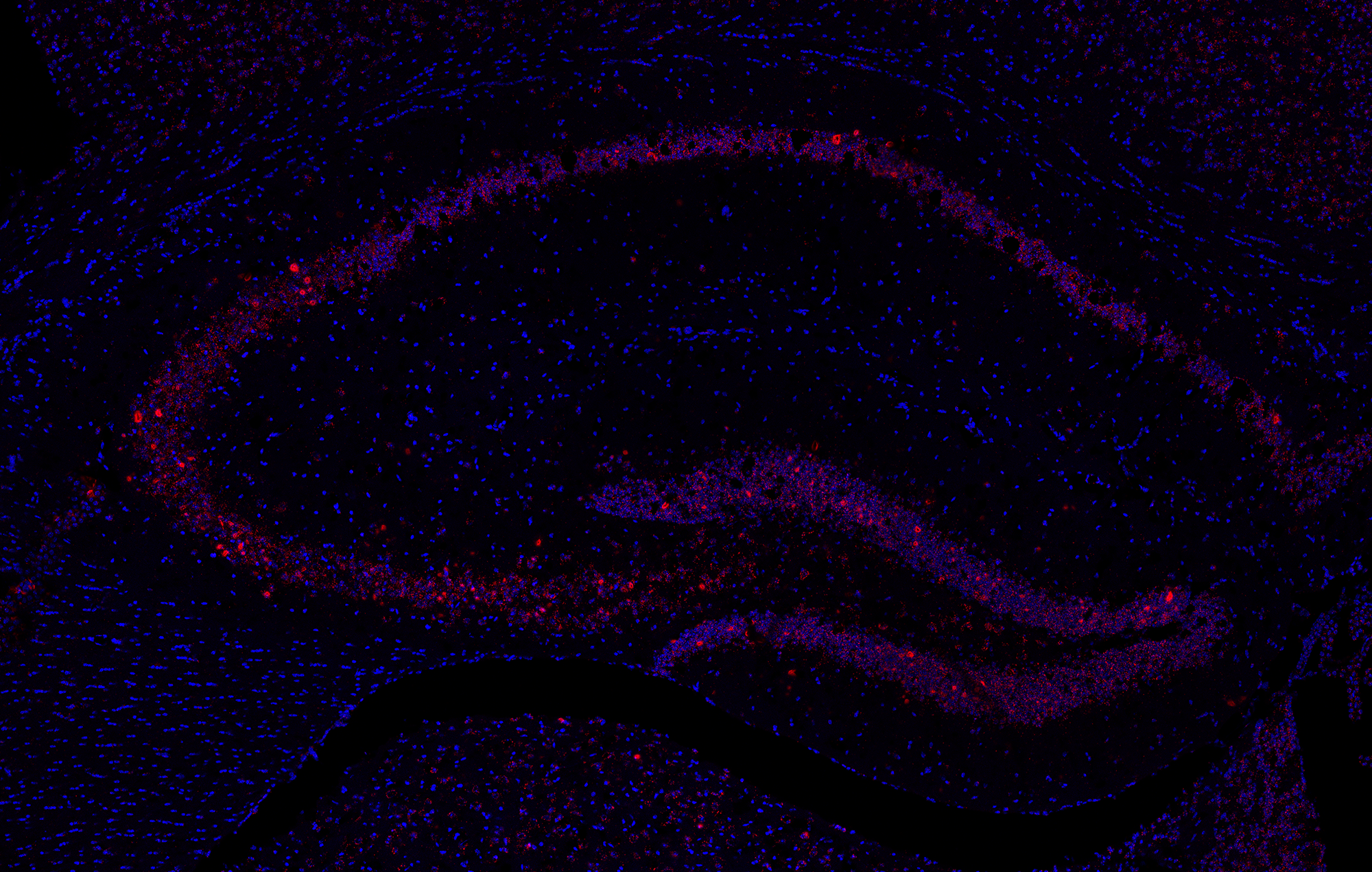

Supplement: Supplementary file 3 — Source data Fig. 1 [file 44321_2024_54_MOESM3_ESM.zip › Figure 1/1B/CRBN-WT/CRBN-WT - HC 6.tif]

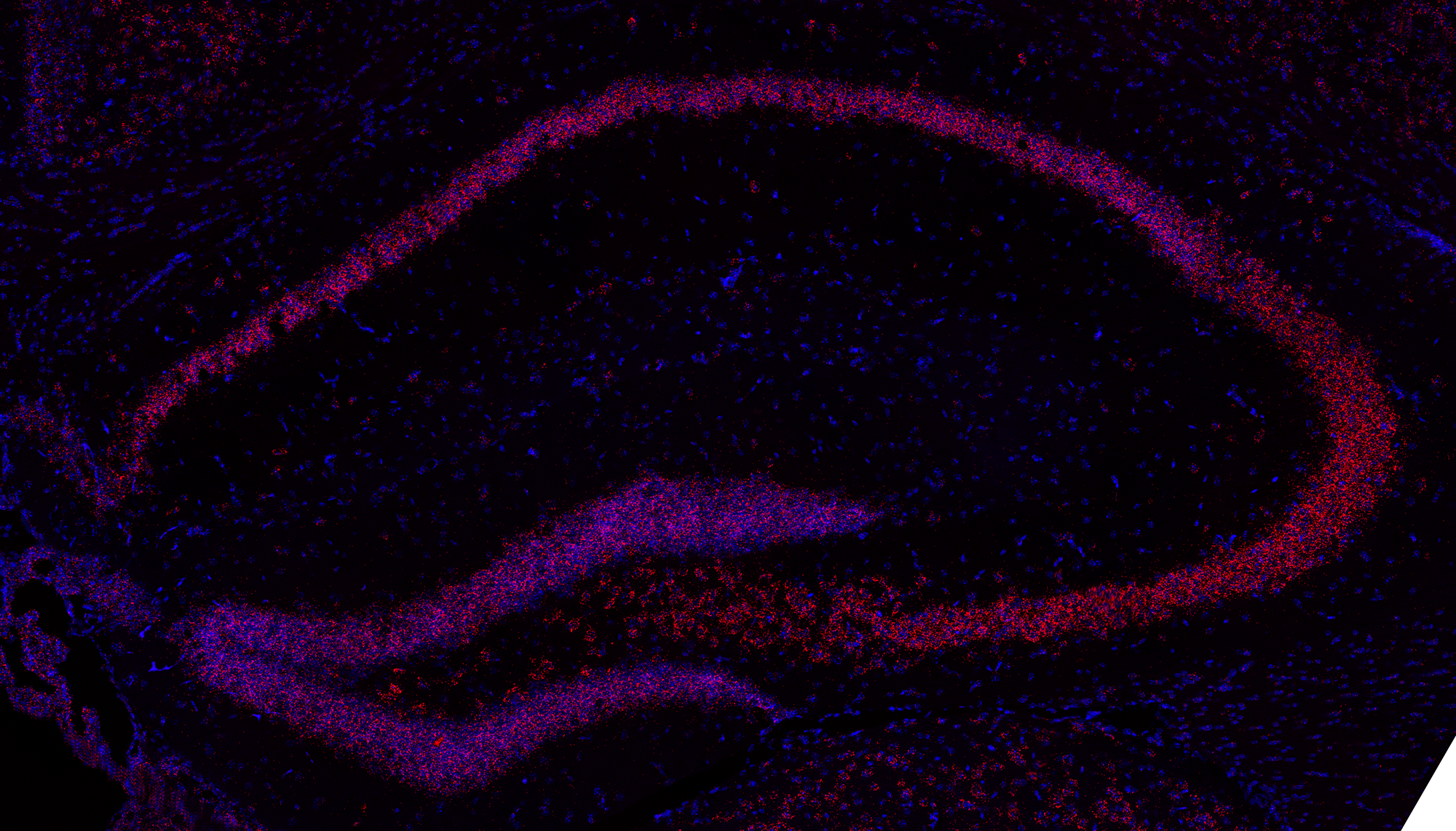

Supplement: Supplementary file 3 — Source data Fig. 1 [file 44321_2024_54_MOESM3_ESM.zip › Figure 1/1B/GABA-CRBN-KO/GABA-CRBN-KO - HC 1.tif]

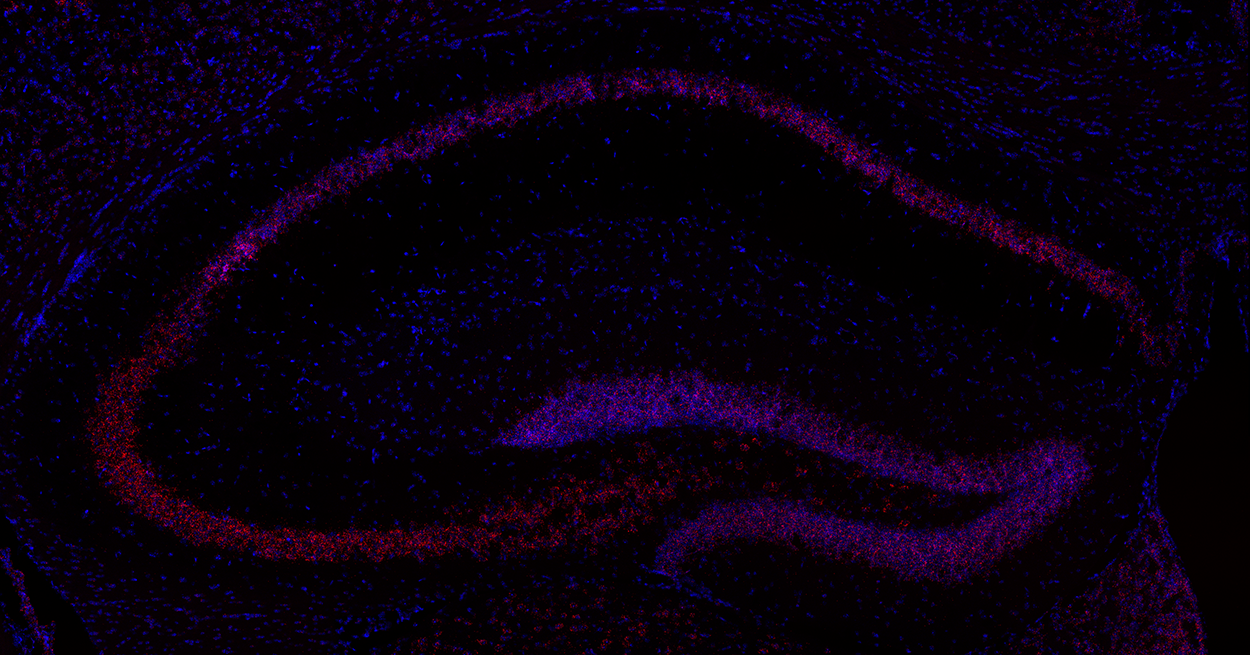

Supplement: Supplementary file 3 — Source data Fig. 1 [file 44321_2024_54_MOESM3_ESM.zip › Figure 1/1B/GABA-CRBN-KO/GABA-CRBN-KO - HC 2.tif]

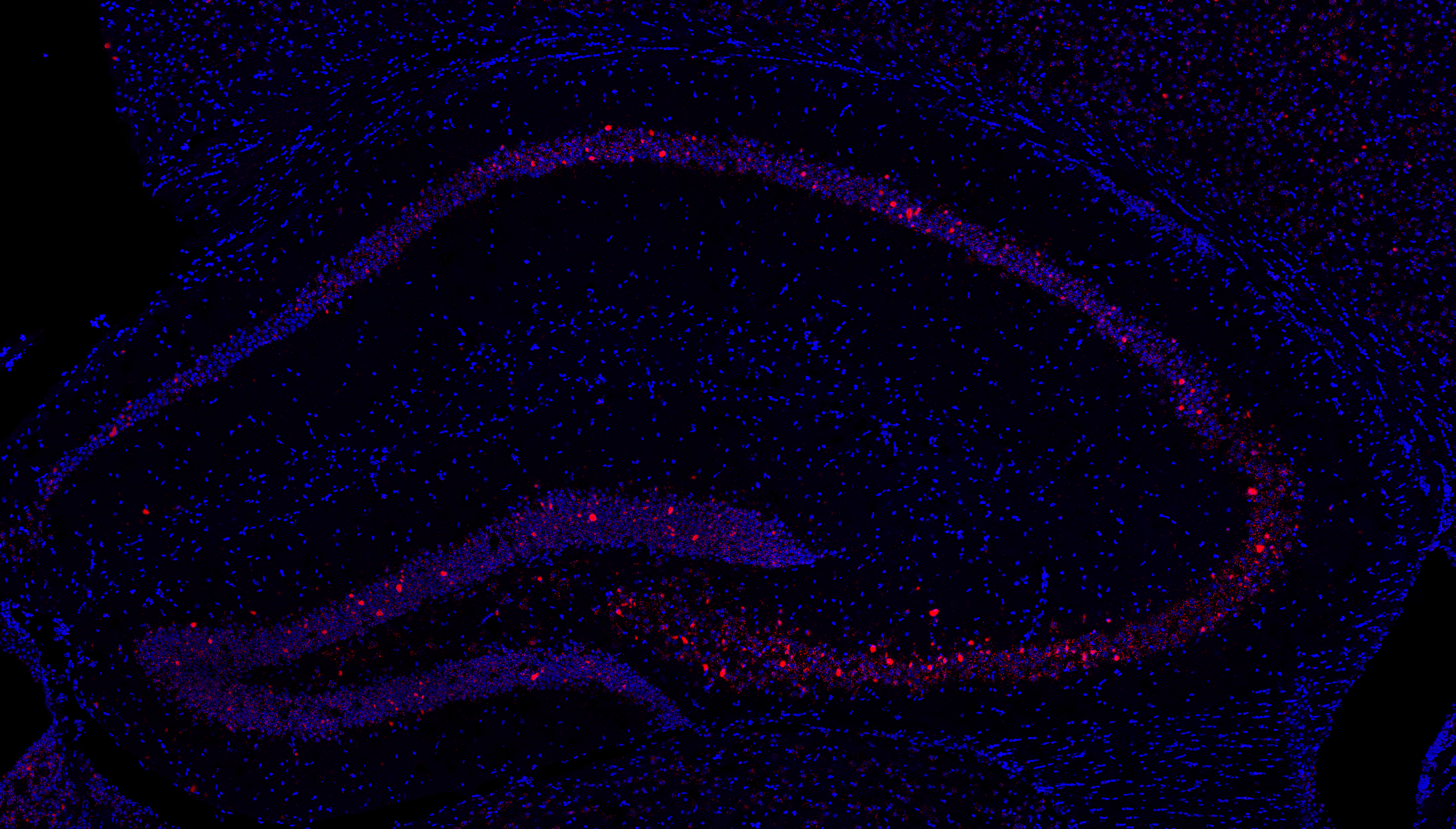

Supplement: Supplementary file 3 — Source data Fig. 1 [file 44321_2024_54_MOESM3_ESM.zip › Figure 1/1B/GABA-CRBN-KO/GABA-CRBN-KO - HC 3.tif]

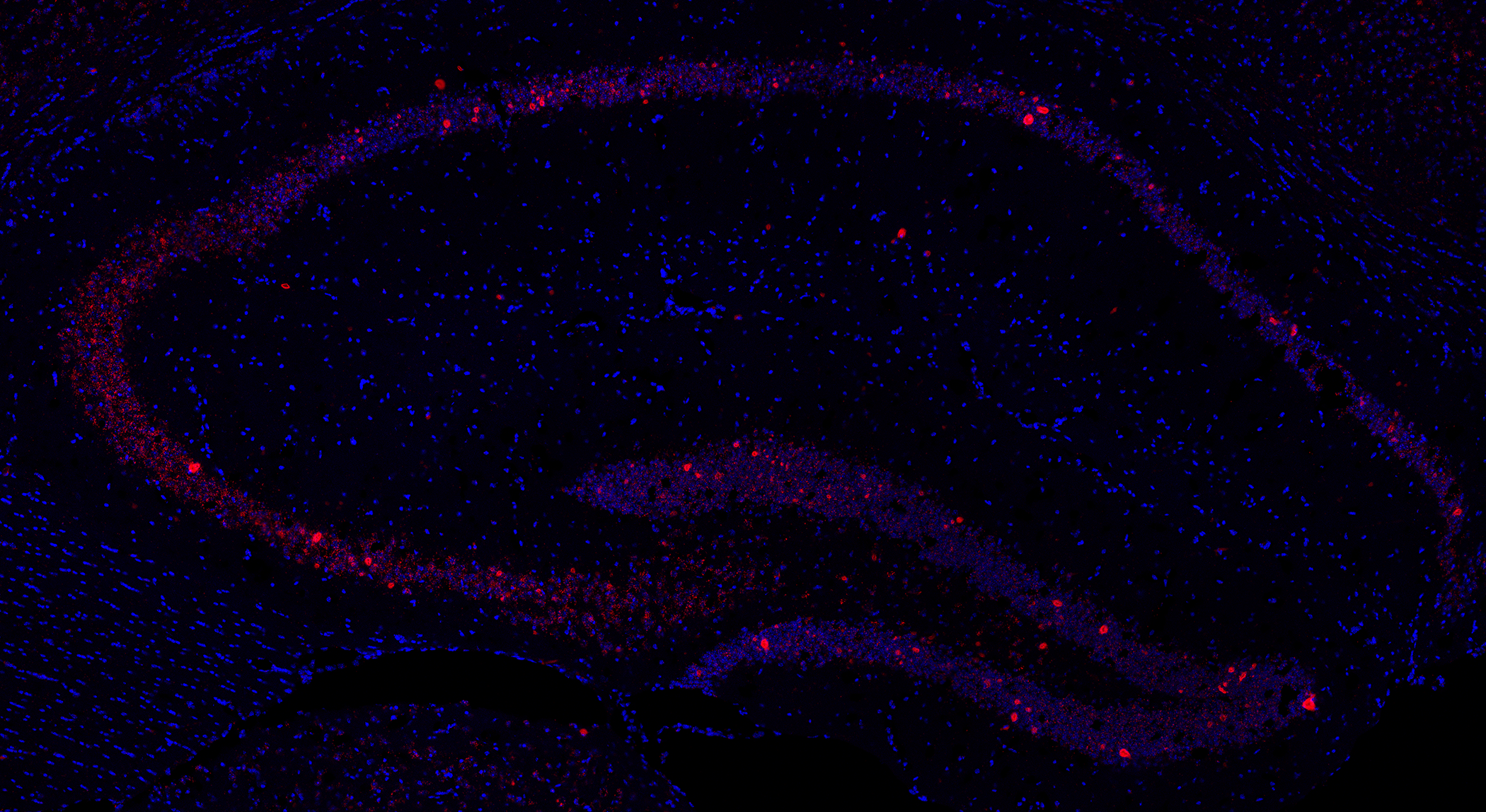

Supplement: Supplementary file 3 — Source data Fig. 1 [file 44321_2024_54_MOESM3_ESM.zip › Figure 1/1B/GABA-CRBN-KO/GABA-CRBN-KO - HC 4.tif]

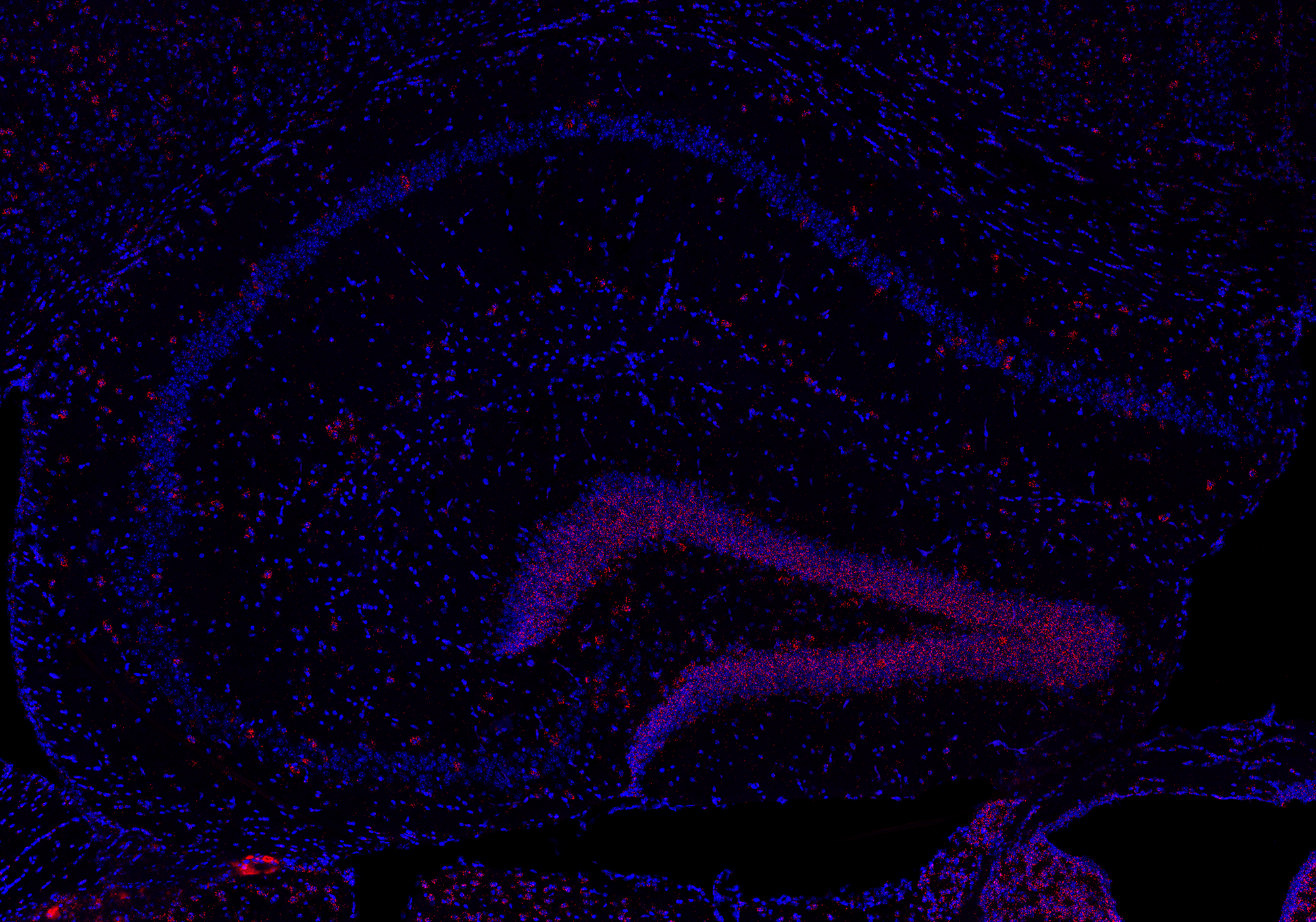

Supplement: Supplementary file 3 — Source data Fig. 1 [file 44321_2024_54_MOESM3_ESM.zip › Figure 1/1B/Glu-CRBN-KO/Fig 1. Glu-CRBN-KO - HC 3.tif]

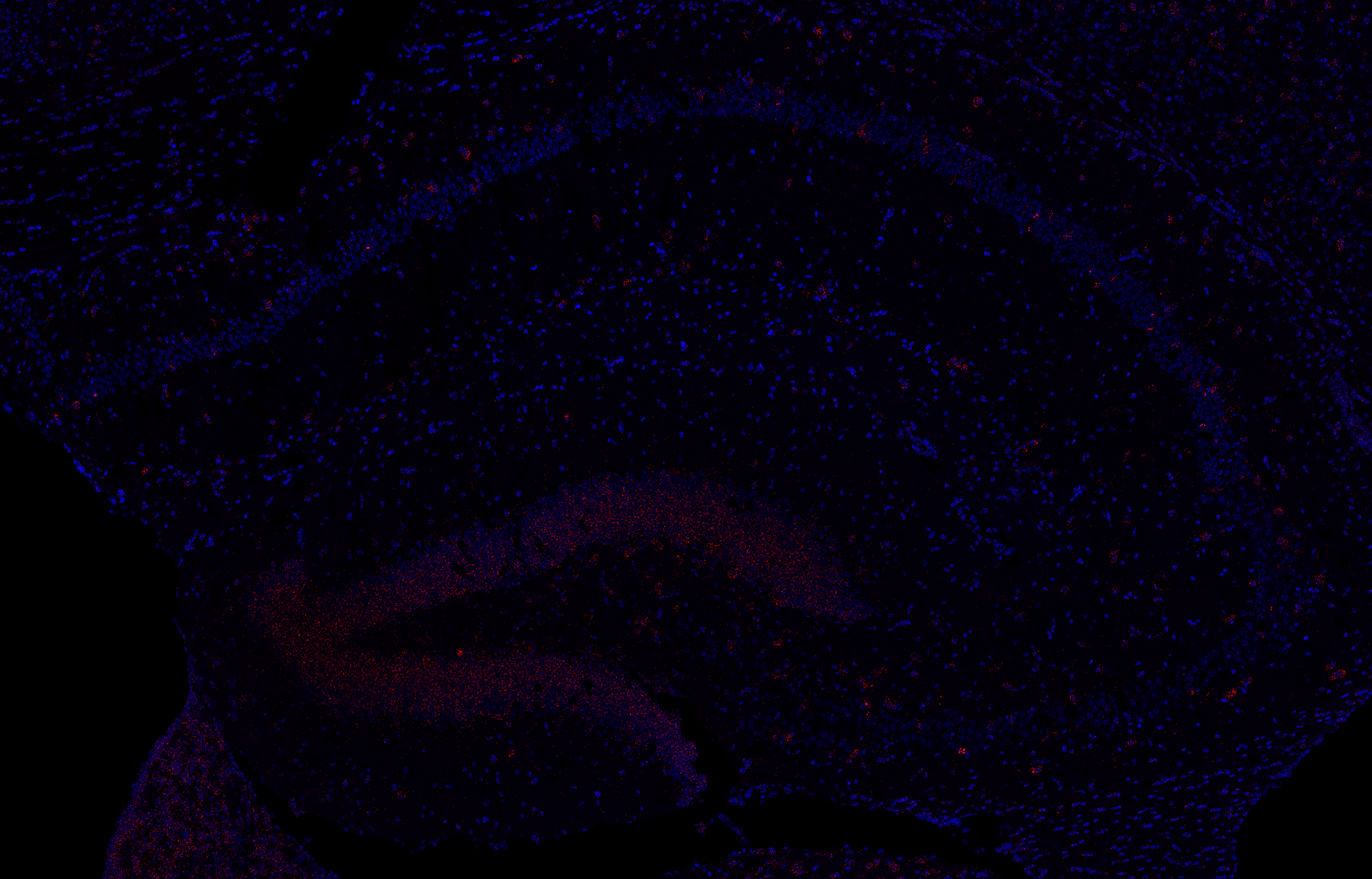

Supplement: Supplementary file 3 — Source data Fig. 1 [file 44321_2024_54_MOESM3_ESM.zip › Figure 1/1B/Glu-CRBN-KO/Glu-CRBN-KO - HC 1.tif]

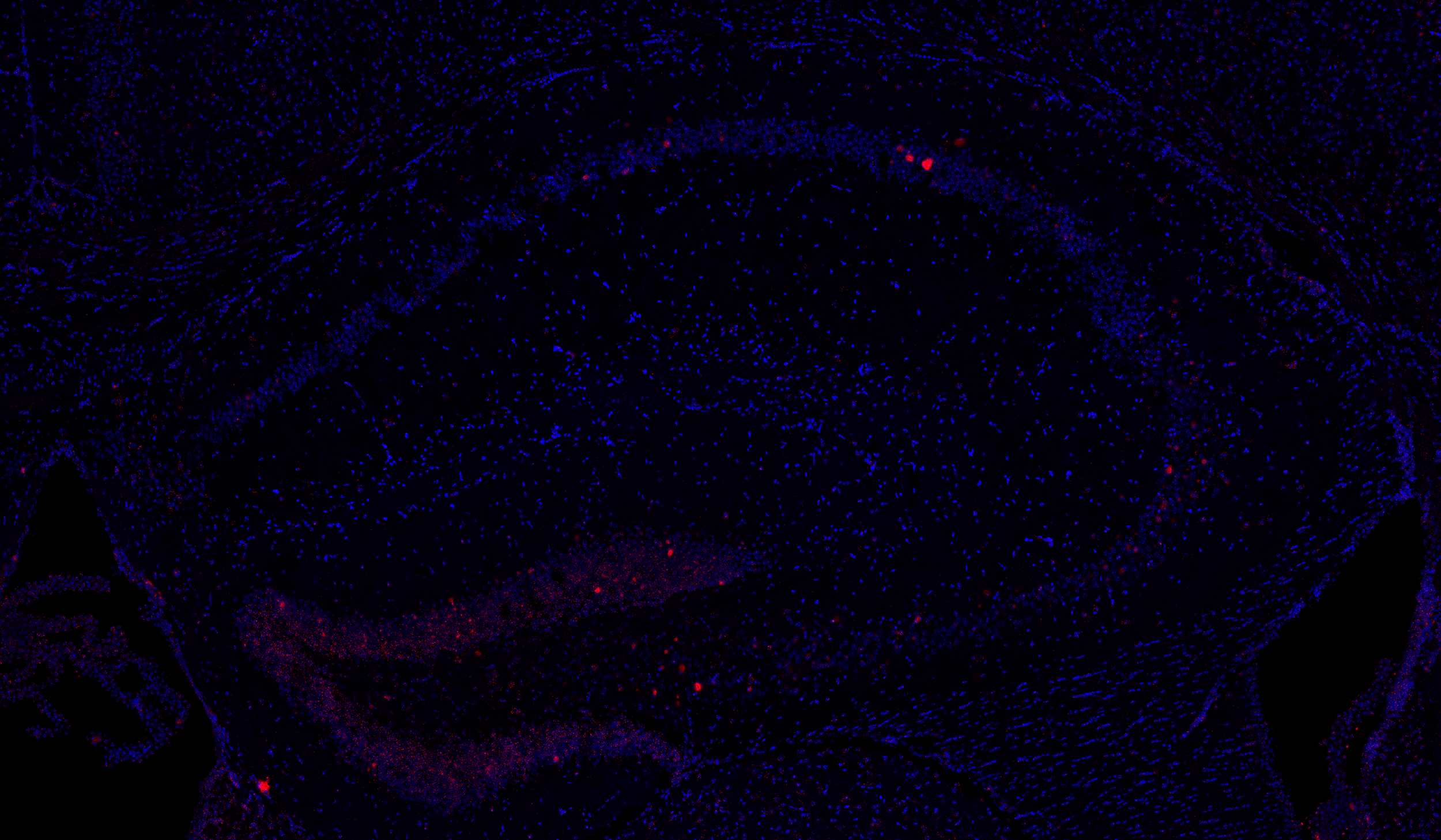

Supplement: Supplementary file 3 — Source data Fig. 1 [file 44321_2024_54_MOESM3_ESM.zip › Figure 1/1B/Glu-CRBN-KO/Glu-CRBN-KO - HC 2.tif]

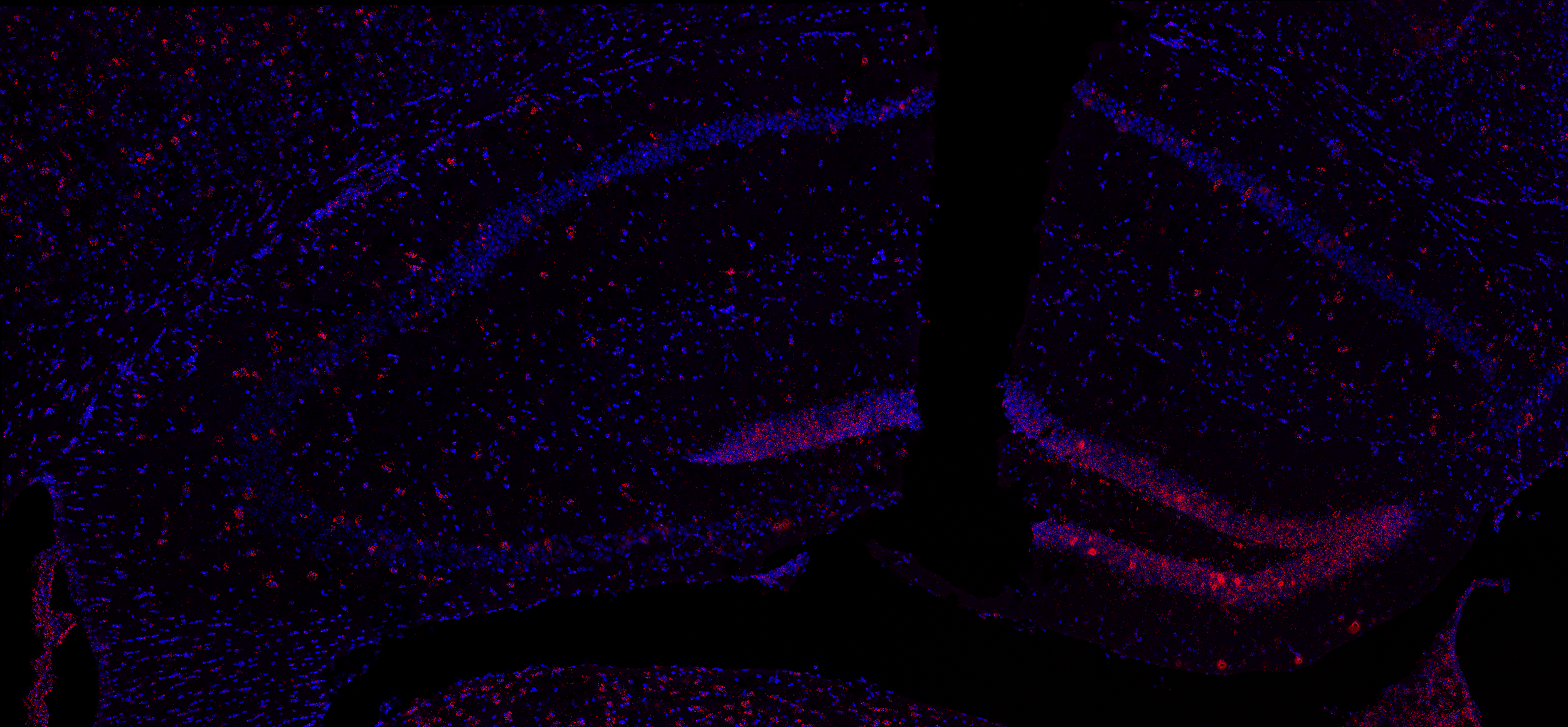

Supplement: Supplementary file 3 — Source data Fig. 1 [file 44321_2024_54_MOESM3_ESM.zip › Figure 1/1B/Glu-CRBN-KO/Glu-CRBN-KO - HC 4.tif]

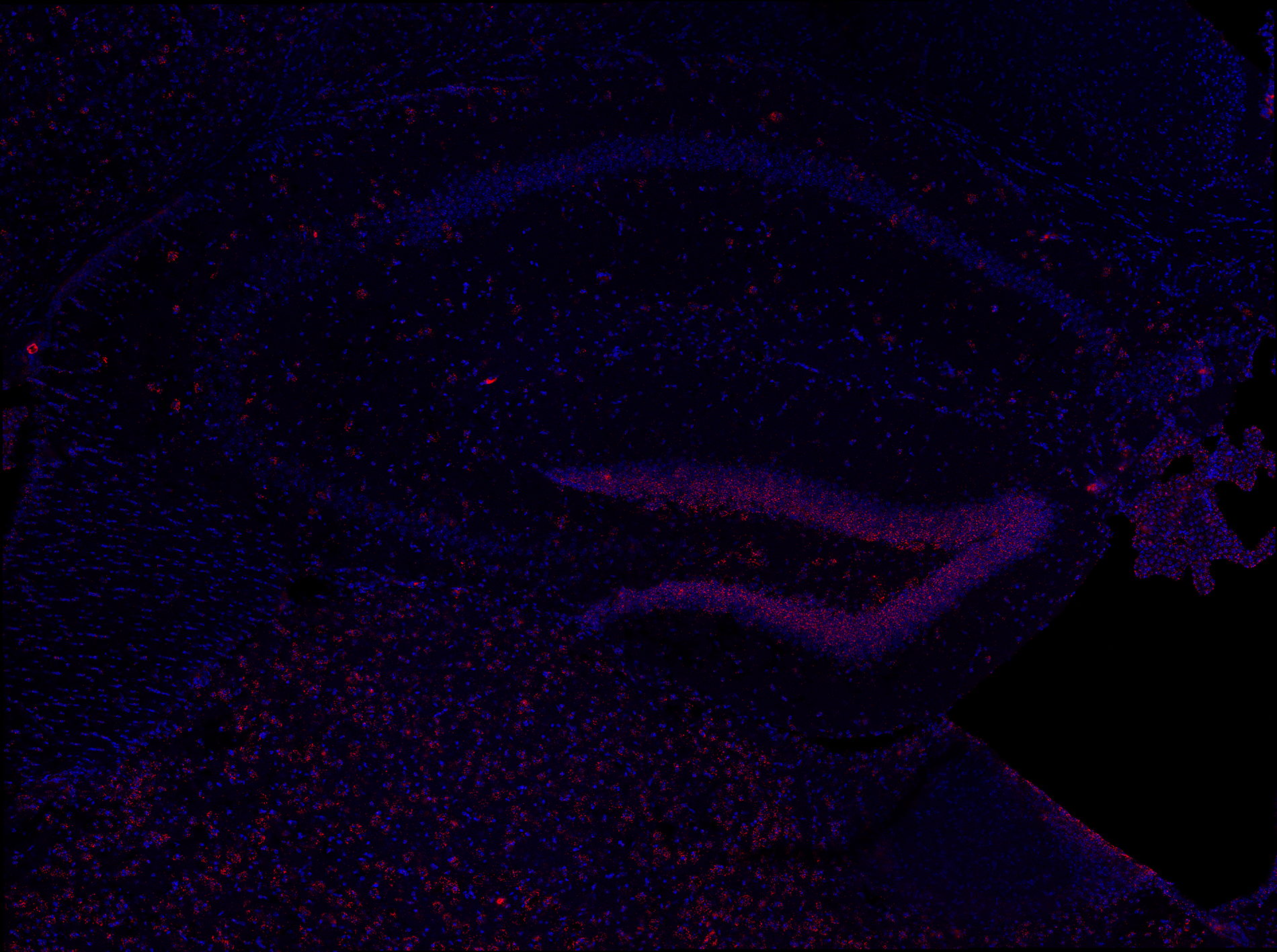

Supplement: Supplementary file 3 — Source data Fig. 1 [file 44321_2024_54_MOESM3_ESM.zip › Figure 1/1B/Glu-CRBN-KO/Glu-CRBN-KO - HC 5.tif]

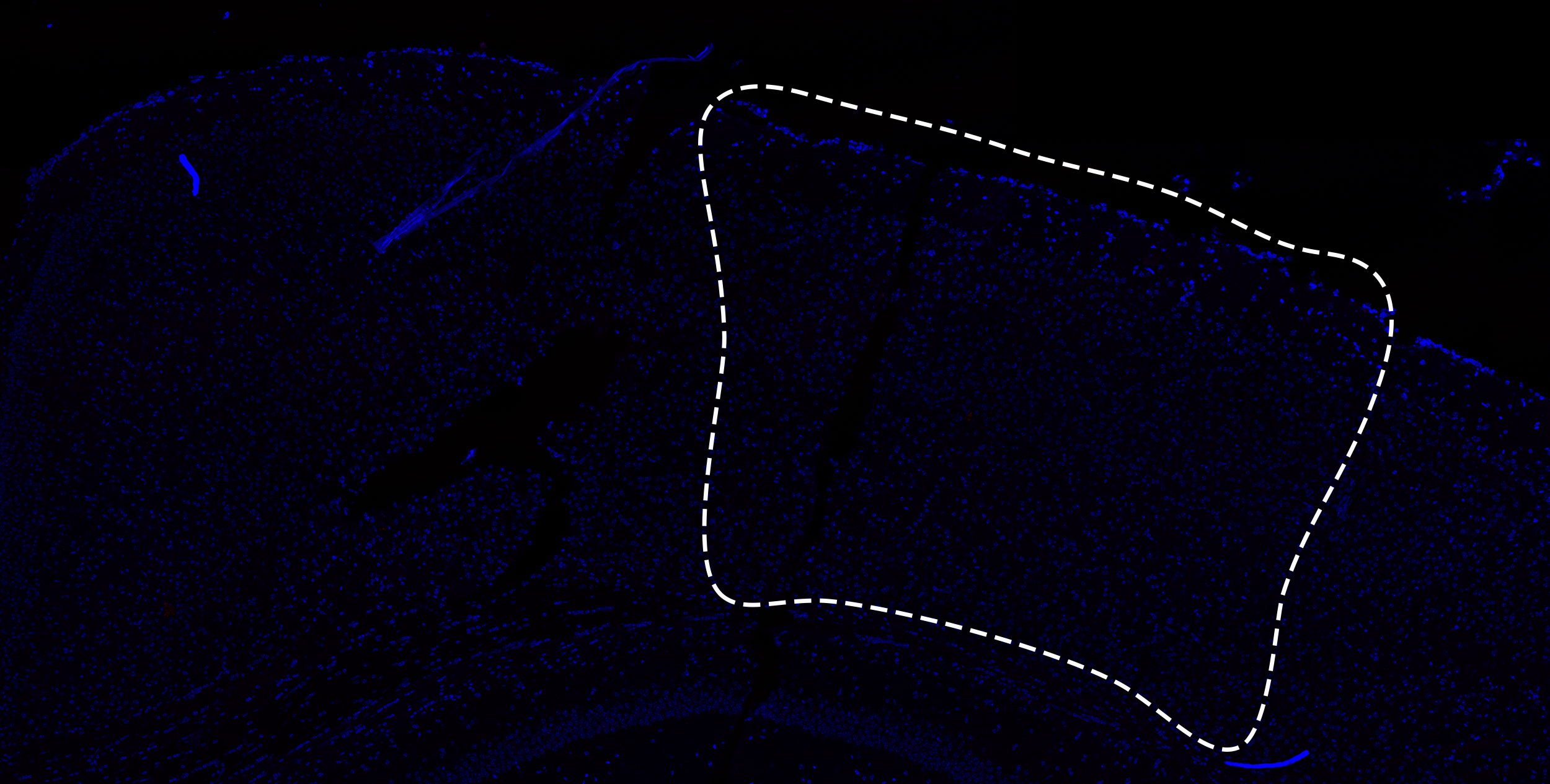

Supplement: Supplementary file 3 — Source data Fig. 1 [file 44321_2024_54_MOESM3_ESM.zip › Figure 1/1C/CRBN-KO/CRBN-KO - CX 1.tif]

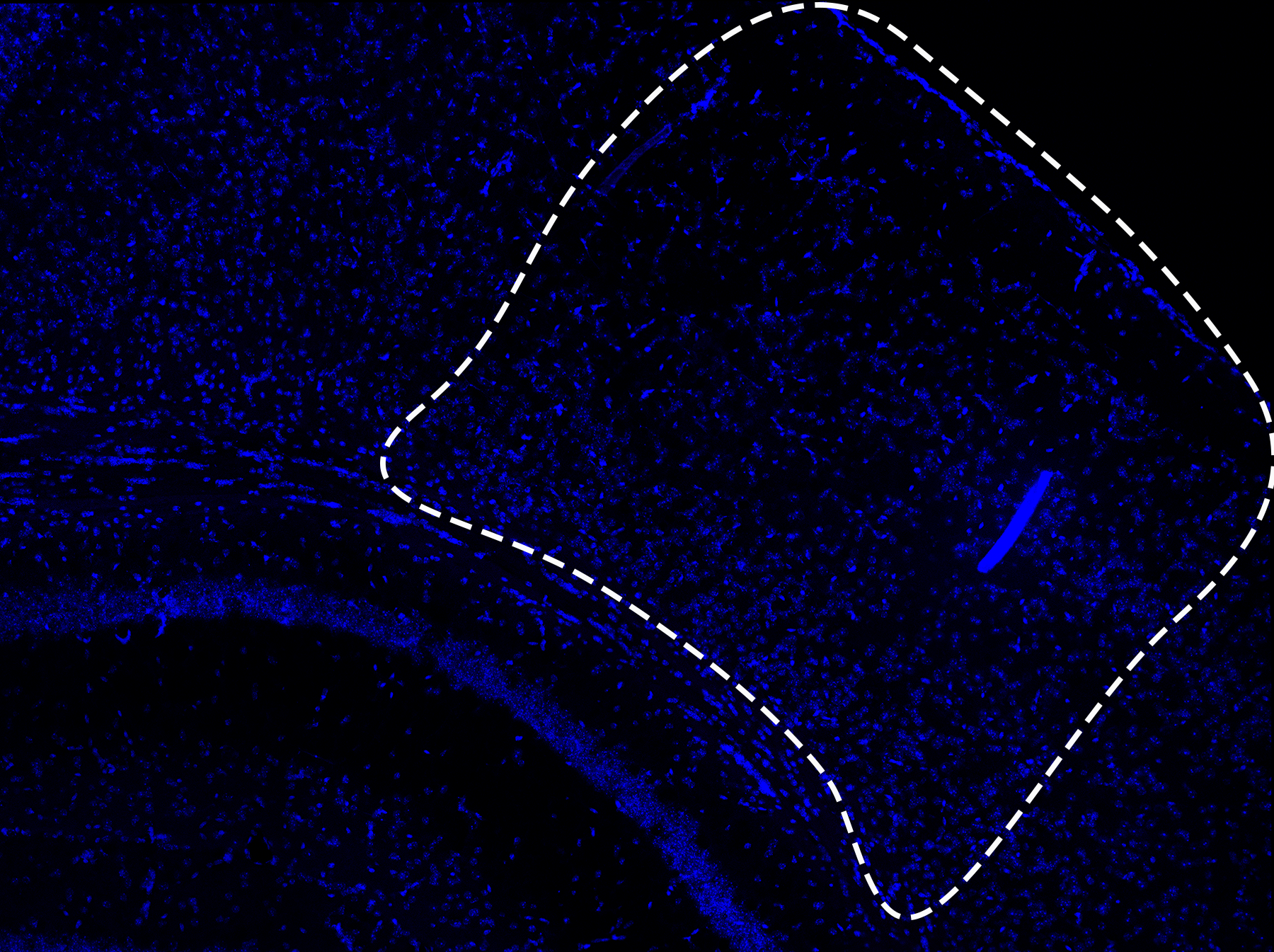

Supplement: Supplementary file 3 — Source data Fig. 1 [file 44321_2024_54_MOESM3_ESM.zip › Figure 1/1C/CRBN-KO/CRBN-KO - CX 2.tif]

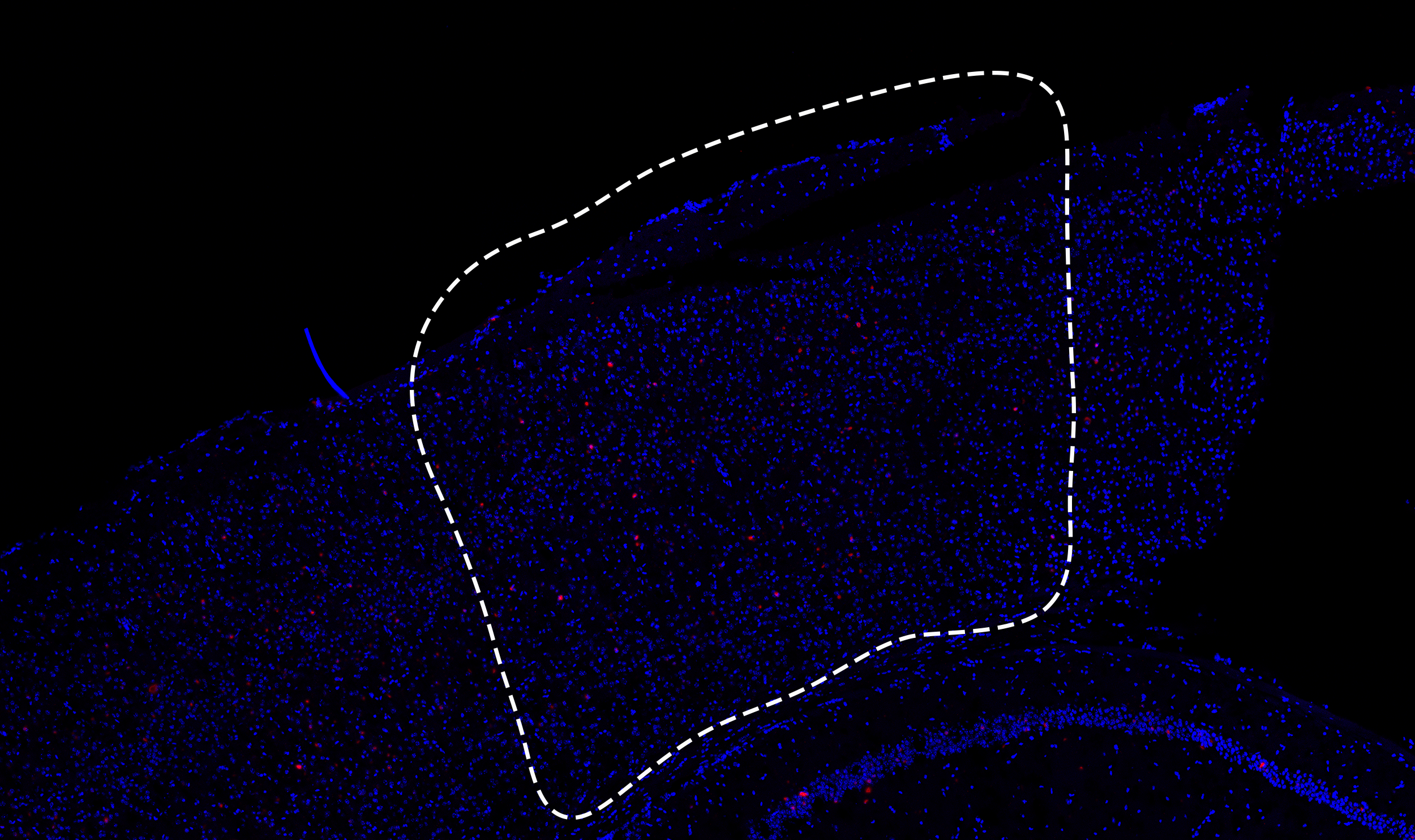

Supplement: Supplementary file 3 — Source data Fig. 1 [file 44321_2024_54_MOESM3_ESM.zip › Figure 1/1C/CRBN-KO/CRBN-KO - CX 3.tif]

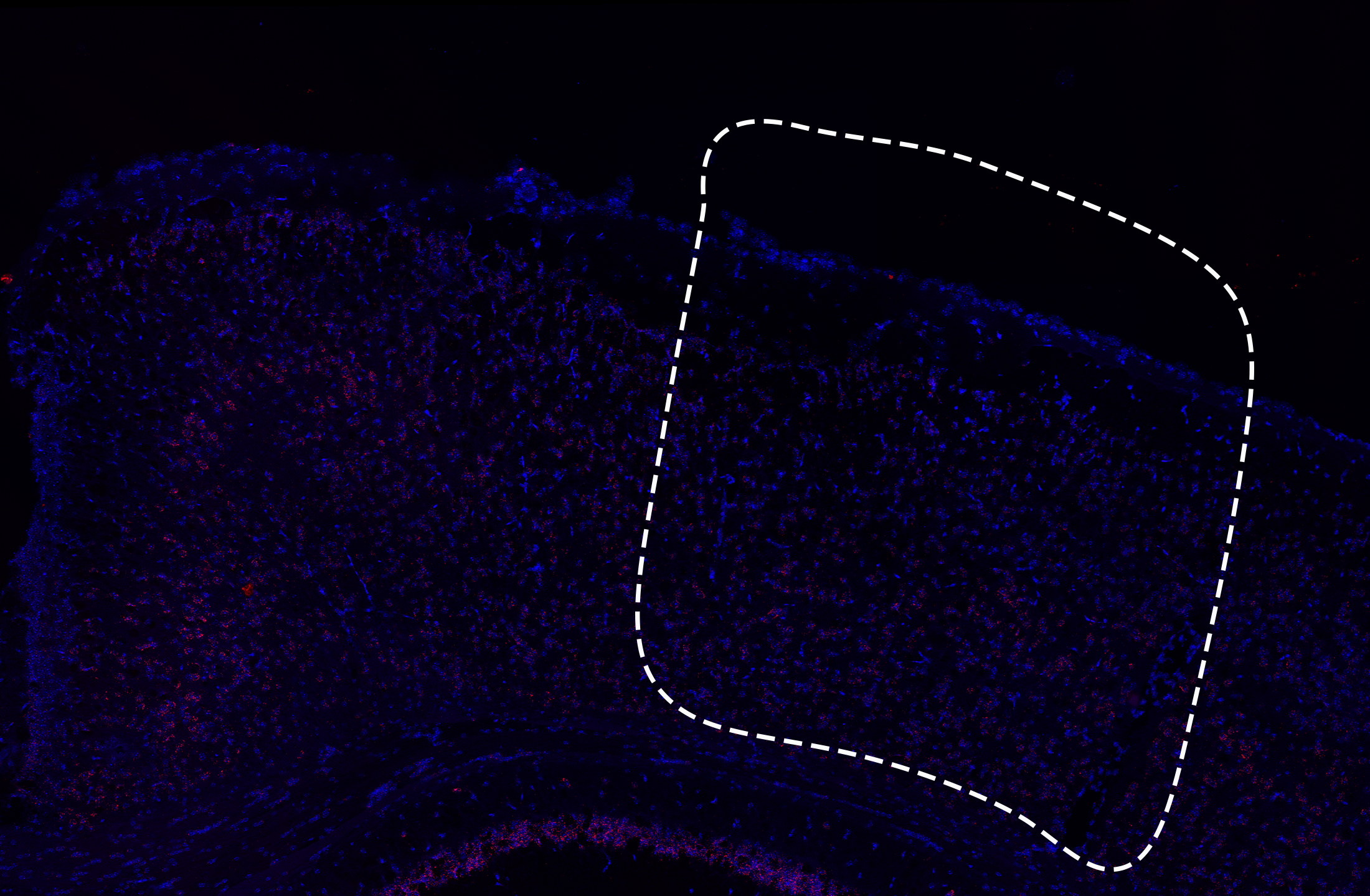

Supplement: Supplementary file 3 — Source data Fig. 1 [file 44321_2024_54_MOESM3_ESM.zip › Figure 1/1C/CRBN-WT/CRBN-WT - CX 1.tif]

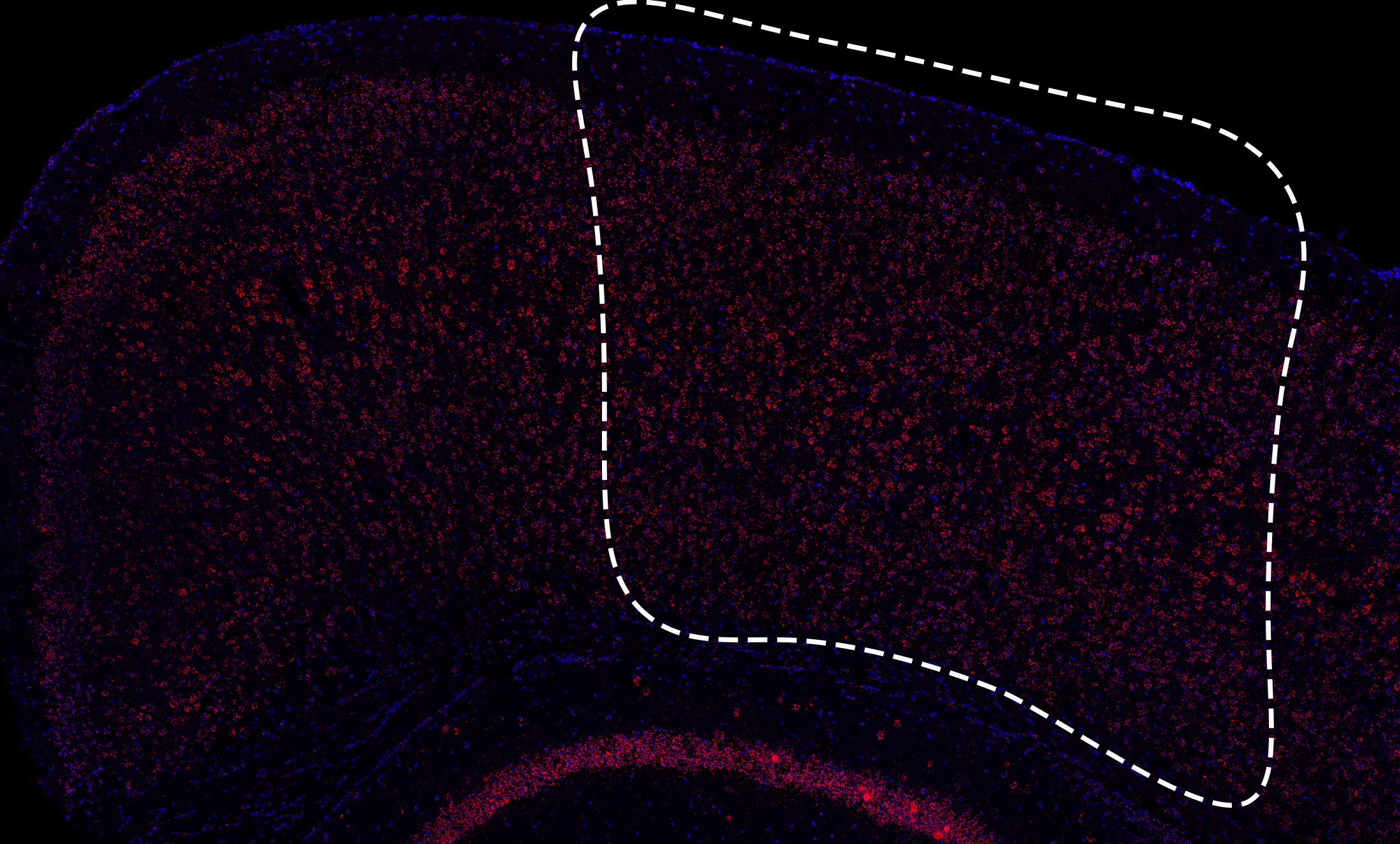

Supplement: Supplementary file 3 — Source data Fig. 1 [file 44321_2024_54_MOESM3_ESM.zip › Figure 1/1C/CRBN-WT/CRBN-WT - CX 2.tif]

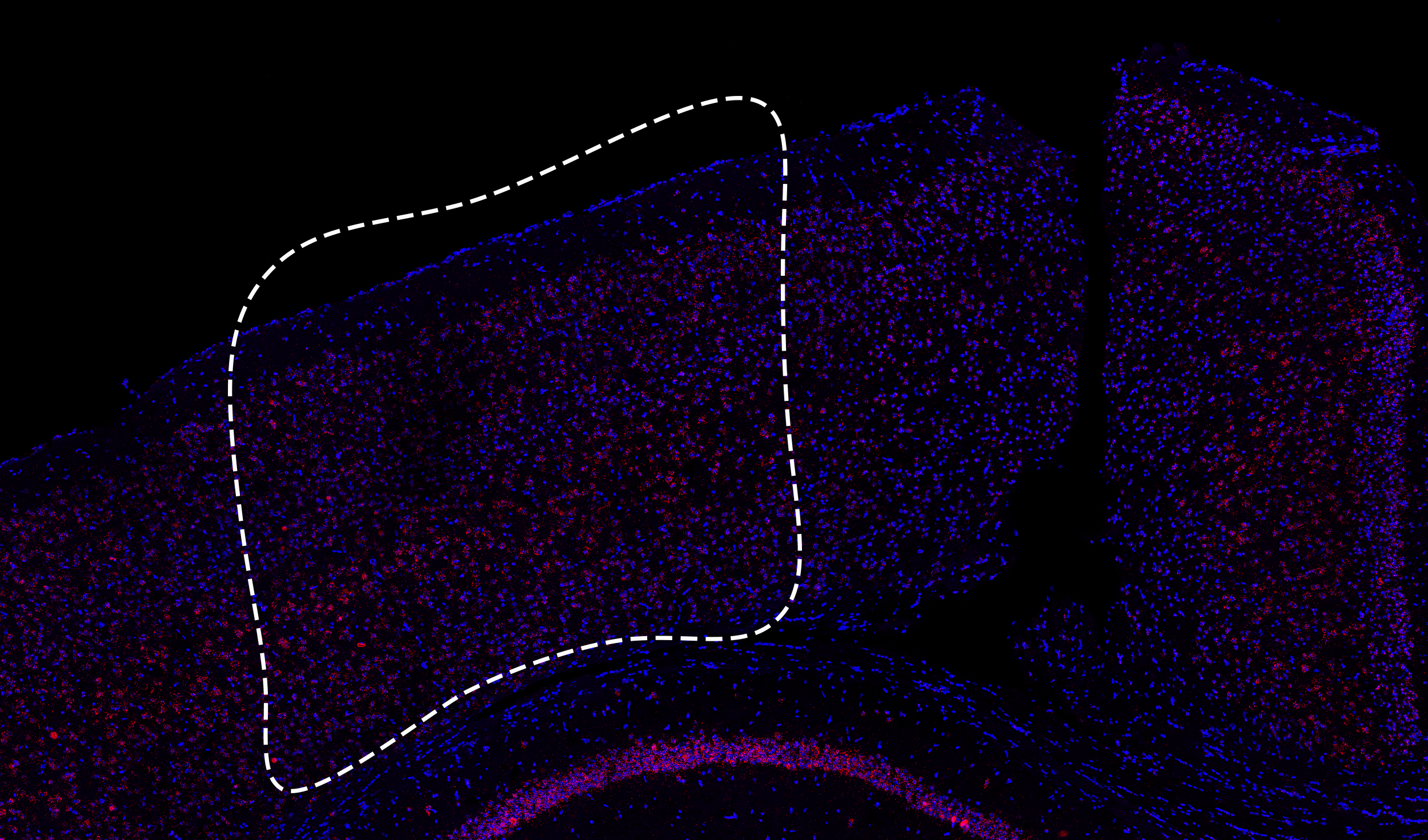

Supplement: Supplementary file 3 — Source data Fig. 1 [file 44321_2024_54_MOESM3_ESM.zip › Figure 1/1C/CRBN-WT/CRBN-WT - CX 3.tif]

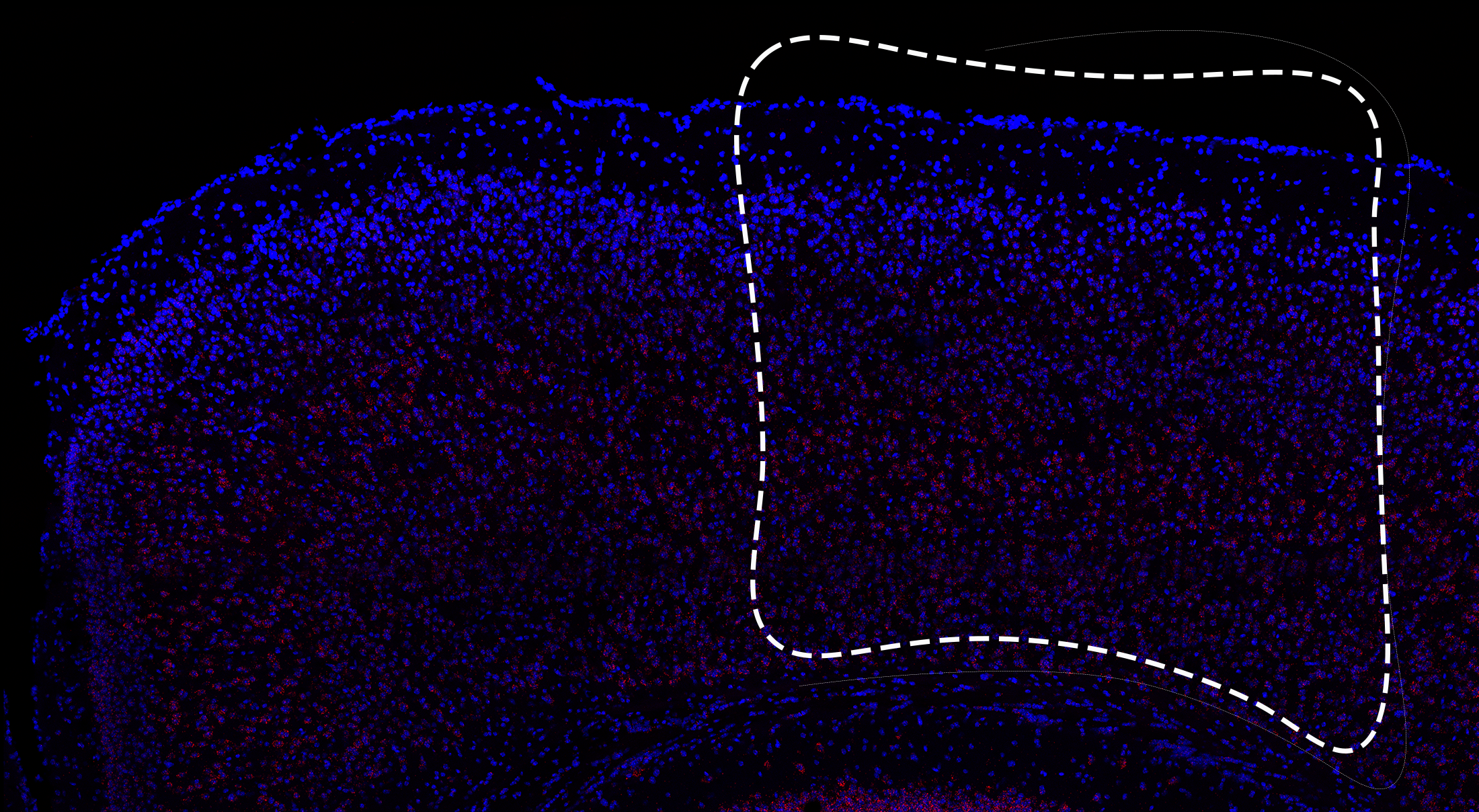

Supplement: Supplementary file 3 — Source data Fig. 1 [file 44321_2024_54_MOESM3_ESM.zip › Figure 1/1C/CRBN-WT/CRBN-WT - CX 4.tif]

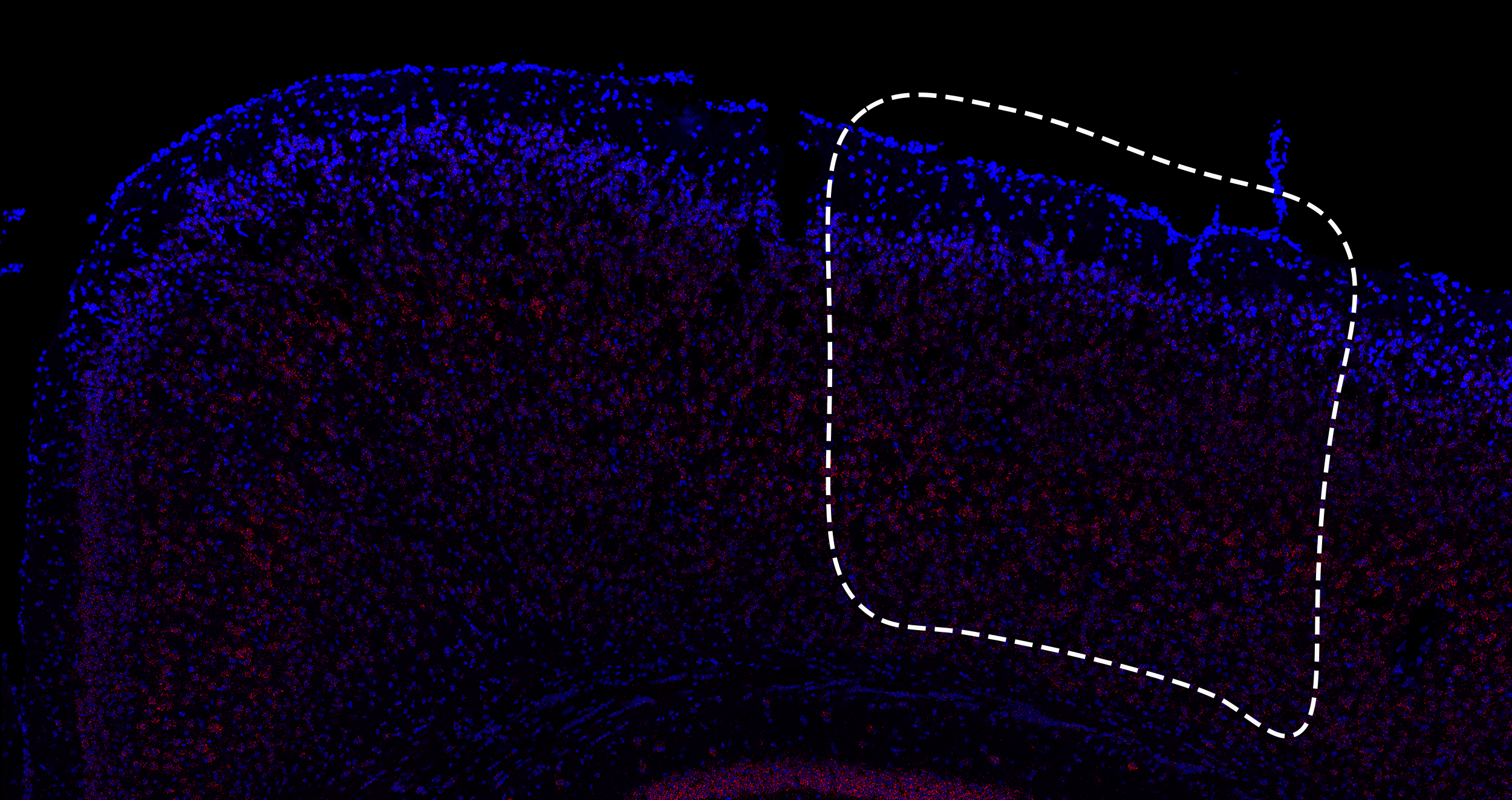

Supplement: Supplementary file 3 — Source data Fig. 1 [file 44321_2024_54_MOESM3_ESM.zip › Figure 1/1C/CRBN-WT/CRBN-WT - CX 5.tif]

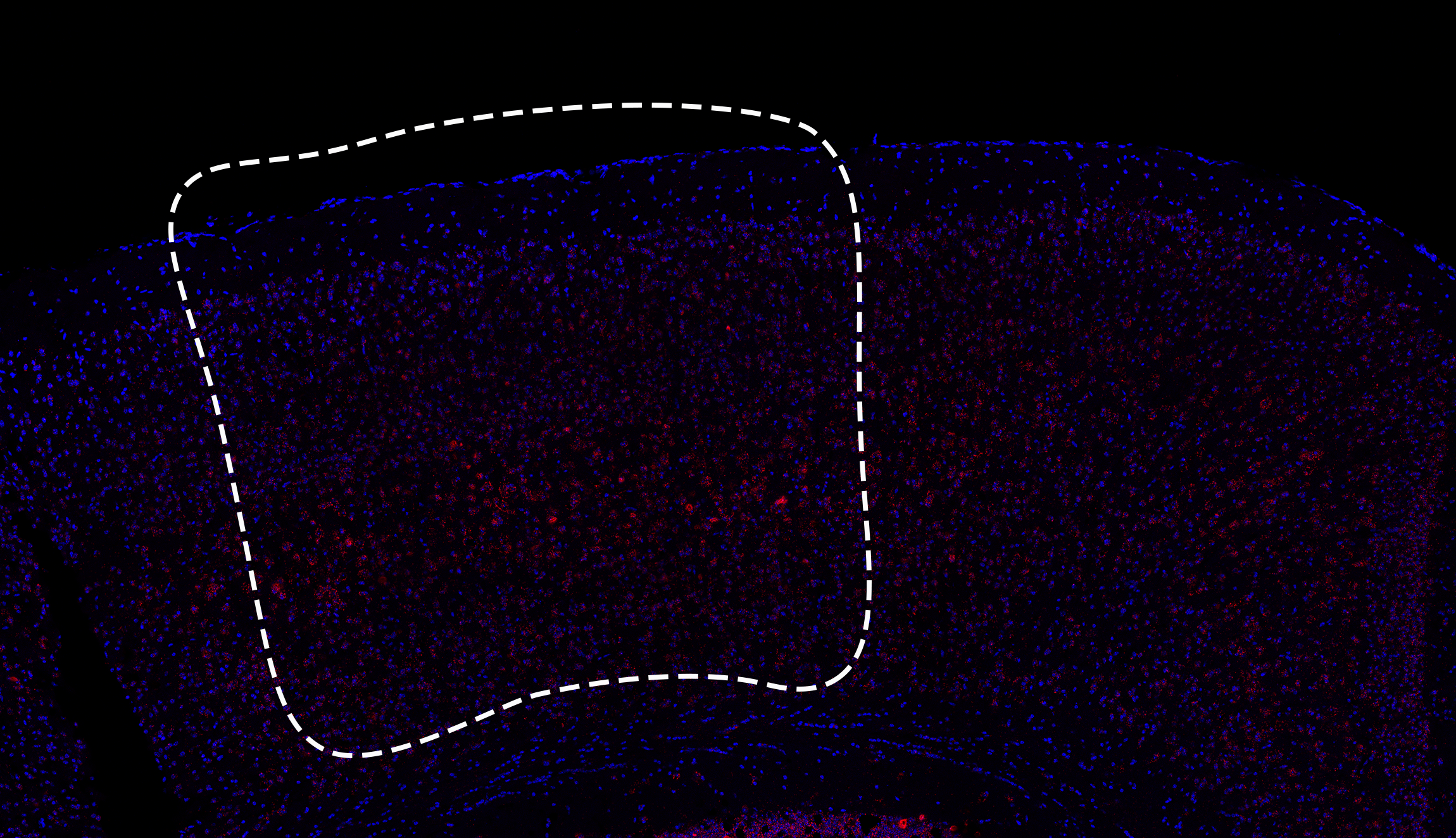

Supplement: Supplementary file 3 — Source data Fig. 1 [file 44321_2024_54_MOESM3_ESM.zip › Figure 1/1C/CRBN-WT/CRBN-WT - CX 6.tif]

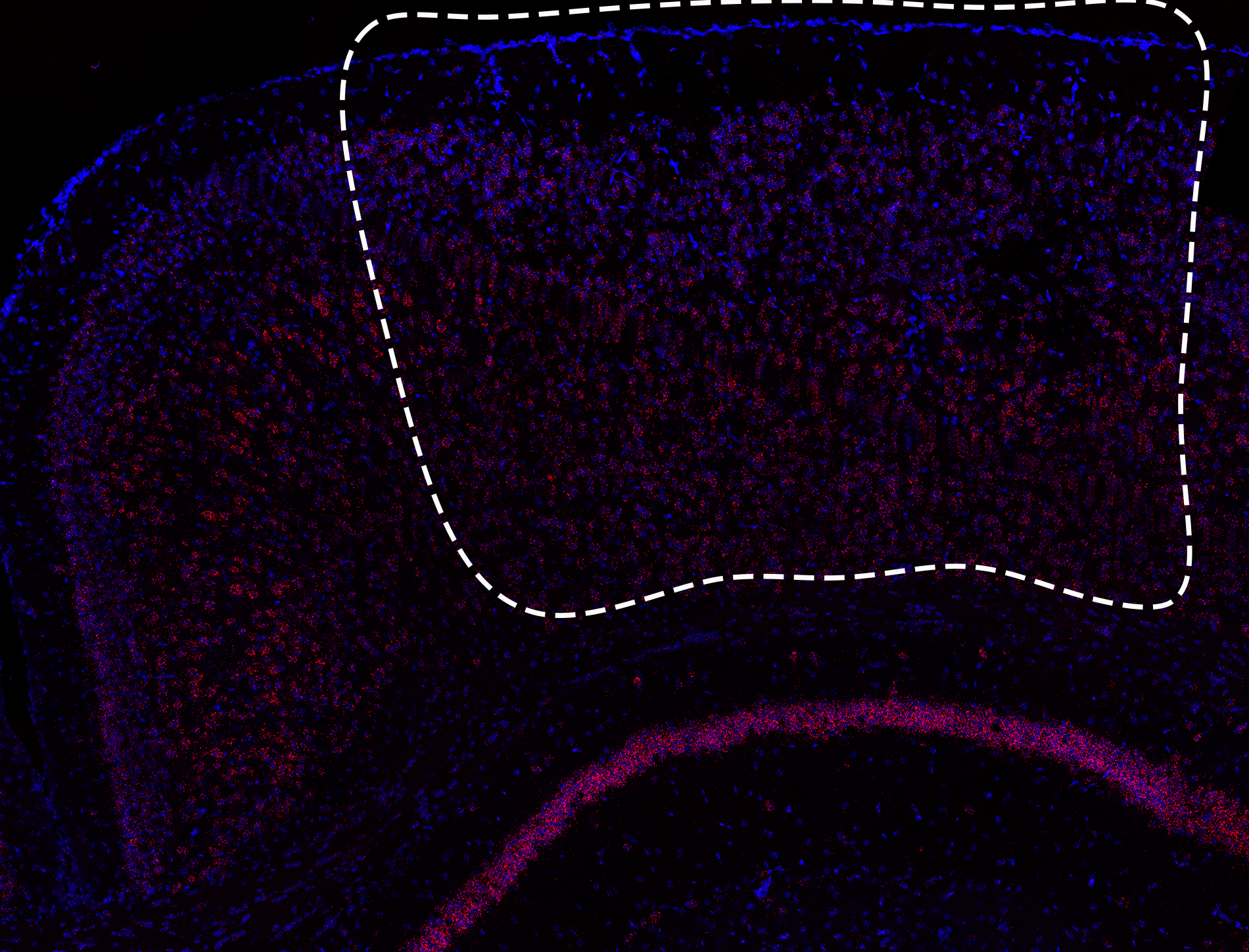

Supplement: Supplementary file 3 — Source data Fig. 1 [file 44321_2024_54_MOESM3_ESM.zip › Figure 1/1C/GABA-CRBN-KO/GABA-CRBN-KO - CX 1.tif]

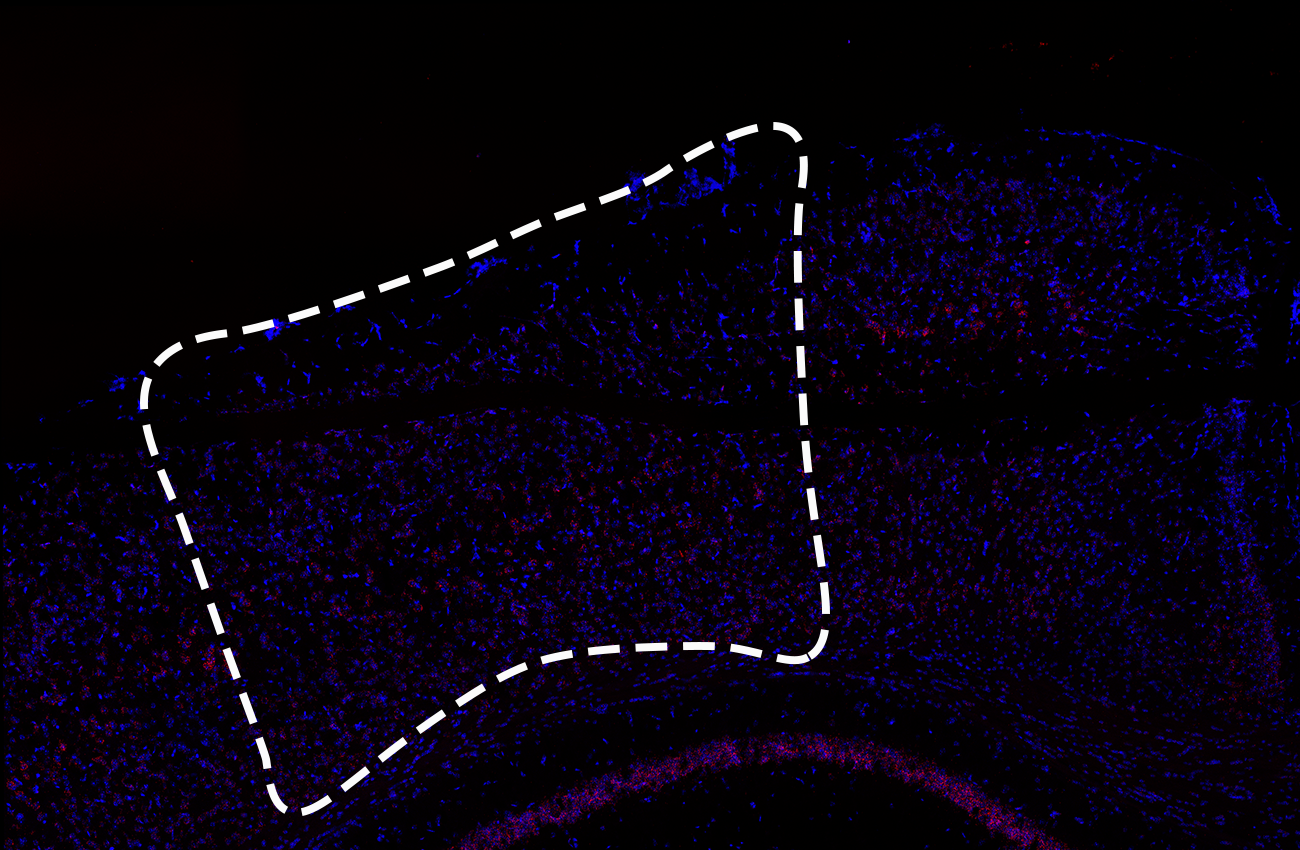

Supplement: Supplementary file 3 — Source data Fig. 1 [file 44321_2024_54_MOESM3_ESM.zip › Figure 1/1C/GABA-CRBN-KO/GABA-CRBN-KO - CX 2.tif]

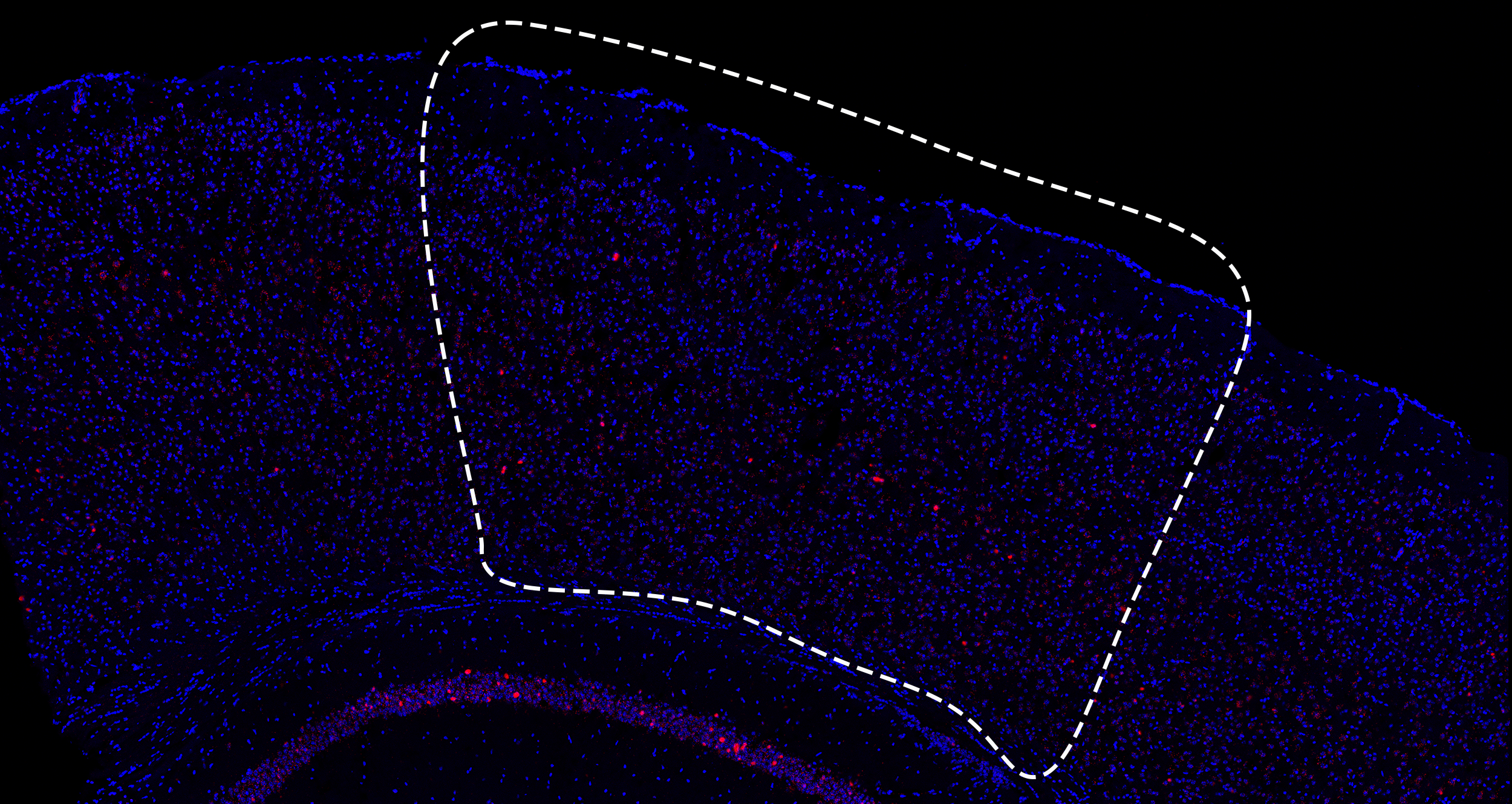

Supplement: Supplementary file 3 — Source data Fig. 1 [file 44321_2024_54_MOESM3_ESM.zip › Figure 1/1C/GABA-CRBN-KO/GABA-CRBN-KO - CX 3.tif]

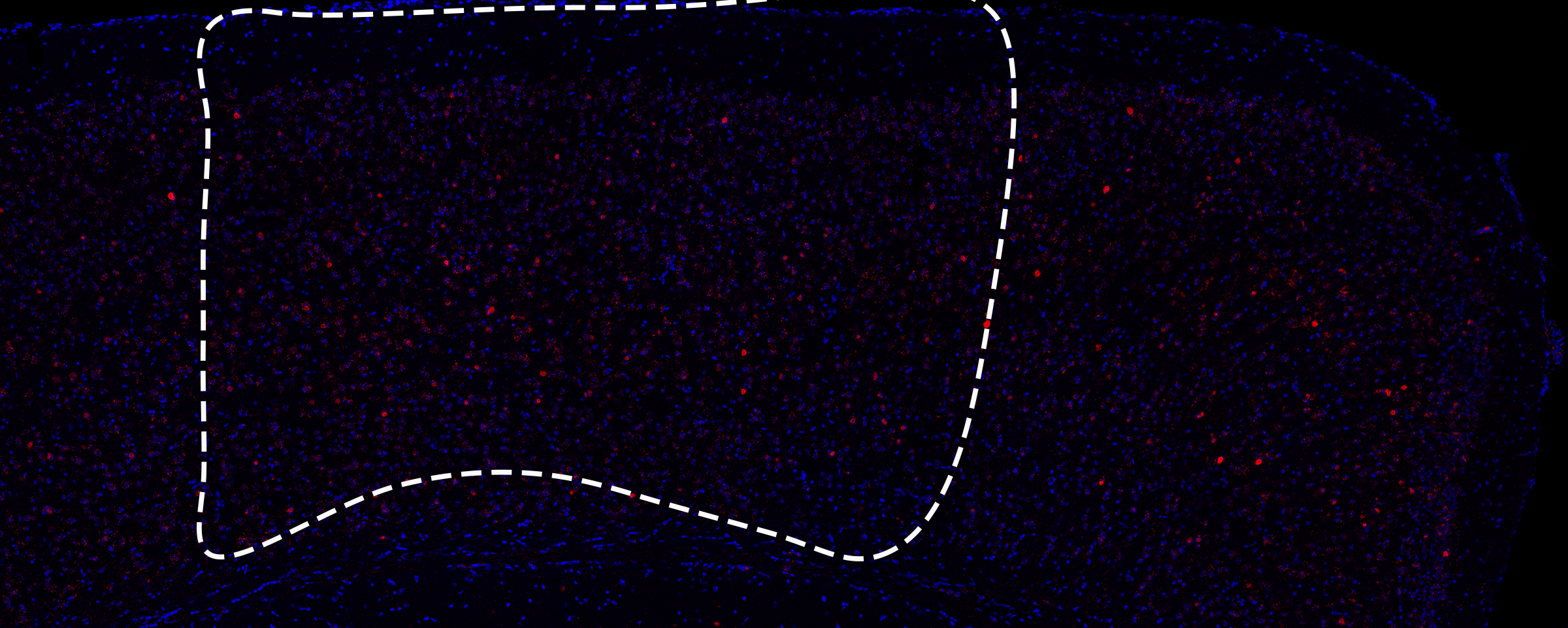

Supplement: Supplementary file 3 — Source data Fig. 1 [file 44321_2024_54_MOESM3_ESM.zip › Figure 1/1C/GABA-CRBN-KO/GABA-CRBN-KO - CX 4.tif]

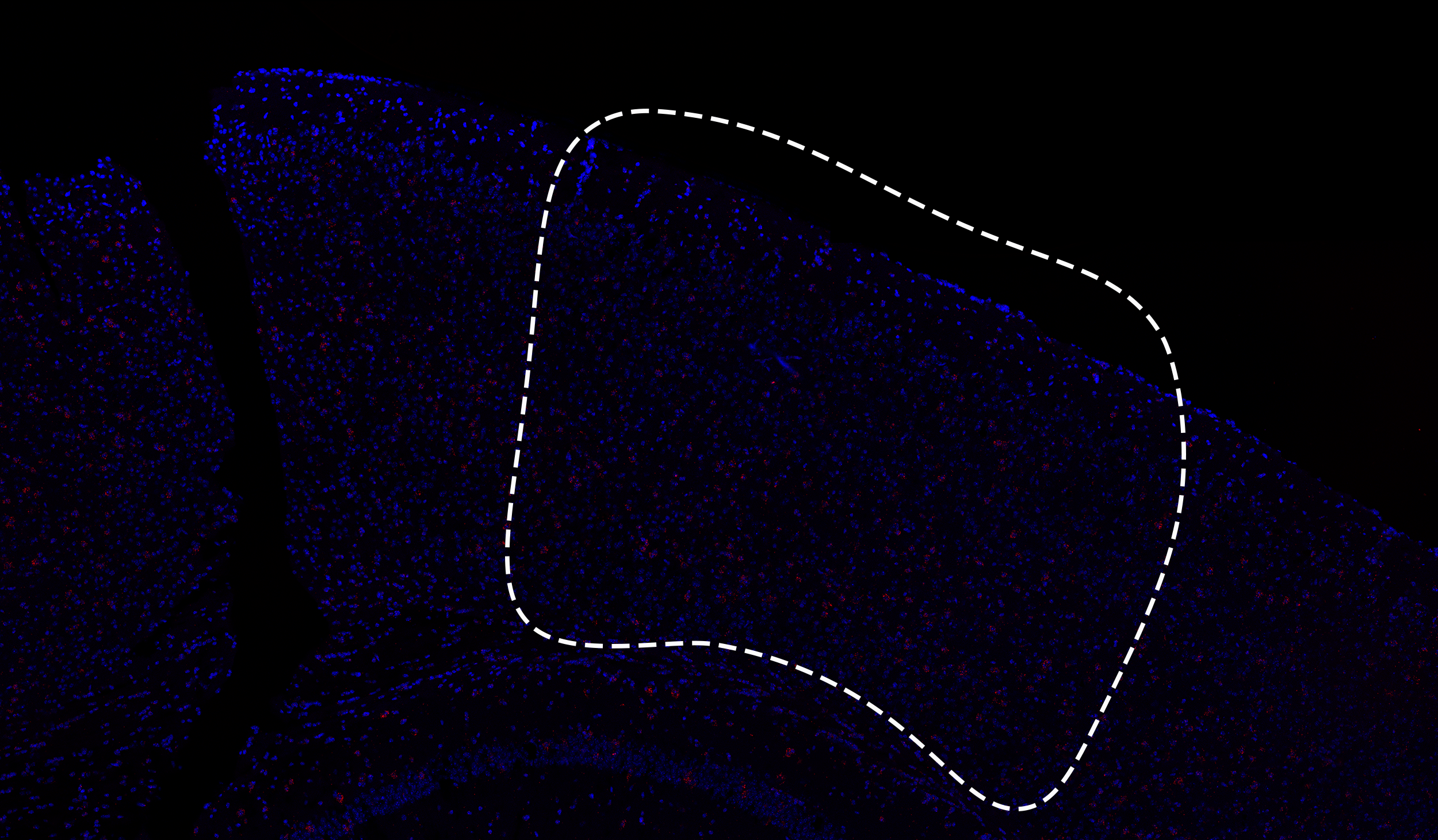

Supplement: Supplementary file 3 — Source data Fig. 1 [file 44321_2024_54_MOESM3_ESM.zip › Figure 1/1C/Glu-CRBN-KO/Glu-CRBN-KO - CX 1.tif]

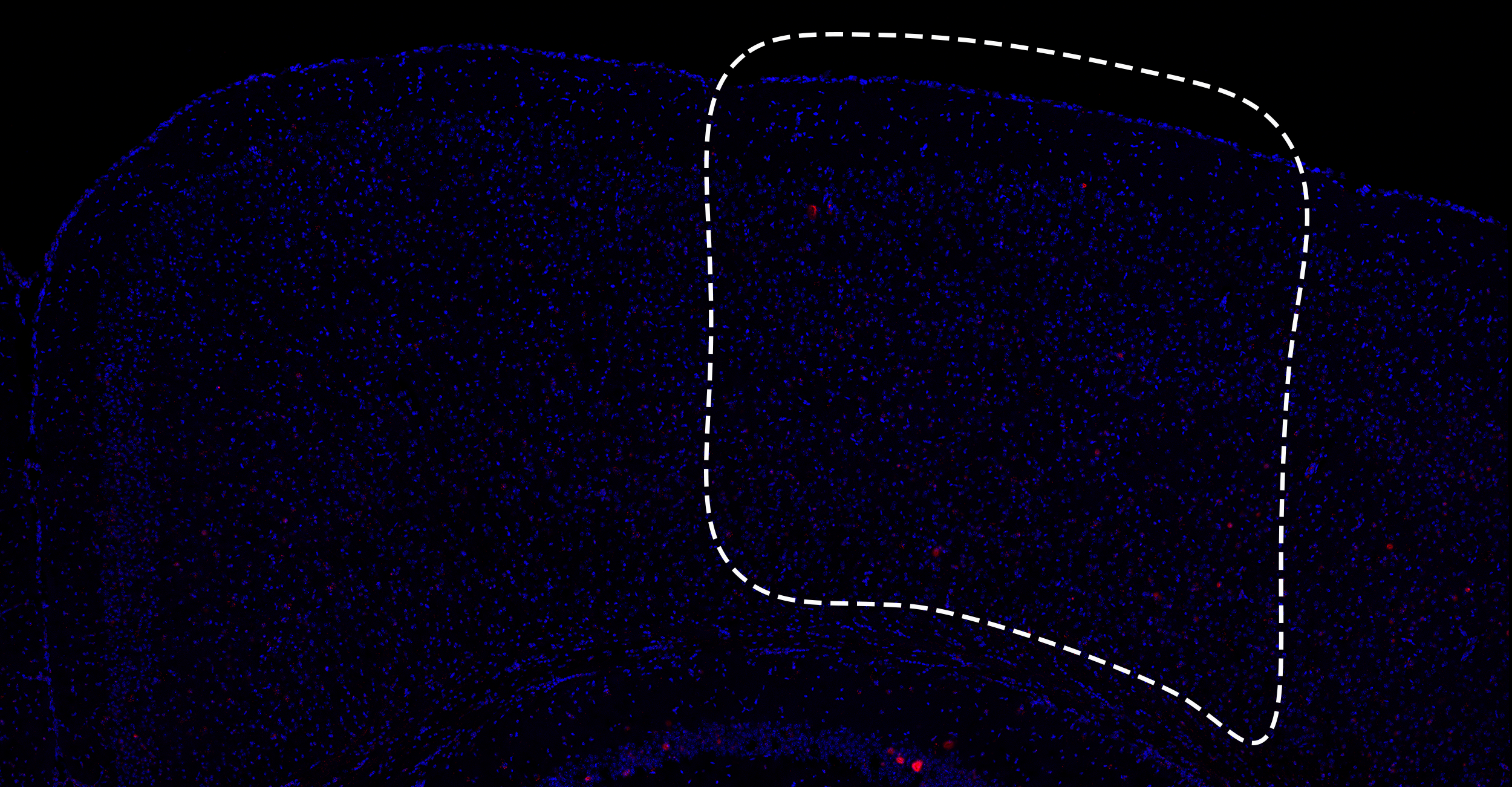

Supplement: Supplementary file 3 — Source data Fig. 1 [file 44321_2024_54_MOESM3_ESM.zip › Figure 1/1C/Glu-CRBN-KO/Glu-CRBN-KO - CX 2.tif]

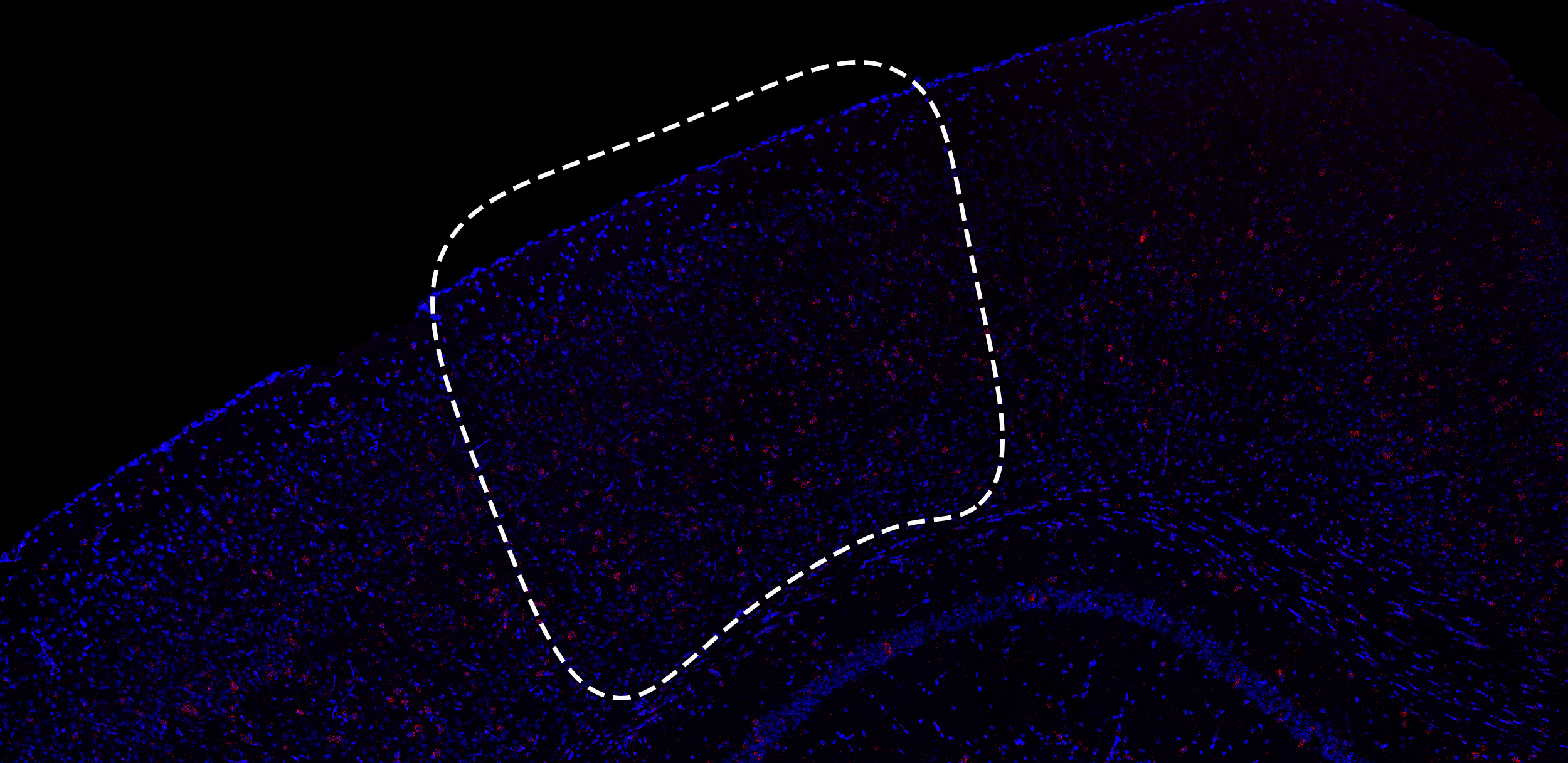

Supplement: Supplementary file 3 — Source data Fig. 1 [file 44321_2024_54_MOESM3_ESM.zip › Figure 1/1C/Glu-CRBN-KO/Glu-CRBN-KO - CX 3.tif]

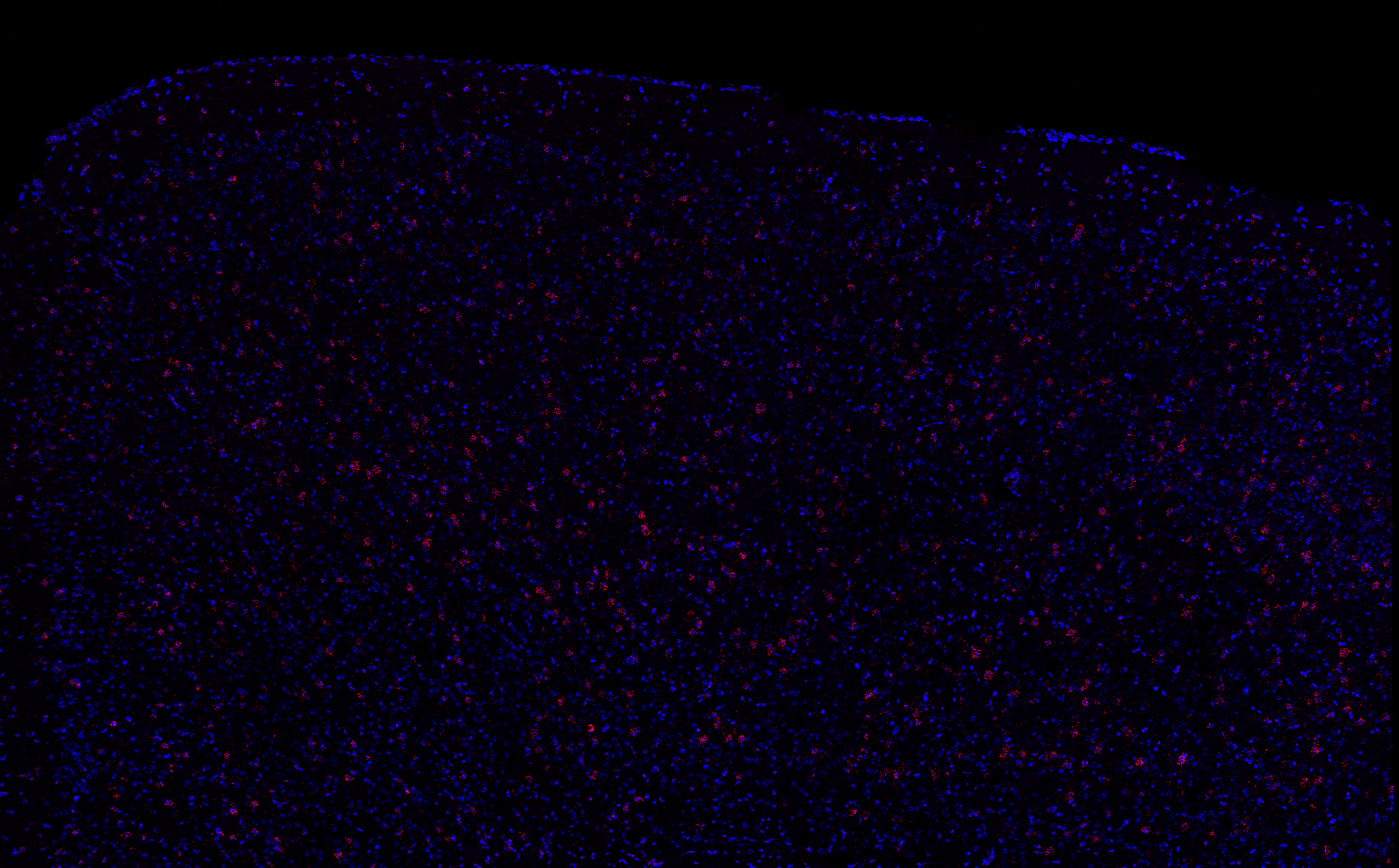

Supplement: Supplementary file 3 — Source data Fig. 1 [file 44321_2024_54_MOESM3_ESM.zip › Figure 1/1C/Glu-CRBN-KO/Glu-CRBN-KO - CX 4.tif]

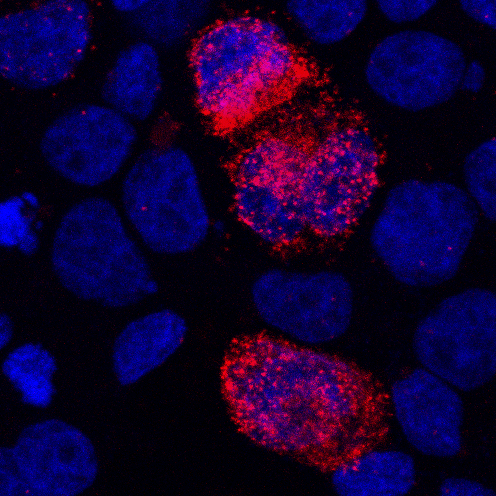

Supplement: Supplementary file 5 — Source data Fig. 3 [file 44321_2024_54_MOESM5_ESM.zip › Figure 3/Figure 3E/Fig 3E - CB1R-myc + HA-CRBN.tif]

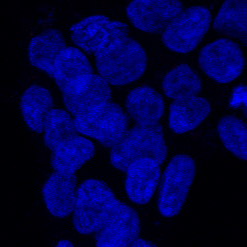

Supplement: Supplementary file 5 — Source data Fig. 3 [file 44321_2024_54_MOESM5_ESM.zip › Figure 3/Figure 3E/Fig 3E - CB1R-myc only.tif]

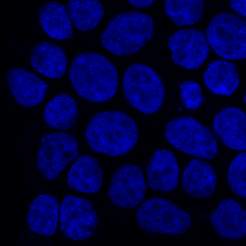

Supplement: Supplementary file 5 — Source data Fig. 3 [file 44321_2024_54_MOESM5_ESM.zip › Figure 3/Figure 3E/Fig 3E - HA-CRBN only.tif]

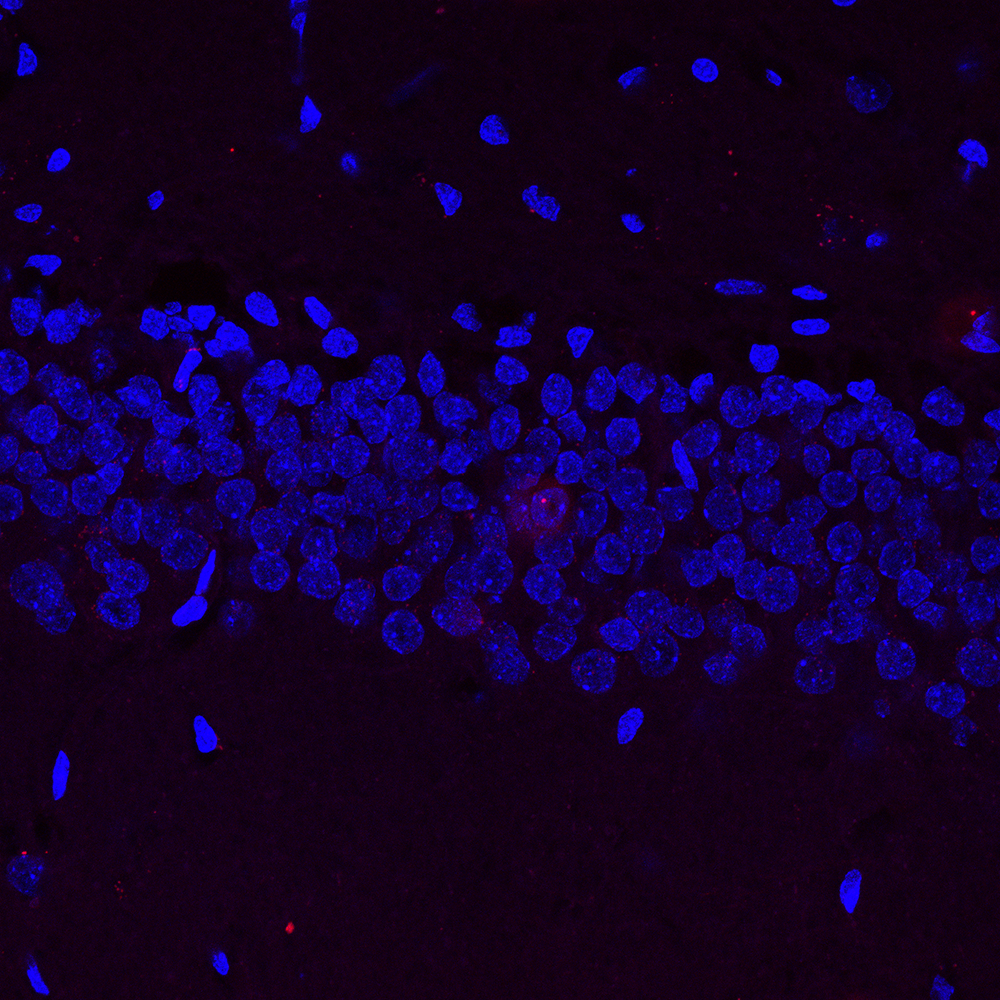

Supplement: Supplementary file 7 — Source data Fig. 5 [file 44321_2024_54_MOESM7_ESM.zip › Figure 5/Figure 5C/CB1R-KO/CB1R-KO CA1.tif]

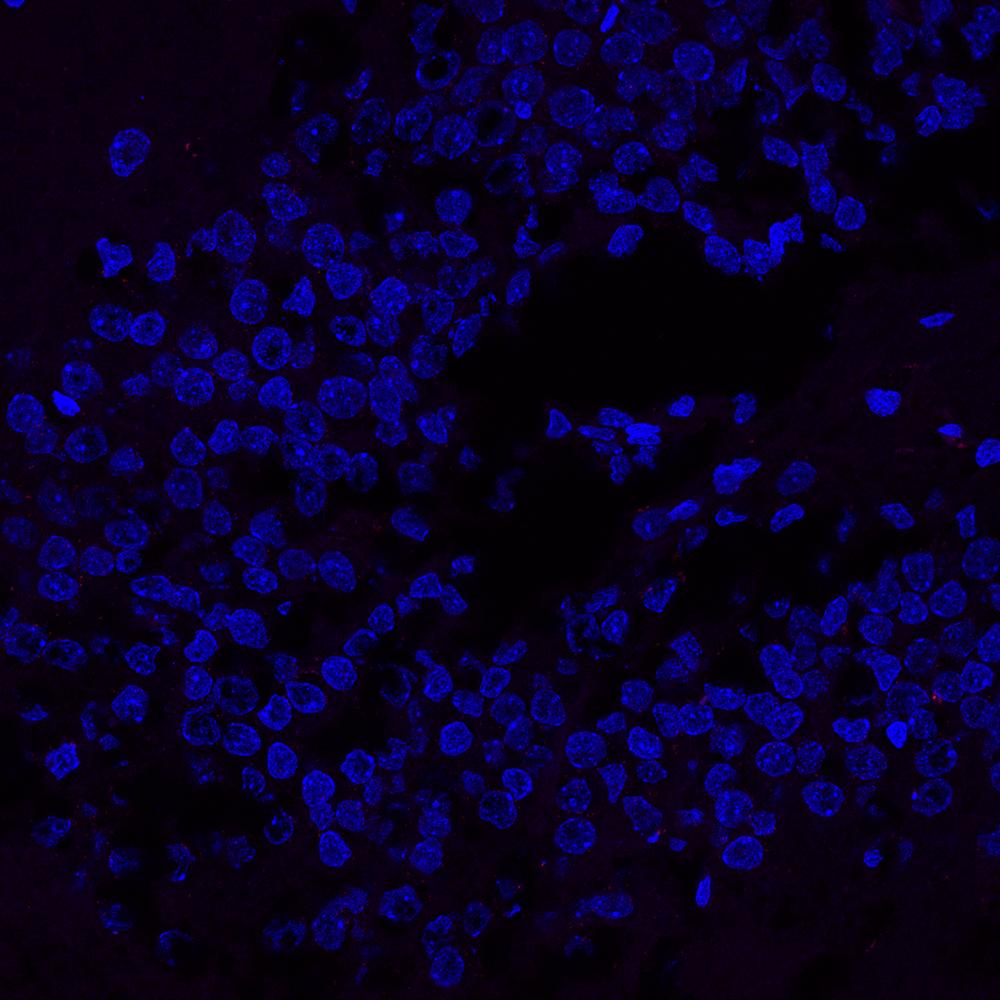

Supplement: Supplementary file 7 — Source data Fig. 5 [file 44321_2024_54_MOESM7_ESM.zip › Figure 5/Figure 5C/CB1R-KO/CB1R-KO CA3.tif]

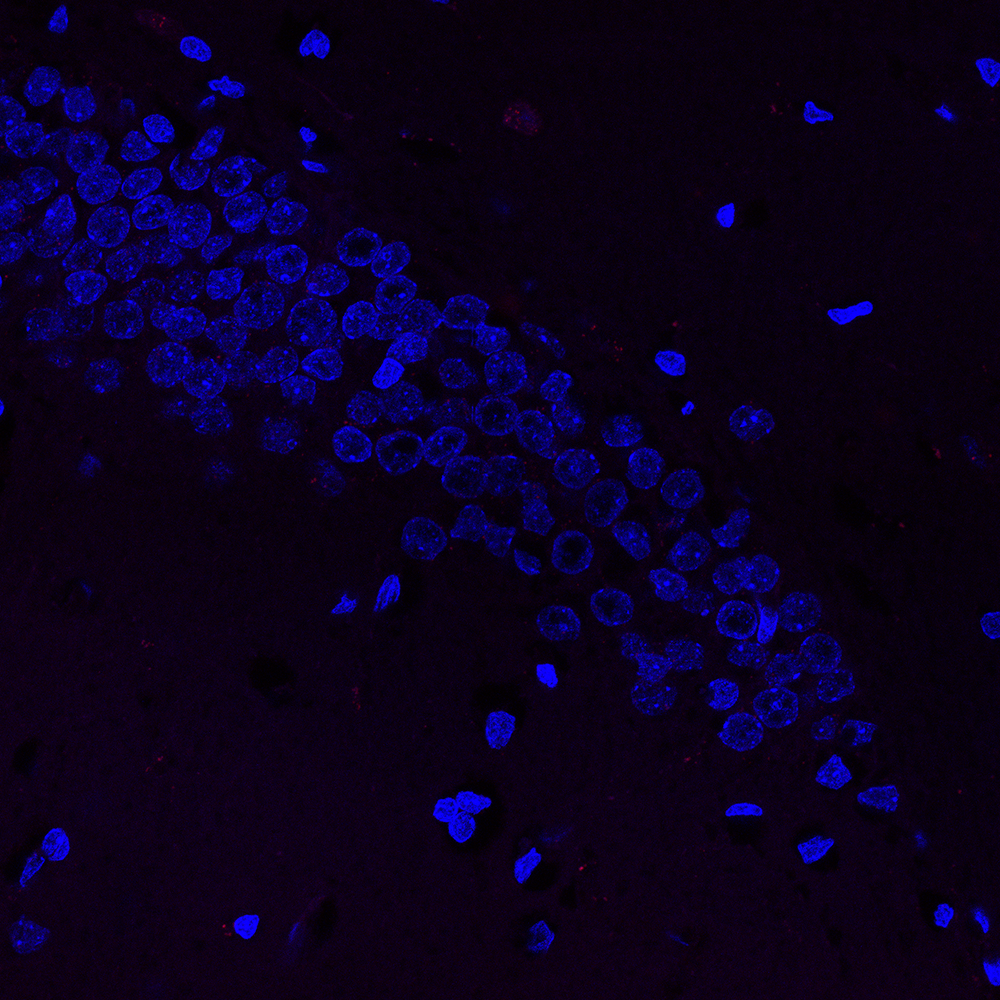

Supplement: Supplementary file 7 — Source data Fig. 5 [file 44321_2024_54_MOESM7_ESM.zip › Figure 5/Figure 5C/CB1R-KO/CB1R-KO Dentate gyrus.tif]

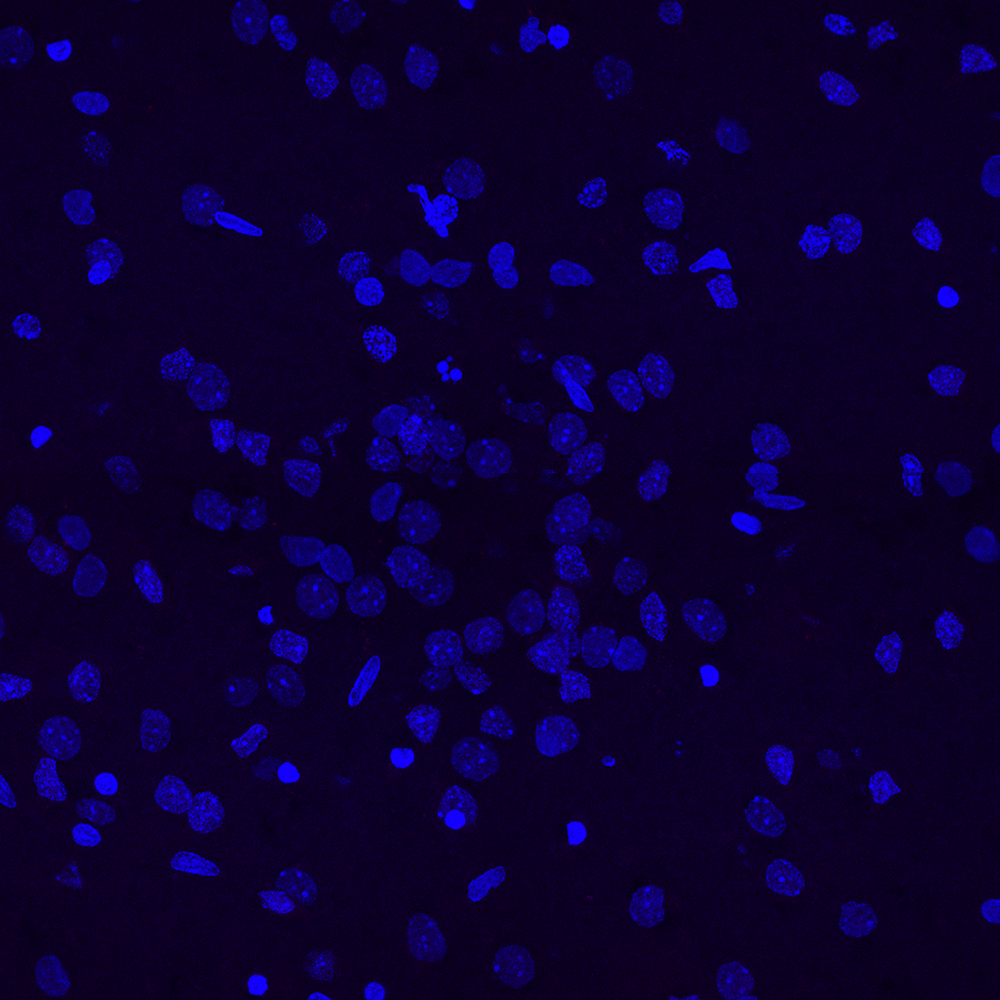

Supplement: Supplementary file 7 — Source data Fig. 5 [file 44321_2024_54_MOESM7_ESM.zip › Figure 5/Figure 5C/CB1R-KO/CB1R-KO Hilus.tif]

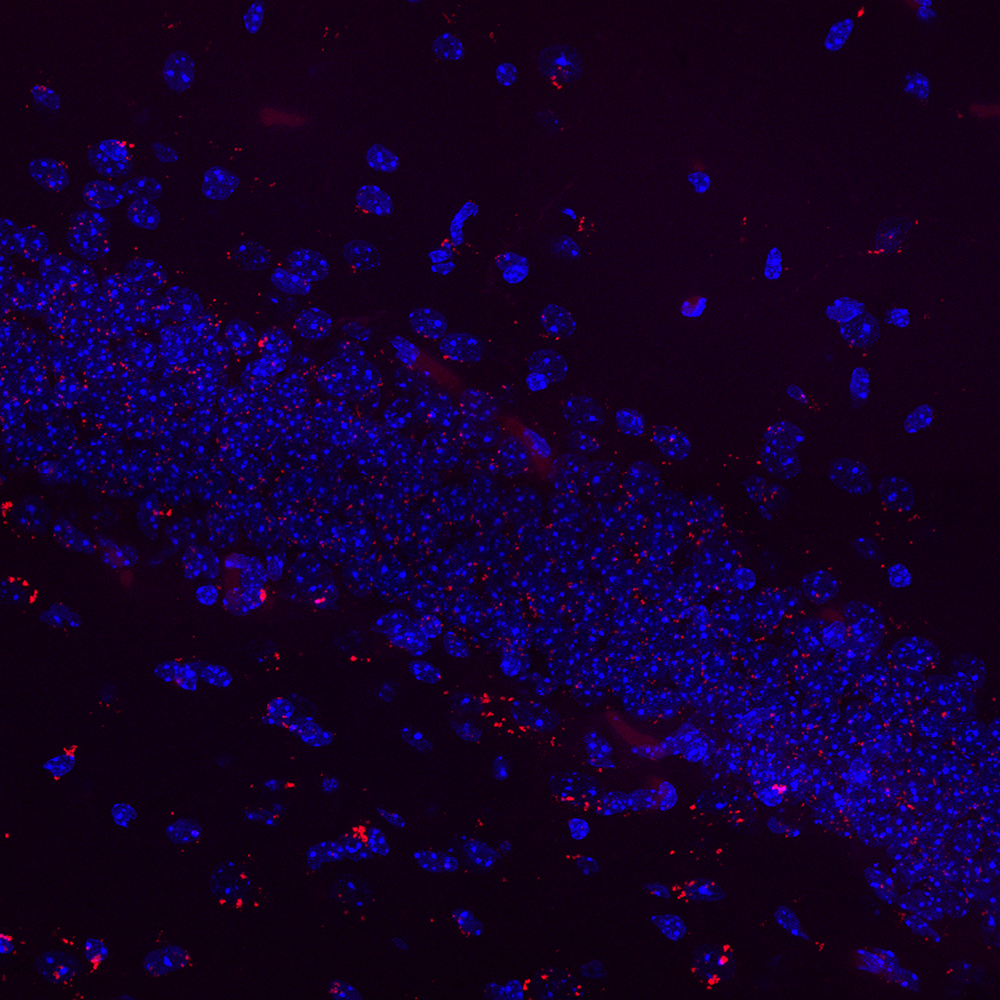

Supplement: Supplementary file 7 — Source data Fig. 5 [file 44321_2024_54_MOESM7_ESM.zip › Figure 5/Figure 5C/CB1R-WT/CB1R-WT CA1.tif]

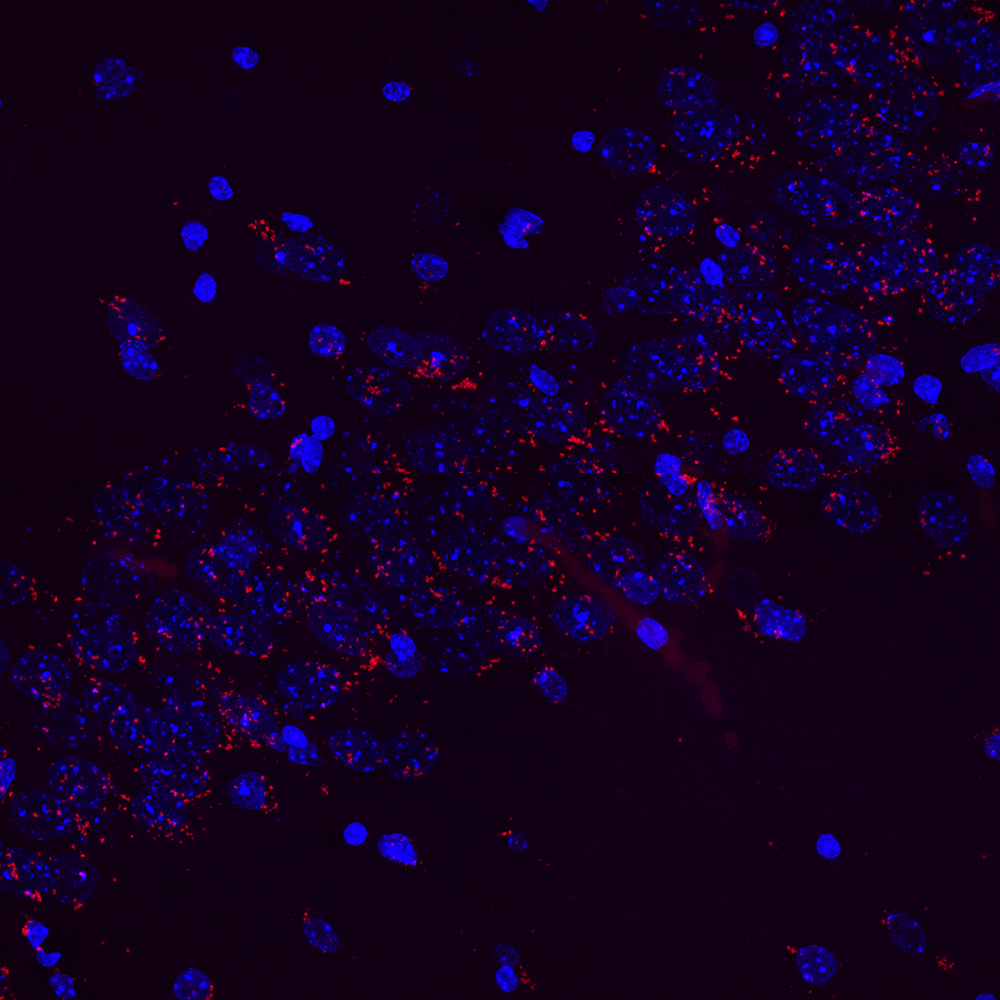

Supplement: Supplementary file 7 — Source data Fig. 5 [file 44321_2024_54_MOESM7_ESM.zip › Figure 5/Figure 5C/CB1R-WT/CB1R-WT CA3.tif]

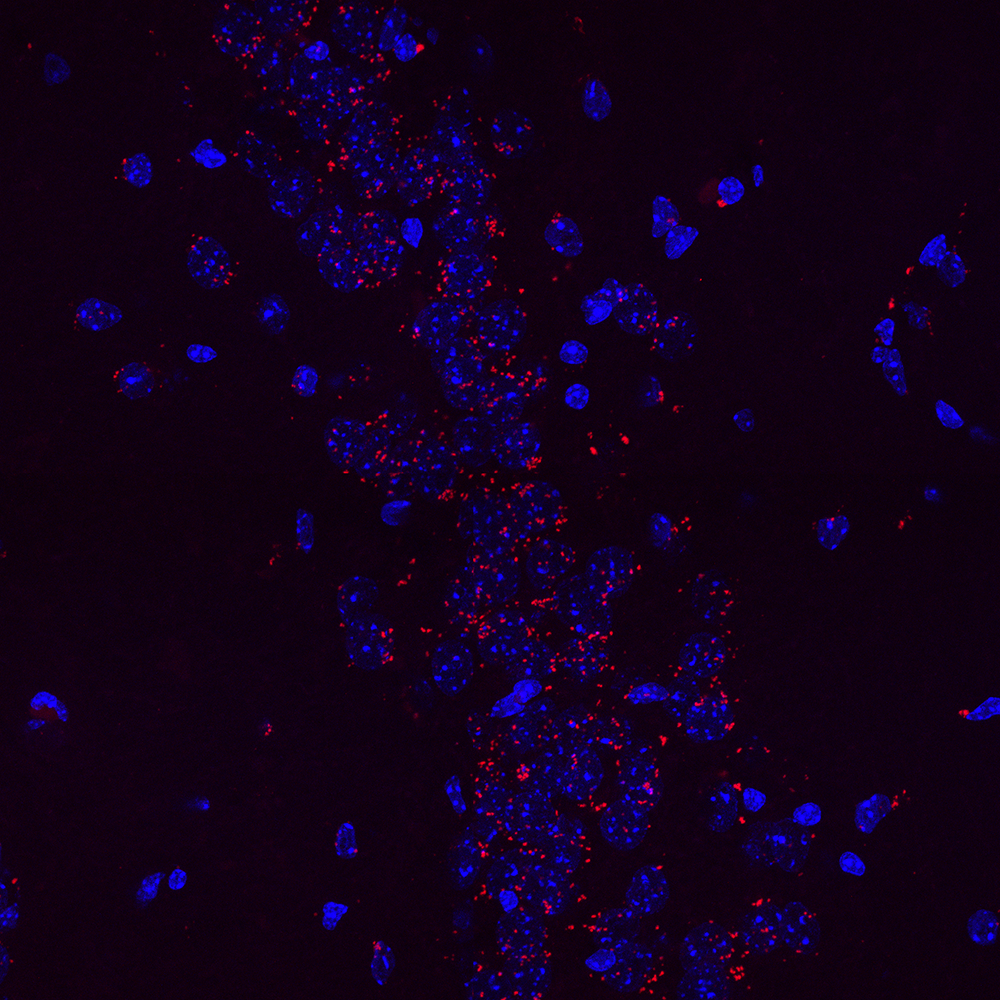

Supplement: Supplementary file 7 — Source data Fig. 5 [file 44321_2024_54_MOESM7_ESM.zip › Figure 5/Figure 5C/CB1R-WT/CB1R-WT Gyrus.tif]

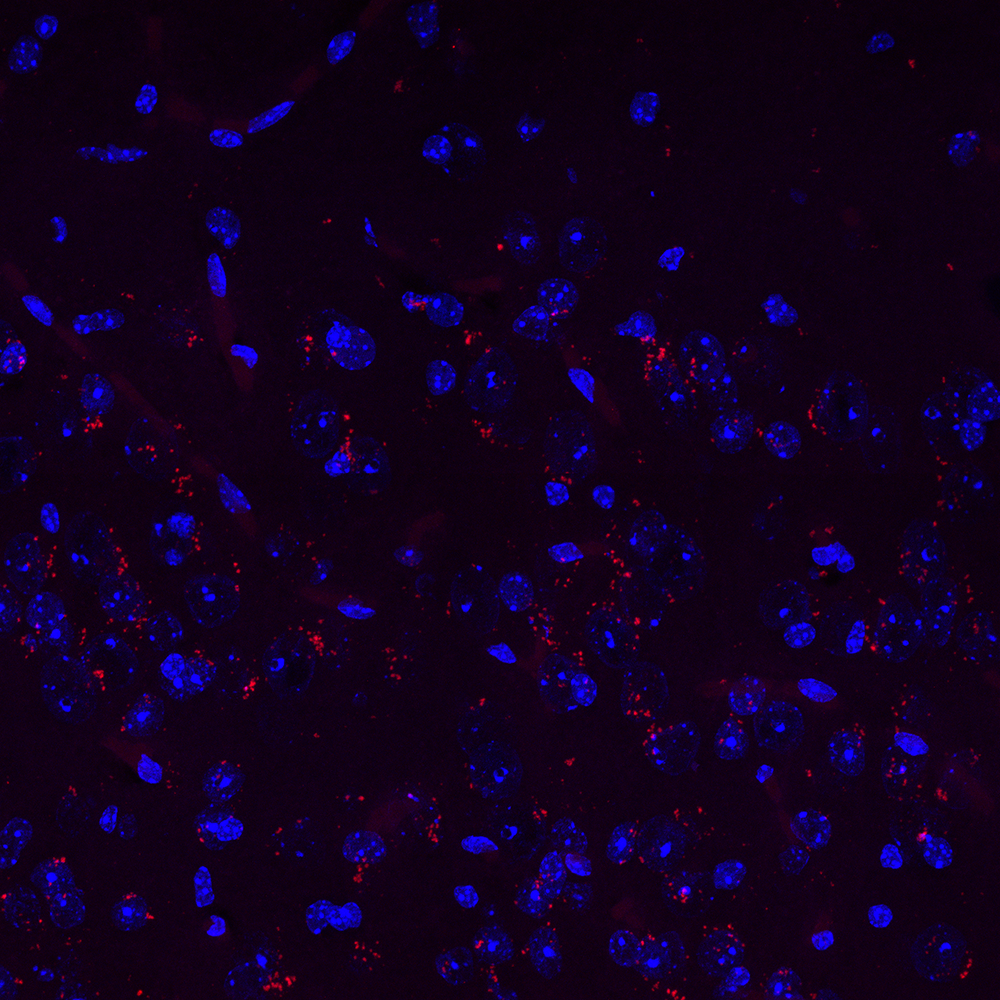

Supplement: Supplementary file 7 — Source data Fig. 5 [file 44321_2024_54_MOESM7_ESM.zip › Figure 5/Figure 5C/CB1R-WT/CB1R-WT Hilus.tif]

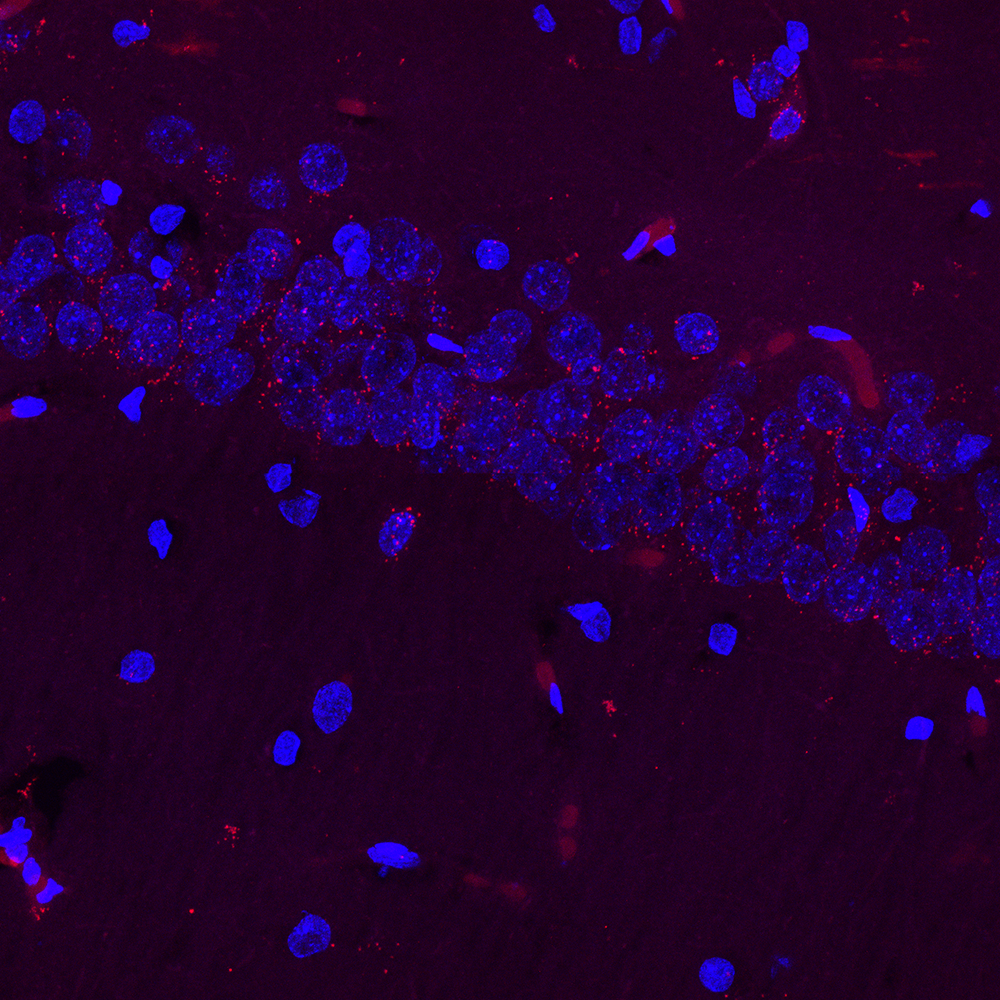

Supplement: Supplementary file 7 — Source data Fig. 5 [file 44321_2024_54_MOESM7_ESM.zip › Figure 5/Figure 5C/GABA-CB1R-KO/GABA-CB1R-KO CA1.tif]

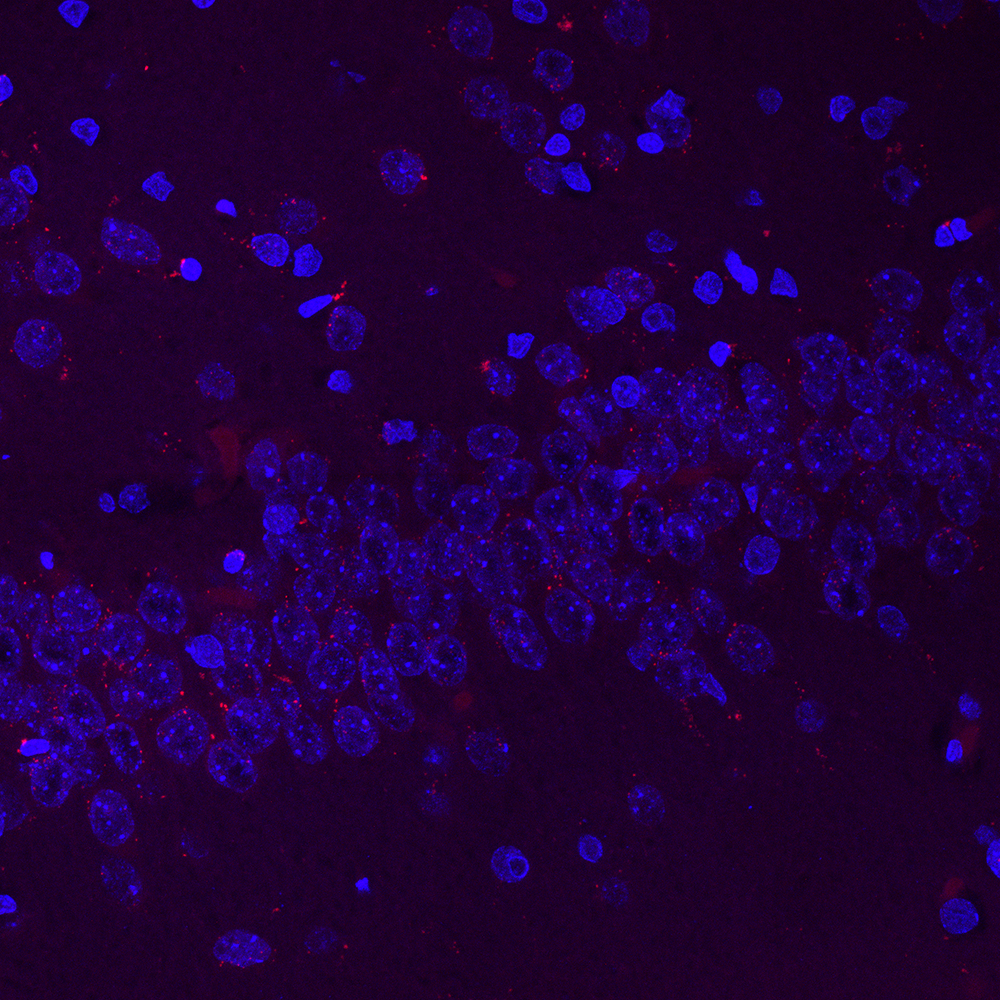

Supplement: Supplementary file 7 — Source data Fig. 5 [file 44321_2024_54_MOESM7_ESM.zip › Figure 5/Figure 5C/GABA-CB1R-KO/GABA-CB1R-KO CA3.tif]

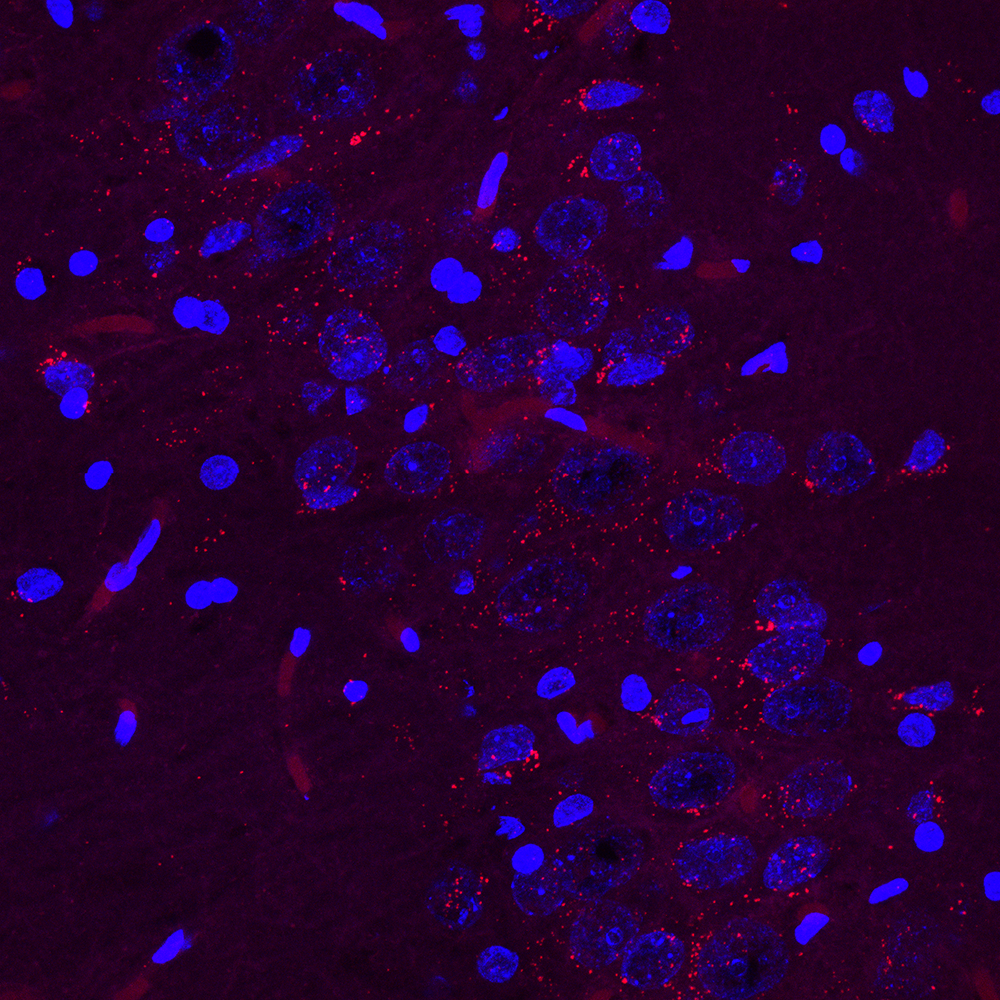

Supplement: Supplementary file 7 — Source data Fig. 5 [file 44321_2024_54_MOESM7_ESM.zip › Figure 5/Figure 5C/GABA-CB1R-KO/GABA-CB1R-KO Dentate gyrus.tif]

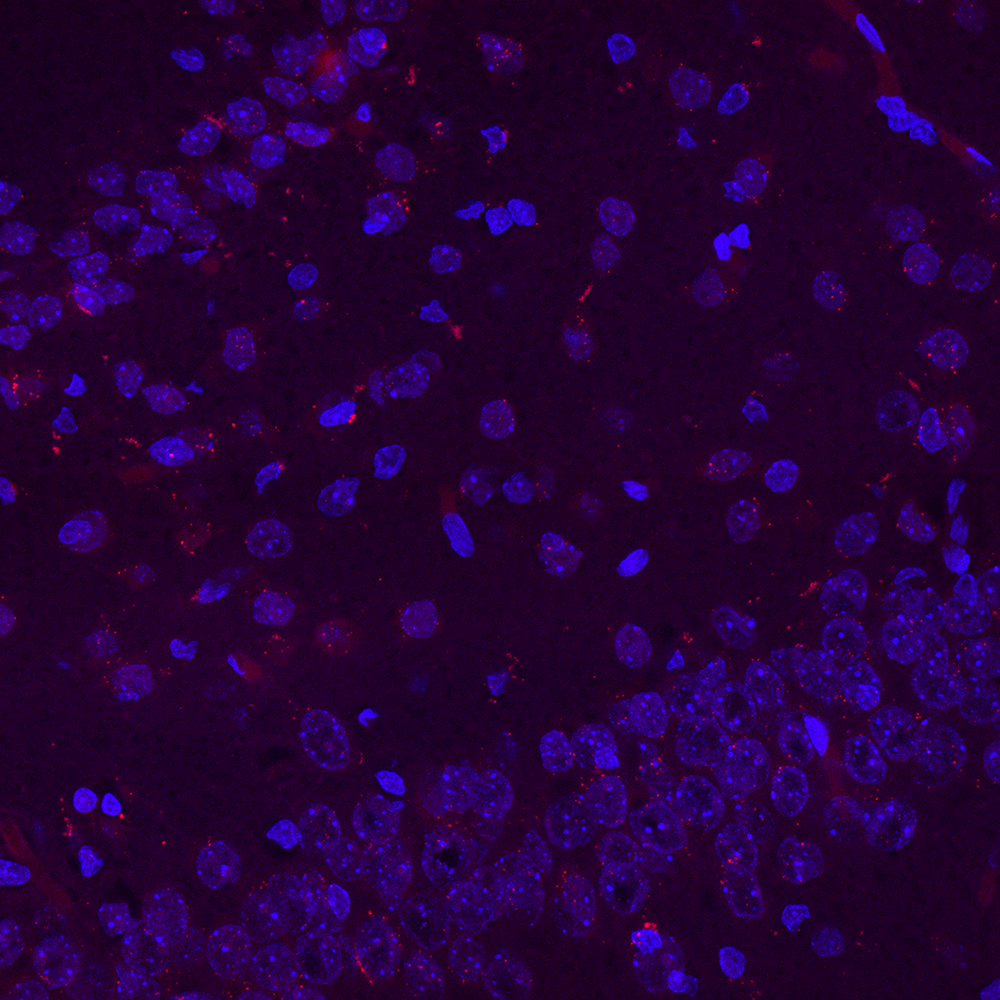

Supplement: Supplementary file 7 — Source data Fig. 5 [file 44321_2024_54_MOESM7_ESM.zip › Figure 5/Figure 5C/GABA-CB1R-KO/GABA-CB1R-KO Hilus.tif]

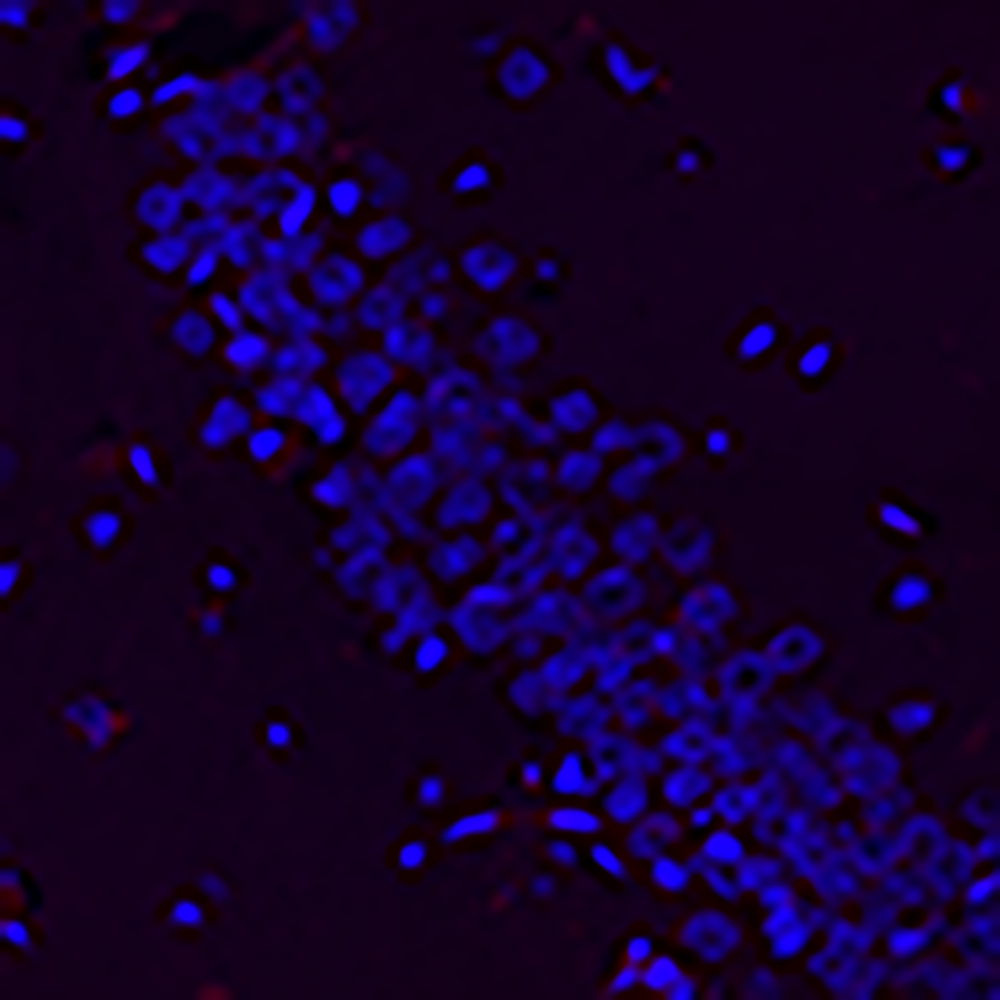

Supplement: Supplementary file 7 — Source data Fig. 5 [file 44321_2024_54_MOESM7_ESM.zip › Figure 5/Figure 5C/Glu-CB1R-KO/Glu-CB1R-KO CA1.tif]

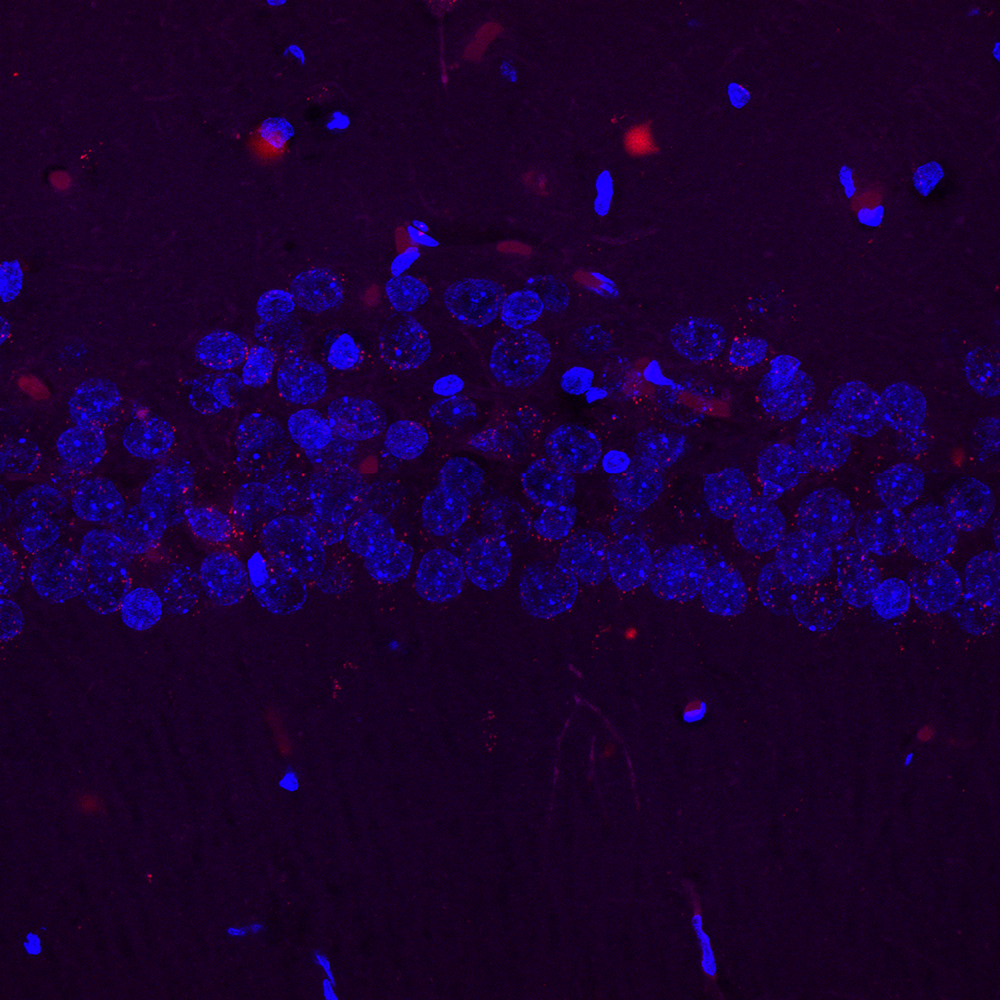

Supplement: Supplementary file 7 — Source data Fig. 5 [file 44321_2024_54_MOESM7_ESM.zip › Figure 5/Figure 5C/Glu-CB1R-KO/Glu-CB1R-KO CA3.tif]

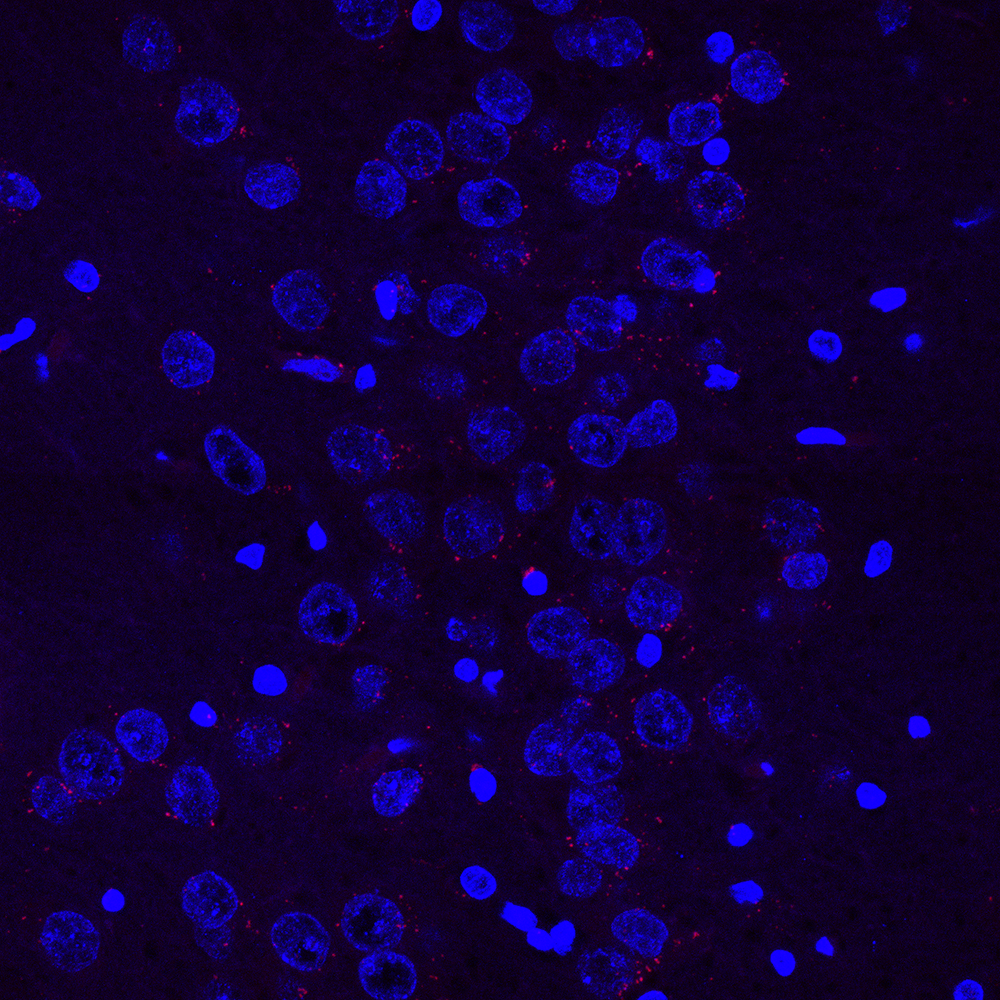

Supplement: Supplementary file 7 — Source data Fig. 5 [file 44321_2024_54_MOESM7_ESM.zip › Figure 5/Figure 5C/Glu-CB1R-KO/Glu-CB1R-KO Dentate gyrus.tif]

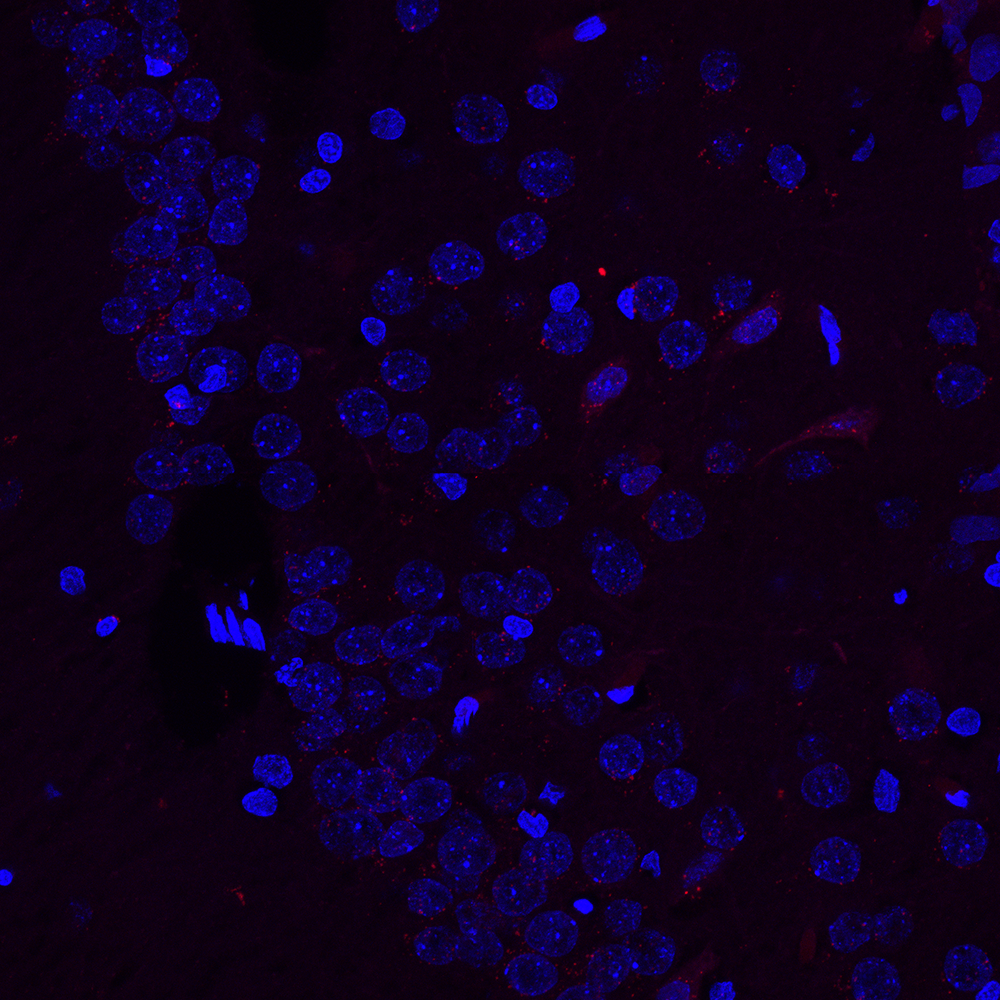

Supplement: Supplementary file 7 — Source data Fig. 5 [file 44321_2024_54_MOESM7_ESM.zip › Figure 5/Figure 5C/Glu-CB1R-KO/Glu-CB1R-KO Hilus.tif]

## Slide 1
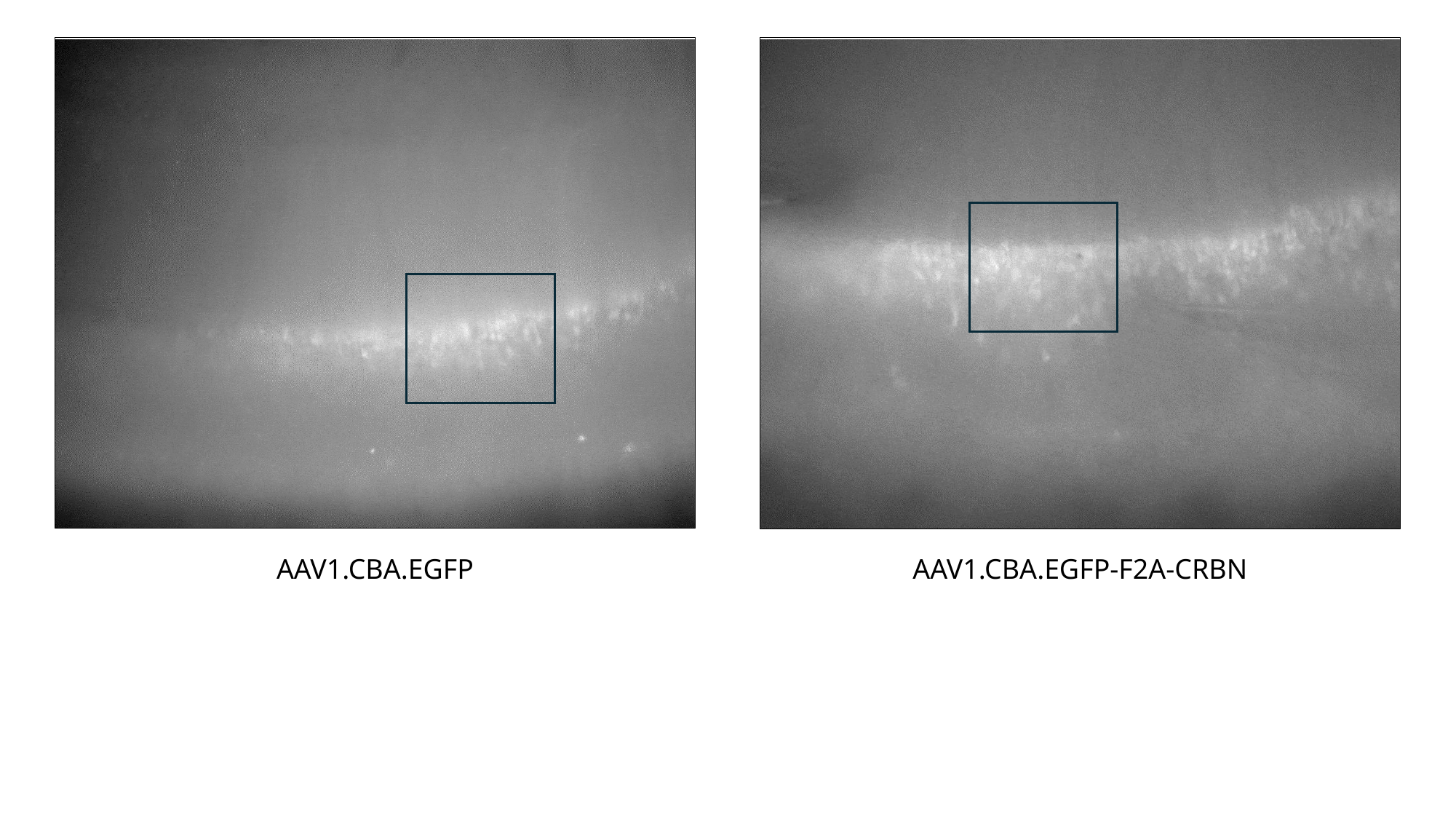

AAV1.CBA.EGFP
AAV1.CBA.EGFP-F2A-CRBN

Supplement: Supplementary file 7 — Source data Fig. 5 [file 44321_2024_54_MOESM7_ESM.zip › Figure 5/Figure 5E/Figure 5E - fluorescence images.pptx]
