# Supplementary material for: The CB1 receptor interacts with cereblon and drives cereblon deficiency-associated memory shortfalls
Source: EMBO Mol Med. 2024 Mar 21;16(4):11. doi: 10.1038/s44321-024-00054-w (PMC11018632; doi:10.1038/s44321-024-00054-w)
Supplement: Supplementary file 3 — Source data Fig. 1 [file 44321_2024_54_MOESM3_ESM.zip › Figure 1/1E/Figure 1E - uncropped WBs.pptx]

## Slide 1
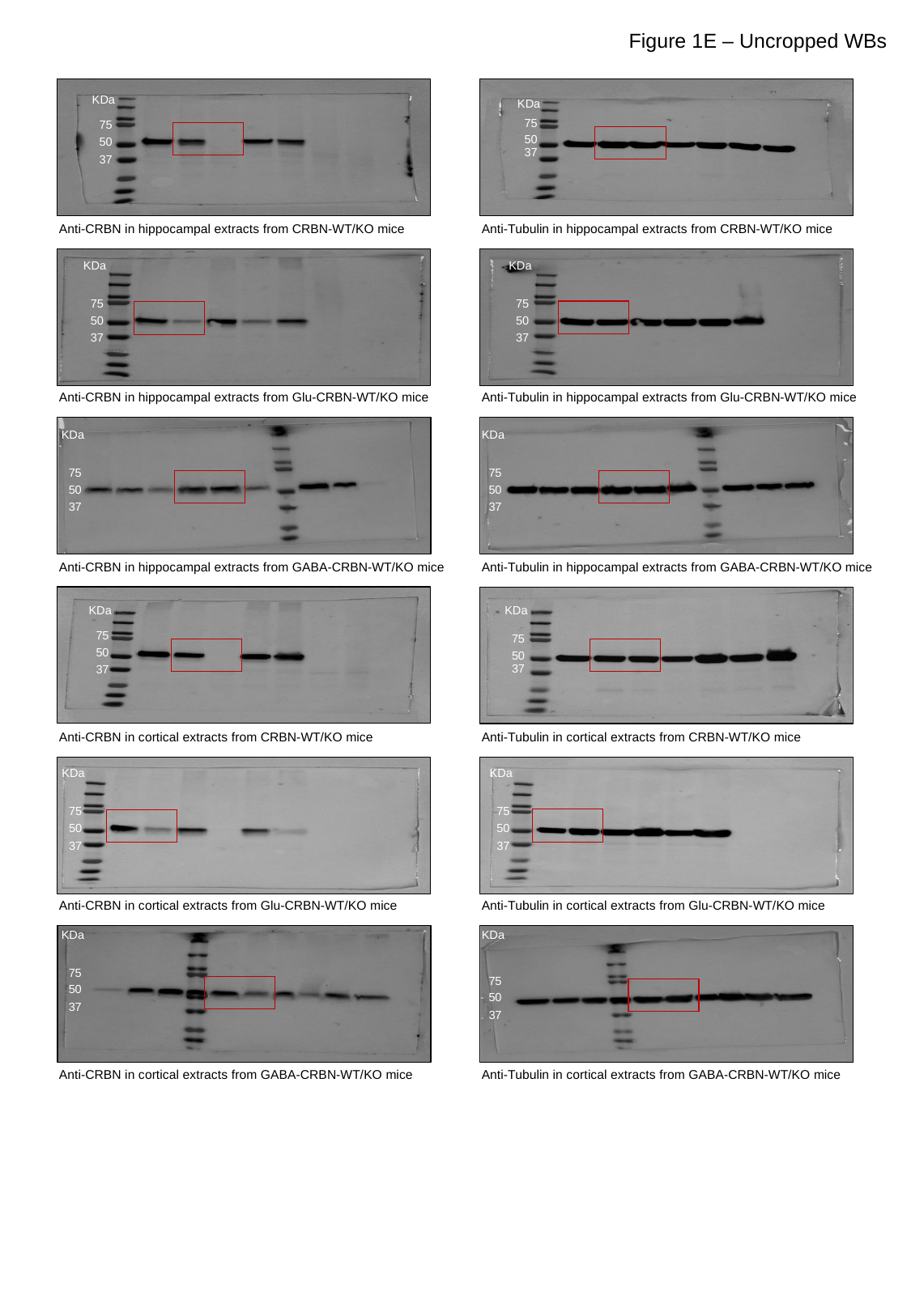

Figure 1E – Uncropped WBs
KDa
75
50
37
KDa
75
50
37
Anti-CRBN in hippocampal extracts from CRBN-WT/KO mice
Anti-Tubulin in hippocampal extracts from CRBN-WT/KO mice
KDa
75
50
37
KDa
75
50
37
Anti-CRBN in hippocampal extracts from Glu-CRBN-WT/KO mice
Anti-Tubulin in hippocampal extracts from Glu-CRBN-WT/KO mice
KDa
75
50
37
KDa
75
50
37
Anti-CRBN in hippocampal extracts from GABA-CRBN-WT/KO mice
Anti-Tubulin in hippocampal extracts from GABA-CRBN-WT/KO mice
KDa
75
50
37
KDa
75
50
37
Anti-CRBN in cortical extracts from CRBN-WT/KO mice
Anti-Tubulin in cortical extracts from CRBN-WT/KO mice
KDa
75
50
37
KDa
75
50
37
Anti-CRBN in cortical extracts from Glu-CRBN-WT/KO mice
Anti-Tubulin in cortical extracts from Glu-CRBN-WT/KO mice
KDa
75
50
37
KDa
75
50
37
Anti-CRBN in cortical extracts from GABA-CRBN-WT/KO mice
Anti-Tubulin in cortical extracts from GABA-CRBN-WT/KO mice
