# Supplementary material for: The CB1 receptor interacts with cereblon and drives cereblon deficiency-associated memory shortfalls
Source: EMBO Mol Med. 2024 Mar 21;16(4):11. doi: 10.1038/s44321-024-00054-w (PMC11018632; doi:10.1038/s44321-024-00054-w)
Supplement: Supplementary file 5 — Source data Fig. 3 [file 44321_2024_54_MOESM5_ESM.zip › Figure 3/Figure 3C/Figure 3C - uncropped WBs.pptx]

## Slide 1
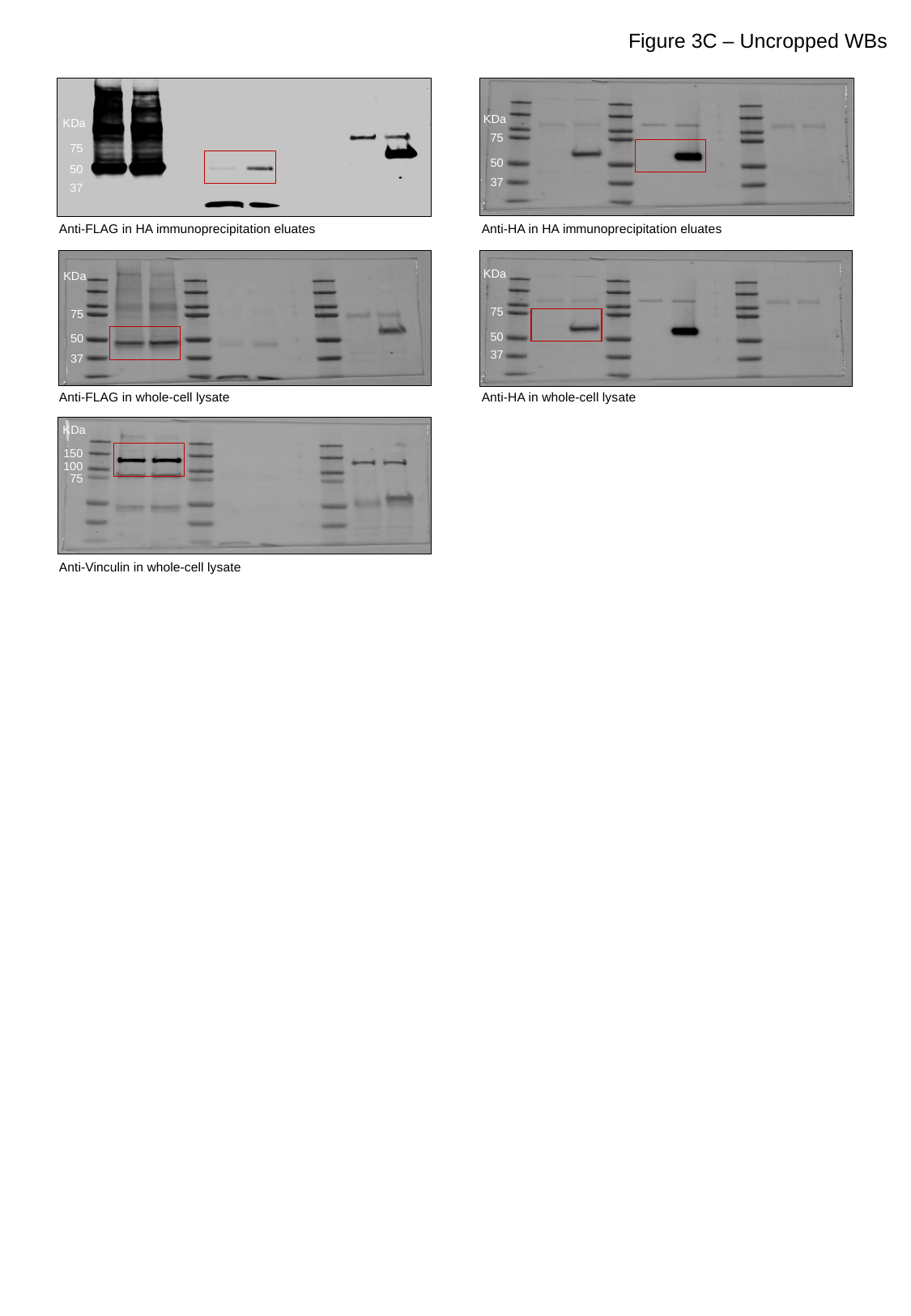

Figure 3C – Uncropped WBs
KDa
75
50
37
KDa
75
50
37
Anti-FLAG in HA immunoprecipitation eluates
Anti-HA in HA immunoprecipitation eluates
KDa
75
50
37
KDa
75
50
37
Anti-FLAG in whole-cell lysate
Anti-HA in whole-cell lysate
KDa
150
100
75
Anti-Vinculin in whole-cell lysate
