# Supplementary material for: The CB1 receptor interacts with cereblon and drives cereblon deficiency-associated memory shortfalls
Source: EMBO Mol Med. 2024 Mar 21;16(4):11. doi: 10.1038/s44321-024-00054-w (PMC11018632; doi:10.1038/s44321-024-00054-w)
Supplement: Supplementary file 6 — Source data Fig. 4 [file 44321_2024_54_MOESM6_ESM.zip › Figure 4/Figure 4I/Figure 4I - uncropped WBs.pptx]

## Slide 1
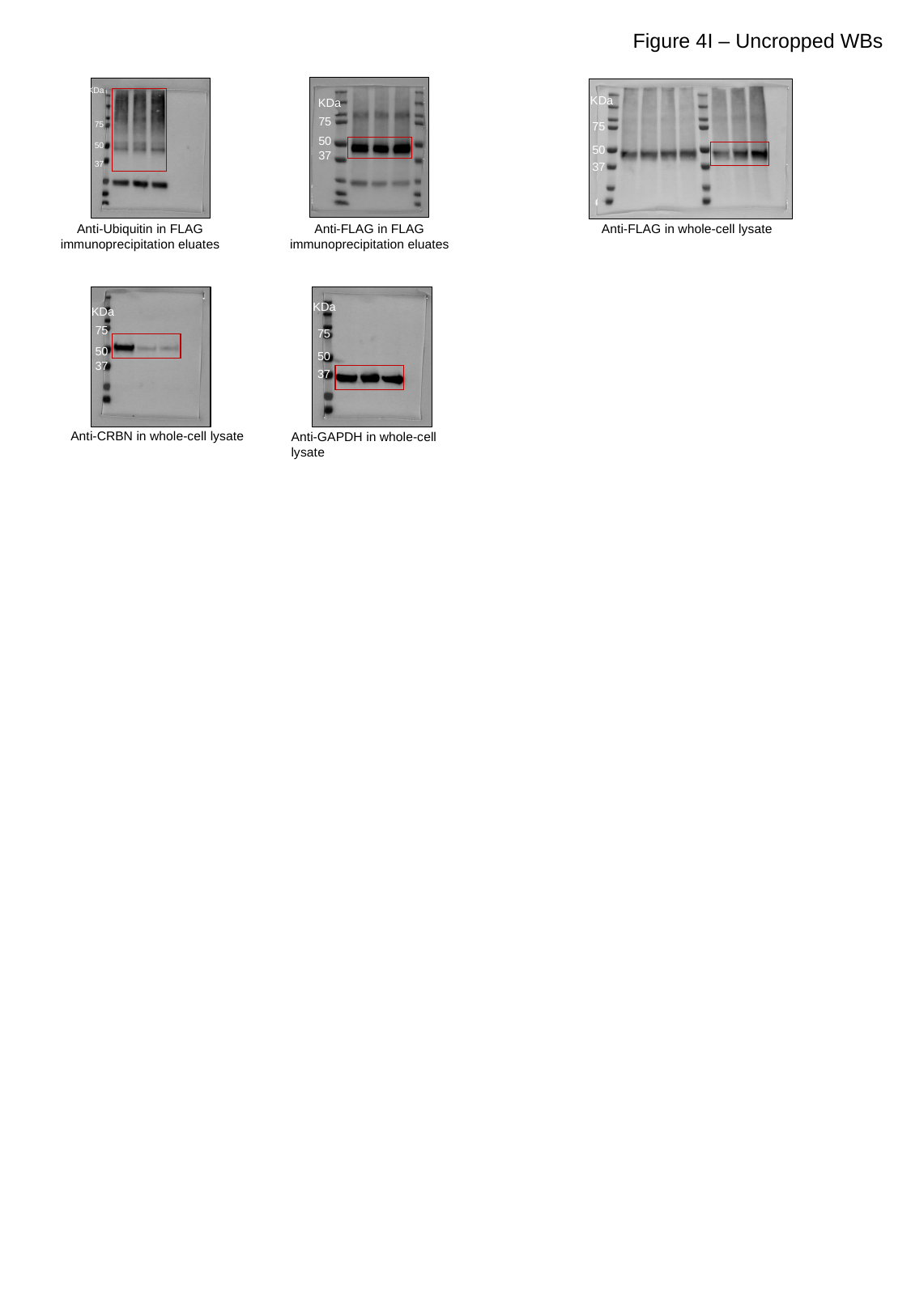

Figure 4I – Uncropped WBs
KDa
75
50
37
KDa
75
50
37
KDa
75
50
37
Anti-Ubiquitin in FLAG immunoprecipitation eluates
Anti-FLAG in FLAG immunoprecipitation eluates
Anti-FLAG in whole-cell lysate
KDa
KDa
75
75
50
50
37
37
Anti-CRBN in whole-cell lysate
Anti-GAPDH in whole-cell lysate
