# Supplementary material for: The CB1 receptor interacts with cereblon and drives cereblon deficiency-associated memory shortfalls
Source: EMBO Mol Med. 2024 Mar 21;16(4):11. doi: 10.1038/s44321-024-00054-w (PMC11018632; doi:10.1038/s44321-024-00054-w)
Supplement: Supplementary file 10 — Expanded View Figures [file 44321_2024_54_MOESM10_ESM.pdf]

Expanded View Figures

Figure EV1. Additional characterization of the conditional CRBN knockout mouse lines.

(**A**) Representative images and fluorescent signal quantification of RNAscope in situ hybridization labelling in the striatum of CRBN-WT ($n = 6$), Glu-CRBN-KO ($n = 5$), GABA-CRBN-KO ($n = 4$) and CRBN-KO ($n = 3$) mice (mean ± SEM). Circles, male mice; triangles, female mice. *p* values were obtained by one-way ANOVA with Dunnett's post hoc test. (**B**) Representative images and fluorescent signal quantification of RNAscope in situ hybridization labelling in the cerebellum of CRBN-WT ($n = 6$), Glu-CRBN-KO ($n = 5$), GABA-CRBN-KO ($n = 3$) and CRBN-KO ($n = 3$) mice (mean ± SEM). Circles, male mice; triangles, female mice. *p* values were obtained by one-way ANOVA with Dunnett's post hoc test. (**C**) *Crbn* mRNA levels (% of WT mice) as assessed by q-PCR in the striatum or cerebellum of CRBN-WT, CRBN-KO, Glu-CRBN-WT, Glu-CRBN-KO, GABA-CRBN-WT and GABA-CRBN-KO mice ($n = 3$ animals per group; mean ± SEM). Circles, male mice; triangles, female mice. *p* values were obtained by unpaired two-tailed Student's *t* test. (**D**) CRBN protein levels (% of WT mice) as assessed by western blotting in the striatum or cerebellum of CRBN-WT, CRBN-KO, Glu-CRBN-WT, Glu-CRBN-KO, GABA-CRBN-WT and GABA-CRBN-KO mice ($n = 6$–8 animals per group; mean ± SEM). Circles, male mice; triangles, female mice. *p* values were obtained by unpaired two-tailed Student's *t* test. Source data are available online for this figure.

© The Author(s)

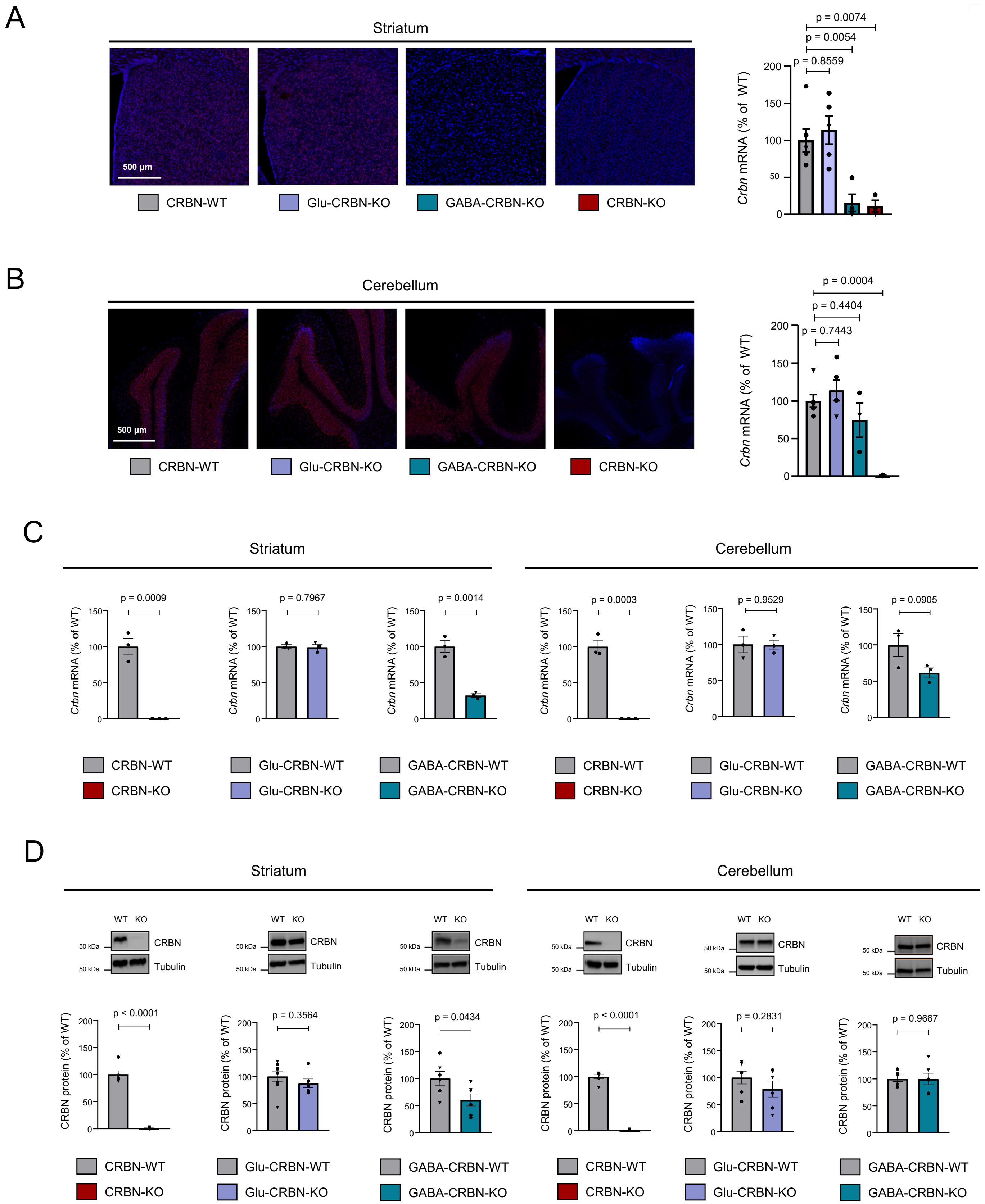
A
Striatum
500 µm
CRBN-WT
Glu-CRBN-KO
GABA-CRBN-KO
CRBN-KO
p = 0.0074
p = 0.0054
p = 0.8559
Crbn mRNA (% of WT)
B
Cerebellum
500 µm
p = 0.0004
p = 0.4404
p = 0.7443
C
Striatum
Cerebellum
p = 0.0009
p = 0.7967
p = 0.0014
p = 0.0003
p = 0.9529
p = 0.0905
Glu-CRBN-WT
GABA-CRBN-WT
D
WT KO
CRBN
Tubulin
50 kDa
p < 0.0001
p = 0.3564
p = 0.0434
p < 0.0001
p = 0.2831
p = 0.9667
CRBN protein (% of WT)

 © The Author(s)

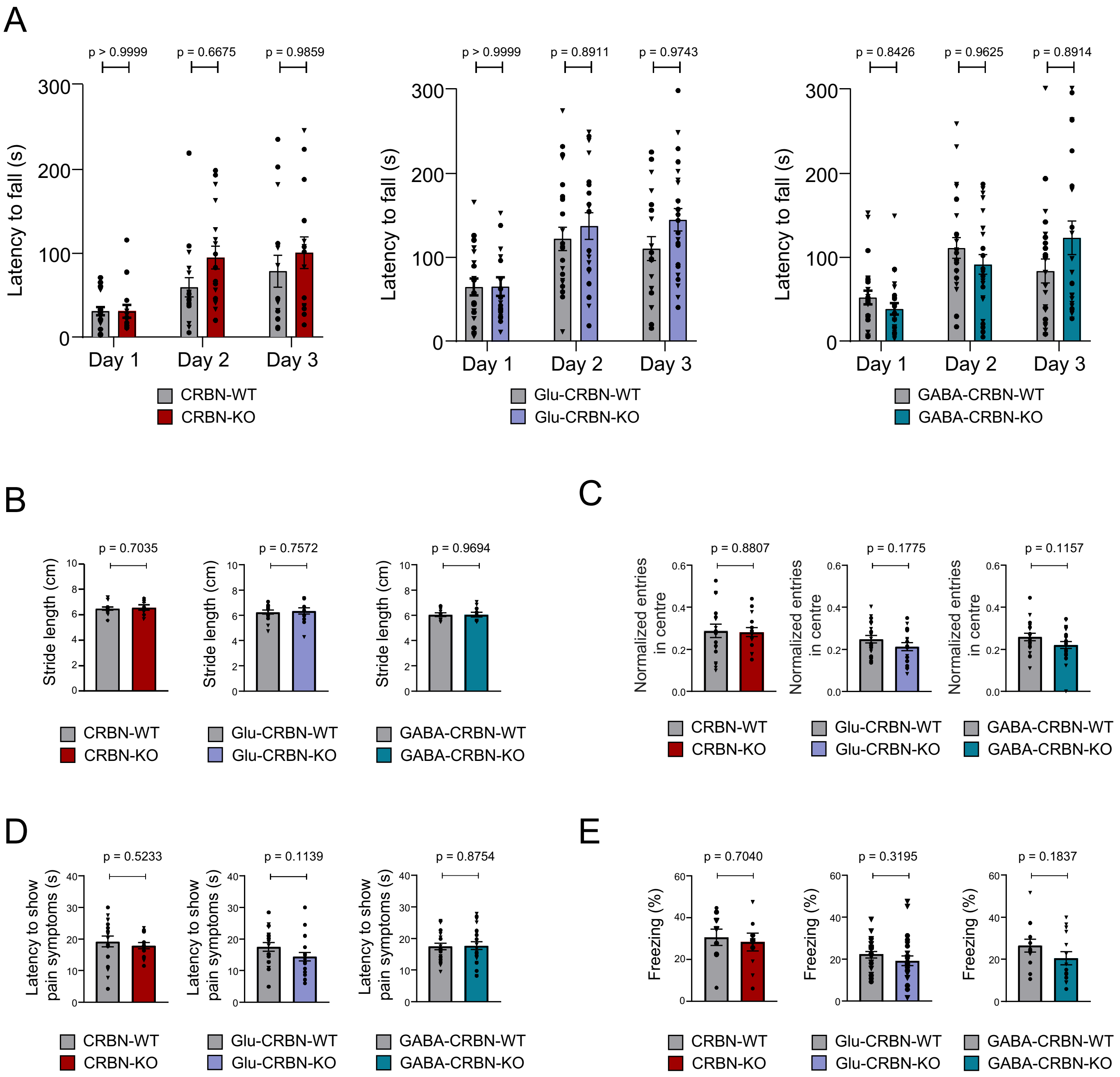

Figure EV2. Additional behavioural phenotyping of the CRBN knockout mouse lines.

(**A**) Motor learning in the rotarod test (mean time to fall from the apparatus on day 1, 2 or 3; in s). CRBN-WT ($n = 18$), CRBN-KO ($n = 15$), Glu-CRBN-WT ($n = 20$), Glu-CRBN-KO ($n = 24$), GABA-CRBN-WT ($n = 24$), GABA-CRBN-KO ($n = 24$) mice (mean ± SEM). Circles, male mice; triangles, female mice. *p* values were obtained by two-way ANOVA with Sidak's post hoc test. (**B**) Stride length (in cm) in the footprint test. CRBN-WT (n = 13), CRBN-KO ($n = 8$), Glu-CRBN-WT ($n = 17$), Glu-CRBN-KO ($n = 13$), GABA-CRBN-WT ($n = 9$), GABA-CRBN-KO ($n = 11$) mice (mean ± SEM). Circles, male mice; triangles, female mice. *p* values were obtained by unpaired two-tailed Student's *t* test. (**C**) Entries in the central part of an open field arena (normalized to total ambulation). CRBN-WT ($n = 18$), CRBN-KO ($n = 15$), Glu-CRBN-WT ($n = 20$), Glu-CRBN-KO ($n = 19$), GABA-CRBN-WT ($n = 19$), GABA-CRBN-KO ($n = 24$) mice (mean ± SEM). Circles, male mice; triangles, female mice. *p* values were obtained by unpaired two-tailed Student's *t* test. (**D**) Time to show pain symptoms (in s) in the hot plate test. CRBN-WT ($n = 18$), CRBN-KO ($n = 15$), Glu-CRBN-WT ($n = 20$), Glu-CRBN-KO ($n = 19$), GABA-CRBN-WT ($n = 21$), GABA-CRBN-KO ($n = 24$) mice (mean ± SEM). Circles, male mice; triangles, female mice. *p* values were obtained by unpaired two-tailed Student's *t* test. (**E**) Time (in %) spent freezing in the conditioning session of the fear conditioning protocol. CRBN-WT ($n = 10$), CRBN-KO ($n = 10$), Glu-CRBN-WT ($n = 24$), Glu-CRBN-KO ($n = 24$), GABA-CRBN-WT ($n = 13$), GABA-CRBN-KO ($n = 14$) mice (mean ± SEM). Circles, male mice; triangles, female mice. *p* values were obtained by unpaired two-tailed Student's *t* test. Source data are available online for this figure.

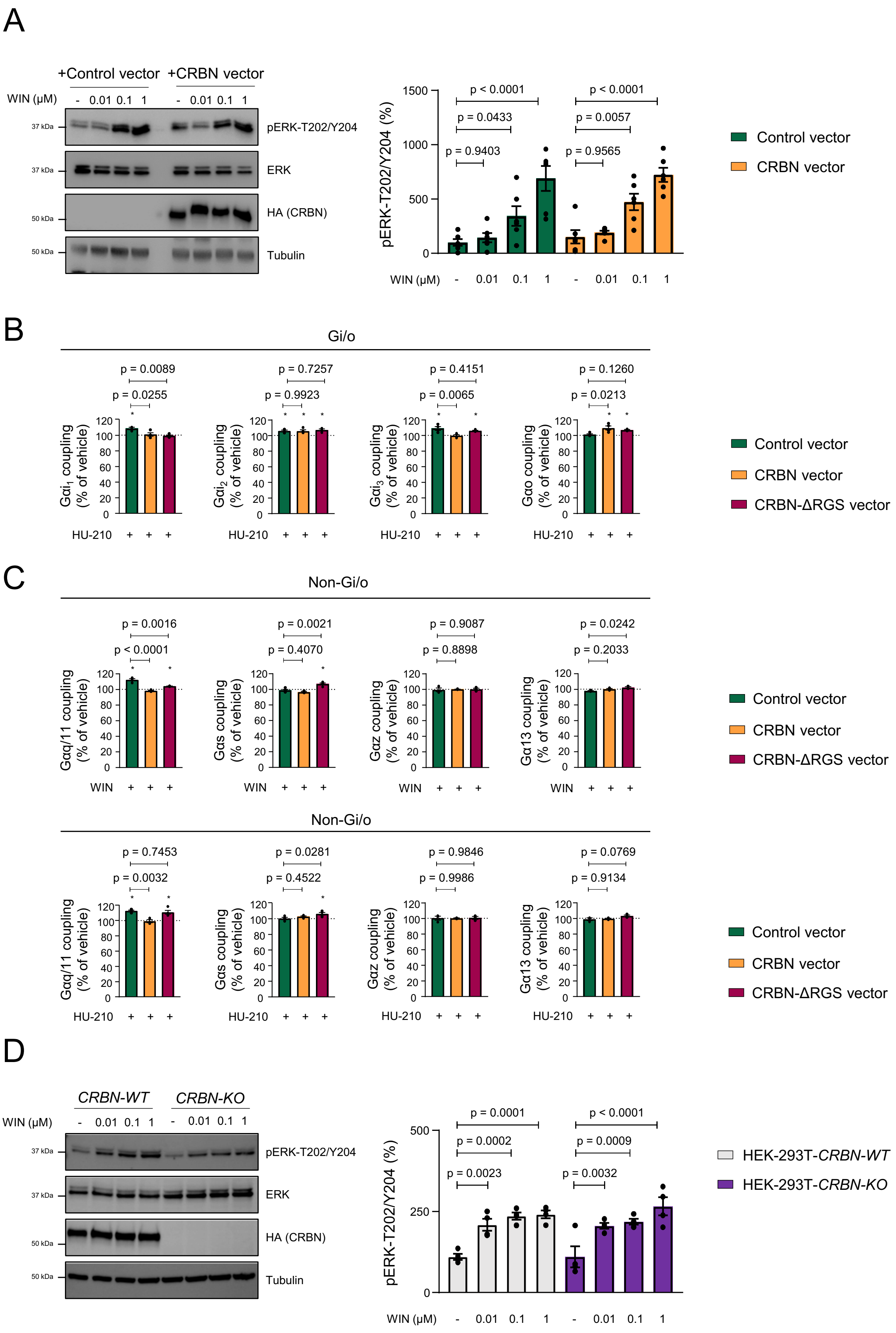
A
+Control vector
+CRBN vector
WIN (μM) - 0.01 0.1 1 - 0.01 0.1 1
37 kDa
pERK-T202/Y204
37 kDa
ERK
50 kDa
HA (CRBN)
50 kDa
Tubulin
pERK-T202/Y204 (%)
p < 0.0001
p = 0.0433
p = 0.9403
p < 0.0001
p = 0.0057
p = 0.9565
Control vector
CRBN vector
B
Gi/o
p = 0.0089
p = 0.0255
p = 0.7257
p = 0.9923
p = 0.4151
p = 0.0065
p = 0.1260
p = 0.0213
Gαi1 coupling (% of vehicle)
Gαi2 coupling (% of vehicle)
Gαi3 coupling (% of vehicle)
Gαo coupling (% of vehicle)
HU-210 + + +
Control vector
CRBN vector
CRBN-ΔRGS vector
C
Non-Gi/o
p = 0.0016
p < 0.0001
p = 0.0021
p = 0.4070
p = 0.9087
p = 0.8898
p = 0.0242
p = 0.2033
Gαq/11 coupling (% of vehicle)
Gαs coupling (% of vehicle)
Gαz coupling (% of vehicle)
Gα13 coupling (% of vehicle)
WIN + + +
Control vector
CRBN vector
CRBN-ΔRGS vector
Non-Gi/o
p = 0.7453
p = 0.0032
p = 0.0281
p = 0.4522
p = 0.9846
p = 0.9986
p = 0.0769
p = 0.9134
HU-210 + + +
Control vector
CRBN vector
CRBN-ΔRGS vector
D
CRBN-WT
CRBN-KO
WIN (μM) - 0.01 0.1 1 - 0.01 0.1 1
pERK-T202/Y204
ERK
HA (CRBN)
Tubulin
pERK-T202/Y204 (%)
p = 0.0001
p = 0.0002
p = 0.0023
p < 0.0001
p = 0.0009
p = 0.0032
HEK-293T-CRBN-WT
HEK-293T-CRBN-KO

 © The Author(s)

Figure EV3. Additional data on the CRBN-mediated inhibition of CB_1R-evoked $G_{i/o}$ protein signalling in vitro.

(**A**) HEK-293T cells expressing CB_1R, together or not with CRBN, were incubated for 10 min with vehicle or WIN55,212-2 (doses ranging from 0.01 to 1 μM), and cell extracts were blotted for ERK phosphorylation (mean ± SEM). A representative experiment is shown. *p* values were obtained by two-way ANOVA with Dunnett's multiple comparisons test ($n = 6$ independent experiments). (**B**) Coupling of CB_1R to $G\alpha_{i/o}$ proteins in membrane extracts from HEK-293T cells expressing CB_1R, together or not with CRBN or CRBN-ΔRGS, after HU-210 stimulation (10 μM) (mean ± SEM). *$p < 0.05$ from basal (dashed line) by one-sample Student's *t* test. *p* values between constructs were obtained by one-way ANOVA with Dunnett's multiple comparisons test ($n = 3$–4 independent experiments). (**C**) Coupling of CB_1R to non-$G\alpha_{i/o}$ proteins in membrane extracts from HEK-293T cells expressing CB_1R, together or not with CRBN or CRBN-ΔRGS, after WIN55,212-2 stimulation (10 μM) (mean ± SEM). *$p < 0.05$ from basal (dashed line) by one-sample Student's *t* test. *p* values between constructs were obtained by one-way ANOVA with Dunnett's multiple comparisons test ($n = 3$–4 independent experiments). (**D**) HEK-293T-*CRBN-WT* and HEK-293T-*CRBN-KO* cells expressing CB_1R were incubated for 10 min with vehicle or WIN55,212-2 (doses ranging from 0.01 to 1 μM), and cell extracts were blotted for ERK phosphorylation (mean ± SEM). A representative experiment is shown. p values were obtained by two-way ANOVA with Dunnett's multiple comparisons test ($n = 4$ independent experiments). Source data are available online for this figure.

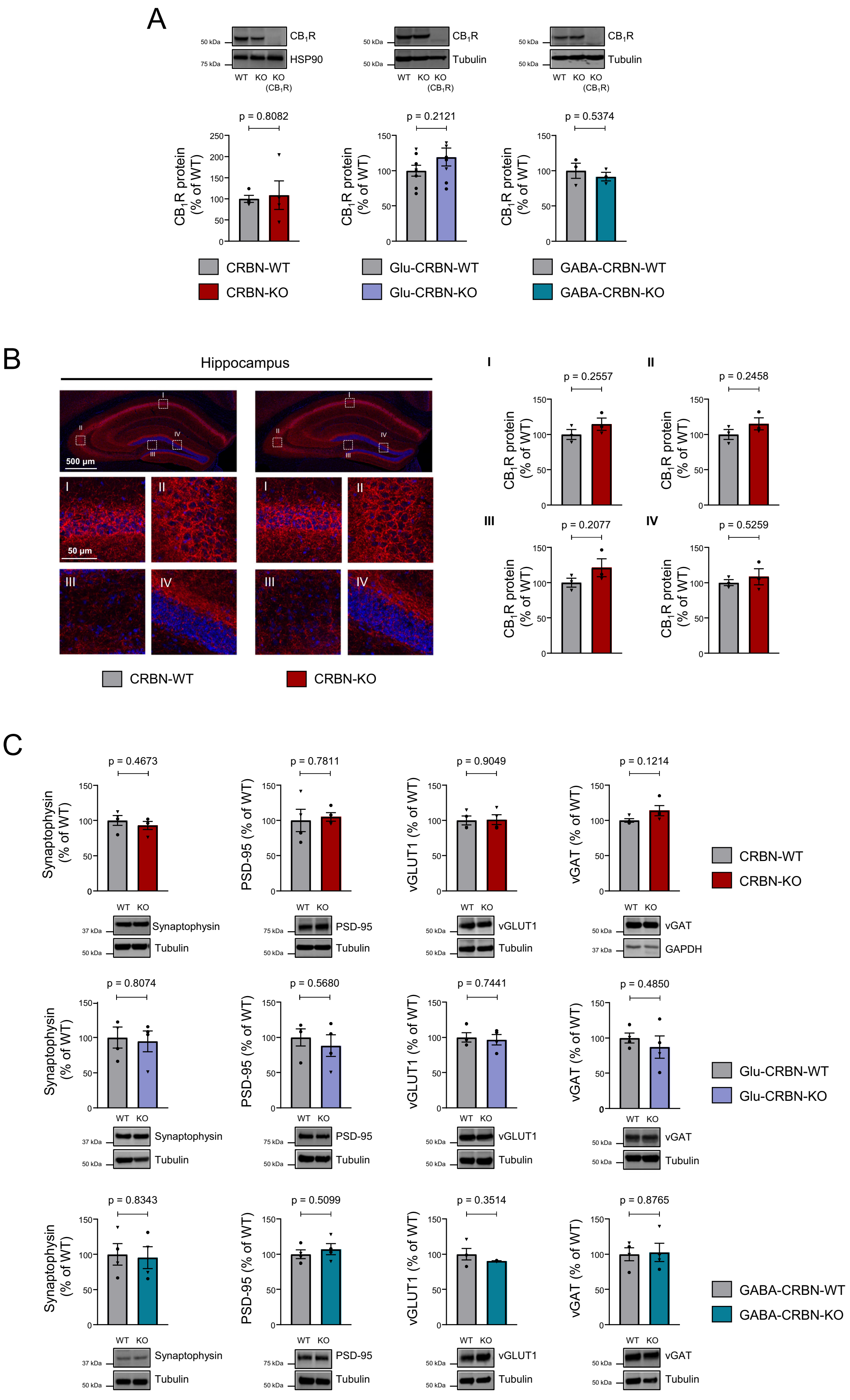
A
CB1R
HSP90
Tubulin
50 kDa
75 kDa
WT KO KO (CB1R)
p = 0.8082
p = 0.2121
p = 0.5374
CB1R protein (% of WT)
CRBN-WT
CRBN-KO
Glu-CRBN-WT
Glu-CRBN-KO
GABA-CRBN-WT
GABA-CRBN-KO
B
Hippocampus
500 µm
50 µm
I
II
III
IV
p = 0.2557
p = 0.2458
p = 0.2077
p = 0.5259
CB1R protein (% of WT)
CRBN-WT
CRBN-KO
C
p = 0.4673
p = 0.7811
p = 0.9049
p = 0.1214
p = 0.8074
p = 0.5680
p = 0.7441
p = 0.4850
p = 0.8343
p = 0.5099
p = 0.3514
p = 0.8765
Synaptophysin (% of WT)
PSD-95 (% of WT)
vGLUT1 (% of WT)
vGAT (% of WT)
WT KO
Synaptophysin
PSD-95
vGLUT1
vGAT
Tubulin
GAPDH
37 kDa
50 kDa
75 kDa
CRBN-WT
CRBN-KO
Glu-CRBN-WT
Glu-CRBN-KO
GABA-CRBN-WT
GABA-CRBN-KO

 © The Author(s)

Figure EV4. *Crbn* deletion does not alter the levels of CB_1R and synapse-marker proteins in the mouse hippocampus.

(**A**) CB_1R protein levels (% of WT mice) as assessed by western blotting in the hippocampus of CRBN-WT ($n = 4$), CRBN-KO ($n = 4$), Glu-CRBN-WT ($n = 8$), Glu-CRBN-KO ($n = 8$), GABA-CRBN-WT ($n = 3$) or GABA-CRBN-KO ($n = 3$) mice (mean ± SEM). Circles, male mice; triangles, female mice. *p* values were obtained by unpaired two-tailed Student's *t* test. (**B**) CB_1R immunoreactivity (% of WT mice) in the hippocampus of CRBN-WT and CRBN-KO mice ($n = 3$ animals per group). High magnification images of CA1 (I), CA3 (II), hilus (III) and granule cell layer of the dentate gyrus (IV) are shown (mean ± SEM). Circles, male mice; triangles, female mice. *p* values were obtained by unpaired two-tailed Student's *t* test. (**C**) Synaptophysin, PSD-95, vGLUT1 and vGAT protein levels (% of WT mice) as assessed by western blotting in the hippocampus of CRBN-WT, CRBN-KO, Glu-CRBN-WT, Glu-CRBN-KO, GABA-CRBN-WT or GABA-CRBN-KO mice ($n = 3$–4 animals per group; mean ± SEM). Circles, male mice; triangles, female mice. p values were obtained by unpaired two-tailed Student's *t* test. Source data are available online for this figure.

© The Author(s)
